# A comparative benchmark of DeepSeek-R1 on the USMLE: surpassing human and AI performance averages

**DOI:** 10.1016/j.clinsp.2026.101021

**Published:** 2026-06-15

**Authors:** Yuchen Zhou, Weiping Wang, Xianhe Zhao, Ke Hu

**Affiliations:** a Department of Radiation Oncology, Peking Union Medical College Hospital, Chinese Academy of Medical Sciences & Peking Union Medical College, Beijing, China; b Tsinghua Medicine, School of Medicine, Tsinghua University, Beijing, China; c Peking Union Medical College Hospital, Chinese Academy of Medical Sciences & Peking Union Medical College, Beijing, China

**Keywords:** Artificial intelligence, Large language model, Clinical reasoning, Medical licensure, Medical education

## Abstract

**Objective:**

The rapid advancement of Large Language Models (LLMs) has generated interest in their application to medical education, particularly for high-stakes assessments like the USMLE. This study aims to evaluate the performance of DeepSeek-R1, a state-of-the-art LLM developed in China, compared to OpenAI models, to assess its feasibility for medical education and assessment.

**Methods:**

The authors evaluated the performance of five models, including DeepSeek-R1, DeepSeek-V3, and three OpenAI models (GPT-4 Omni, OpenAI o3-mini, OpenAI o1 pro), on 321 text-based USMLE-style questions. Accuracy rates were calculated, and statistical comparisons were performed using Chi-Square tests with Bonferroni correction.

**Results:**

DeepSeek-R1 achieved the highest overall accuracy of 92.5% (95% CI 89.1%‒94.9%), significantly outperforming the OpenAI models (all 78.8%, p < 0.0001). DeepSeek-R1 also surpassed the reported average human examinee performance across all USMLE steps. The inter-model consensus between DeepSeek-R1 and OpenAI o1 pro yielded 94.9% accuracy, indicating high reliability for straightforward queries. Furthermore, in discordant cases, DeepSeek-R1 demonstrated superior capability with 82.8% accuracy compared to 14.1%‒28.1% for the OpenAI models (p < 0.0001).

**Conclusion:**

DeepSeek-R1 emerges as a compelling candidate in the AI-driven healthcare landscape, demonstrating superior accuracy and reasoning capabilities. However, its current limitation in multimodal data processing underscores the need for further innovation. These findings provide valuable insights for educators and policymakers regarding the integration of non-Western LLMs into medical assessment.

## Highlights

• DeepSeek-R1 (92.5%) outperforms OpenAI models and human examinees on USMLE.
• Shows superior reasoning in discordant questions (82.8%vs. 14.1%‒28.1%).
• First LLM to surpass human-level performance on all three USMLE steps.
• Positions DeepSeek-R1 as a key tool for medical education and clinical AI.

## Introduction

Artificial Intelligence (AI) refers to the development of systems capable of simulating human intelligence to perform tasks traditionally requiring human cognition. These tasks include a wide array of capabilities, including learning, reasoning, problem-solving, perception, and natural language understanding.^1^ Among the most notable advancements in AI are Large Language Models (LLMs), which are trained on extensive text datasets to recognize and replicate complex linguistic patterns. In the medical field, LLMs enable a variety of applications, such as answering medical queries, drafting documentation, translating languages, and generating creative content.^2^^,^^3^

LLMs have emerged as a transformative technology largely due to their unprecedented ability to process and analyse language. Their capacity to generate contextually relevant and coherent responses has impacted numerous fields, including medical diagnosis, literature analysis, and medical education.^4^^,^^5^ As these models evolve, their integration into diverse applications is reshaping institutional and individual interactions with technology.^6^

One of the most widely recognized LLMs is ChatGPT, developed by OpenAI. Built on the GPT (Generative Pre-trained Transformer) architecture, ChatGPT is trained on diverse internet-sourced datasets, enabling it to generate coherent responses across a broad range of topics.^7^ Since its launch in 2022, ChatGPT has garnered significant attention for its ability to engage in natural conversation, assist with creative writing, and support academic tasks.^7^^,^^8^

Research suggests that ChatGPT can outperform first- and second-year medical students in answering complex clinical reasoning questions.^9^ Notably, studies have demonstrated its ability to pass the United States Medical Licensing Examination (USMLE) without specialized medical training.^10^ The USMLE is a comprehensive series of exams designed to evaluate the knowledge and skills essential for medical licensure in the United States. Such standardized assessments play a critical role in ensuring fairness, consistency, and objectivity in evaluating the proficiency of medical students and professionals.

Despite its promise, ChatGPT faces limitations in medical education. Like many AI models, it may inadvertently produce biased content due to training data imbalances, potentially perpetuating stereotypes. This raises ethical concerns regarding the fairness and reliability in the medical context.^11^ Furthermore, the ‘black box’ nature of these models obscures their decision-making mechanisms.^5^^,^^12^^,^^13^ This lack of transparency complicates efforts to evaluate the reasoning behind its outputs, which is a critical issue for fields requiring high accountability.^12^ Additionally, accessibility and regulatory challenges restrict ChatGPT’s use in certain regions. For example, OpenAI’s services, including ChatGPT, are not officially available in China, forcing users to rely on unstable workarounds. Moreover, many Chinese institutions prohibit unapproved foreign software, limiting ChatGPT’s adoption in local medical education.^14^

To meet the ethical, linguistic, and regulatory requirements of the Chinese market, domestic LLMs have evolved rapidly. Leading technology companies have introduced models such as Baidu’s ERNIE Bot, Alibaba’s Tongyi Qianwen, Tencent’s Hunyuan, Huawei’s Pangu Model, iFlyTek’s SparkDesk, Tsinghua University’s ChatGLM, and DeepSeek. Among these, DeepSeek, developed by DeepSeek-AI, stands out. Leveraging advanced deep learning techniques and a Mixture of Experts (MoE) architecture, DeepSeek reduces computational demands while enhancing performance. As an open-source LLM, it allows for code modification and customization, fostering continuous improvement.

Since its launch in January 2025, DeepSeek has rapidly gained traction in China. However, its performance in standardized medical assessments like the USMLE remains largely uncharacterized. Unlike ChatGPT, DeepSeek’s capabilities in medical reasoning have not been systematically evaluated. To address this gap, this study benchmarks DeepSeek’s performance on the USMLE against ChatGPT. By systematically evaluating accuracy and response quality, this research provides educators and policymakers with valuable insights into the feasibility of integrating DeepSeek into medical education, particularly in regions where it may serve as a compliant alternative to Western models.

## Material and methods

### Study design

This study evaluates DeepSeek’s performance on the USMLE in comparison to ChatGPT through a systematic analysis of accuracy and response quality. As an *in silico* benchmarking study evaluating artificial intelligence models on a publicly available question bank, it does not involve human participants, animal subjects, or clinical data. Therefore, standard clinical reporting guidelines (e.g., CONSORT, STROBE, ARRIVE) are not strictly applicable. However, to maintain rigorous reporting standards, the authors have ensured full transparency regarding the data sources, LLM versions, and evaluation protocols throughout the methodology.

### Large language models

The authors assessed two primary LLMs across multiple versions: the GPT series (OpenAI, USA) and DeepSeek (DeepSeek, China).

#### OpenAI models

GPT-4 Omni (GPT-4o), released in May 2024, is a multimodal model capable of processing and generating text, images, and audio.^15^ OpenAI o3-mini, officially launched in January 2025, is a high-speed, cost-effective reasoning model fine-tuned for STEM applications. It demonstrates exceptional performance in science, mathematics, and coding, while supporting developer features such as function calling and structured outputs.^16^ OpenAI o1 pro, released December 2024, represents an advanced iteration designed for complex problem-solving; it leverages increased computational resources to enhance accuracy and generate detailed responses.^17^ All OpenAI models were accessed via the official website. Version identifiers: ChatGPT-4o (latest), o3-mini (v1.0), and o1 pro.

#### DeepSeek models

DeepSeek-V3 is a high-performance, open-source MoE language model comprising 671 billion total parameters, with 37 billion active parameters per token. Designed for coding, mathematics, and general reasoning, it was pretrained on a 14.8 trillion-token multilingual corpus, followed by supervised fine-tuning on approximately 1.5 million reasoning and conversational samples, and is deployed with a 128K-token context window.^18^ DeepSeek-R1 utilizes the same large-scale MoE architecture (671 billion parameters, ∼37 billion active) but incorporates a distinct multi-stage training pipeline. Building upon DeepSeek-V3-Base, R1 integrates supervised fine-tuning on curated ‘cold-start’ examples and reinforcement learning using both rule-based and model-based rewards. This process, augmented by data synthesis (∼800 K samples), is designed to significantly enhance reasoning capabilities and output clarity.^19^

This study exclusively utilized official, off-the-shelf versions of these models accessed via their respective web interfaces. No additional pre-training or task-specific fine-tuning was performed. All evaluations reflect the models’ default capabilities as provided by the developer.

### Data sources

The authors utilized an officially released USMLE question bank comprising sample questions from Step 1 (basic sciences), Step 2 CK (Clinical Knowledge), and Step 3 (patient management). This resource is publicly available on the official USMLE website: https://www.usmle.org/exam-resources and in the Supplementary Material S1. All sample questions are single ‘One-Best-Answer’ questions with 4 to 6 response options. To ensure methodological consistency with prior benchmarks and isolate textual reasoning capabilities (e.g., MedQA),^20^ the authors excluded questions requiring image interpretation. This resulted in a final dataset of 321 text-only questions.

### Performance evaluation

A zero-shot evaluation approach was employed, with all model testing conducted between February and March 2025. The authors employed a standardized prompt across all models: ‘Please provide the best option for the following question’. The temperature was set to 1.0 for the OpenAI models and 0.7 for the DeepSeek models, with a fixed context window of 4096 tokens.

Accuracy was defined as the percentage of correct responses matching the standard key. Accuracy consensus refers to questions for which all models provided the correct answer. For the complete question bank (n = 321), the detailed responses of all five models are provided in Supplementary Material S2.

### Statistical analysis

Model performance was compared using chi-square tests with Bonferroni correction. Confidence Intervals (95% CIs) were calculated using the Wilson score method. Analyses and visualisation were conducted using GraphPad Prism (version 10.4.0).

## Results

### The DeepSeek series demonstrated superior performance on USMLE-styled questions

The initial question bank consisted of 376 items, including a subset with interactive clinical images. To ensure equitable comparison across text-only LLMs (e.g., DeepSeek-V3, DeepSeek-R1, and OpenAI o3-mini), the authors restricted their analysis to text-based USMLE questions. An analysis of the excluded image-based questions (n = 55) confirmed no specialty bias, showing a balanced distribution across disciplines: Radiology (25.5%), Dermatology (21.8%), and Pathology (14.5%, Supplementary Material S3). Consequently, the final dataset for comparative evaluation comprised 321 text-based questions.

**Fig. 1:**
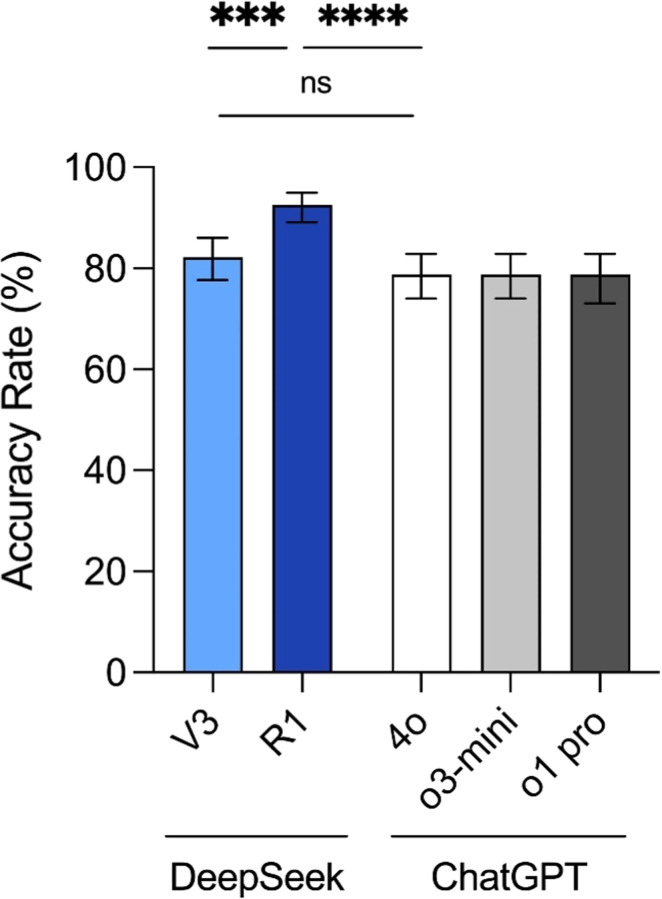
Overall comparative performance of LLMs on USMLE-style questions. Bar graph showing the accuracy rates of five LLMs including DeepSeek-R1, DeepSeek-V3, and three GPT models (GPT-4 Omni, OpenAI o3-mini, and OpenAI o1 pro), with 95% CI, evaluated on a dataset of 321 USMLE-style questions. Chi-square test with Bonferroni correction, ns, p > 0.05, ***p < 0.001, ****p < 0.0001.

Regarding the overall accuracy across the question bank, DeepSeek-R1 significantly outperformed all other evaluated models, achieving an accuracy rate of 92.5% (Fig. 1, 95% CI 89.1%‒94.9%). DeepSeek-V3 ranked second with an accuracy rate of 82.2% (95% CI 77.7%‒86.0%). However, this performance did not differ significantly from that of the GPT series models (Fig. 1, p > 0.05). Notably, all three GPT models (GPT-4 Omni, OpenAI o3-mini, and OpenAI o1 pro) achieved identical accuracy rates of 78.8% (95% CI 74.0%‒82.9%).

The USMLE is a three-step examination system aiming to assess a candidate’s readiness for medical practice. Each step targets distinct competencies: Step 1 focuses on the mastery of foundational basic science concepts essential to clinical medicine. Step 2 Clinical Knowledge (CK) evaluates the application of medical knowledge and clinical science in supervised patient care scenarios. Step 3 measures the ability to apply biomedical and clinical science knowledge in the context of unsupervised medical practice, with an emphasis on ambulatory management and advanced clinical decision-making. To investigate potential performance variations across these distinct assessment domains, the authors analyzed the accuracy rates of the five evaluated LLMs in each of the three USMLE steps.

**Fig. 2:**
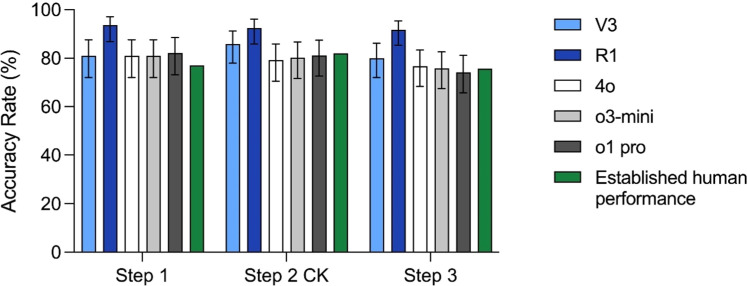
Comparative performance of LLMs across USMLE steps. Bar graph showing the accuracy rates of five LLMs on question banks divided by USMLE sections (Step 1, Step 2 CK, and Step 3). Error bars represent 95% CI. Benchmarks for average human examinee performance are derived from 2021 NBME data.

The present analysis demonstrates that DeepSeek-R1 consistently outperformed all other models across all three steps, maintaining superior accuracy rates (Table 1 and Fig. 2). Notably, while OpenAI o1-pro achieved the second-highest accuracy rate in Step 1, DeepSeek-V3 emerged as the second-best performer in both Step 2 CK and Step 3 (Table 1 and Fig. 2). Despite these rankings, all five models successfully surpassed the approximate passing threshold for each step (defined as an accuracy rate of 60%).

**Table 1:** Performance comparison of LLMs on USMLE-style questions. Accuracy rates were calculated based on the performance of each model on a filtered dataset of 321 USMLE-style questions with three sections (Step 1, Step 2 CK and Step 3). Chi-Square test with Bonferroni correction was conducted to compare the performance of each model against DeepSeek-R1 (reference model).

Model	**Step 1** **(n = 95)**	**Step 2 CK** **(n = 106)**	**Step 3** **(n = 120)**	**Total** **(n = 321)**	**Adjusted p-value**
DeepSeek-R1	89 (93.7%)	98 (92.5%)	110 (91.7%)	297 (92.5%)	Reference
DeepSeek-V3	77 (81.0%)	91 (85.8%)	96 (80.0%)	264 (82.2%)	<0.001
GPT-4 Omni	77 (81.0%)	84 (79.2%)	92 (76.7%)	253 (78.8%)	<0.0001
OpenAI o3-mini	77 (81.0%)	85 (80.2%)	91 (75.8%)	253 (78.8%)	<0.0001
OpenAI o1 pro	78 (82.1%)	86 (81.1%)	89 (74,2%)	253 (78.8%)	<0.0001

The authors further benchmarked the LLMs against the official average performance of examinees from LCME-accredited U.S. and Canadian medical schools in 2021.^21^ This cohort was selected as 2021 marked the final year in which Step 1 scores were reported numerically prior to the transition to a pass/fail system in 2022. Notably, DeepSeek-R1 exceeded historical human performance benchmarks across all steps (Step 1: +16.7 percentage points, Step 2 CK: +10.5 percentage points, Step 3: +16.0 percentage points).

### The performance of LLMs varies across different medical specialties

To analyze model capabilities across diverse medical domains, the authors stratified the dataset into 10 distinct specialties based on clinical content. These specialties included cardiology (n = 27), pulmonology (n = 23), endocrinology (n = 24), nephrology (n = 19), hematology (n = 19), gastroenterology (n = 19), neurology (n = 20), immunology (n = 8), infectious disease (n = 12), and psychiatry (n = 15).

**Fig. 3:**
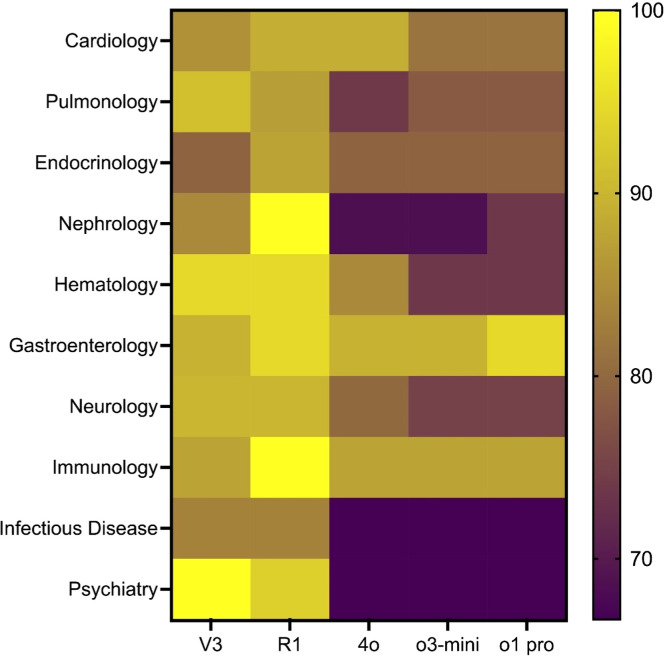
Heatmap of LLM accuracy stratified by medical specialty. The heatmap illustrating the accuracy rates of five LLMs across 10 medical specialties: cardiology, pulmonology, endocrinology, nephrology, haematology, gastroenterology, neurology, immunology, infectious disease, and psychiatry. The color scale on the right represents diagnostic accuracy ranging from 67% to 100%, with lighter yellow intensities corresponding to higher accuracy and darker purple indicating lower performance.

Consistent with the aggregate trends observed in Fig. 1, Fig. 2, the DeepSeek series (V3 and R1) demonstrated superior accuracy rates across all 10 medical specialties. However, performance gaps narrowed in specific domains. In certain specialties, such as cardiology, endocrinology, gastroenterology, and immunology, the GPT series achieved comparable accuracy rates to the DeepSeek models (Fig. 3). Conversely, significant disparities emerged in infectious disease and psychiatry, where the GPT series lagged behind, showing accuracy rates between 60% and 70%, whereas the DeepSeek series demonstrated robust performance in these areas (Fig. 3). Detailed responses of the five models across different specialties are provided in Supplementary Material S4.

### Analysis of consensus and discordant performance

**Fig. 4:**
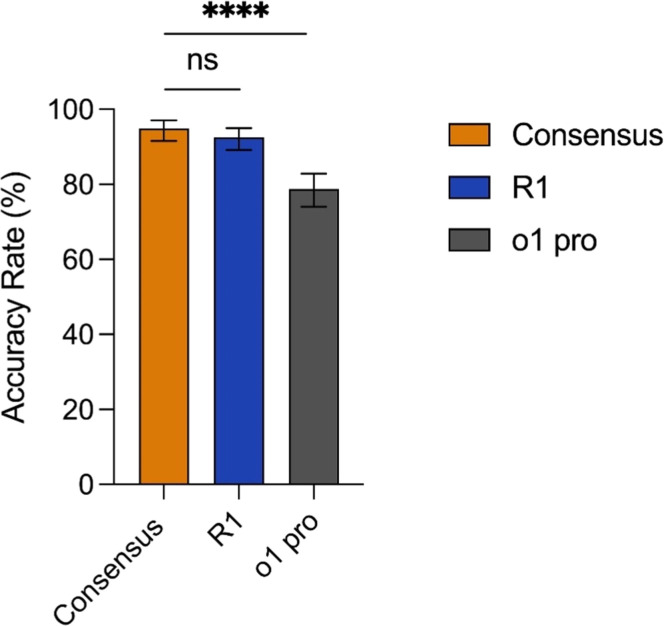
The performance of DeepSeek-R1 and OpenAI o1 pro on concordant questions. Bar graph showing the individual accuracy rates of DeepSeek-R1 and OpenAI o1 pro among the whole question bank (n = 321), and the accuracy rate achieved when both models provided concordant answers (n = 257), with 95% CI. Chi-Square test with Bonferroni correction, ns, p > 0.05, **** p < 0.0001.

In the present study, both DeepSeek-R1 and OpenAI o1 pro achieved superior accuracy rates compared to their respective model cohorts. Given their different training methodologies and architectures, the authors hypothesized that a combined approach might enhance performance on USMLE-style questions. To explore this, the authors analyzed the subset of questions where both models yielded concordant responses. Of the 321 questions evaluated, DeepSeek-R1 and OpenAI o1 pro agreed on 257 items, resulting in a concordance rate of 80.1% (Fig. 4). Notably, within this subset of concordant responses, the accuracy rate reached 94.9% (244/257, 95% CI 91.5%‒97.0%). However, the consensus accuracy did not significantly differ from DeepSeek-R1’s overall accuracy (p > 0.05). This suggests that the high accuracy of the consensus set was primarily driven by DeepSeek-R1’s robust baseline performance, rather than a synergistic effect. Furthermore, the superiority of the consensus subset over OpenAI o1 pro’s general performance indicates that discordance often stemmed from errors by OpenAI o1 pro rather than DeepSeek-R1 (Fig. 4).

To further evaluate performance distinctiveness, the authors analysed the discordant subset (n = 64), where the two models provided conflicting answers. In this subset, DeepSeek-R1 demonstrated statistically superior performance, achieving 82.8% accuracy (95% CI 71.8%‒90.1%), significantly outperforming all benchmark models (p < 0.01). This advantage was pronounced when contrasted with OpenAI models, which exhibited markedly lower accuracy rates ranging from 14.1% to 28.1% (Table 2). These findings collectively suggest that DeepSeek-R1 possesses enhanced clinical reasoning capabilities, particularly when navigating diagnostically ambiguous or complex medical scenarios.

**Table 2:** Performance comparison of LLMs on 64 discordant items. This table compares the accuracy of five LLMs (DeepSeek-R1, DeepSeek-V3, OpenAI-4o, OpenAI-o3 mini, and OpenAI-o1 pro) on 64 USMLE questions where DeepSeek-R1 and OpenAI-o1 pro initially disagreed. Chi-Square test with Bonferroni correction was conducted to compare the performance of each model against DeepSeek-R1 (reference model).

**Model**	**Correct answers**	**Accuracy (%)**	**95% CI**	**Adjusted p-value**
DeepSeek-R1	53/64	82.8	[71.8%, 90.1%]	Reference
DeepSeek-V3	35/64	54.7	[42.6%, 66.3%]	<0.01
GPT-4 Omni	18/64	28.1	[18.6%, 40.1%]	<0.0001
OpenAI o3-mini	13/64	20.3	[12.3%, 31.7%]	<0.0001
OpenAI o1 pro	9/64	14.1	[7.6%, 24.6%]	<0.0001

## Discussion

The advent of AI and the rapid evolution of LLMs have revolutionized medical education and assessment.^2^^,^^3^ Models like ChatGPT have sparked significant interest for their potential to streamline assessments and provide personalized learning.^4^^,^^5^ Recent literature has further expanded this landscape. For instance, scholars have explored the ChatGPT-empowered machine learning paradigm, highlighting its potential as an easy-to-use aide-memoire for medical professionals.^22^ Furthermore, comparative studies in specific domains, such as pancreatic cancer queries, have evaluated AI against surgeons, offering critical insights into the accuracy and empathy of AI-generated responses.^23^ In this context, this study explores the performance of DeepSeek-R1 and OpenAI o1 pro on USMLE-style questions to advance the understanding of AI-driven solutions in healthcare.

The USMLE is characterized by its rigorous emphasis on clinical problem-solving and strict alignment with real-world medical practice. Consequently, the performance of LLMs on this examination serves as a robust indicator of their potential utility in clinical settings. In this comprehensive evaluation, DeepSeek-R1 emerged as the top-performing model, achieving the highest overall accuracy (92.5%) and consistently outperforming the reported average of human examinees across all three USMLE steps (Fig. 1, Fig. 2). This suggests that DeepSeek-R1 possesses advanced capabilities in integrating medical knowledge, particularly in basic sciences (Step 1) and patient management (Step 3), where its advantage was most pronounced (Fig. 2).

In contrast, the GPT series models (GPT-4 Omni, OpenAI o3-mini, and OpenAI o1 pro) converged at a lower accuracy plateau (78.8%). Despite their architectural differences, the similar performance among OpenAI models suggests a potential ceiling in their training data regarding specific medical nuances compared to the optimization strategies employed by DeepSeek.

While the DeepSeek series excels across a broad spectrum of medical specialties, the GPT series demonstrates competitive performance in specific domains such as cardiology and endocrinology (Fig. 3). However, the observed disparities in specialties like infectious disease and psychiatry underscore the importance of domain-specific evaluation. These variations may reflect differences in training data distribution and architectural priorities between the models.

Specifically, DeepSeek-R1 excels in tasks requiring deep medical knowledge and clinical reasoning, attributed to its advanced architecture and high-quality fine-tuning. Conversely, OpenAI O1 pro leverages a diverse training corpus to offer adaptability in interdisciplinary scenarios.^20^ Investigating their performance on concordant versus discordant questions offers critical insights for clinical AI deployment.

First, the 94.9% accuracy in consensus questions suggests that agreement between top-tier LLMs could serve as a reliability marker for straightforward queries. This aligns with prior work demonstrating that ensemble approaches reduce variance in medical AI systems.^24^ The consensus accuracy mirrored DeepSeek-R1’s overall performance (p > 0.05), indicating that DeepSeek-R1’s strong baseline performance was a key factor in the agreement. Nevertheless, the consensus subset outperformed OpenAI o1 pro’s general performance, while disagreements primarily stemmed from OpenAI o1 pro’s errors. Crucially, the notable performance gap between consensus and discordant items implies that consensus may correlate with question simplicity, cautioning against overreliance on this metric for complex cases.

Second, DeepSeek-R1’s sustained accuracy on discordant questions (82.8%vs. 14.1%‒28.1% for OpenAI models) demonstrates its superior robustness in handling controversial or ambiguous clinical questions. While promising, this capability warrants further validation through real-world testing in multidisciplinary diagnosis teams.

The authors acknowledge several limitations in the present study. First, the exclusion of image-based questions restricts the evaluation to text-based reasoning, omitting the multimodal complexity of real clinical practice. Future research may prioritize multimodal integration to fully replicate clinical decision-making. Second, while the authors employed a standardized zero-shot prompting strategy to ensure fair comparison, advanced techniques like chain-of-thought prompting might yield different results. Prior research indicates that CoT prompting, which guides models through intermediate reasoning steps, can significantly enhance performance in complex reasoning tasks.^25^ Although the preliminary experiment with 30-questions revealed that CoT did not significantly alter accuracy compared to direct prompting ‒ DeepSeek-R1 and GPT-4o produced identical final answers (100%, 30/30), while DeepSeek-V3 showed a concordance rate of 93.3% (28/30, Supplementary Material S5) ‒ future studies should further explore how diverse prompting strategies affect LLM performance on the USMLE. Furthermore, this design choice, while enabling standardized comparison across models, prevented detailed analysis of whether errors resulted from factual inaccuracies, reasoning flaws, or other failure modes. To ensure transparency and facilitate independent analysis, all incorrect responses are provided in Supplementary Material S2. Finally, specific to DeepSeek-R1, while it demonstrates strong performance on the USMLE, a potential imbalance in its training data, which possibly favours Chinese over non-Chinese sources, may impact its performance on cases that occur more frequently in specific regions. However, the proportion of Chinese-centric training data has not been officially disclosed.

In addition to these methodological constraints, the scope of this evaluation presents another limitation. While this evaluation demonstrates strong performance on USMLE content, the authors recognize that this exam primarily assesses knowledge of common conditions. This focus may not adequately reflect real-world diagnostic challenges where rare diseases collectively account for a notable proportion of clinical cases. Future studies should specifically evaluate LLM performance across the full spectrum of disease prevalence.

Extending beyond technical metrics and scope, the integration of LLMs into high-stakes medical assessments raises critical ethical challenges that must be proactively addressed. First, algorithmic bias remains a persistent concern, as models may underperform on questions involving underrepresented populations (e.g., racial/ethnic minorities) due to skewed training data distributions.^26^ Second, the lack of explainability in LLM decision-making complicates accountability ‒ a non-negotiable requirement in clinical settings where erroneous reasoning could have life-altering consequences.^27^ Third, overreliance risk emerges when users misinterpret statistically plausible outputs as ground truth, a phenomenon exacerbated by the models’ tendency to generate confident but incorrect responses.^28^

Notwithstanding these limitations and ethical concerns, the rapid technological evolution of DeepSeek-R1 offers a promising outlook. Since its rise to prominence in January 2025, DeepSeek-R1 has undergone substantial enhancements. The May 2025 update notably improved reasoning capabilities, reduced hallucinations, and provided smoother front-end outputs,^29^ leading to more stable operation.^30^ These improvements have rendered DeepSeek more reliable and versatile for real-world applications. Consequently, multiple Chinese healthcare institutions have successfully integrated DeepSeek-R1 into real-world workflows for automated documentation.^31^ For instance, Tsinghua Chang Gung Hospital has employed the model for automated medical note generation, reporting service interruptions of <1%.^32^ These advancements position DeepSeek-R1 as a promising tool for resource-limited settings by bringing specialist-level insights to primary care, enhancing diagnostic consistency in academic medical centres, and reducing documentation burden across healthcare systems.

## Conclusions

This study represents a comprehensive evaluation of DeepSeek’s performance in medical assessment and practice, offering novel insights into its capabilities relative to established models like ChatGPT. The present results demonstrate that DeepSeek-R1 achieved the highest accuracy among the evaluated models, surpassing the performance benchmark of the average human examinee. However, persistent limitations ‒ specifically the exclusion of multimodal data processing and technical hurdles in real-world deployment ‒ highlight the need for continued innovation in hybrid AI systems and infrastructure optimization. Future research should prioritize multimodal integration, domain-specific fine-tuning, and scalable architectures to effectively bridge the gap between AI capabilities and clinical demands. Addressing these challenges will empower the next generation of LLMs to further benefit medical education, enhance diagnostic accuracy, and ultimately improve patient care.

## Ethics approval and consent to participate

Not applicable.

## Consent for publication

Not applicable.

## Data availability

The datasets used and/or analysed during the current study are available from the corresponding author on reasonable request.

## Funding

This work is supported by 10.13039/501100001809National Natural Science Foundation of China (n°62476287); Beijing Municipal Science and Technology Plan (Special Project: Cultivation of Innovative Pharmaceutical Varieties and Platforms), Beijing Municipal Science and Technology Commission & Zhongguancun Science Park Administration Committee (n°Z251100004625026); and National High-Level Hospital Clinical Research Funding (n°2025-PUMCH-A-028).

## CRediT authorship contribution statement

**Yuchen Zhou:** Conceptualization, Methodology, Investigation, Formal analysis, Data curation, Writing – original draft. **Weiping Wang:** Methodology, Validation, Investigation, Formal analysis, Writing – original draft. **Xianhe Zhao:** Data curation, Validation, Visualization, Writing – review & editing. **Ke Hu:** Conceptualization, Resources, Supervision, Project administration, Funding acquisition, Writing – review & editing.

## Declaration of competing interest

The authors declare no conflicts of interest.

## Appendix Supplementary materials

Image, application 1

Image, application 2

Image, application 3

Image, application 4

Image, application 5

Image, application 6

## Footnotes

Supplementary material associated with this article can be found, in the online version, at doi:10.1016/j.clinsp.2026.101021.
