# Supplementary material for: A comparative benchmark of DeepSeek-R1 on the USMLE: surpassing human and AI performance averages
Source: Clinics (Sao Paulo). 2026 Jun 15;81:101021. doi: 10.1016/j.clinsp.2026.101021 (PMC13285263; doi:10.1016/j.clinsp.2026.101021)
Supplement: Supplementary file 1 [file mmc1.docx]

**CLINICS-D-25-00295**

**Supplementary Material**

**Supplementary Material S1**

**Supplementary Material S2**

**Supplementary Material S3**

**Supplementary Material S4**

**Supplementary Material S5**
