# Supplementary material for: A comparative benchmark of DeepSeek-R1 on the USMLE: surpassing human and AI performance averages
Source: Clinics (Sao Paulo). 2026 Jun 15;81:101021. doi: 10.1016/j.clinsp.2026.101021 (PMC13285263; doi:10.1016/j.clinsp.2026.101021)
Supplement: Supplementary file 2 [file mmc2.pdf]

USMLE[SM]

Sample Test Questions

Step 1

A Joint Program of the Federation of State Medical Boards of the United States, Inc. (FSMB), and National Board of Medical Examiners® (NBME®)

This booklet was updated January 2024.
For Public Release

Copyright © 2024 by the Federation of State Medical Boards of the United States, Inc. (FSMB), and National Board of Medical Examiners (NBME). All rights reserved. Printed in the United States of America. The United States Medical Licensing Examination (USMLE®) is a joint program of the FSMB and NBME.

CONTENTS

USMLE Step 1 Test Question Formats 3

Introduction to USMLE Step 1 Sample Test Questions 4

USMLE Laboratory Values 5

USMLE Step 1 Sample Test Questions.. 8

Answer Form for USMLE Step 1 Sample Test Questions... 52

Answer Key for USMLE Step 1 Sample Test Questions.. 53

USMLE STEP 1 TEST QUESTION FORMATS

Single-Item Questions

A single patient-centered vignette is associated with one question followed by four or more response options. The response options are lettered (A, B, C, D, E). A portion of the questions require interpretation of graphic or pictorial materials. You are required to select the best answer to the question. Other options may be partially correct, but there is only ONE BEST answer. This is the traditional, most frequently used multiple-choice question format on the examination.

Strategies for Answering Single One-Best-Answer Test Questions

The following are strategies for answering one-best-answer items:

- Read each patient vignette and question carefully. It is important to understand what is being asked.
- Try to generate an answer and then look for it in the response option list.
- Alternatively, read each response option carefully, eliminating those that are clearly incorrect.
- Of the remaining options, select the one that is most correct.
- If unsure about an answer, it is better to guess since unanswered questions are automatically counted as wrong answers.

Example Item

A 32-year-old woman with type 1 diabetes mellitus has had progressive renal failure during the past 2 years. She has not yet started dialysis. Examination shows no abnormalities. Her hemoglobin concentration is 9 g/dL, hematocrit is 28%, and mean corpuscular volume is 94 μm^3. A blood smear shows normochromic, normocytic cells. Which of the following is the most likely cause?

(A) Acute blood loss
(B) Chronic lymphocytic leukemia
(C) Erythrocyte enzyme deficiency
(D) Erythropoietin deficiency
(E) Immunohemolysis
(F) Microangiopathic hemolysis
(G) Polycythemia vera
(H) Sickle cell disease
(I) Sideroblastic anemia
(J) β-Thalassemia trait

(Answer: D)

NOTE: Some item types that appear on the Step 1 examination are NOT depicted in the sample items provided in this booklet, eg, items with multimedia features, such as audio. Also, when additional item formats are added to the exam, notice will be provided at the USMLE website: http://www.usmle.org. You must monitor the website to stay informed about the types of items that occur in the exam, and you must practice with the downloadable sample test items available on the USMLE website to be fully prepared for the examination.

INTRODUCTION TO USMLE STEP 1 SAMPLE TEST QUESTIONS

The following pages include 119 sample test questions. Most of these questions are the same as those you can install on your computer from the USMLE website. Please note that reviewing the sample questions as they appear on pages 8–51 is not a substitute for practicing with the test software. You should download and run the Step 1 tutorial and practice test items that are provided on the USMLE website well before your test date. The sample materials available on the USMLE website include an additional item with associated audio findings that does not appear in this booklet. You should become familiar with this item format that will be used in the actual examination.

Although the sample questions exemplify content on the Step 1 examination overall, they may not reflect the content coverage on individual examinations. In the actual examination, questions will be presented in random order; they will not be grouped according to specific content. The questions will be presented one at a time in a format designed for easy on-screen reading, including use of the USMLE Laboratory Values table (included here on pages 5–7) and some pictorials. Photographs, charts, and x-rays in this booklet are not of the same quality as the pictorials used in the actual examination. In addition, you will be able to adjust the brightness and contrast of pictorials on the computer screen.

To take the following sample test questions as they would be timed in the actual examination, you should allow a maximum of 1 hour for each 40-item block, and a maximum of 58 minutes, 30 seconds, for the 39-item block, for a total of 2 hours, 58 minutes, 30 seconds. Please note that the third block has 39 items instead of 40 because the multimedia item has been removed, and the recommended time to complete the block has been adjusted accordingly. Please be aware that most examinees perceive the time pressure to be greater during an actual examination. All examinees are strongly encouraged to practice with the downloadable version to become familiar with all item formats and exam timing. An answer form for recording answers is provided on page 52. An answer key is provided on page 53. In the actual examination, answers will be selected on the screen; no answer form will be provided.

	Reference Range	SI Reference Intervals
**SERUM**		
General Chemistry:		
Electrolytes		
Sodium (Na^+)	136–146 mEq/L	136–146 mmol/L
Potassium (K^+)	3.5–5.0 mEq/L	3.5–5.0 mmol/L
Chloride (Cl^-)	95–105 mEq/L	95–105 mmol/L
Bicarbonate (HCO_3^-)	22–28 mEq/L	22–28 mmol/L
Urea nitrogen	7–18 mg/dL	2.5–6.4 mmol/L
Creatinine	0.6–1.2 mg/dL	53–106 µmol/L
Glucose	Fasting: 70–100 mg/dL	3.8–5.6 mmol/L
	Random, non-fasting: <140 mg/dL	<7.77 mmol/L
Calcium	8.4–10.2 mg/dL	2.1–2.6 mmol/L
Magnesium (Mg^{2+})	1.5–2.0 mg/dL	0.75–1.0 mmol/L
Phosphorus (inorganic)	3.0–4.5 mg/dL	1.0–1.5 mmol/L
Hepatic:		
Alanine aminotransferase (ALT)	10–40 U/L	10–40 U/L
Aspartate aminotransferase (AST)	12–38 U/L	12–38 U/L
Alkaline phosphatase	25–100 U/L	25–100 U/L
Bilirubin, total // direct	0.1–1.0 mg/dL // 0.0–0.3 mg/dL	2–17 µmol/L // 0–5 µmol/L
Proteins, total	6.0–7.8 g/dL	60–78 g/L
Albumin	3.5–5.5 g/dL	35–55 g/L
Globulin	2.3–3.5 g/dL	23–35 g/L
Other, serum:		
Amylase	25–125 U/L	25–125 U/L
Lipase	13–60 U/L	13–60 U/L
Creatinine clearance	Male: 97–137 mL/min	97–137 mL/min
	Female: 88–128 mL/min	88–128 mL/min
Creatine kinase	Male: 25–90 U/L	25–90 U/L
	Female: 10–70 U/L	10–70 U/L
Lactate dehydrogenase	45–200 U/L	45–200 U/L
Osmolality	275–295 mOsmol/kg H_2O	275–295 mOsmol/kg H_2O
Troponin I	≤0.04 ng/mL	≤0.04 µg/L
Uric acid	3.0–8.2 mg/dL	0.18–0.48 mmol/L
Lipids:		
Cholesterol		
Total	Normal: <200 mg/dL	<5.2 mmol/L
	High: >240 mg/dL	>6.2 mmol/L
HDL	40–60 mg/dL	1.0–1.6 mmol/L
LDL	<160 mg/dL	<4.2 mmol/L
Triglycerides	Normal: <150 mg/dL	<1.70 mmol/L
	Borderline: 151–199 mg/dL	1.71–2.25 mmol/L
Iron Studies:		
Ferritin	Male: 20–250 ng/mL	20–250 µg/L
	Female: 10–120 ng/mL	10–120 µg/L
Iron	Male: 65–175 µg/dL	11.6–31.3 µmol/L
	Female: 50–170 µg/dL	9.0–30.4 µmol/L
Total iron-binding capacity	250–400 µg/dL	44.8–71.6 µmol/L
Transferrin	200–360 mg/dL	2.0–3.6 g/L

Continued on Next Page

	Reference Range	SI Reference Intervals
Endocrine:		
Follicle-stimulating hormone	Male: 4–25 mIU/mL	4–25 IU/L
	Female: premenopause 4–30 mIU/mL	4–30 IU/L
	midcycle peak 10–90 mIU/mL	10–90 IU/L
	postmenopause 40–250 mIU/mL	40–250 IU/L
Luteinizing hormone	Male: 6–23 mIU/mL	6–23 IU/L
	Female: follicular phase 5–30 mIU/mL	5–30 IU/L
	midcycle 75–150 mIU/mL	75–150 IU/L
	postmenopause 30–200 mIU/mL	30–200 IU/L
Growth hormone - arginine stimulation	Fasting: <5 ng/mL	<5 µg/L
	Provocative stimuli: >7 ng/mL	>7 µg/L
Prolactin (hPRL)	Male: <17 ng/mL	<17 µg/L
	Female: <25 ng/mL	<25 µg/L
Cortisol	0800 h: 5–23 µg/dL	138–635 nmol/L
	1600 h: 3–15 µg/dL	82–413 nmol/L
	2000 h: <50% of 0800 h	Fraction of 0800 h: <0.50
TSH	0.4–4.0 µU/mL	0.4–4.0 mIU/L
Triiodothyronine (T_3) (RIA)	100–200 ng/dL	1.5–3.1 nmol/L
Triiodothyronine (T_3) resin uptake	25%–35%	0.25–0.35
Thyroxine (T_4)	5–12 µg/dL	64–155 nmol/L
Free T_4	0.9–1.7 ng/dL	12.0–21.9 pmol/L
Thyroidal iodine (^{123}I) uptake	8%–30% of administered dose/24 h	0.08–0.30/24 h
Intact PTH	10–60 pg/mL	10–60 ng/L
17-Hydroxycorticosteroids	Male: 3.0–10.0 mg/24 h	8.2–27.6 µmol/24 h
	Female: 2.0–8.0 mg/24 h	5.5–22.0 µmol/24 h
17-Ketosteroids, total	Male: 8–20 mg/24 h	28–70 µmol/24 h
	Female: 6–15 mg/24 h	21–52 µmol/24 h
Immunoglobulins:		
IgA	76–390 mg/dL	0.76–3.90 g/L
IgE	0–380 IU/mL	0–380 kIU/L
IgG	650–1500 mg/dL	6.5–15.0 g/L
IgM	50–300 mg/dL	0.5–3.0 g/L
**GASES, ARTERIAL BLOOD (ROOM AIR)**		
P_{O_2}	75–105 mm Hg	10.0–14.0 kPa
P_{CO_2}	33–45 mm Hg	4.4–5.9 kPa
pH	7.35–7.45	$[H^+]$ 36–44 nmol/L
**CEREBROSPINAL FLUID**		
Cell count	0–5/mm^3	0–5 × 10^6/L
Chloride	118–132 mEq/L	118–132 mmol/L
Gamma globulin	3%–12% total proteins	0.03–0.12
Glucose	40–70 mg/dL	2.2–3.9 mmol/L
Pressure	70–180 mm H_2O	70–180 mm H_2O
Proteins, total	<40 mg/dL	<0.40 g/L

Continued on Next Page

	Reference Range	SI Reference Intervals
**HEMATOLOGIC**		
Complete Blood Count:		
Hematocrit	Male: 41%–53%	0.41–0.53
	Female: 36%–46%	0.36–0.46
Hemoglobin, blood	Male: 13.5–17.5 g/dL	135–175 g/L
	Female: 12.0–16.0 g/dL	120–160 g/L
Mean corpuscular hemoglobin (MCH)	25–35 pg/cell	0.39–0.54 fmol/cell
Mean corpuscular hemoglobin conc. (MCHC)	31%–36% Hb/cell	4.8–5.6 mmol Hb/L
Mean corpuscular volume (MCV)	80–100 μm^3	80–100 fL
Volume		
Plasma	Male: 25–43 mL/kg	0.025–0.043 L/kg
	Female: 28–45 mL/kg	0.028–0.045 L/kg
Red cell	Male: 20–36 mL/kg	0.020–0.036 L/kg
	Female: 19–31 mL/kg	0.019–0.031 L/kg
Leukocyte count (WBC)	4500–11,000/mm^3	4.5–11.0 × 10^9/L
Neutrophils, segmented	54%–62%	0.54–0.62
Neutrophils, bands	3%–5%	0.03–0.05
Lymphocytes	25%–33%	0.25–0.33
Monocytes	3%–7%	0.03–0.07
Eosinophils	1%–3%	0.01–0.03
Basophils	0%–0.75%	0.00–0.0075
Platelet count	150,000–400,000/mm^3	150–400 × 10^9/L
Coagulation:		
Partial thromboplastin time (PTT/aPTT) (activated)	25–40 seconds	25–40 seconds
Prothrombin time (PT)	11–15 seconds	11–15 seconds
D-Dimer	≤250 ng/mL	≤1.4 nmol/L
Other, Hematologic:		
Reticulocyte count	0.5%–1.5%	0.005–0.015
Erythrocyte count (RBC)	Male: 4.3–5.9 million/mm^3	4.3–5.9 × 10^{12}/L
	Female: 3.5–5.5 million/mm^3	3.5–5.5 × 10^{12}/L
Erythrocyte sedimentation rate (Westergren)	Male: 0–15 mm/h	0–15 mm/h
	Female: 0–20 mm/h	0–20 mm/h
CD4+ T-lymphocyte count	≥500/mm^3	≥0.5 × 10^9/L
Endocrine:		
Hemoglobin A_{1c}	≤6%	≤42 mmol/mol
**URINE**		
Calcium	100–300 mg/24 h	2.5–7.5 mmol/24 h
Osmolality	50–1200 mOsmol/kg H_2O	50–1200 mOsmol/kg H_2O
Oxalate	8–40 µg/mL	90–445 µmol/L
Proteins, total	<150 mg/24 h	<0.15 g/24 h
**BODY MASS INDEX (BMI)**	Adult: 19–25 kg/m^2	

USMLE STEP 1 SAMPLE TEST QUESTIONS

BLOCK 1, ITEMS 1-40

1. A 14-year-old boy is brought to the emergency department by his parents because of a 1-month history of intermittent right knee pain that has worsened during the past day. He rates his current pain as a 6 on a 10-point scale and says that it worsens when he walks and lessens when he sits. During the past 2 weeks, he has been walking 1 mile daily in preparation for participation in the school marching band. He has not taken any medications for his pain. He sustained a right tibia and fibula fracture at the age of 8 years after a skateboarding accident, which was treated with internal fixation and casting. He has asthma treated with inhaled budesonide daily and inhaled albuterol as needed. His mother has type 2 diabetes mellitus, and his maternal grandmother has osteoporosis. The patient is 170 cm (5 ft 7 in; 77th percentile) tall and weighs 88 kg (195 lb; >95th percentile); BMI is 31 kg/m^2 (98th percentile). Temperature is 37.0°C (98.6°F), pulse is 95/min, and blood pressure is 130/80 mm Hg. Physical examination shows hyperpigmented, thickened skin at the nape of the neck. There is tenderness to palpation of the anterior aspect of the right hip and limited range of motion on abduction, internal rotation, and flexion of the right hip. The left hip and knees are nontender; range of motion is full in all directions. The remainder of the examination discloses no abnormalities. Which of the following factors in this patient's history most increased his risk for developing this condition?

(A) BMI
(B) Family history
(C) Medication use
(D) Previous fractures
(E) Recent physical activity

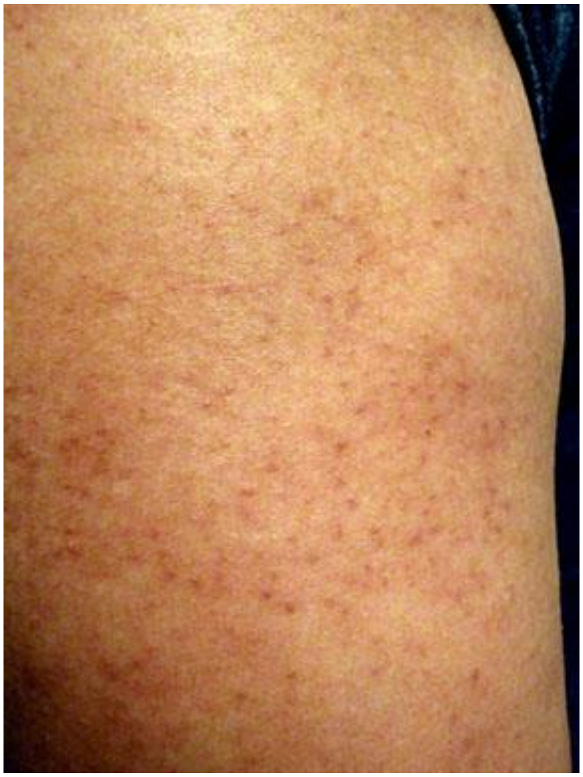

2. A 14-year-old girl is brought to the office by her mother because of a 3-month history of red bumps on her skin. The patient says the bumps are not itchy or painful but that she finds them embarrassing. She has no history of major medical illness and takes no medications. Her vital signs are within normal limits. Physical examination shows the findings in the photograph. Which of the following is the most likely diagnosis?

(A) Eczema
(B) Folliculitis
(C) Hidradenitis
(D) Keratosis pilaris
(E) Urticaria

---

3. A 50-year-old man comes to the office because of a 2-month history of increasing daytime somnolence. He has obstructive sleep apnea for which he has only intermittently used a continuous positive airway pressure device. He is 170 cm (5 ft 7 in) tall and weighs 181 kg (400 lb); BMI is 63 kg/m^2. His temperature is 37°C (98.6°F), pulse is 100/min, respirations are 12/min, and blood pressure is 135/80 mm Hg. Physical examination shows a gray-blue tinge to the lips, earlobes, and nail beds. Cardiac examination shows no other abnormalities. Arterial blood gas analysis on room air shows a pH of 7.31, P_{CO_2} of 70 mm Hg, and P_{O_2} of 50 mm Hg. Which of the following additional findings would be most likely in this patient?

(A) Decreased serum bicarbonate concentration
(B) Increased hemoglobin concentration
(C) Increased total lung capacity
(D) Left ventricular hypertrophy

4. A 32-year-old man comes to the office because of a 1-day history of cough productive of small amounts of blood and a 2-day history of shortness of breath and swelling of his ankles. He also has a 2-week history of progressive fatigue and episodes of dark urine. He has no history of major medical illness and takes no medications. His temperature is 37°C (98.6°F), pulse is 90/min, respirations are 18/min, and blood pressure is 175/110 mm Hg. Pulse oximetry on room air shows an oxygen saturation of 91%. Diffuse inspiratory crackles are heard over all lung bases. There is 2+ pitting edema of both ankles. Results of laboratory studies are shown:

Hemoglobin	8.9 g/dL
Hematocrit	27%
Serum	
Urea nitrogen	55 mg/dL
Creatinine	2.9 mg/dL
Urine RBC	20–40/hpf

Urinalysis also shows some dysmorphic RBCs and rare RBC casts. Examination of a kidney biopsy specimen shows crescentic glomerulonephritis and linear deposition of IgG along the glomerular capillaries. This patient most likely has antibodies directed against which of the following antigens?

(A) Collagen
(B) Double-stranded DNA
(C) Nucleolar protein
(D) Phospholipid
(E) Proteins in neutrophil cytoplasm

---

5. A 5-year-old girl is brought to the emergency department because of a 2-day history of fever, urinary urgency, and burning pain with urination. She has had four similar episodes during the past year. A diagnosis of urinary tract infection is made. Subsequent renal ultrasonography shows one large U-shaped kidney. Which of the following is the most likely embryologic origin of this patient's condition?

(A) Failure of the kidneys to rotate 90 degrees medially
(B) Failure of normal kidney ascent
(C) Failure of one ureteric bud to develop normally
(D) Fusion of the inferior poles of the metanephros during ascent

6. A 78-year-old man comes to the office for a follow-up examination. He was discharged from the hospital 1 week ago after being treated for a nontuberculous mycobacterial infection. He started treatment with ciprofloxacin and rifampin at that time. He also has hypertension and underwent placement of a mechanical aortic valve 6 years ago for aortic stenosis. Other current medications are hydrochlorothiazide, lisinopril, and warfarin. His warfarin dose was doubled 4 days ago. He says that he is trying to follow a healthier diet. He drinks two 12-oz beers daily. Results of laboratory studies done 4 days ago and today are shown

	**4 Days Ago**	**Today**
Prothrombin time	11 sec (INR=1)	11.2 sec (INR=1.1)
Partial thromboplastin time	29 sec	27 sec

Which of the following is the most likely cause of this patient's laboratory findings?

(A) Decreased protein binding
(B) Eradication of gut flora
(C) Increased alcohol intake
(D) Increased vegetable consumption
(E) Induction of cytochrome enzymes

---

7. A 32-year-old man comes to the office because of a 2-week history of fever and throat pain. He is 173 cm (5 ft 8 in) tall and weighs 63 kg (140 lb); BMI is 21 kg/m^2. His pulse is 110/min, respirations are 16/min, and blood pressure is 98/68 mm Hg. Physical examination shows scattered 2- to 4-cm lymph nodes in the neck, axillae, and inguinal regions. There is a bilateral tonsillar exudate but no ulcerations. Results of laboratory studies are shown:

Hemoglobin	9.6 g/dL
Hematocrit	29%
Leukocyte count	1500/mm^3
Platelet count	60,000/mm^3

A heterophile antibody test result is negative. Which of the following is the most likely diagnosis?

(A) Epstein-Barr virus infection
(B) Gonococcal pharyngitis
(C) HIV infection
(D) Lymphogranuloma venereum infection
(E) Streptococcal pharyngitis

---

8. A 50-year-old man comes to the office for a follow-up examination. He has a 2-month history of headache and shortness of breath with exertion. He also has hypertension treated with hydrochlorothiazide for the past 2 years. His blood pressure is 180/105 mm Hg. Ophthalmoscopic examination is most likely to show which of the following in this patient?

(A) Arteriovenous nicking
(B) Melanocytes in the uvea
(C) Optic neuritis
(D) Posterior subcapsular cataracts
(E) Tractional retinal detachment

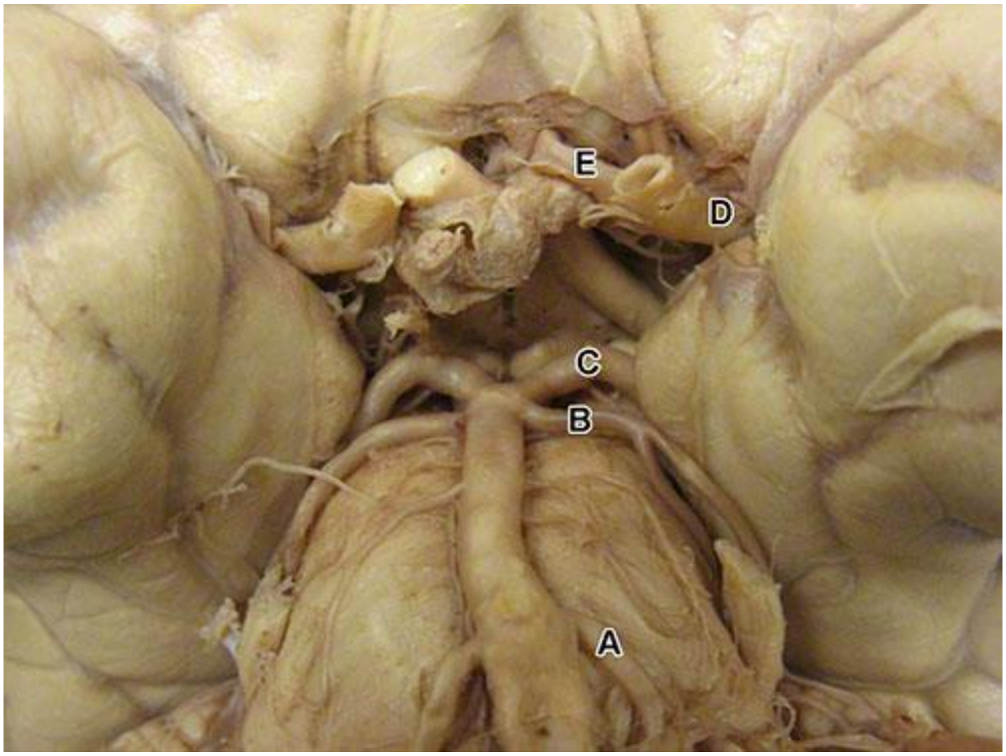

9. A 65-year-old woman with a history of rheumatic mitral valve disease is brought to the emergency department 30 minutes after the sudden onset of right-sided weakness and inability to speak. Neurologic examination shows weakness of the right lower side of the face and difficulty swallowing. Muscle strength is 3/5 on the right side. She can understand what is said to her, but she cannot repeat words or answer questions. An ECG shows atrial fibrillation. The most likely cause of the neurologic findings in this patient is occlusion of which of the following labeled arteries in the photograph of a normal brain?

(A) A
(B) B
(C) C
(D) D
(E) E

---

10. A 51-year-old man with a 10-year history of gastroesophageal reflux and suspected Barrett esophagus comes to the office because his omeprazole dose "doesn't work around the Christmas holidays." He states that he prides himself on having a large appetite and "holding his liquor" during the holidays. He currently takes the maximum dose of omeprazole. Which of the following is the most appropriate initial action by the physician?

(A) Ask the patient how much he is eating and drinking during the holidays
(B) Explain the hazards of untreated reflux in the presence of Barrett esophagus
(C) Order an upper endoscopy
(D) Refer the patient to a gastroenterologist
(E) Switch the omeprazole to pantoprazole

11. A 60-year-old man comes to the office because of weakness, tingling of his hands and feet, irritability, and forgetfulness for 4 months. Physical examination shows pallor, weakness, and spasticity. Deep tendon reflexes are increased. Sensation to vibration is absent in the lower extremities. Laboratory studies show megaloblastic anemia, serum antiparietal cell antibodies, and increased serum concentrations of methylmalonic acid and total homocyst(e)ine. The synthesis of which of the following amino acids is most likely impaired in this patient?

(A) Cysteine
(B) Glutamine
(C) Methionine
(D) Phenylalanine
(E) Tyrosine

12. A 65-year-old woman comes to the office for a follow-up examination 1 year after she underwent operative resection of the right colon and chemotherapy for stage III colon cancer. She reports fatigue. Physical examination shows no abnormalities. A staging CT scan of the chest and abdomen shows five new 2- to 3-cm masses in the liver and both lungs. This patient's cancer most likely spread to the lungs via which of the following structures?

(A) Inferior mesenteric vein
(B) Inferior vena cava
(C) Left colic vein
(D) Middle colic artery
(E) Pulmonary vein
(F) Superior mesenteric artery
(G) Superior vena cava

13. A 26-year-old man comes to the office because of a 1-week history of increased urinary frequency accompanied by excessive thirst. He says he has been urinating hourly. Physical examination shows no abnormalities. Serum chemistry studies are within the reference ranges. Urine osmolality is 50 mOsmol/kg H_2O. After administration of ADH (vasopressin), his urine osmolality is within the reference range. The most likely cause of this patient's symptoms is dysfunction of which of the following structures?

(A) Anterior pituitary gland
(B) Bowman capsule
(C) Glomerulus
(D) Hypophysial portal system
(E) Loop of Henle
(F) Supraoptic nucleus

14. A 52-year-old woman comes to the office because of a 6-month history of intermittent headaches. Sometimes the pain improves when the patient lies down in a quiet room. Her temperature is 37.5°C (99.5°F), pulse is 86/min, respirations are 16/min, and blood pressure is 154/100 mm Hg. The lungs are clear. Cardiac examination shows the point of maximal impulse displaced to the left and occasional skipped beats; there are no murmurs or rubs. There is no S_3. Resting electrocardiography shows left axis deviation with R waves greater than 30 mm in leads V_5 through V_6. Which of the following processes best explains the development of the left ventricular abnormalities in this patient?

(A) Excessive accumulation of glycogen
(B) Fibrosis of intraventricular conduction pathways
(C) Increased synthesis of contractile filaments
(D) Misfolding and aggregation of cytoskeletal proteins
(E) Myocyte hyperplasia as a result of induction of embryonic genes

15. A 53-year-old man comes to the physician because of a dry scaly rash on his body for the past year. He has had a 15-kg (33-lb) weight loss during the past year. He is 178 cm (5 ft 10 in) tall and now weighs 54 kg (120 lb); BMI is 17 kg/m^2. His stools have a large volume and float. Which of the following nutrient deficiencies is most likely?

(A) Magnesium
(B) Vitamin A
(C) Vitamin B_{12} (cobalamin)
(D) Vitamin C
(E) Zinc

16. Serum LDL-cholesterol concentrations are measured in blood samples collected from 25 healthy volunteers. The data follow a normal distribution. The mean and standard deviation for this group are 130 mg/dL and 25 mg/dL, respectively. The standard error of the mean is 5.0. With a 95% confidence level, the true mean for the population from which this sample was drawn falls within which of the following ranges (in mg/dL)?

(A) 105-155
(B) 120-140
(C) 125-135
(D) 128-132
(E) 129-131

17. A 39-year-old man comes to the physician because of a 6-month history of progressive shortness of breath. He has had a cough productive of white sputum for 2 years. He smoked 1 pack of cigarettes daily for 16 years but quit 10 years ago. He is in mild respiratory distress with pursed lips and a barrel chest; he is using the accessory muscles of respiration. Breath sounds are distant and crackles are present in the lower lung fields bilaterally. Pulmonary function tests show a decreased FEV_1:FVC ratio, increased residual volume, and decreased diffusion capacity. An x-ray of the chest shows hyperinflation and hypertranslucency of the lower lobes of both lungs. Which of the following is the most likely diagnosis?

(A) Asthma
(B) Bronchiectasis
(C) Chronic pulmonary fibrosis
(D) Cystic fibrosis
(E) Emphysema

18. Investigators conduct a study that evaluates the effect of finasteride on the incidence of prostate cancer in 500 patients. The investigators recruit an additional 1000 patients for the study. Which of the following effects will this have on the research study?

(A) Greater chance of a Type I error
(B) Greater chance of a Type II error
(C) Less chance of a Type I error
(D) Less chance of a Type II error
(E) Impossible to predict

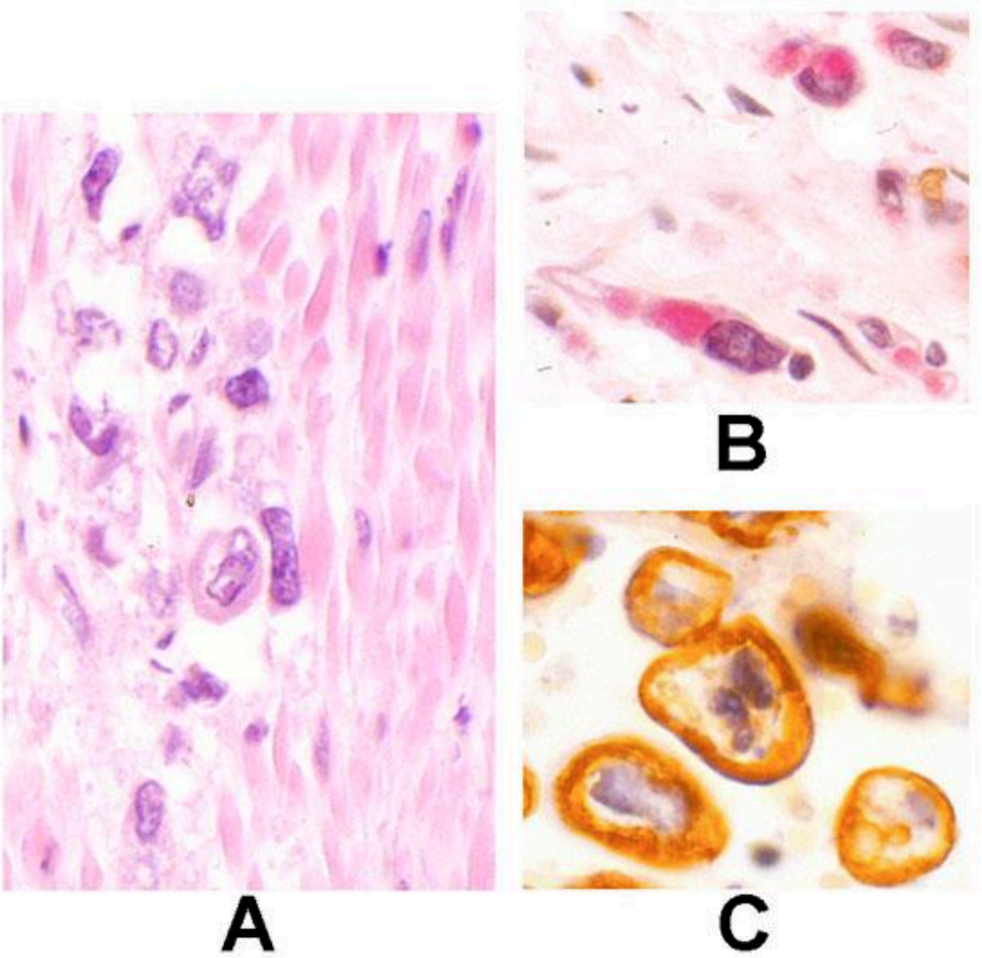

19. An 82-year-old woman has a 7-month history of increasing indigestion, upper abdominal bloating, and early satiety. Photomicrographs of her gastric wall are shown: A is a hematoxylin and eosin stain; B is a mucin stain; C is an immunostain for cytokeratin. Which of the following is most likely to have decreased expression in these cells?

(A) Cathepsin D
(B) Epithelial cadherins
(C) Heparin-binding fibroblast growth factors
(D) Integrins
(E) Type IV collagenase
(F) Vascular endothelial growth factor

---

20. A 54-year-old woman comes to the physician because she would like to lose weight. She has been on numerous diets in the past with limited success. Both her parents have type 2 diabetes mellitus. She is 160 cm (5 ft 3 in) tall and weighs 69 kg (152 lb); BMI is 27 kg/m^2. Her blood pressure is 140/90 mm Hg. Fasting serum glucose concentration is 102 mg/dL. Compared with a woman of the same age whose weight is normal, which of the following serum abnormalities is most likely in this patient?

(A) Decreased cholesterol excretion
(B) Decreased estrone concentration
(C) Decreased leptin concentration
(D) Increased fasting insulin concentration
(E) Increased growth hormone concentration
(F) Increased thyroid-stimulating hormone concentration

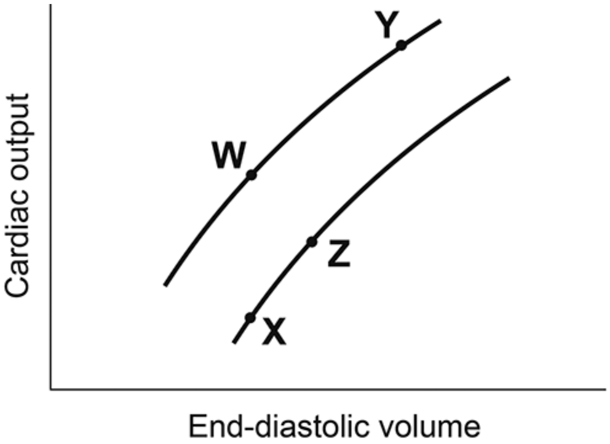

21. A 65-year-old woman is brought to the emergency department because of a 10-minute history of chest tightness and severe pain of her left arm. Physical examination shows jugular venous distention. Crackles are heard over the lung fields. An ECG shows ST-segment elevation greater than 1 mm in leads V_4 through V_6 and new Q waves. Serum studies show an increased troponin I concentration. Which of the following labeled points in the graph best represents the changes in cardiac function that occurred during the first 10 seconds after the onset of pain in this patient?

(A) W → X
(B) W → Y
(C) W → Z
(D) X → W
(E) X → Y
(F) X → Z
(G) Z → W
(H) Z → X
(I) Z → Y

---

22. A previously healthy 33-year-old woman is brought to the emergency department by the Secret Service for stalking the president of the USA for 2 months. She claims to be married to the president's twin brother and states that the president just had his twin kidnapped to avoid competition. She speaks rapidly and is difficult to interrupt. Her associations are often loose. She says, "I haven't slept for days, but I won't even try to sleep until my husband is rescued. God has been instructing me to take over the White House. I can't wait to be reunited with my husband. I hear his voice telling me what to do." When asked about drug use, she says she uses only natural substances. She refuses to permit blood or urine tests, saying, "I don't have time to wait for the results." Which of the following is the most likely diagnosis?

(A) Bipolar disorder, manic, with psychotic features
(B) Brief psychotic disorder
(C) Delusional disorder
(D) Psychotic disorder due to general medical condition
(E) Schizophrenia

23. In informing a couple that their newborn has Down syndrome, there is a specific, relatively limited amount of information that the consulting physician should give immediately. The rest can be discussed at a later time. Which of the following best explains the purpose of using this approach to disclosure?

(A) Allowing the couple's primary care physician to discuss most of the information with them
(B) Allowing the parents time to tell other family members
(C) Delaying parental distress until the information is completely disclosed
(D) Disclosing the most important information so that it can be understood as fully as possible
(E) Influencing the parents' course of action about what is medically most appropriate

24. A 62-year-old man comes to the physician because of a 6-month history of urinary hesitancy and dribbling after urination. He has to urinate two to three times nightly. Physical examination shows a diffusely enlarged, firm, and nontender prostate. Which of the following is most likely to have contributed to the development of this patient's condition?

(A) Activation of the α_1-adrenergic receptor
(B) Conversion of testosterone to dihydrotestosterone
(C) Conversion of testosterone to estradiol
(D) Inhibition of the α_1-adrenergic receptor
(E) Production of prostate-specific antigen

25. A 19-year-old man who is in the US Army is brought to the emergency department 45 minutes after he sustained a knife wound to the right side of his chest during an altercation. He has no history of major medical illness and takes no medications. His temperature is 36.9°C (98.4°F), pulse is 110/min, respirations are 24/min, and blood pressure is 114/76 mm Hg. Pulse oximetry on room air shows an oxygen saturation of 94%. On physical examination, the trachea appears to be shifted to the left. Pulmonary examination of the right chest is most likely to show which of the following findings?

	**Fremitus**	**Percussion**	**Breath Sounds**
(A)	Decreased	dull	decreased
(B)	Decreased	hyperresonant	decreased
(C)	Decreased	hyperresonant	dull
(D)	Increased	dull	bronchial
(E)	Increased	dull	decreased

---

26. A 34-year-old man comes to the office because of a 1-month history of diarrhea. He has a history of pheochromocytoma treated 2 years ago. His mother is being treated for a tumor of her parathyroid gland. He has no other history of major medical illness and takes no medications. His temperature is 37.0°C (98.6°F), pulse is 84/min, respirations are 10/min, and blood pressure is 120/75 mm Hg. Pulse oximetry on room air shows an oxygen saturation of 97%. Vital signs are within normal limits. Physical examination shows a 3-cm, palpable mass on the right side of the neck. A biopsy specimen of the mass shows a neuroendocrine neoplasm of parafollicular cell origin. The most likely cause of the findings in this patient is a mutation in which of the following types of genes?

(A) Cell cycle regulation gene
(B) DNA mismatch repair gene
(C) Metastasis suppressor gene
(D) Proto-oncogene
(E) Tumor suppressor gene

27. A 56-year-old man is brought to the emergency department by his wife 30 minutes after he had severe upper back pain and hoarseness before becoming comatose. He has hypertension treated with hydrochlorothiazide. His temperature is 37°C (98.6°F), pulse is 100/min, and blood pressure is 160/80 mm Hg in the right arm and 100/60 mm Hg in the left arm. Ophthalmologic examination shows ptosis and miosis of the left eye. There is anhidrosis of the left side of the forehead, right hemiplegia, and a decreased left radial pulse. A chest x-ray shows a widened mediastinum. Which of the following conditions is the most likely cause of these findings?

(A) Dissection of the aorta distal to the left subclavian artery
(B) Dissection of the aorta extending into the left carotid artery
(C) Dissection of the aorta extending into the left carotid artery and distal aortic arch
(D) Dissection of the proximal aorta extending into the right subclavian artery
(E) Superior sulcus tumor
(F) Thrombus of the left carotid artery

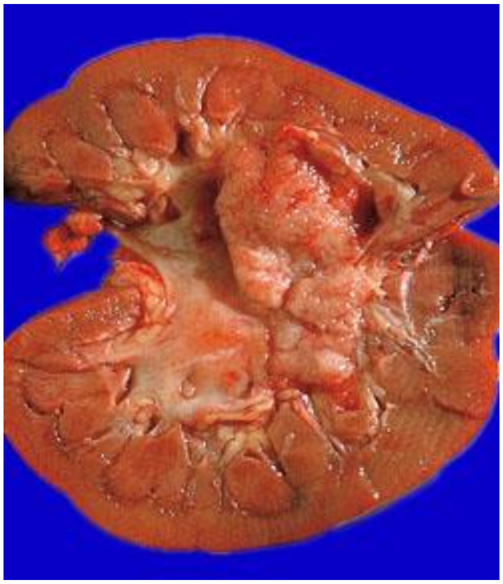

28. A 68-year-old man with alcohol use disorder comes to the office because of a 3-month history of intermittent blood in his urine; he has had no pain. He is a retired laboratory technician from a company that produces naphthylamine. He has smoked 1½ packs of cigarettes daily for 45 years. A CT scan of the abdomen shows a mass in the pelvis of the left kidney. A photograph of the surgically resected kidney is shown. The neoplastic process in this kidney is most likely to be which of the following?

(A) Angiomyolipoma
(B) Metastatic melanoma
(C) Nephroblastoma
(D) Oncocytoma
(E) Urothelial carcinoma

---

29. A 27-year-old woman comes to the emergency department because of a 1-hour history of severe shortness of breath. She has just returned from a cross-country flight. She has a history of borderline hypertension. Her temperature is 36.9°C (98.5°F), pulse is 113/min, respirations are 28/min, and blood pressure is 138/85 mm Hg. Physical examination shows that the right calf has an increased circumference compared with the left calf, and there is tenderness behind the right knee. Which of the following is the most likely underlying cause of this patient's condition?

(A) Antithrombin III deficiency
(B) Factor V Leiden mutation
(C) Glanzmann thrombasthenia
(D) Protein C deficiency
(E) von Willebrand disease

30. A 20-year-old woman is brought to the urgent care center because of a 2-month history of progressive weakness of her arms. She also has a 1-week history of moderate back pain and headache. Her only medication is ibuprofen as needed for pain. Muscle strength is 3/5 in the upper extremities. Sensation to pinprick is decreased over the upper extremities. MRI of the spine shows a central syrinx in the cervical spinal cord. It is most appropriate to obtain specific additional history regarding which of the following in this patient?

(A) Diet
(B) Family illness
(C) Recent travel
(D) Trauma
(E) Unintended weight loss

31. A 3-year-old boy is brought to the office because of a 2-day history of bulging of his left eye. He says his eye hurts. He has no history of major medical illness or recent trauma to the area, and he receives no medications. Vital signs are within normal limits. Physical examination shows exophthalmos of the left eye. MRI of the brain shows a 2-cm mass involving the ocular muscles of the left eye. A biopsy specimen of the mass shows malignant cells, some of which have striations. Which of the following is the most likely diagnosis?

(A) Neuroblastoma
(B) Pheochromocytoma
(C) Retinoblastoma
(D) Rhabdomyosarcoma
(E) Thyroid cancer

32. An 18-year-old woman with sickle cell disease is brought to the emergency department by her parents because of a 2-hour history of severe abdominal pain and nausea. Her parents say that she had a cheeseburger, milk shake, and chocolate bar for lunch. Her temperature is 37°C (98.6°F). Physical examination shows tenderness over the right upper quadrant of the abdomen, radiating to the right shoulder. Ultrasonography of the right upper quadrant of the abdomen shows gallstones. Which of the following is the most likely underlying cause of this patient's current condition?

(A) Decreased hepatic secretion of lecithin
(B) Decreased reabsorption of bile salts
(C) High ratio of cholesterol to bile acids in bile
(D) Infestation with parasites secreting β-glucuronidase
(E) Overload of unconjugated bilirubin

33. In a sample of 100 individuals, the mean leukocyte count is $7500/mm^3$, with a standard deviation of $1000/mm^3$. If the leukocyte counts in this population follow a normal (gaussian) distribution, approximately 50% of individuals will have which of the following total leukocyte counts?

(A) $5500–9500/mm^3$
(B) $<6500/mm^3$ or $>8500/mm^3$
(C) $6500–8500/mm^3$
(D) $<7500/mm^3$
(E) $>9500/mm^3$

34. A previously healthy 52-year-old woman comes to the physician because of a 2-month history of fatigue, constipation, and frequent urination. Her temperature is 37.1°C (98.8°F), pulse is 80/min, respirations are 14/min, and blood pressure is 140/90 mm Hg. Diffuse crackles are heard bilaterally. Her serum calcium concentration is 11.1 mg/dL, and serum parathyroid hormone concentration is decreased. A chest x-ray shows bilateral hilar lymphadenopathy and interstitial infiltrates. Which of the following is the most likely cause of this patient's hypercalcemia?

(A) Calcitriol production by activated macrophages
(B) Local resorption of bone by metastases
(C) Parathyroid hormone-related peptide secretion
(D) Secretion of parathyroid hormone
(E) Secretion of thyroid-stimulating hormone

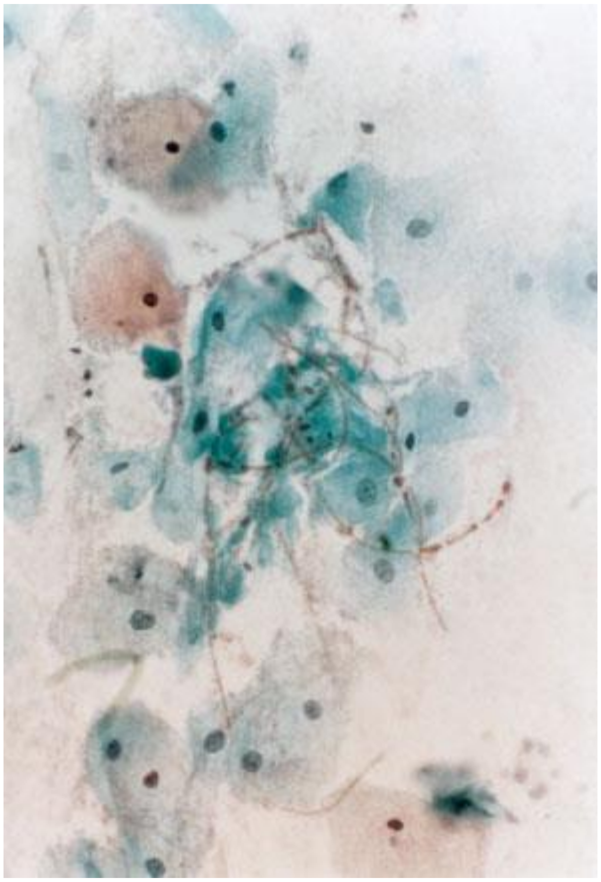

35. A 55-year-old woman comes to the clinic because of a 2-month history of increasingly severe vaginal pain and itching during sexual intercourse. She avoids intercourse with her husband because of the symptoms. She has been in a monogamous relationship with her husband for the past 25 years. She has type 2 diabetes mellitus. Her vital signs are within normal limits. Pelvic examination shows edematous and erythematous vaginal mucosa with white discharge. A photomicrograph of a vaginal smear is shown. Which of the following is the most likely causal infectious agent?

(A) *Candida albicans*
(B) *Chlamydia trachomatis*
(C) Herpes simplex virus
(D) Human papillomavirus
(E) *Trichomonas vaginalis*

---

36. A 24-year-old woman is brought to the physician 1 month after she was involved in a motor vehicle collision that left her weak and unable to walk. Physical examination shows weakness of both hands and atrophy of the intrinsic hand muscles bilaterally. There is weakness and increased muscle tone of the lower extremities on passive range of motion. Deep tendon reflexes are normal at the biceps and triceps bilaterally and are increased at the knees and ankles. Babinski sign is present bilaterally. Sensation to pinprick is absent at and below the level of the clavicles. The lesion in this patient is most likely located at which of the following spinal cord levels?

(A) C5
(B) C7
(C) T1
(D) T3
(E) T5

37. A 45-year-old man is brought to the emergency department 30 minutes after the sudden onset of crushing chest pain. His father, maternal aunt, and paternal uncle all died of myocardial infarctions under the age of 50 years. Physical examination shows tendinous xanthomas on the hands and thickened Achilles tendons. Serum lipid studies show a total cholesterol concentration of 410 mg/dL, HDL-cholesterol concentration of 30 mg/dL, and triglyceride concentration of 140 mg/dL. The diagnosis of myocardial infarction is made. This patient most likely has a deficiency of which of the following?

(A) Apo B48
(B) Apo C
(C) HMG-CoA reductase activity
(D) LDL receptor
(E) Lipoprotein lipase activity

38. A previously healthy 19-year-old man is brought to the emergency department 30 minutes after he collapsed while playing softball. He had severe, sharp, upper back pain prior to the game. He is 196 cm (6 ft 5 in) tall. His temperature is 37°C (98.6°F), pulse is 130/min, respirations are 24/min, and blood pressure is 80/50 mm Hg. Physical examination shows pallor and no jugular venous distention. Breath sounds are clear. The carotid pulses are weak. A grade 4/6, late diastolic murmur is heard at the lower left sternal border. Which of the following is the most likely cause of this patient's cardiac findings?

(A) Atrial septal defect
(B) Mitral stenosis
(C) Papillary muscle rupture
(D) Perforated tricuspid valve
(E) Stretched aortic anulus

39. A 27-year-old woman is brought to the emergency department because of a 2-week history of double vision. Neurologic examination shows that the left eye does not adduct past the midline on horizontal gaze when the patient looks to the right. Leftward horizontal gaze is normal. This patient's ocular movement deficit is most likely caused by damage to which of the following structures?

(A) Left abducens nerve
(B) Left medial longitudinal fasciculus
(C) Left nucleus of the abducens nerve
(D) Right abducens nerve
(E) Right medial longitudinal fasciculus
(F) Right nucleus of the abducens nerve

40. A 45-year-old woman is recovering from acute respiratory distress syndrome secondary to gallstone pancreatitis. A drawing of the alveolar wall is shown. Which of the following labeled cells will most likely proliferate and reestablish the injured epithelial layer in this patient?

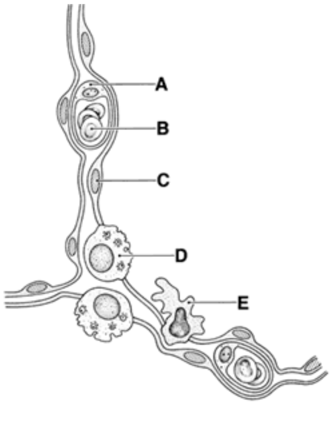

USMLE STEP 1 SAMPLE TEST QUESTIONS

BLOCK 2, ITEMS 41-80

41. An 11-year-old girl is brought to the emergency department by her parents because of a 1-week history of breast enlargement. She has not had pain or nipple discharge. She has asthma treated with inhaled albuterol as needed. She does not smoke cigarettes, drink alcoholic beverages, or use illicit drugs. She is at the 50th percentile for height and weight. Vital signs are within normal limits. Examination of the breasts shows minimal enlargement under the areolae and mild enlargement of the diameter of the areolae without nipple discharge; no masses are palpated. There is scant pubic hair. Which of the following best describes the sexual maturity rating for this patient?

(A) 1
(B) 2
(C) 3
(D) 4
(E) 5

42. A 5-year-old boy is brought to the emergency department by his mother because of an episode of bloody stool 3 hours ago. The mother says the stool was hard "like pebbles" and she noted bright red blood on the tissue when the patient cleaned himself. His previous bowel movement was 5 days ago. The patient has no abdominal or rectal pain now, but he did have abdominal pain during his bowel movement 5 days ago. He has no history of major medical illness and receives no medications. Vaccinations are up-to-date. The patient has no recent history of travel. He is at the 5th percentile for height and the 10th percentile for weight; BMI is at the 50th percentile. Vital signs are within normal limits. Abdominal examination shows hypoactive bowel sounds and a soft, slightly distended abdomen that is not tender to palpation. Rectal examination shows 1 cm of bright red rectal mucosa protruding from the right side of the anus; there is no rectal bleeding. The remainder of the examination shows no abnormalities. Which of the following is the most likely cause of this patient's physical findings?

(A) Constipation
(B) Cystic fibrosis
(C) Hirschsprung disease
(D) Hookworm infestation
(E) Intussusception

43. A 25-year-old woman comes to the office because of a 3-day history of fever, chills, severe headache, weakness, muscle pain, loss of appetite, vomiting, diarrhea, and moderate abdominal pain. She is in nursing school and returned from a medical missions trip in West Africa 10 days ago. Her symptoms began abruptly while she was shopping in a supermarket after her return. Temperature is 39.0°C (102.2°F), pulse is 100/min, respirations are 22/min, and blood pressure is 110/70 mm Hg. The patient appears ill and in mild respiratory distress. Physical examination discloses poor skin turgor and hyperactive bowel sounds. Muscle strength is 4/5 throughout. Laboratory studies show leukopenia and thrombocytopenia. Which of the following is the most sensitive and specific test for detection of the suspected viral genome in this patient?

(A) Microarray analysis
(B) Northern blot
(C) Reverse transcription-polymerase chain reaction test
(D) Southern blot
(E) Western blot

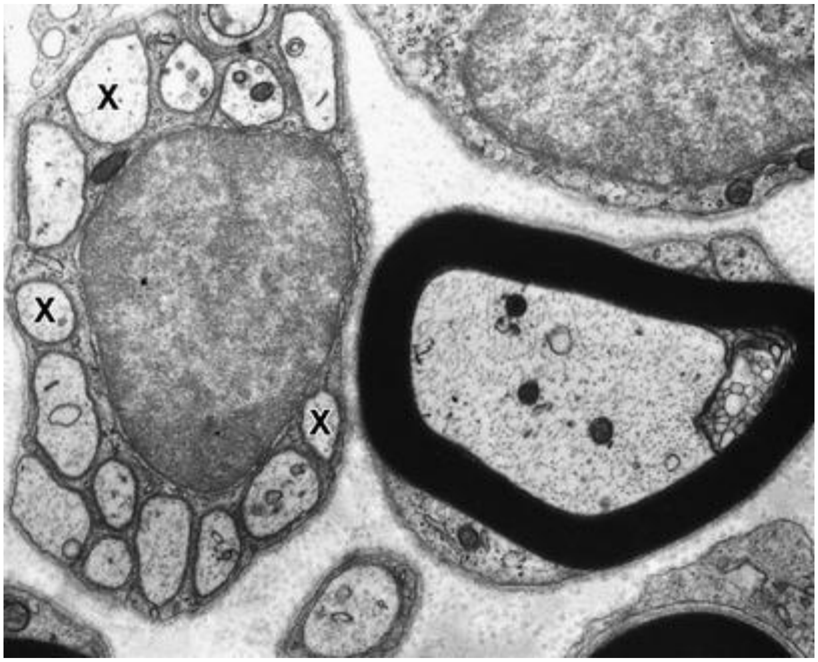

44. A 56-year-old man comes to the office because of a 1-month history of pain and tingling of his hands and a 6-month history of paresthesia of his feet. As part of the workup, a nerve biopsy specimen is obtained and analyzed at the electron microscopic level. The biopsy specimen shows marked loss of structures labeled by the X's in the photomicrograph of a normal biopsy specimen shown. The loss of these structures is most likely to cause which of the following neurologic findings in this patient?

(A) Decreased sensitivity of the deep tendon reflex
(B) Impaired stereognosis
(C) Loss of pain and temperature sensation
(D) Muscle atrophy
(E) Vasoconstriction

---

45. A 5-month-old boy is brought to the clinic by his mother because of a 10-day history of “coughing spells” that occur several times daily and last 1 to 2 minutes; he often vomits afterwards. He was delivered at term to a 16-year-old patient, gravida 1, para 1, following an uncomplicated pregnancy and spontaneous vaginal delivery in Mexico. His parents immigrated to the USA shortly after his birth. He has no history of major medical illness and receives no medications. He has never been to a physician for a well-child examination and has not received any vaccinations. He appears well. Vital signs, including oxygen saturation, are within normal limits. During the physical examination, he coughs uncontrollably for 2 minutes, after which there is a gasping sound and subsequent vomiting. Afterwards, he appears exhausted. Physical examination shows no nasal flaring or intercostal or subcostal retractions. The lungs are clear; no wheezes or crackles are heard. A drug from which of the following classes is most appropriate for this patient?

(A) Cephalosporin
(B) Fluoroquinolone
(C) Macrolide
(D) Penicillin
(E) Sulfonamide

46. A 40-year-old woman comes to the physician because of a runny nose, sneezing, and itching eyes on exposure to cats since childhood. Antiallergy drugs have not provided relief. Her symptoms improve with a program of desensitization involving administration of increasing doses of the allergen. Which of the following is the most likely mechanism of the beneficial effect of this treatment?

(A) Formation of dimers that stimulate clearance by macrophage phagocytosis
(B) Increased production of IgG antibodies that block allergen binding to mast cells
(C) Overwhelming IgE binding sites to result in inactivation of mast cells
(D) Production of an anti-idiotypic antibody to IgE that results in its clearance
(E) Stimulation of macrophages to produce anti-inflammatory cytokines

47. A 3-week-old girl delivered at term with no complications is brought to the physician by her mother because of a 1-week history of yellow eyes and skin, tan-colored stools, and dark brown urine. The newborn has been breast-feeding without difficulty. She is alert and appears to be in no distress. She is at the 50th percentile for length and weight. Physical examination shows scleral icterus and jaundice. There is mild hepatomegaly; the spleen is not palpable. Laboratory studies show:

Hemoglobin	14.4 g/dL
Hematocrit	43%
Leukocyte count	8000/mm^3
Serum	
Albumin	3.5 g/dL
Bilirubin, total	14 mg/dL
Direct	12.5 mg/dL
AST	50 U/L
ALT	45 U/L

Which of the following is the most likely diagnosis?

(A) Biliary atresia
(B) Crigler-Najjar syndrome, type I
(C) Gilbert syndrome
(D) Hemolytic disease of the newborn
(E) Physiologic jaundice

---

48. A 65-year-old woman comes to the physician for a follow-up examination after blood pressure measurements were 175/105 mm Hg and 185/110 mm Hg 1 and 3 weeks ago, respectively. She has well-controlled type 2 diabetes mellitus. Her blood pressure now is 175/110 mm Hg. Physical examination shows no other abnormalities. Antihypertensive therapy is started, but her blood pressure remains elevated at her next visit 3 weeks later. Laboratory studies show increased plasma renin activity; the erythrocyte sedimentation rate and serum electrolytes are within the reference ranges. Angiography shows a high-grade stenosis of the proximal right renal artery; the left renal artery appears normal. Which of the following is the most likely diagnosis?

(A) Atherosclerosis
(B) Congenital renal artery hypoplasia
(C) Fibromuscular dysplasia
(D) Takayasu arteritis
(E) Temporal arteritis

49. A 46-year-old woman comes to the physician because of a 1-month history of fatigue, weakness, and palpitations. She has advanced kidney disease as a result of continued use of combination analgesics for low back pain. She has hypertension well controlled with a loop diuretic, a β-adrenergic blocker, and a dihydropyridine calcium channel blocker. She has a history of gastritis and hemorrhoids associated with occult blood in the stools. She is in no acute distress. Her temperature is 36.8°C (98.2°F), pulse is 74/min, respirations are 14/min, and blood pressure is 150/86 mm Hg. Physical examination shows mild midepigastric tenderness to palpation and 2+ edema of the lower extremities. Cardiac examination shows an S_4 gallop. Test of the stool for occult blood is positive. Laboratory studies show:

Hemoglobin	8.8 g/dL
Hematocrit	26.8%
Mean corpuscular volume	82 μm^3
Serum	
Ferritin	262 ng/mL
Folate	284 ng/mL (N=150–450)
Total iron	60 µg/dL
Transferrin saturation	22% (N=20%–50%)
Lactate dehydrogenase	62 U/L

Which of the following is the most likely cause of this patient's anemia?

(A) Bone marrow suppression
(B) Decreased erythropoietin production
(C) Intravascular hemolysis
(D) Iron deficiency anemia
(E) Splenic sequestration

---

50. A 46-year-old woman comes to the physician because of a 3-day history of intermittent pain with urination and increased urinary frequency. She says that she had one similar episode during the past 6 months. She also has had irregular menses, and her last menstrual period occurred 2 months ago. She has not had fever, nausea, vomiting, or blood in her urine. She is sexually active with one male partner. Physical examination shows no abnormalities. Urinalysis shows:

RBC	3–5/hpf
WBC	10–20/hpf
Nitrites	positive
Leukocyte esterase	positive
Bacteria	positive

Which of the following is the strongest predisposing risk factor for the development of this patient's condition?

(A) Leiomyomata uteri
(B) Perimenopause
(C) Pregnancy
(D) Sexual intercourse

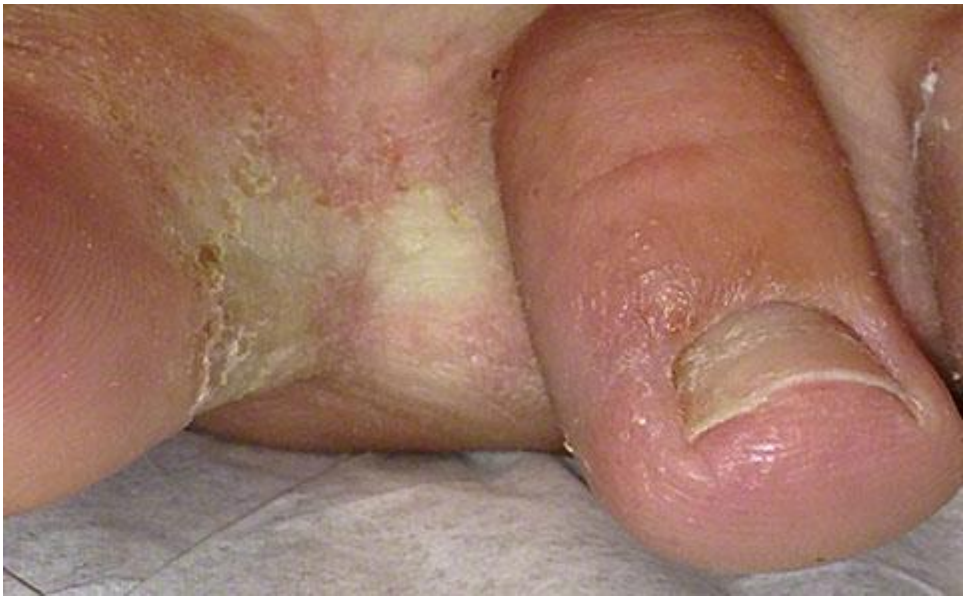

51. A 37-year-old man comes to the office because of a 3-week history of itchy patches of skin and a burning sensation in his feet. He works as a dishwasher and says he frequently works in wet shoes and socks. Vital signs are within normal limits. Physical examination shows the findings in the photograph; these findings are also present on the soles of both feet. The most appropriate pharmacotherapy for this patient will inhibit the activity of which of the following enzymes?

(A) Chitin synthase
(B) 1,3-β-D-Glucan synthase complex
(C) Phospholipase D
(D) Squalene monooxygenase
(E) Thymidylate synthase

---

52. A 10-year-old boy is brought to the emergency department in the middle of summer because of fever, headache, and photophobia. Several of his camp mates have had a similar illness. Physical examination shows mild nuchal rigidity. A lumbar puncture is performed. Laboratory studies show:

Serum glucose	90 mg/dL
Cerebrospinal fluid	
Pressure, opening	50 mm H_2O
Glucose	65 mg/dL
Total protein	70 mg/dL
Leukocyte count	43/mm^3 (95% lymphocytes)

Which of the following infectious agents is the most likely cause of these findings?

(A) Adenovirus
(B) Enterovirus
(C) Herpes simplex virus
(D) *Neisseria meningitidis*
(E) *Streptococcus pneumoniae*

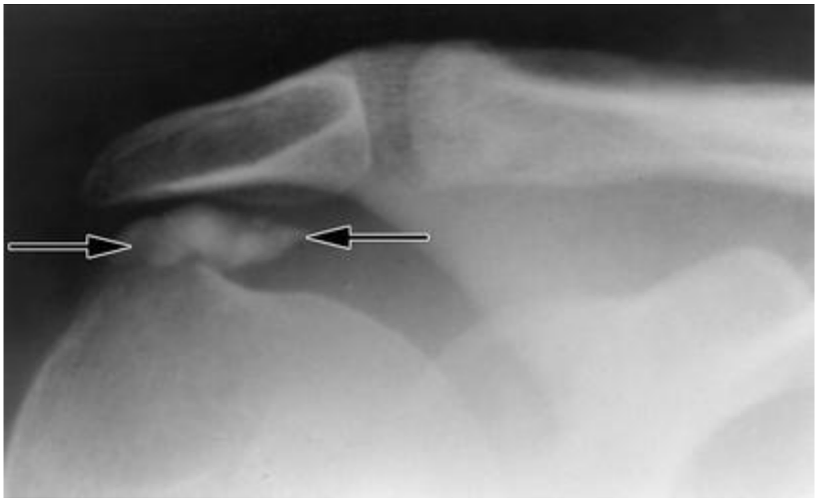

53. A 55-year-old woman comes to the physician because of a 3-week history of right shoulder pain that occurs when she is lying on her right shoulder in bed. There is tenderness to palpation over the greater tubercle. An x-ray of the right shoulder is shown; the arrows indicate a calcium deposit. Further examination of this patient will most likely show impairment of which of the following active shoulder movements?

(A) Abduction
(B) Adduction
(C) External rotation
(D) Flexion
(E) Internal rotation

---

54. A 31-year-old woman with a 5-year history of fatigue comes to the physician for an initial visit. She has seen four other physicians for the same condition within the past 6 months, but no abnormalities were found. She gives the physician a large folder that contains her medical records. She says, "I can barely get out of bed most mornings, but nobody can figure out why because all of my tests turn out normal. I really think I have chronic fatigue syndrome. What do you know about this condition?" The physician has not treated any patient with chronic fatigue syndrome before. Which of the following initial statements by the physician is most appropriate to establish rapport with this patient?

(A) "From the size of the folder you brought, it looks like you've had very thorough examinations in the past."
(B) "I don't have much experience with chronic fatigue syndrome, but I'm committed to learning as much as I can about it."
(C) "I'm not familiar with chronic fatigue syndrome, except that many physicians don't think it's a real disease."
(D) "Let's start over from scratch. We'll need to repeat complete testing."
(E) "When nothing abnormal is found during thorough examinations and laboratory studies, there is often an underlying psychological cause of the symptoms."

55. A 28-year-old man comes to the physician because of a 2-month history of a rash on his wrists and hands. He is a first-year mortuary science student. He also works on his grandfather's farm each weekend. His hobbies include raising homing pigeons and repairing vintage motorcycles. He recently changed to a different type of laundry detergent to save money. Physical examination shows a diffuse erythematous rash involving both hands up to the wrist creases. The rash does not extend over any other parts of the body. Which of the following is the most likely cause of this patient's rash?

(A) Change in laundry detergent
(B) Exposure to chemicals during motorcycle repair
(C) Handling pigeons
(D) Pesticide exposure
(E) Use of latex gloves

56. A 22-year-old woman at 32 weeks' gestation comes to the office for a prenatal visit. Vital signs are within normal limits. Fundal height and fetal heart tones are also normal. At the end of the examination, the patient tells the physician that her friend is a certified lay midwife and has recently convinced her to have a home birth. The patient asks the physician if he would be the backup for the midwife in case of an emergency. Which of the following is the most appropriate initial response by the physician?

(A) "I'm sorry, but I would no longer be able to be your doctor if you pursue a home birth."
(B) "I would like to meet with your friend before I decide."
(C) "If there's a problem, I could still act as your doctor if you arrive at the hospital in labor."
(D) "Let's set up an appointment next week to discuss your birth plan in detail."
(E) "Perhaps your midwife could act as your birth coach instead."

57. A 36-year-old woman with hypertension comes to the office because she thinks she may be "going through early menopause." She has not had a menstrual period since her most recent office visit 6 months ago. During this time, she also has been "gaining weight around the middle" despite increased exercise; she has had a 6.3-kg (14-lb) weight gain. She has no other history of major medical illness. Her only medication is lisinopril. She does not smoke, drink alcohol, or use illicit drugs. She is 168 cm (5 ft 6 in) tall and weighs 107 kg (236 lb); BMI is 38 kg/m^2. Vital signs are within normal limits. Examination shows a uterus consistent in size with a 24-week gestation. Pelvic ultrasonography shows oligohydramnios and a fetus with a misshapen cranium, pericardial effusion, small bladder, and echogenic bowel. The most likely cause of the fetal abnormalities in this patient's pregnancy is interference with which of the following?

(A) Fetal lung/epithelial differentiation
(B) Fetal lung/surfactant development
(C) Fetal renal hemodynamics
(D) Maternal placental perfusion
(E) Maternal prostaglandin synthesis

58. A 67-year-old man comes to the office because he is concerned about memory loss. He says he sometimes forgets the names of acquaintances he sees while he is out shopping. He also has occasional word-finding difficulty and forgets to buy some items when he goes shopping unless he makes a list. He lives alone and is able to manage his finances, cook, and shop without help. He works part-time as an accountant. He has gastroesophageal reflux disease and hypertension. Current medications are hydrochlorothiazide and omeprazole. Vital signs are within normal limits. Physical and neurologic examinations show no abnormalities. On mental status examination, he is fully oriented. His speech is normal, and thoughts are organized. His mood is euthymic, and he has a full range of affect. His concentration is intact, and he is able to perform calculations quickly and accurately. He can name objects accurately and follow written and verbal commands. He recalls three of four objects after 5 minutes. Which of the following is the most appropriate physician response to this patient's concern?

(A) "I am concerned about your memory loss. Let's discuss how to further evaluate your memory."
(B) "There's no need to worry right now, but let's meet again in 6 months."
(C) "Unfortunately, your memory loss will likely increase significantly during the next 5 years; let's discuss some ways to plan for the future."
(D) "Your episodes of forgetfulness are likely just 'Senior Moments,' but we should obtain in-depth laboratory test results and an MRI to be certain."
(E) "Your examination findings indicate that your memory loss is likely consistent with the normal aging process."

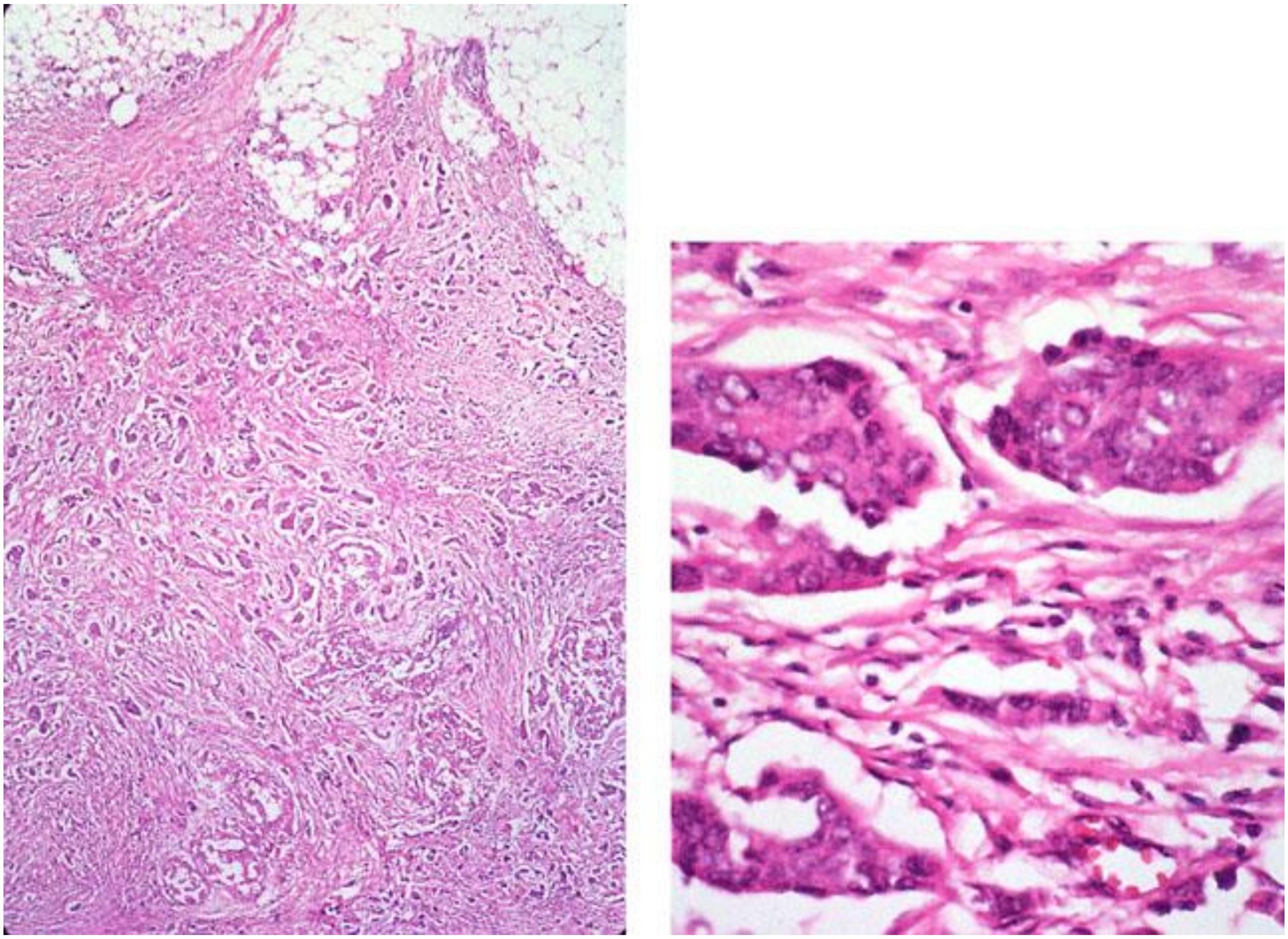

59. A 42-year-old woman comes to the office because of a 1-year history of a left breast mass. She had been living in another country for the past 2 years; a physician in that country told her that the lesion was a cyst. Since returning to the USA 12 weeks ago, she has noticed that the mass has increased in size and is now so tender that any physical contact is painful. She has not had any other breast masses, skin changes, or nipple changes. She has a history of cysts in both breasts, and has undergone aspirations of 2 prior cysts; pathologic examination both times showed no abnormalities. She has a 1-year history of yellow nipple discharge from both breasts and premenstrual bilateral breast pain. She has no other history of serious illness and takes no medications. There is no family history of breast cancer. Vital signs are within normal limits. Physical examination shows a 3-cm, firm, extremely tender and partially mobile mass in the upper outer quadrant of the left breast. Mammography shows a 3.1-cm, irregular mass in the left breast corresponding to the palpable area of the mass. Photomicrographs of an excisional biopsy specimen are shown. Which of the following pathologic findings most strongly suggests malignancy in this patient?

(A) Compressed ducts
(B) Dense fibrosis
(C) Expanded ducts
(D) Multiple large and small cysts
(E) Pleomorphic irregular glands

---

60. A 30-year-old woman comes to the office because of a 4-day history of an increasingly severe, painful rash over her body and in her mouth. The rash began over her trunk area but spread within a day to her face and extremities. Two days before development of the rash, she had flu-like symptoms with muscle aches and fatigue as well as a nonproductive cough, sore throat, and runny nose. Ten days ago, she began treatment with trimethoprim-sulfamethoxazole for a urinary tract infection; she takes no other medications. Temperature is 39.0°C (102.2°F), pulse is 120/min, respirations are 25/min, and blood pressure is 165/105 mm Hg. Physical examination shows diffuse brownish red macular exanthema with bullous lesions. Epidermis at an uninvolved site can be removed with mild tangential pressure. Examination of a

biopsy specimen of one of the lesions shows necrosis of keratinocytes throughout the epidermis. There is minimal lymphocytic infiltration within the superficial dermis. Which of the following is the most likely diagnosis?

(A) Erythema multiforme
(B) Linear IgA bullous dermatosis
(C) Pemphigus vulgaris
(D) Staphylococcal scalded skin syndrome
(E) Toxic epidermal necrolysis

61. A 78-year-old woman is admitted to the hospital because of a 1-week history of jaundice. CT scan of the abdomen shows a mass suggestive of pancreatic cancer. Three hours later, the on-call physician enters the patient's room to discuss the prognosis and obtain consent for a biopsy scheduled for the next morning. On entering the room, the physician greets the patient and her husband. The physician then learns that the patient speaks only Mandarin. Her husband is fluent in Mandarin and English. The hospital interpreter is not available until tomorrow morning. The patient's husband appears anxious and insists that the physician speaks to him and allows him to serve as an interpreter for his wife. Which of the following is the most appropriate next step in management?

(A) Allow the patient's husband to serve as an interpreter
(B) Consult the hospital ethics committee
(C) Explain to the husband that information cannot be provided until the hospital interpreter arrives in the morning
(D) Use a telephone interpreter service

62. Results of a study that examined the impact of risk factors on cardiovascular health are being evaluated. In the study, serum LDL-cholesterol (LDL) concentration is found to have a correlation of 0.6 with serum high-sensitivity C-reactive protein (hs-CRP) concentration. Which of the following statements best describes the relationship between LDL concentration and hs-CRP concentration based on this finding?

(A) Higher LDL concentrations are associated with higher hs-CRP concentrations
(B) Higher LDL concentrations are associated with lower hs-CRP concentrations
(C) Higher LDL concentrations cause higher hs-CRP concentrations
(D) Higher LDL concentrations cause lower hs-CRP concentrations

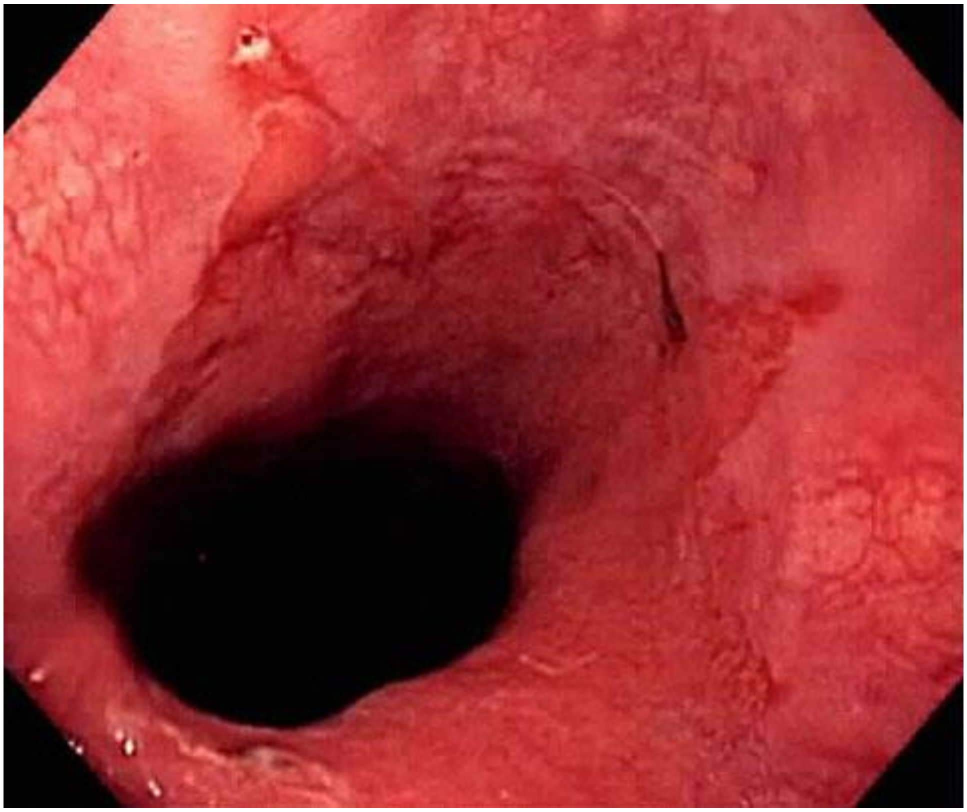

63. A 45-year-old man comes to the physician because of a 10-year history of heartburn that occurs after he eats late at night or consumes spicy food. He has had no weight loss or difficulty swallowing. He takes over-the-counter antacids as needed, but they relieve his discomfort only temporarily. Physical examination shows no abnormalities. An endoscopy is done. The distal esophagus is shown in the photograph. Which of the following is the most likely cause of this patient's symptoms?

(A) Defect in secretin production
(B) Excessive gastrin production
(C) Excessive transient lower esophageal relaxations
(D) Failure of primary esophageal peristalsis
(E) Failure of saliva production

---

64. A 72-year-old man comes to the physician for a health maintenance examination. He has no personal or family history of major medical illnesses. He has never smoked cigarettes. Pulse oximetry on room air shows an oxygen saturation of 98%. Physical examination shows plethoric skin and splenomegaly. Laboratory studies show:

Hemoglobin	21.1 g/dL
Hematocrit	61%
Leukocyte count	15,000/mm^3
Segmented neutrophils	68%
Basophils	4%
Lymphocytes	28%
Platelet count	501,000/mm^3
Leukocyte alkaline phosphatase	increased

A peripheral blood smear shows occasional giant platelets. The primary hematologic defect in this patient most likely occurred in which of the following cells?

(A) Erythroid progenitor
(B) Hematopoietic stem
(C) Lymphatic progenitor
(D) Megakaryocyte progenitor
(E) Pluripotent stem

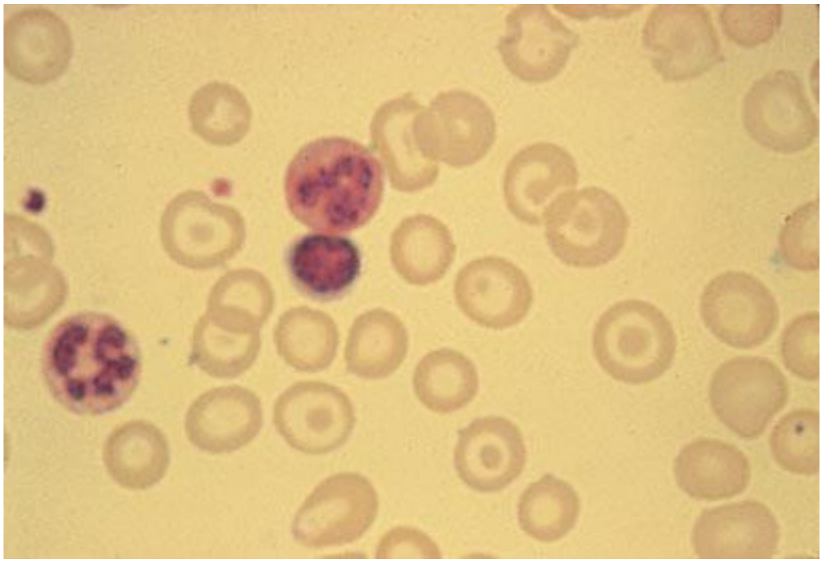

65. A 35-year-old man is brought to the emergency department 30 minutes after he sustained a cut on his hand while loading cargo at his job. He lives alone and takes most of his meals at a local restaurant. He eats mostly snack foods at the bar and fast food. He drinks four to six 12-oz beers daily and double that amount on weekends. He takes no medications. Physical examination shows a 3-cm laceration on the right hand that is bleeding steadily. Laboratory studies show a hemoglobin concentration of 11 g/dL, leukocyte count of 4000/mm^3, and platelet count of 150,000/mm^3. A photomicrograph of a peripheral blood smear is shown. A deficiency of which of the following is the most likely cause of this patient's anemia?

(A) Folic acid
(B) Glucose-6-phosphate dehydrogenase
(C) Iron
(D) Vitamin B_1 (thiamine)
(E) Vitamin B_6 (pyridoxine)

---

66. A 3800-g (8-lb 6-oz) newborn is delivered vaginally at 39 weeks' gestation after an uncomplicated pregnancy. Apgar scores are 9 and 9 at 1 and 5 minutes, respectively. The newborn is crying, has pink skin, and appears vigorous. Physical examination shows a vagina and also a structure that appears to be a penis, with the urethra located at the base near the opening of the vagina. Chromosomal analysis is ordered. Which of the following is the most likely cause of the intersex findings in this newborn if the karyotype is found to be 46,XX?

(A) 17α-Hydroxyprogesterone deficiency
(B) Increased concentration of müllerian-inhibiting substance
(C) Maternal androgen exposure
(D) Presence of the sex-determining region Y gene
(E) 5α-Reductase deficiency

67. An 80-year-old woman is brought to the emergency department (ED) 30 minutes after she fell out of her wheelchair at home. This is the second visit to the ED for a fall during the past 3 months. She lives with her daughter and son-in-law, who say they "do the best we can." The patient has dementia, Alzheimer type, coronary artery disease, type 2 diabetes mellitus, and hypertension. Current medications are amlodipine, aspirin, atorvastatin, donepezil, long-acting insulin, and lisinopril. Five years ago, she underwent bilateral below-the-knee amputations because of infected ulcers of the feet. She uses a wheelchair for ambulation. Ten years ago, she underwent three-vessel coronary artery bypass grafting. She has smoked one-half pack of cigarettes daily for 60 years. She drinks one shot of whiskey nightly. She is thin and appears ill and disheveled. Her temperature is 37.2°C (99.0°F), pulse is 80/min, respirations are 20/min, and blood pressure is 120/80 mm Hg. Pulse oximetry on 2 L/min of oxygen by nasal cannula shows an oxygen saturation of 95%. Physical examination shows temporal wasting. There are scattered ecchymoses over the abdomen and all extremities. No other abnormalities are noted. Which of the following is the most appropriate initial history to obtain from this patient?

(A) Abnormal bleeding
(B) Diet
(C) Relationship with her family
(D) Respiratory symptoms
(E) Urinary symptoms

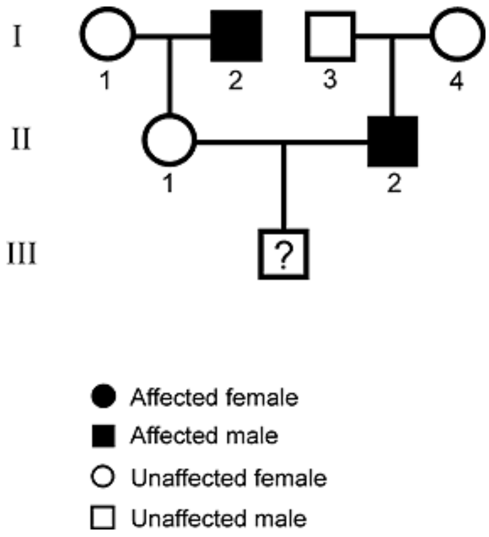

68. A healthy 26-year-old man with red-green color blindness marries a woman whose father also has red-green color blindness. A pedigree is shown. Which of the following best predicts the risk that a son of this couple will be affected?

(A) 0%
(B) 25%
(C) 50%
(D) 75%
(E) 100%

---

69. A 25-year-old woman comes to the physician because of a long history of pain with menses. The pain occurs on the first day of her 5-day menstrual period and lasts all day. She rates the pain as 10 on a 10-point scale. The most appropriate initial pharmacotherapy to relieve this patient's pain has which of the following mechanisms of action?

(A) Inhibition of estrogen synthesis
(B) Inhibition of 11β-hydroxylase activity
(C) Inhibition of prostaglandin synthesis
(D) Stimulation of follicle-stimulating hormone synthesis
(E) Stimulation of luteinizing hormone synthesis

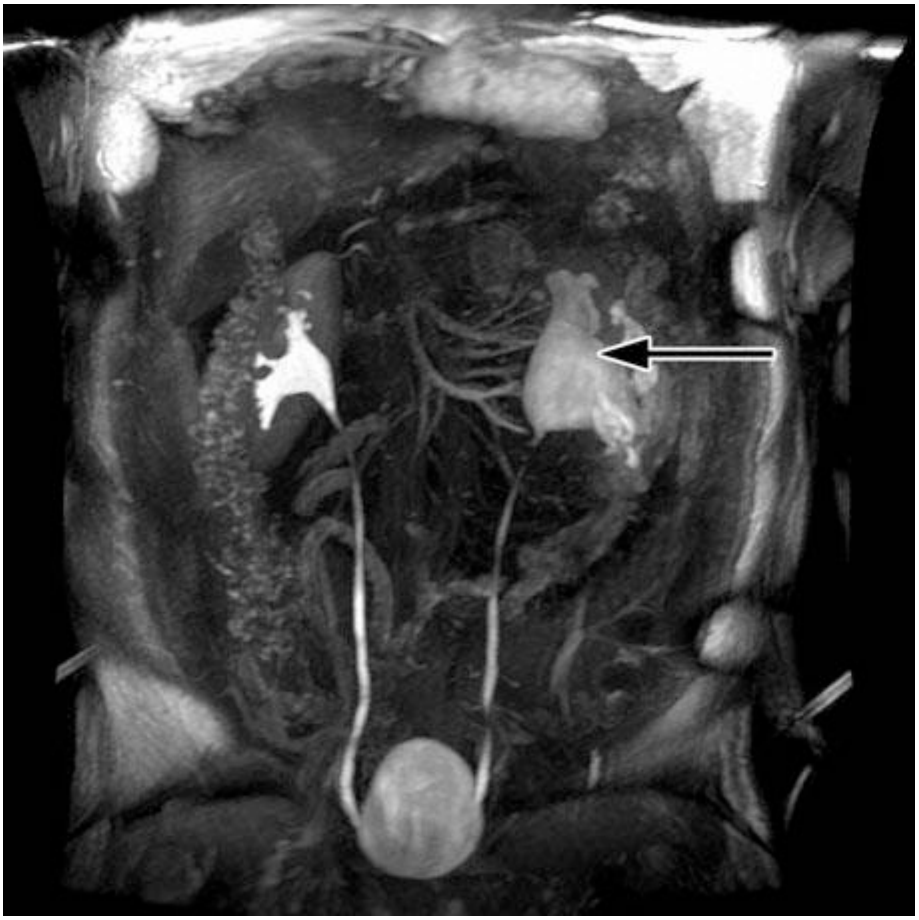

70. A 56-year-old woman comes to the physician because of a 2-year history of recurrent urinary tract infections accompanied by left flank pain. Physical examination shows no abnormalities. Renal ultrasonography shows left-sided hydronephrosis. A T2-weighted coronal MRI of the abdomen is shown; the arrow indicates the hydronephrosis. The left renal collecting system is most likely obstructed at which of the following anatomic locations in this patient?

(A) Bladder neck
(B) Mid ureter
(C) Renal calyx
(D) Ureteropelvic junction
(E) Ureterovesical junction

---

71. A randomized clinical trial is conducted to compare wound healing and cosmetic differences between two surgical procedures for closing skin wounds following cesarean delivery. A total of 1000 women undergoing cesarean delivery during a 6-month period are enrolled in the study, which was 85% of the total number of patients undergoing the procedure. The results show a wound infection rate of 12 cases per 1000 women for Procedure A and 18 cases per 1000 women for Procedure B. Which of the following is the best estimate of the absolute risk reduction for wound infection following Procedure A compared with Procedure B?

(A) (18/1000) – (12/1000)
(B) 0.85 × (12/1000)
(C) 0.85 × (18/1000)
(D) [0.85 × (18/1000–12/1000)]
(E) [(1.2/100) – (1.8/100)]/(1.8/100)

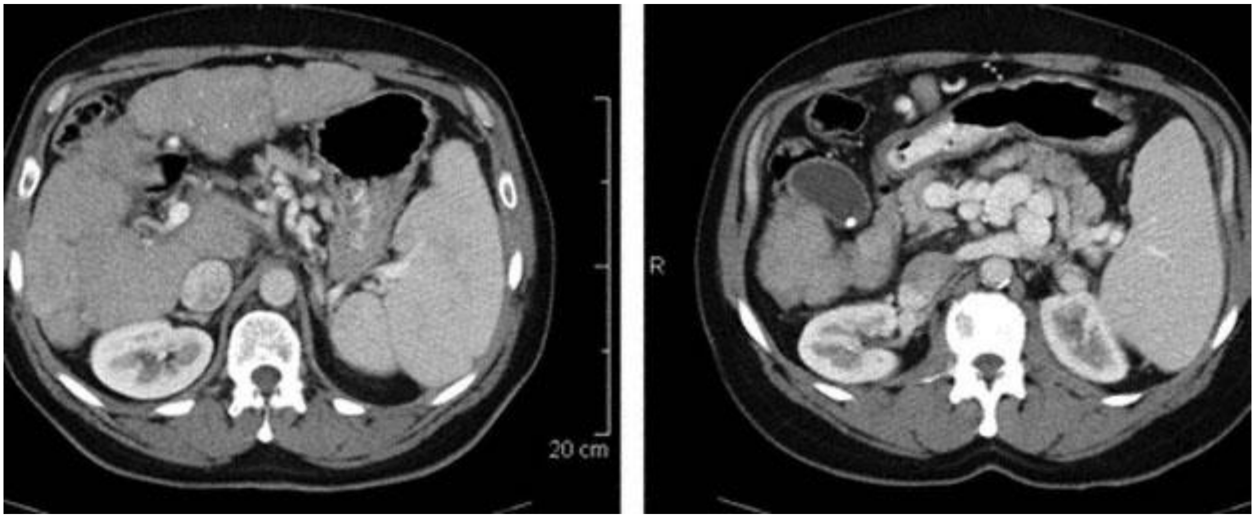

72. A 52-year-old man is admitted to the hospital because of a 2-hour history of vomiting bright red blood. His pulse is 125/min, and blood pressure is 90/60 mm Hg. Physical examination shows jaundice and visible blood vessels surrounding the umbilicus. CT scans of the abdomen are shown. To decrease portal venous pressure in this patient, it is most appropriate to place a shunt between the portal vein and which of the following additional vessels?

(A) Inferior mesenteric vein
(B) Inferior vena cava
(C) Left gastric vein
(D) Splenic vein
(E) Superior mesenteric vein

---

73. A 58-year-old man with chronic obstructive pulmonary disease comes to the clinic with his wife for a follow-up examination. He has smoked one pack of cigarettes daily for 35 years. He has tried to quit smoking twice but was unsuccessful both times. At today's visit, when the physician asks the patient about smoking cessation, he says he is not ready to do so. The patient's wife states her husband's smoking makes her cough and gives her chest tightness. Which of the following is the most appropriate physician statement?

(A) "Are there any reasons why you might want to quit smoking?"
(B) "Are you aware that your lung condition is chronic at this point?"
(C) "I'm sure you don't want your wife to suffer as a result of your smoking."
(D) "The majority of your health issues would improve if you quit smoking."
(E) "Why haven't you been able to stay off cigarettes?"

74. Seven days after admission to the psychiatric unit for treatment of eating disorders, a hospitalized 20-year-old woman has a 2-day history of moderate mouth pain on the inside of both cheeks. She also reports feeling "worthless and fat." Fluoxetine was started on admission. She appears distressed. She is 170 cm (5 ft 7 in) tall and weighs 77 kg (170 lb); BMI is 27 kg/m^2. Vital signs are within normal limits. Physical examination shows bilateral swelling of the parotid and submandibular glands, discoloration of several teeth, and scarring on the dorsum of the right hand. Mental status examination shows no suicidal ideation or intent. Results of laboratory studies are within the reference ranges. A medication with which of the following mechanisms of action is most appropriate to treat this patient's current symptoms?

(A) Binding to muscarinic acetylcholine receptors
(B) Binding to nicotinic acetylcholine receptors
(C) Inhibition of protein synthesis via binding to 50S ribosomal subunits and preventing peptide bond formation
(D) Inhibition of protein synthesis via loss of DNA helical structure
(E) Interference with bacterial wall synthesis

75. A 35-year-old woman, gravida 2, para 1, at 15 weeks' gestation is brought to the hospital in active labor. The physician care team confers and determines that the birth cannot be prevented and that the fetus will not survive. After explaining this fact to the patient, the patient says, "I know it is a long shot, but I still want my baby resuscitated and everything done to save him." After empathizing with the patient, which of the following is the most appropriate initial response by the physician?

(A) "I will ask for a second opinion."
(B) "I will call the father of the baby to get his opinion as well."
(C) "I will call for a medical ethics consultation."
(D) "I will do everything I can to save the baby."
(E) "I'm very sorry, but the baby will not survive."

76. A 48-year-old man who is a contractor interpreter working for the military at a US outpost in Afghanistan comes to the medical clinic because of a 20-day history of nonhealing, painless ulcers on his neck and arms. The lesions enlarged over time and began to express clear fluid, eventually forming shallow ulcers. When the symptoms began, he was sleeping on a mattress on the floor in an old building without air conditioning where the ambient temperature ranged from 21.1°C (70.0°F) to 43.3°C (110.0°F). He originally attributed the lesions to bug bites. Vital signs are within normal limits. Physical examination shows six 2-cm, papular lesions scattered over the neck and upper extremities; each lesion has a 0.6-cm ulcer in the center. There is no pus or exudate. Which of the following vectors is the most likely source of the lesions in this patient?

(A) Flea
(B) Mosquito
(C) Sand fly
(D) Spider
(E) Tick

77. A 17-year-old boy is brought to the clinic for a follow-up examination. He has been evaluated for three episodes of full-body weakness at the ages of 13, 16, and 17 years. Each episode occurred when he lay down after playing in a football scrimmage. The weakness improved spontaneously during the next 6 hours; he was asymptomatic by the time he was evaluated by medical personnel. The patient attributes the episodes to eating "a lot of pasta and salty foods" prior to playing football. Results of a complete blood count and comprehensive metabolic profile following each episode have been within the reference ranges. He has no history of serious illness and takes no medications. Vital signs are within normal limits. Physical and neurologic examinations disclose no abnormalities. Which of the following serum concentrations is most likely to be abnormal if measured during one of this patient's episodes?

(A) Calcium
(B) Chloride
(C) Magnesium
(D) Potassium
(E) Sodium

78. A 3-day-old female newborn is brought to the hospital because of a yellowish, milky fluid leaking from both nipples. She was delivered at term to a 20-year-old woman. Pregnancy and delivery were uncomplicated. Examination of the newborn is otherwise unremarkable. Which of the following hormones is the most likely cause of this finding?

(A) Maternal estrogen
(B) Maternal progesterone
(C) Maternal prolactin
(D) Newborn estrogen
(E) Newborn progesterone
(F) Newborn prolactin

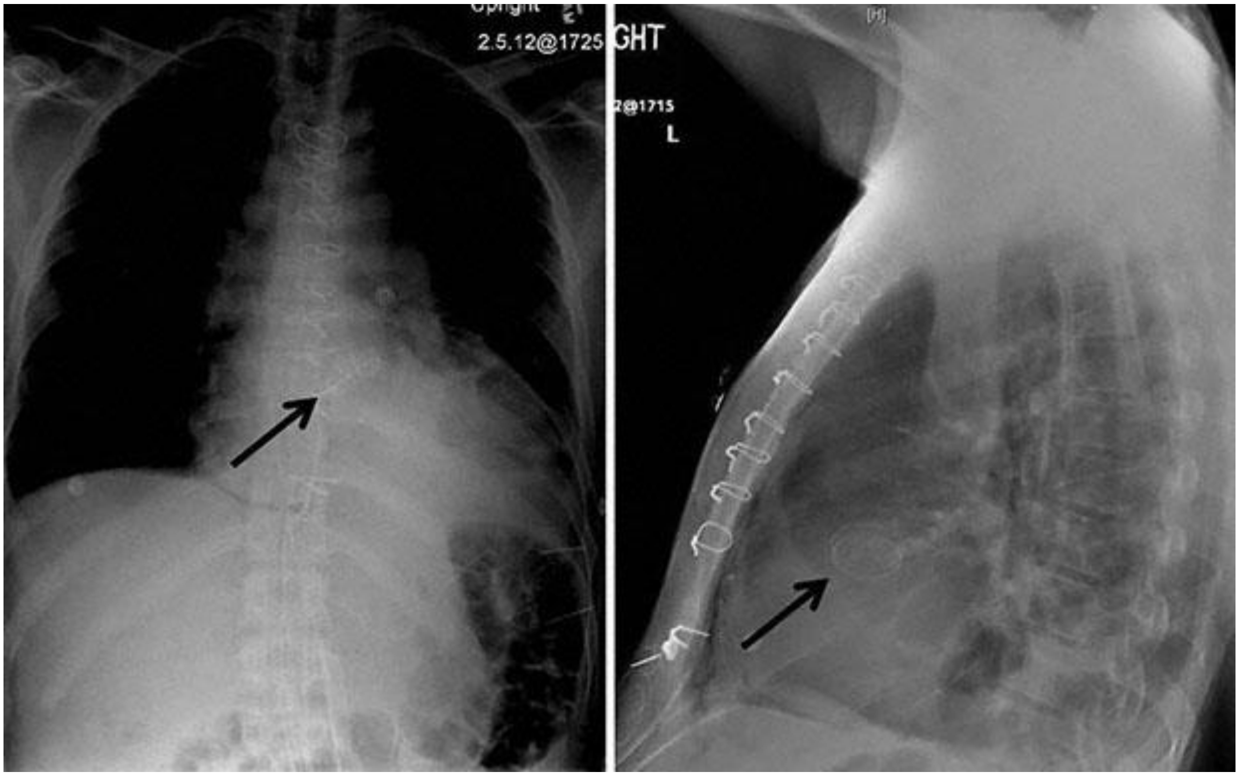

79. A 74-year-old man comes to the office for a follow-up examination 1 month after he was discharged from the hospital following a cardiac valve replacement operation. Physical examination shows a healing median sternotomy wound. A prosthetic click is heard. PA and lateral chest x-rays are shown; the arrows indicate the replaced cardiac valve. Based on these findings, which of the following cardiac valves was most likely replaced in this patient?

(A) Aortic
(B) Mitral
(C) Pulmonic
(D) Tricuspid

---

80. A 37-year-old man who is a carpenter is brought to the emergency department 45 minutes after the sudden onset of fever, shortness of breath, and palpitations. Four days ago, he sustained a puncture wound to his left hand; he treated the wound with antibacterial cream and a bandage. His temperature is 39°C (102.2°F), pulse is 120/min, respirations are 28/min, and blood pressure is 100/60 mm Hg. Examination of the left hand shows diffuse swelling, erythema, and a 2-cm, necrotic puncture wound. His leukocyte count is 14,000/mm^3. Arterial blood gas analysis on room air shows a P_{CO_2} of less than 32 mm Hg. Which of the following is the most likely infectious agent in this patient?

(A) *Clostridium tetani*
(B) *Mycobacterium abscessus*
(C) *Pasteurella multocida*
(D) *Pseudomonas aeruginosa*
(E) *Staphylococcus aureus*

USMLE STEP 1 SAMPLE TEST QUESTIONS

BLOCK 3, ITEMS 81-119

81. A 45-year-old woman comes to the office for a follow-up examination 2 weeks after she sustained a vertebral fracture at L1. The fracture occurred spontaneously and there is no history of trauma to the area or other fractures. She gained 27 kg (60 lb) during the 6 months before the fracture occurred. Her only medication is hydromorphone as needed for pain. She is 163 cm (5 ft 4 in) tall and now weighs 100 kg (220 lb); BMI is 38 kg/m^2. Temperature is 37.0°C (98.6°F), pulse is 86/min, respirations are 12/min, and blood pressure is 145/98 mm Hg. Physical examination shows central obesity and purple striae over the abdomen bilaterally. The lower extremities appear thin. Results of laboratory studies are shown:

Plasma	
Renin activity	5.0 ng/mL/h (N=0.6–4.0)
Metanephrine	0.3 nmol/L (N<0.4)
Serum	
Cortisol, random	43 µg/dL
Adrenocorticotropic hormone	120 pg/mL (N<120)
Aldosterone	8 ng/dL (N=2–9)
Urine 24-hour free cortisol excretion	340 µg/24 h (N=3.5–45)

The most likely cause of the fracture in this patient is an increase in which of the following processes?

(A) Calcium absorption
(B) Calcium excretion
(C) Osteoblast proliferation
(D) Osteoclast proliferation
(E) Phosphorus absorption
(F) Phosphorus excretion

---

82. A 25-year-old woman comes to the emergency department because of a 3-hour history of fever, severe headache, light-headedness, dizziness, shaking chills, and muscle aches. Five hours ago, she was diagnosed with Lyme disease and began doxycycline therapy. She has no other history of serious illness and takes no other medications. Menses occur at regular 28-day intervals. She is currently menstruating and using a tampon. She appears anxious. Temperature is 37.0°C (98.6°F), pulse is 120/min, respirations are 30/min, and blood pressure is 90/60 mm Hg. Pulse oximetry on room air shows an oxygen saturation of 94%. Physical examination shows flushing and diaphoresis. Cardiopulmonary examination shows no other abnormalities. Which of the following is the most likely mechanism of this patient's current condition?

(A) Exacerbation of infection by *Borrelia burgdorferi*
(B) Infection-mediated sepsis
(C) IgE-mediated allergic reaction to doxycycline
(D) Release of bacterial products producing acute inflammation
(E) Secretion of bacterial endotoxins

83. A newborn delivered at 36 weeks' gestation to a 22-year-old woman, gravida 1, para 1, has difficulty feeding and listlessness. The mother received no prenatal care. Spontaneous vaginal delivery was uncomplicated. The mother's only medication was a prenatal vitamin. The newborn's length is 49 cm (19 in; 39th percentile), and weight is 3100 g (6 lb 13 oz; 30th percentile); head circumference is 33 cm (13 in; 12th percentile). Temperature is 37.0°C (98.6°F), pulse is 134/min, respirations are 38/min, and blood pressure is 73/50 mm Hg. Physical examination shows ambiguous genitalia.

Results of serum studies are shown:

Na^+	133 mEq/L
K^+	5.0 mEq/L (N=3.2–5.5)
Cl^-	103 mEq/L
HCO_3^-	17 mEq/L
Glucose	42 mg/dL (N=30–60)

The most appropriate pharmacotherapy for this patient targets which of the following receptors?

(A) Adrenocorticotropic hormone
(B) Aldosterone
(C) Androgen
(D) Gonadotropin-releasing hormone
(E) Growth hormone

---

84. A 60-year-old woman is brought to the emergency department because of a 4-day history of fever, joint aches, and rash. Three weeks ago, she was admitted to the hospital for treatment of *Staphylococcal aureus* endocarditis. She has received 21 days out of a prescribed 42-day course of intravenous oxacillin. Currently, she appears to be in mild distress. Temperature is 38.0°C (100.4°F), pulse is 115/min, respirations are 24/min, and blood pressure is 120/70 mm Hg. Pulse oximetry on room air shows an oxygen saturation of 97%. Physical examination shows a diffuse maculopapular rash over the trunk and upper and lower extremities. There is no pus or erythema at the skin insertion site of the peripherally inserted central catheter line initially placed on the day of hospital discharge. Results of laboratory studies are shown:

Hemoglobin	11.1 g/dL
Hematocrit	33%
Leukocyte count	12,100/mm^3
Segmented neutrophils	78%
Eosinophils	9%
Lymphocytes	7%
Monocytes	6%
Platelet count	341,000/mm^3
Serum	
Na^+	133 mEq/L
K^+	6.5 mEq/L
Cl^-	100 mEq/L
HCO_3^-	15 mEq/L
Urea nitrogen	65 mg/dL
Glucose	96 mg/dL
Creatinine	5.7 mg/dL

Urine microscopy shows eosinophils and WBC casts. Which of the following is the most likely underlying cause of this patient's condition?

(A) Collapsing focal segmental glomerulosclerosis
(B) Glomerular hypertrophy with hemorrhage and necrosis
(C) Interstitial inflammatory infiltrate
(D) Mesangial expansion with glomerular basement membrane thickening
(E) Proximal tubular dilation with loss of brush border

---

85. A 48-year-old man comes to the physician requesting treatment for alcohol withdrawal. He reports a 30-year history of consuming 6 to 10 beers daily. He has had two citations for driving while intoxicated. He has previously experienced alcohol-associated seizures and withdrawal symptoms. His vital signs are within normal limits. Physical examination shows palmar erythema. The most appropriate pharmacotherapy in this patient most likely has which of the following mechanisms?

(A) Blockade of dopamine receptors
(B) Decreased activity of dopamine transporters
(C) Enhancement of the effect of postsynaptic γ-aminobutyric acid (GABA)
(D) Increased GABA transaminase activity
(E) Inhibition of glutamate release
(F) Inhibition of serotonin reuptake
(G) Opening of glutamate channels
(H) Stimulation of 5-hydroxytryptophan receptors

86. A 6-year-old girl is brought to the office because of two episodes of vaginal bleeding during the past 2 months. She has no history of serious illness and receives no medications. She does not appear to be in distress. She is at the 60th percentile for height, 40th percentile for weight, and 35th percentile for BMI. Vital signs are within normal limits. Physical examination discloses palpable breast buds and minimal coarse, pigmented hair on the labia. The remainder of the examination shows no abnormalities. Results of serum studies are shown:

Thyroid-stimulating hormone	2.1 mU/mL (N=0.5–5.0)
Testosterone	680 ng/dL (N=0.17–0.7)
Adrenocorticotropic hormone	18 pg/mL (N=9–52)
Estradiol	185 pg/mL (N<20)
Follicle-stimulating hormone	15 mIU/mL (N<6.7)
Luteinizing hormone	3.0 mIU/mL (N<0.2)

Which of the following is the most likely cause of this patient's condition?

(A) Central activation of neurons
(B) Ectopic prolactin secretion
(C) Exogenous sex steroid secretion
(D) Germline *GNAS* activating mutation
(E) 21-Hydroxylase deficiency

---

87. A previously healthy 64-year-old man is brought to the emergency department 3 hours after the sudden onset of severe flank pain. He has no history of similar pain or serious illness. He takes no medications. He appears to be in distress. His pulse is 100/min, and blood pressure is 168/92 mm Hg. Physical examination shows a soft abdomen with left-sided tenderness. Urinalysis shows microscopic hematuria. A CT scan of the abdomen shows a small ureteric calculus. Analgesic therapy is initiated and the pain resolves 1 hour later. The urine is then strained and a uric acid calculus is found. Which of the following processes is most likely impaired in this patient?

(A) Bile salt metabolism
(B) Cholesterol metabolism
(C) Cytochrome P450 activity
(D) Purine metabolism
(E) Urea cycle

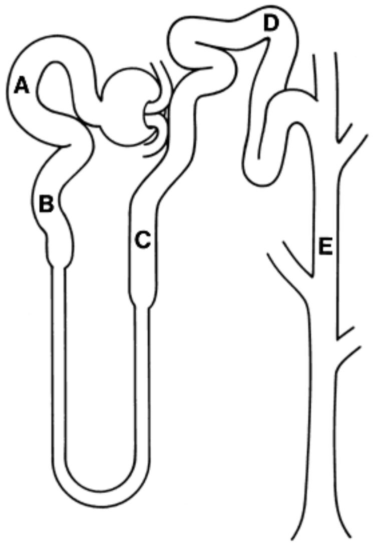

88. A 65-year-old woman has congestive heart failure for which she is treated with an appropriate drug regimen including an ACE inhibitor. Because of insufficient improvement in her symptoms of dyspnea on exertion and edema, the physician prescribes a second agent that results in hyperkalemia. This adverse effect most likely resulted from a drug that targets which of the following labeled locations in the diagram of a nephron?

(A) A
(B) B
(C) C
(D) D
(E) E

---

89. <u>**Patient Information**</u>
Age: 34 years
Gender: M, self-identified
Race/Ethnicity: unspecified
Site of Care: office

The patient presents because of a 2-year history of recurrent abdominal pain and diarrhea; he also has had an 11-kg (25-lb) weight loss during this period. He describes his stools as copious, loose, oily and foul-smelling. His bowel movements occur six to eight times daily, often immediately after meals, and are associated with bloating and cramping. Since the age of 21 years, he has had several episodes of acute pancreatitis. He has not had fevers or bloody stools. He is 178 cm (5 ft 10 in) tall and weighs 57 kg (125 lb); BMI is 18 kg/m^2. Physical examination shows a flat abdomen with hyperactive bowel sounds and diffuse mild tenderness. Digital rectal examination shows no abnormalities. Test of the stool for occult blood is negative. Treatment with which of the following enzymes is most likely to be beneficial in this patient?

(A) Amylase
(B) Lactase
(C) Lipase
(D) Protease
(E) Tryptase

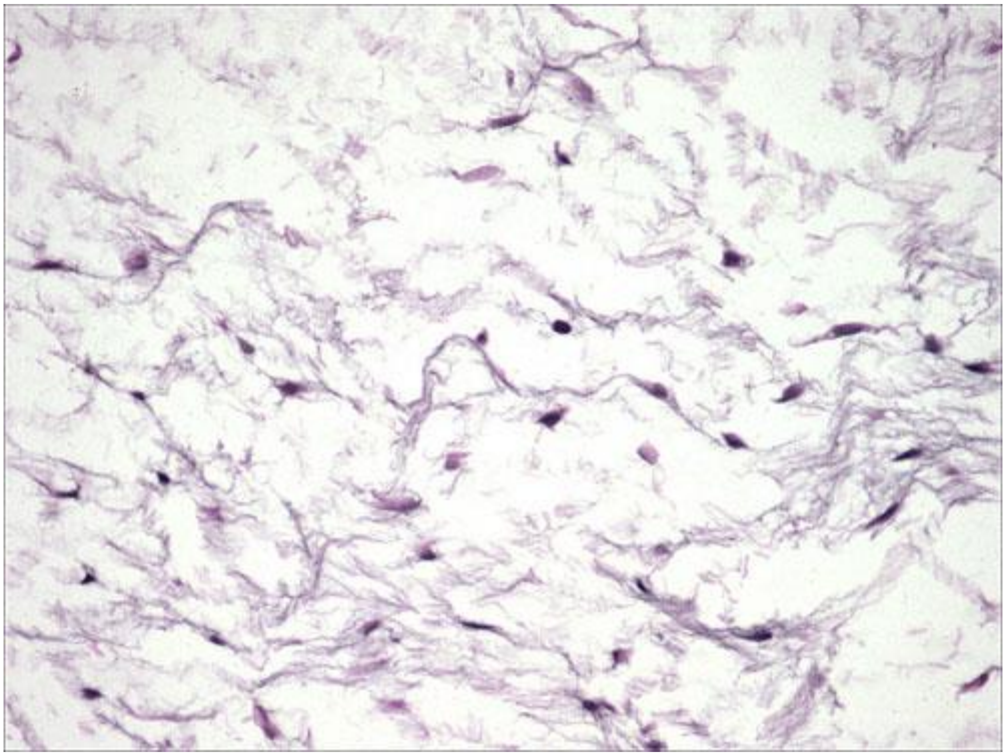

90. A previously healthy 65-year-old woman comes to the physician because of several episodes of fainting during the past 2 months. Each episode lasted several minutes. Her pulse is 82/min, respirations are 20/min, and blood pressure is 135/87 mm Hg. Cardiac examination shows S_3 and S_4. Echocardiography shows a pedunculated intracardiac mass. The lesion is resected. A photomicrograph of the resected lesion is shown. This lesion was most likely obtained from which of the following locations?

(A) Interventricular septum
(B) Left atrium
(C) Left ventricle
(D) Right atrium
(E) Right ventricle

---

91. A 4-year-old girl with type 1 diabetes mellitus is brought to the emergency department by her father because of a 4-hour episode of restlessness, sweating, and confusion that occurred during the night. Yesterday, he allowed her to eat cupcakes and cotton candy at a county fair. At her bedtime that evening, he increased her dose of subcutaneous intermediate-acting and long-acting insulin. Her symptoms began 6 hours later, then resolved spontaneously. After being informed this morning of this nighttime episode, the mother insisted the father bring the patient to the hospital. On arrival, the patient is alert. Her vital signs are within normal limits. Examination shows no abnormalities. Her fingerstick blood glucose concentration is 72 mg/dL. Urinalysis is negative for glucose and ketones. Which of the following is the most likely explanation for this patient's nighttime symptoms?

(A) Hyperglycemia caused by increased glucose consumption
(B) Hyperglycemia caused by increased glycogen metabolism
(C) Hyperglycemia caused by insufficient exogenous insulin
(D) Hypoglycemia caused by excess exogenous insulin
(E) Hypoglycemia caused by excessive renal glucose loss
(F) Hypoglycemia caused by increased glucagon secretion
(G) Nightmare disorder
(H) Sleep terror disorder

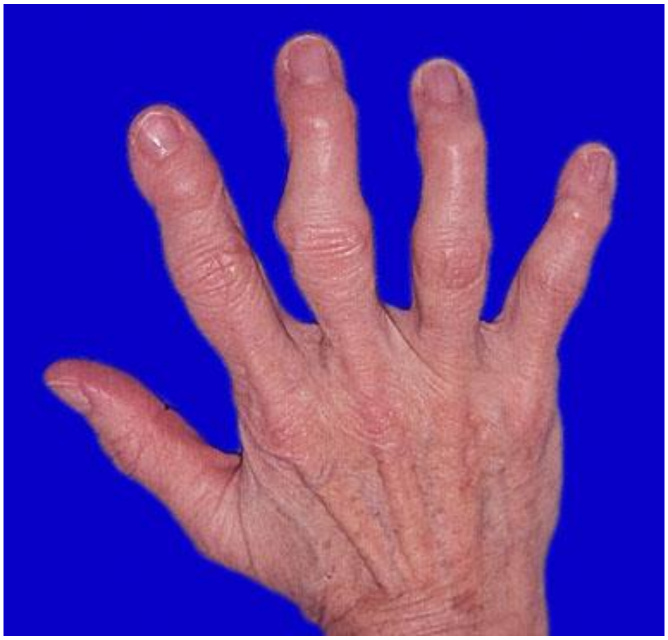

92. A 75-year-old woman comes to the physician because of a 3-year history of pain in her wrists and hands. She says that the pain has become more severe during the past 3 months. She has difficulty buttoning her coat because of the pain. Physical examination shows the findings in the photograph. Which of the following additional hand findings is most likely in this patient?

(A) Cortical thinning
(B) Degenerative changes of the cartilage
(C) Inflammation of digital tendons
(D) Neutrophilic infiltration of the synovium
(E) Thickening of the synovium with pannus

---

93. A 66-year-old man who was recently diagnosed with Parkinson disease comes to the physician for a follow-up examination. Carbidopa-levodopa therapy was initiated at the time of diagnosis. The patient tells the physician that he still has episodes during which he "freezes." He has a clumsy gait, and there is rigidity of his upper extremities and hands. An adjunct therapy designed to inhibit which of the following enzymes is most likely indicated in this patient?

(A) Aromatic L-amino acid decarboxylase
(B) Dopamine β-hydroxylase
(C) Monoamine oxidase B
(D) Phenylethanolamine *N*-methyltransferase
(E) Tyrosine hydroxylase

94. A 2-week-old male newborn is brought to the office for a well-child examination. He was delivered following an uncomplicated, spontaneous vaginal delivery at 41 weeks' gestation. The mother has no history of serious illness and did not receive prenatal care. Her only medication is a prenatal vitamin. She has consumed large amounts of vodka nightly for 10 years. Which of the following examination findings is most likely to be present in this patient?

(A) Hypospadias
(B) Limb hypoplasia
(C) Neck webbing
(D) Short palpebral fissures
(E) Spasticity

95. **Patient Information**

Age: 16 years
Gender: M, self-identified
Race/Ethnicity: Hispanic, self-identified
Site of Care: clinic

History

Reason for Visit/Chief Concern: "I haven't started puberty yet."

History of Present Illness:
• absence of pubic and facial hair
• small penis and testicle size

Past Medical History:
• no serious illness

Medications:
• none

Allergies:
• no known drug allergies

Psychosocial History:
• does not smoke cigarettes, drink alcoholic beverages, or use other substances

Physical Examination

Temp	Pulse	Resp	BP	O_2 Sat	Ht	Wt	BMI
36.0°C (96.8°F)	76/min	13/min	108/61 mm Hg	98% on RA	184 cm (6 ft) 90th %ile	90 kg (198 lb) 97th %ile	27 kg/m^2 94th %ile

• Appearance: long extremities
• Skin: no facial or axillary hair
• HEENT: PERRLA
• Chest: bilateral gynecomastia
• Pulmonary: clear to auscultation
• Cardiac: normal S_1 and S_2
• Abdominal: normoactive bowel sounds; no tenderness to palpation; no hepatosplenomegaly
• Genitourinary: small testicular and penile size; minimal pubic hair; SMR 2
• Neurologic: muscle strength 5/5 throughout

Diagnostic Studies:

Serum	
Follicle-stimulating hormone	45 mIU/mL
Luteinizing hormone	34 mIU/mL
Testosterone	45 ng/dL (N=270–950)
TSH	2.2 µU/L

Question: Which of the following is the most likely underlying cause of this patient's condition?

(A) Constitutional delay in puberty
(B) Hypoplasia of hypothalamic gonadotropin-releasing hormone neurons
(C) Inactivating mutation of the follicle-stimulating hormone-receptor
(D) Mutation in the 21-hydroxylase gene
(E) Nondisjunction of X chromosome during meiosis

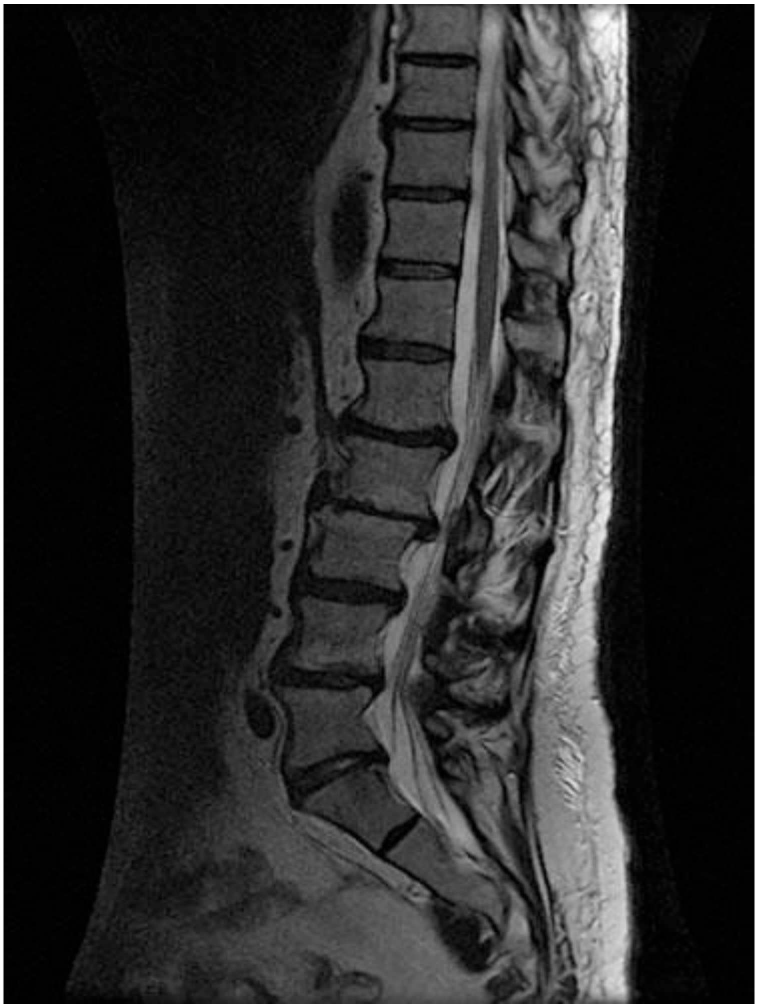

96. A 57-year-old man with chronic low back pain comes to the office for a routine health maintenance examination. The patient's last visit to the office was 2 years ago, and today he says he is "doing about the same," except for an unintentional 10-kg (22-lb) weight gain. He attributes the weight gain to inability to exercise because of his back pain, and he is now considering applying for disability benefits. He was evaluated by a back pain specialist 3 months ago and underwent an MRI of the lumbar spine at that time; however, he says he did not understand the specialist's explanation regarding the MRI results and requests further explanation. Medical history also is remarkable for hyperlipidemia, seasonal allergies, and opioid use disorder; he has not used opioids of any kind since he stopped prescription oxycodone use 5 years ago. Routine medications are atorvastatin, diclofenac, and loratadine. He develops skin flushing when taking niacin, but he has no known drug allergies. He has smoked two packs of cigarettes daily for 25 years. He previously drank two beers daily, but he has not consumed any alcoholic beverages or used any recreational drugs for the past 5 years. He is 178 cm (5 ft 10 in) tall and weighs 104 kg (230 lb); BMI is 33 kg/m^2. Vital signs are within normal limits; blood pressure is 128/70 mm Hg. While standing, the patient leans forward slightly. Range of motion on lumbar extension and flexion is decreased. The remainder of the examination discloses no abnormalities. Results of fasting serum studies are shown:

Na^+	140 mEq/L
K^+	4.7 mEq/L
Cl^-	100 mEq/L
HCO_3^-	24 mEq/L
Urea nitrogen	15 mg/dL
Creatinine	0.7 mg/dL

Cholesterol	
Total	230 mg/dL
HDL	60 mg/dL
LDL	154 mg/dL
Triglycerides	80 mg/dL

MRI of the lumbar spine is shown. Which of the following factors in this patient's history most strongly contributed to the MRI findings?

(A) Alcohol consumption
(B) Cigarette smoking
(C) Diclofenac use
(D) Loratadine use
(E) Opioid use

---

97. A 15-year-old boy is brought to the office by his parents to discuss results of a biopsy done 3 days ago for a rapidly enlarging neck mass. He first noted the mass 1 month ago. Two weeks ago, he was evaluated for an episode of prolonged epistaxis and was found to have a right ear effusion, which was treated with amoxicillin-clavulanic acid. He has no other history of serious illness and takes no medications. Height is at the 10th percentile, weight is at the 50th percentile, and BMI is at the 75th percentile. Vital signs are within normal limits. Physical examination shows a right ear effusion and a 4-cm, firm mass in the right posterior triangle of the neck. Results of a biopsy specimen show squamous epithelium with indistinct cell margins, enlarged atypical nuclei, and absent keratin formation. Which of the following infectious agents is the most likely underlying cause of this patient's current condition?

(A) Cytomegalovirus
(B) Epstein-Barr virus
(C) HIV
(D) Human herpesvirus 8

98. A 48-year-old woman with type 2 diabetes mellitus comes to the physician for a follow-up examination. Current medications are metformin and once-daily insulin. She travels frequently and works long hours. She says that her meals are usually fast food. She leads a sedentary lifestyle. She often forgets to measure her blood glucose concentration. Her last hemoglobin A_{1c} was measured as 8.4%. Which of the following is the most appropriate action by the physician to help this patient improve her diabetic control?

(A) Create an exercise regimen for the patient
(B) Explore barriers to diet adherence
(C) Increase the patient's insulin dosage
(D) Increase the patient's metformin dosage
(E) Measure the patient's blood glucose concentration
(F) Order measurement of the patient's microalbumin concentration
(G) Refer the patient to a nutritionist

99. A 74-year-old woman with a history of coronary artery disease is brought to the emergency department 30 minutes after the sudden onset of crushing chest pain. Her pulse is 120/min, and systolic blood pressure is 70 mm Hg. An ECG shows sinus rhythm with ST elevation in leads V_1 through V_3. The physician plans to administer a drug to increase the patient's blood pressure without increasing her pulse. Which of the following drugs is most appropriate for this patient?

(A) Dopamine
(B) Epinephrine
(C) Isoproterenol
(D) Norepinephrine
(E) Phenylephrine

100. A 2-month-old girl is admitted to the hospital because of severe pneumonia. She has a history of mucocutaneous candidiasis and chronic diarrhea. Her pulse is 160/min, and respirations are 40/min. Crackles are heard over both lung fields. Her leukocyte count is 5400/mm^3 (N=5000–19,500), with 86% segmented neutrophils, 6% lymphocytes, and 8% monocytes. Immunologic testing of a specimen obtained via bronchoscopy is positive for *Pneumocystis jirovecii*. Flow cytometry shows normal concentrations of natural killer cells but no T or B lymphocytes in the peripheral blood. Results of a polymerase chain reaction test of the immunoglobulin V(D)J regions from a bone marrow specimen show an absence of characteristic rearrangements. The most likely cause of these findings is an alteration of which of the following?

(A) Bruton tyrosine kinase
(B) CD40 ligand
(C) Mevalonate kinase
(D) NADPH oxidase
(E) Recombination-activating gene 1 (RAG1) and RAG2

101. A 25-year-old woman, gravida 1, para 1, comes to the office because of a 2-week history of palpitations and heat intolerance. She delivered her child 3 months ago following an uncomplicated pregnancy and delivery. She is breast-feeding. She has no history of serious illness and takes no medications. She is 163 cm (5 ft 4 in) tall and weighs 54 kg (120 lb); BMI is 21 kg/m^2. Temperature is 37°C (98.6°F), pulse is 106/min, respirations are 20/min, and blood pressure is 124/68 mm Hg. Examination shows moist palms and bilateral lid lag. No exophthalmos is noted. The thyroid gland is enlarged and nontender. No murmurs are heard on cardiac examination. Deep tendon reflexes are 3+. Serum studies show an undetectable TSH concentration, thyroxine (T_4) concentration of 20 µg/dL, and triiodothyronine (T_3) concentration of 275 ng/dL. Which of the following is the most likely mechanism of this patient's symptoms?

(A) Activation of mutations of TSH receptors
(B) Increased serum thyroglobulin concentration
(C) Ischemic injury to the hypothalamus
(D) Lymphocytic infiltration of the thyroid
(E) Presence of TSH receptor autoantibodies

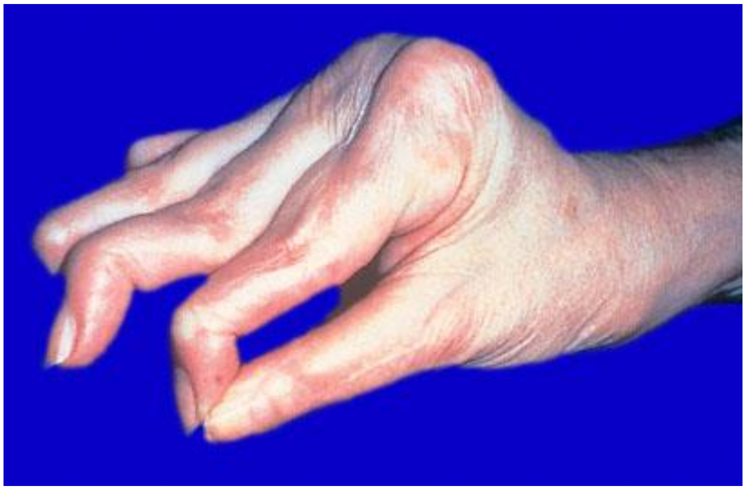

102. A 53-year-old woman comes to the office because of a 6-year history of stiffness and pain of her hands. She has difficulty buttoning her clothes because of the symptoms. She takes no medications. Physical examination shows the findings in the photograph. An abnormality of which of the following is most likely to confirm the diagnosis in this patient?

(A) Anti-citrullinated peptide antibody
(B) Antimitochondrial antibody assay
(C) Human leukocyte antigen-DQ2 antibody assay
(D) Precursor of the erythroid cell line
(E) Precursor of the thrombopoietic line

103. A 73-year-old woman comes to the emergency department because of a 6-hour history of severe abdominal pain. She has chronic atrial fibrillation and underwent placement of a prosthetic mitral valve 4 years ago. Her pulse is 120/min and irregularly irregular, and blood pressure is 80/60 mm Hg. Arteriography shows an embolus in the superior mesenteric artery lodged just beyond the patent middle colic artery. A laparotomy is done. During this procedure, which of the following segments of the abdominal viscera is most likely to appear normal?

(A) Ascending colon
(B) Distal ileum
(C) Distal jejunum
(D) Proximal ileum
(E) Proximal jejunum

104. A 2-year-old boy is brought to the physician because of failure to thrive. He also has had loose, fatty, foul-smelling stools and a cough during the past 2 weeks. He is at the 30th percentile for height and 10th percentile for weight. Physical examination shows no other abnormalities. Laboratory studies show steatorrhea and a sweat chloride concentration of 80 mmol/L. A chest x-ray shows hyperinflation. Sputum culture grows *Haemophilus influenzae* and *Staphylococcus aureus*. Secretion of which of the following substances is most likely to be decreased in this patient?

(A) Bicarbonate
(B) Gastric acid
(C) Glucagon
(D) Insulin
(E) Intrinsic factor

105. A 73-year-old man is brought to the emergency department by his family 1 hour after he walked into the left side of a door frame when leaving his bedroom and then tripped over a chair that was on his left side. Visual field testing shows left lower quadrantanopia. When tactile stimuli are presented on both the left and right sides simultaneously, the patient correctly identifies only the ones on the right. Further examination shows no motor or language deficits. Proprioception is intact. This patient most likely has a brain lesion in which of the following lobes?

(A) Left frontal
(B) Left parietal
(C) Left temporal
(D) Right frontal
(E) Right parietal
(F) Right temporal

106. A 10-year-old boy is brought to the physician because of a 3-week history of nosebleeds and easy bruisability. His older brother has had similar episodes. He is at the 30th percentile for height and weight. Physical examination shows nasal and gingival bleeding and several ecchymoses over the trunk and upper and lower extremities in various stages of healing. Laboratory studies show a platelet count of 300,000/mm^3 (N=150,000–400,000). Platelet adhesion testing shows a normal response to ristocetin, but aggregation does not occur in response to thrombin; platelet morphology is normal. Prothrombin time and activated partial thromboplastin time are within the reference ranges. A defect in which of the following is the most likely cause of the findings in this patient?

(A) Factor VII (proconvertin)
(B) Fibrinogen
(C) Glycoprotein IIb-IIIa
(D) Granule storage pool
(E) von Willebrand factor

107. A previously healthy 45-year-old woman who works as a park ranger comes to the physician because of a 1-week history of shortness of breath, even at rest. She has lived in the mountains at 10,000 feet above sea level for 2 years; the physician's office is located at sea level. Her pulse is 85/min, respirations are 18/min, and blood pressure is 125/90 mm Hg. Physical examination while sitting upright shows jugular venous distention and 2+ pedal edema. During the past 2 years, which of the following has most likely decreased in this patient?

(A) Height of P waves in lead I of the patient's ECG
(B) Height of R waves in lead V_1 of the patient's ECG
(C) Hematocrit
(D) Pulmonary vascular resistance
(E) Right ventricular diastolic compliance
(F) Right ventricular wall thickness

108. A 5-year-old girl is brought to the office by her mother because of a 6-hour history of bloody diarrhea. She is interactive and in no acute distress. Her blood pressure is 90/55 mm Hg. Abdominal examination shows normoactive bowel sounds. Stool cultures are obtained, and the patient's mother is advised to give the girl plenty of fluids. Five days later, the patient develops decreased urine output and is brought back to the office. Her blood pressure is now 135/88 mm Hg. Physical examination shows pallor. Laboratory studies show:

Hemoglobin	8.5 g/dL (N=11–15)
Hematocrit	26% (N=28%–45%)
Platelet count	45,000/mm^3 (N=150,000–400,000)
Serum creatinine	3.3 mg/dL (N=0.3–0.7)

Which of the following infectious agents is the most likely cause of these findings?

(A) *Campylobacter jejuni*
(B) *Escherichia coli*
(C) Rotavirus
(D) *Salmonella enterica* serovar *enteritidis*
(E) *Yersinia pestis*

---

109. A 78-year-old woman is admitted to the intensive care unit because of diverticulitis complicated by *Escherichia coli* sepsis. Treatment with ciprofloxacin is started. Three days later, her serum creatinine concentration has increased from 0.7 mg/dL on admission to 1.3 mg/dL. Urinalysis shows muddy brown casts. The most likely cause of the findings in this patient is ischemia of which of the following structures?

(A) Bowman capsule
(B) Glomerulus
(C) Interstitium
(D) Proximal tubule
(E) Renal vein

110. A 19-year-old man who is a college freshman comes to the office because of a 4-day history of tender, swollen glands. He also has a 6-day history of fever, malaise, and decreased appetite. His temperature is 38.7°C (101.7°F). Physical examination shows swelling of the parotid glands. Which of the following infectious agents is the most likely cause of these findings?

(A) Epstein-Barr virus
(B) Hepatitis B virus
(C) Measles virus
(D) Mumps virus
(E) Rubella virus

111. A 34-year-old woman with myasthenia gravis comes to the emergency department because of a 2-day history of increasing weakness, shortness of breath, and abdominal cramping. Current medications are prednisone and pyridostigmine. Her temperature is 37°C (98.6°F), pulse is 45/min, and respirations are 25/min and shallow. Her voice is soft and hypernasal, and she coughs weakly when swallowing water. Breath and heart sounds are normal. Pulmonary testing shows inability to generate a normal negative inspiratory force during forced inspiration. The abdomen is soft and nontender, with increased bowel sounds. Muscle strength is 4/5 diffusely, with severe, continuous, and diffuse fasciculations. Deep tendon reflexes are sluggish, but symmetric. Which of the following is the most likely cause of this patient's weakness?

(A) Aspiration pneumonia
(B) Guillain-Barré syndrome
(C) Insufficient dose of prednisone
(D) Motor neuron disease
(E) Pyridostigmine overdose

112. A 35-year-old woman comes to the office because she has had three first-trimester spontaneous abortions during the past 3 years. Physical examination shows no abnormalities. Laboratory studies show no endocrine abnormalities. Chromosomal analysis shows a paracentric inversion of the long arm of chromosome 1. Which of the following best describes this patient's risk for early spontaneous abortions and a liveborn child with aneuploidy?

	Risk for Early Spontaneous Abortions	Risk for Liveborn Child With Aneuploidy
(A)	High	high
(B)	High	low
(C)	Low	high
(D)	Low	low

---

113. A 25-year-old man comes to the office because of a 4-hour history of irritability, restlessness, tremor, and palpitations. He is a known user of amphetamines. His pulse is 120/min, respirations are 25/min, and blood pressure is 150/100 mm Hg. Physical examination shows no abnormalities. The most likely cause of this patient's symptoms is sympathomimetic activity arising from which of the following?

(A) Decreased intracellular metabolism of biogenic amines
(B) Decreased monoamine oxidase activity
(C) Decreased presynaptic receptor activation
(D) Increased intracellular metabolism of biogenic amines
(E) Increased presynaptic receptor activation
(F) Increased release of biogenic amines

114. A 14-year-old boy is brought to the office by his mother because of a 1-week history of seizures and difficulty walking. Physical examination shows decreased sensation over the hands and feet and generalized weakness. He walks with an ataxic gait. A muscle biopsy specimen shows coarsely granular fibers that stain red with Gomori trichrome stain. This patient most likely has a genetic defect that most directly affects the synthesis of which of the following?

(A) ATP
(B) Creatine phosphate
(C) Glycogen
(D) NADH
(E) Pyruvate

115. A 72-year-old man is brought to the office because of a 6-month history of increasing fatigue and a 1-month history of numbness of his feet. Physical examination shows marked pallor. Proprioception and sensation to vibration are decreased in the lower extremities. Laboratory studies show:

Hemoglobin	7.6 g/dL
Mean corpuscular volume	117 μm^3
Reticulocyte count	0%
Serum vitamin B_{12} (cobalamin)	23.6 pg/mL (N=200–900)

Histopathology of a gastric mucosal biopsy specimen shows atrophic gastritis with extensive lymphocyte infiltration. Serologic studies will most likely show autoantibodies to which of the following labeled cell types in the photomicrograph of normal mucosa or to one of its secretory products?

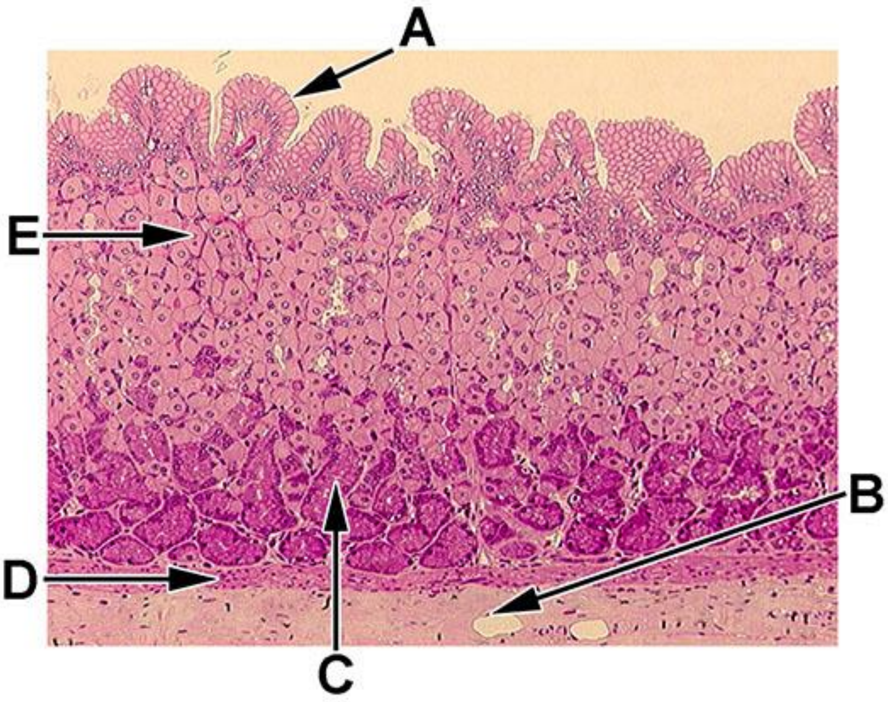

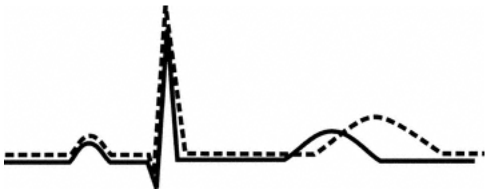

116. A 66-year-old woman comes to the office for a follow-up examination 1 month after being diagnosed with paroxysmal atrial fibrillation. Appropriate pharmacotherapy was initiated at that time, and normal sinus rhythm was restored. Her pulse is 76/min, and blood pressure is 132/86 mm Hg. Physical examination shows no abnormalities. The ECGs shown represent before (solid line) and after (dashed line) administration of the antiarrhythmic drug. Which of the following drugs was most likely prescribed?

(A) Amiodarone
(B) Digoxin
(C) Mexiletine
(D) Phenytoin
(E) Verapamil

---

117. A 4-year-old boy develops fever 3 weeks after being admitted to the hospital for induction chemotherapy for treatment of acute lymphoblastic leukemia. Chemotherapy medications are L-asparaginase, dexamethasone, doxorubicin, and vincristine. His temperature is 38.2°C (100.8°F), pulse is 122/min, and respirations are 24/min. Physical examination shows pallor, alopecia, and ulcerations over the gums. A central venous catheter with entry site in the right upper chest is present but has no surrounding erythema. A blood culture grows gram-negative rods after 36 hours. Which of the following underlying mechanisms is the most likely cause of this patient's susceptibility to infection?

(A) Deficiency of terminal complement
(B) Hypogammaglobulinemia
(C) Impaired T-lymphocyte function
(D) Inhibition of tumor necrosis factor α function
(E) Neutropenia

118. A 6-year-old boy with acute lymphoblastic leukemia is brought to the office for a follow-up examination. He is receiving high-dose methotrexate therapy. A drug is added to the patient's medication regimen to decrease the toxicity of this therapy to normal cells. The beneficial effect of this new drug on normal cells is most likely achieved by bypassing the cellular requirement for which of the following enzymes?

(A) Dihydrofolate reductase
(B) Methionine synthase
(C) Pyruvate decarboxylase
(D) Thiamine pyrophosphate
(E) Thymidylate synthase

119. A screening test for breast cancer is administered to 1000 women with biopsy-proven breast cancer and to 1000 women without breast cancer. The test results are positive for 250 of the subjects with breast cancer and 100 of the subjects without breast cancer. The screening test is now to be used on a population of 100,000 women with a known prevalence rate of breast cancer of 80 per 100,000. Which of the following is the expected number of false-positives?

(A) 20
(B) 80
(C) 8993
(D) 9992
(E) 10,012

ANSWER FORM FOR USMLE STEP 1 SAMPLE TEST QUESTIONS

Block 1 (Questions 1–40)

1.	___	11.	___	21.	___	31.	___
2.	___	12.	___	22.	___	32.	___
3.	___	13.	___	23.	___	33.	___
4.	___	14.	___	24.	___	34.	___
5.	___	15.	___	25.	___	35.	___
6.	___	16.	___	26.	___	36.	___
7.	___	17.	___	27.	___	37.	___
8.	___	18.	___	28.	___	38.	___
9.	___	19.	___	29.	___	39.	___
10.	___	20.	___	30.	___	40.	___

Block 2 (Questions 41–80)

41.	___	51.	___	61.	___	71.	___
42.	___	52.	___	62.	___	72.	___
43.	___	53.	___	63.	___	73.	___
44.	___	54.	___	64.	___	74.	___
45.	___	55.	___	65.	___	75.	___
46.	___	56.	___	66.	___	76.	___
47.	___	57.	___	67.	___	77.	___
48.	___	58.	___	68.	___	78.	___
49.	___	59.	___	69.	___	79.	___
50.	___	60.	___	70.	___	80.	___

Block 3 (Questions 81–119)

81.	___	91.	___	101.	___	111.	___
82.	___	92.	___	102.	___	112.	___
83.	___	93.	___	103.	___	113.	___
84.	___	94.	___	104.	___	114.	___
85.	___	95.	___	105.	___	115.	___
86.	___	96.	___	106.	___	116.	___
87.	___	97.	___	107.	___	117.	___
88.	___	98.	___	108.	___	118.	___
89.	___	99.	___	109.	___	119.	___
90.	___	100.	___	110.	___		

ANSWER KEY FOR USMLE STEP 1 SAMPLE TEST QUESTIONS

Block 1 (Questions 1–40)

1. A
2. D
3. B
4. A
5. D
6. E
7. C
8. A
9. D
10. A
11. C
12. B
13. F
14. C
15. B
16. B
17. E
18. D
19. B
20. D
21. C
22. A
23. D
24. B
25. B
26. D
27. C
28. E
29. B
30. D
31. D
32. E
33. D
34. A
35. A
36. C
37. D
38. E
39. B
40. D

Block 2 (Questions 41–80)

41. B
42. A
43. C
44. C
45. C
46. B
47. A
48. A
49. B
50. D
51. D
52. B
53. A
54. B
55. E
56. D
57. C
58. E
59. E
60. E
61. D
62. A
63. C
64. B
65. A
66. C
67. C
68. C
69. C
70. D
71. A
72. B
73. A
74. A
75. E
76. C
77. D
78. C
79. A
80. E

Block 3 (Questions 81–119)

81. D
82. D
83. B
84. C
85. C
86. A
87. D
88. E
89. C
90. B
91. D
92. B
93. C
94. D
95. E
96. B
97. B
98. B
99. E
100. E
101. D
102. A
103. E
104. A
105. E
106. C
107. E
108. B
109. D
110. D
111. E
112. B
113. F
114. A
115. E
116. A
117. E
118. A
119. D

SAMPLE TEST QUESTIONS

Step 2 Clinical Knowledge (CK)

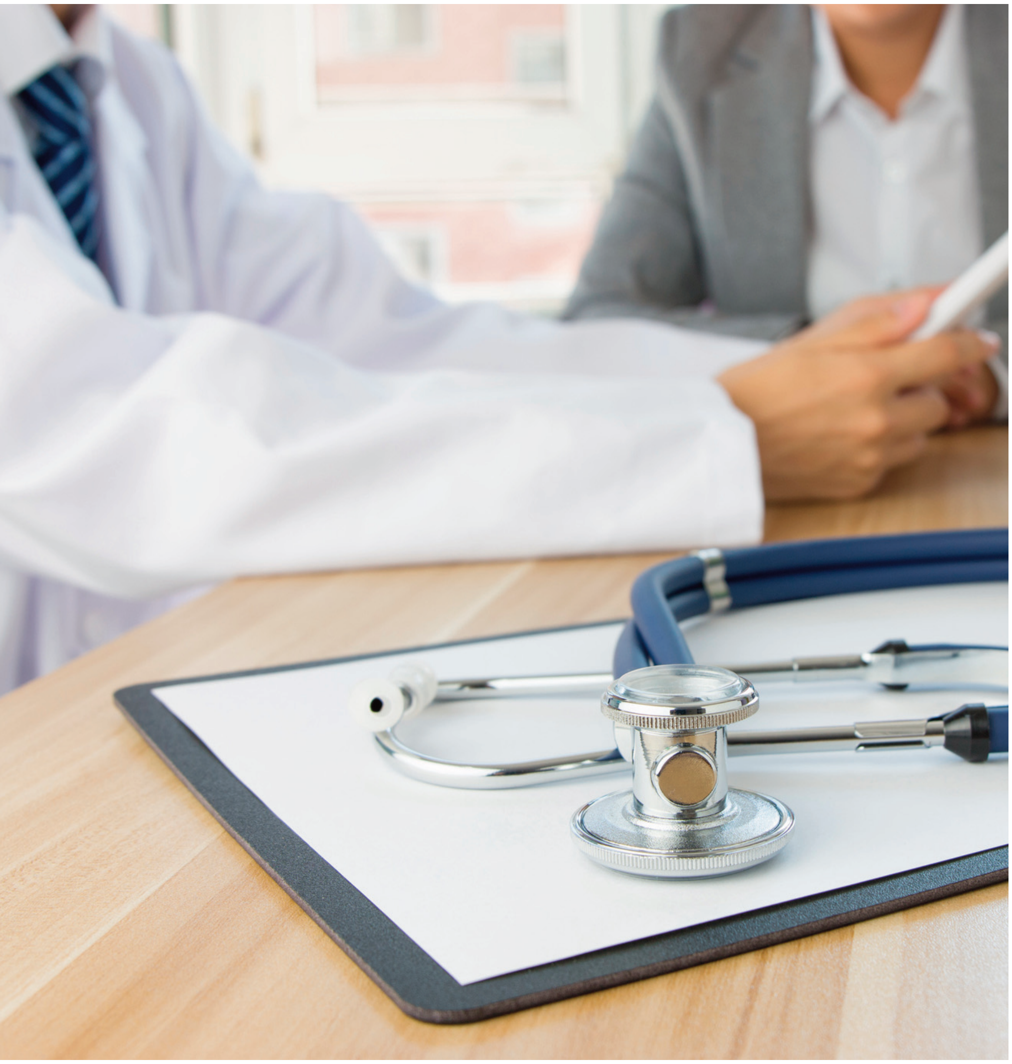

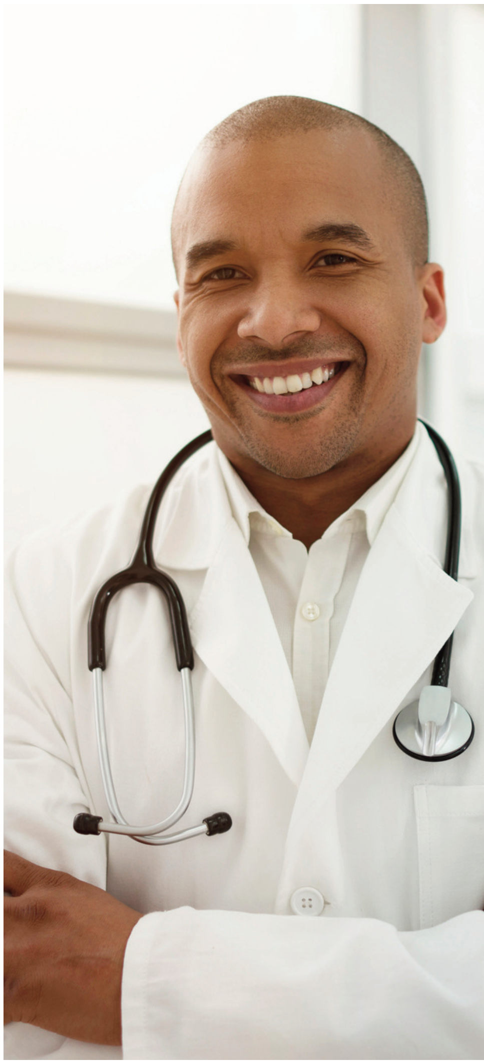

A Joint Program of the Federation of State Medical Boards of the United States, Inc., and National Board of Medical Examiners

This booklet was updated July 2023.
For Public Release

Copyright © 2023 by the Federation of State Medical Boards of the United States, Inc. (FSMB), and the National Board of Medical Examiners® (NBME®). All rights reserved. Printed in the United States of America. The United States Medical Licensing Examination® (USMLE®) is a joint program of the FSMB and NBME.

CONTENTS

USMLE Step 2 CK Test Question Formats 3

Introduction to USMLE Step 2 CK Sample Test Questions 9

USMLE Laboratory Values 10

USMLE Step 2 CK Sample Test Questions.. 13

Block 1, Items 1–40 13

Block 2, Items 41–80 29

Block 3, Items 81–120 47

Answer Form for USMLE Step 2 CK Sample Test Questions....................................... 63

Answer Key for USMLE Step 2 CK Sample Test Questions... 64

The following are strategies for answering one-best-answer items:

- Read each question carefully. It is important to understand what is being asked.
- Try to generate an answer and then look for it in the response option list.
- Alternatively, read each response option carefully, eliminating those that are clearly incorrect.
- Of the remaining response options, select the one that is most correct.
- If unsure about an answer, it is better to guess since unanswered questions are automatically counted as wrong answers.

Patient Scenario Formats

Patient scenarios for any Single Item or Sequential Item Set may be provided in either Vignette (paragraph) format, or in Chart/Tabular format. Test items using the chart/tabular format are designed to resemble patient charts but are not intended to be an exact representation of a patient chart. Questions written in Chart/Tabular format will contain relevant patient information in list form, organized in clearly marked sections for ease of review. Familiar medical abbreviations may be used within Chart/Tabular format questions.

Single-Item Questions

A single patient-centered vignette is associated with one question followed by four or more response options. The response options are lettered (ie, A, B, C, D, E). A portion of the questions involves interpretation of graphic or pictorial materials. You are required to select the best answer to the question. Other options may be partially correct, but there is only ONE BEST answer. This is the traditional, most frequently used multiple-choice question format on the examination.

Example Question 1

A 32-year-old woman with type 1 diabetes mellitus has had progressive renal failure over the past 2 years. She has not yet started dialysis. Examination shows no abnormalities. Her hemoglobin concentration is 9 g/dL, hematocrit is 28%, and mean corpuscular volume is 94 μm^3. A blood smear shows normochromic, normocytic cells. Which of the following is the most likely cause?

(A) Acute blood loss
(B) Chronic lymphocytic leukemia
(C) Erythrocyte enzyme deficiency
(D) Erythropoietin deficiency
(E) Immunohemolysis
(F) Microangiopathic hemolysis
(G) Polycythemia vera
(H) Sickle cell disease
(I) Sideroblastic anemia
(J) β-Thalassemia trait

(Answer: D)

Example Question 2

Patient Information
Age: 18 years
Gender: F, self-identified
Ethnicity: unspecified
Site of Care: emergency department

History
Chief Complaint: "My roommate took a lot of pills and said she wanted to die."
History of Present Illness:
- patient brought by ambulance 2 hours after a suspected acetaminophen overdose
- roommate had returned to their dorm and found the patient distraught; patient was holding an empty bottle of acetaminophen and said, "I just want to die."
- roommate estimates patient consumed 20 to 30 tablets of 500-mg acetaminophen

Past Medical History:
- generalized anxiety disorder
- major depressive disorder

Medications:
- citalopram

Allergies:
- no known drug allergies

Family History:
- noncontributory

Psychosocial History:
- college freshman; parents live 8 hours away

Physical Examination

Temp	Pulse	Resp	BP	O_2 Sat	Ht	Wt	BMI
36.8°C (98.2°F)	89/min	16/min	108/59 mm Hg	99% on RA	170 cm (5 ft 7 in)	63 kg (140 lb)	22 kg/m^2

- Appearance: awake and alert but distraught and fearful; patient says, "This was totally stupid."
- Pulmonary: clear lung fields
- Cardiac: regular rhythm; S_1 and S_2, with an early systolic murmur
- Abdominal: soft and nontender; liver and spleen cannot be palpated
- Neurologic: no abnormalities; fully oriented

Question: In addition to obtaining serum acetaminophen concentration, which of the following is the most appropriate next step in management?

(A) Administer N-acetylcysteine
(B) Arrange hemodialysis
(C) Gastric lavage
(D) Initiate sodium bicarbonate infusion

(Answer: A)

Sequential Item Sets

A single patient-centered vignette may be associated with two or three consecutive questions about the information presented. Each question is associated with the initial patient vignette but is testing a different point. You are required to select the ONE BEST answer to each question. These questions are designed to be answered in sequential order. You must click "Proceed to Next Item" to view the next item in the set; once you click on this button, the next question will be displayed, and you will not be able to change the answer to the previous question.

Example Sequential Question

A 35-year-old woman is brought to the emergency department because of worsening pain and swelling of her right knee for the past 2 days. She has been taking acetaminophen for the knee pain during the past 2 days, but the pain is worse today. She has not had any trauma to the knee or any previous problems with her joints. She is otherwise healthy and she currently takes an oral contraceptive. She is sexually active and has a 10-year-old son who lives with her. She is a receptionist at a local hotel and she tells you she must stand often while working. She is 160 cm (5 ft 3 in) tall and weighs 52 kg (115 lb); BMI is 20 kg/m^2. Temperature is 37.9°C (98.9°F). The right knee is erythematous, swollen, and tender; there is pain on movement. No other joints are affected. X-ray of the knee shows an effusion but no structural abnormalities of the joint.

Which of the following is the most appropriate next step in diagnosis?

(A) Arthrocentesis of the knee
(B) Blood cultures
(C) Complete blood count
(D) MRI of the knee
(E) Urine cultures

(Answer: A)

Arthrocentesis is done. The synovial fluid is cloudy. Gram stain is negative. Analysis of the synovial fluid shows a leukocyte count of 120,000/mm^3 and 90% neutrophils. Which of the following is the most appropriate additional test on the synovial fluid?

(A) Culture for bacteria
(B) Glucose measurement
(C) Polarized light microscopy
(D) Protein level

(Answer: A)

Abstract Set Format

The abstract item format includes a summary of an experiment or clinical investigation presented in a manner commonly encountered by a physician, eg, as an abstract that accompanies a research report in a medical journal. Examinees must interpret the abstract in order to answer questions on various topics, including

- Decisions about care of an individual patient
- Biostatistics/epidemiology
- Pharmacology/therapeutics
- Use of diagnostic studies

Example Abstract Set

Question

In children with type 1 diabetes mellitus, what factors are associated with increased risk for microalbuminuria and macroalbuminuria?

Methods

Design: Inception cohort followed for a mean of 9.8 years.
Setting: St. Bartholomew's Oxford Diabetes Register, Oxford, England, UK.
Patients: 527 children < 16 years of age (mean age 9 years) who were diagnosed with type 1 diabetes mellitus and included in the Diabetes Register from 1986 to 1997 (90% follow-up).
Prognostic factors: Mean glycated hemoglobin concentration (HbA_{1c}), female sex, mean blood pressure, history of smoking, and age at diagnosis.
Outcomes: Microalbuminuria (albumin-to-creatinine ratio 3.5 to 35 mg/mmol in boys and 4.0 to 47 mg/mmol in girls, in two annual, consecutive, early morning urine samples), and macroalbuminuria (albumin-to-creatinine ratio > 35 mg/mmol in boys and > 47 mg/mmol in girls).

Main results

135 patients (26%) developed microalbuminuria, with a cumulative prevalence of 25.7% (95% CI 21 to 30) after 10 years and 51% (CI 41 to 61) after 19 years. Of the 135 patients with microalbuminuria, there was a cumulative prevalence of regression to the normoalbuminuric range of 52% (CI 42 to 62) after 4.9 years from the onset of microalbuminuria. 18 patients (3%) developed macroalbuminuria, with a cumulative prevalence of 14% (CI 13 to 15) after 3.2 years from onset of microalbuminuria. HbA_{1c} and female sex were associated with increased risk for microalbuminuria (Table). HbA_{1c} and persistent and intermittent microalbuminuria were associated with increased risk for macroalbuminuria (Table).

Conclusions

In children with type 1 diabetes mellitus, poor glycemic control and female sex were associated with development of microalbuminuria. About half of patients with microalbuminuria regressed to normoalbuminuria. Poor glycemic control and persistent or intermittent microalbuminuria were associated with development of macroalbuminuria.

Sources of funding: Diabetes UK; Juvenile Diabetes Research Foundation; Wellcome Trust; NIHR Cambridge Biomedical Research Centre.

Structured abstract based on: Amin R, Widmer B, Prevost AT, et al. **Risk of microalbuminuria and progression to macroalbuminuria in a cohort with childhood onset type 1 diabetes: prospective observational study.** BMJ. 2008;336:697-701. 18349042

Abstract continues on next page.

Factors associated with renal outcomes in children with type 1 diabetes mellitus*

Outcomes	Prognostic factors	At a mean 9.8 years of follow-up
		**Hazard ratio (95% CI)**
Microalbuminuria	HbA_{1c} (per % increase)	1.4 (1.3 to 1.5)
	Female sex	1.4 (1.02 to 2.1)
	Diastolic blood pressure	1.0 (1.0 to 1.0)
	Systolic blood pressure	1.0 (1.0 to 1.0)
	History of smoking	1.3 (0.9 to 2.0)
	Younger age at diagnosis	1.0 (1.0 to 1.1)
Macroalbuminuria	HbA_{1c} (per % increase)	1.4 (1.2 to 1.8)
	Persistent microalbuminuria†	28 (8.0 to 96)
	Intermittent microalbuminuria‡	8.8 (2.4 to 31)
	Female sex	1.3 (0.5 to 3.3)
	Diastolic blood pressure	1.1 (0.9 to 1.1)
	Systolic blood pressure	1.1 (0.9 to 1.1)
	History of smoking	1.3 (0.4 to 4.1)
	Younger age at diagnosis	1.0 (1.0 to 1.1)

*HbA_{1c} = glycated haemoglobin.
†Presence of microalbuminuria at every annual visit after first detection.
‡Positive microalbuminuria followed by regression to normoalbuminuria, then recurrence to microalbuminuria at a later date.

A 12-year-old girl is brought to the office for a follow-up examination 6 months after being diagnosed with type 1 diabetes mellitus. The patient feels well. She says she smokes cigarettes occasionally when with friends. She is not sexually active. She is at the 60th percentile for BMI. Her temperature is 37.0°C (98.6°F), pulse is 80/min, respirations are 16/min, and blood pressure is 108/72 mm Hg. Physical examination shows no other abnormalities. Results of urinalysis are within the reference ranges. Her father, who has type 1 diabetes mellitus and chronic renal disease, asks what markers will be followed to determine his daughter's risk for developing chronic renal disease. Which of the following patient characteristics most increases her risk for developing microalbuminuria over the next 10 years?

(A) Age at diagnosis
(B) BMI
(C) Diastolic blood pressure
(D) Gender
(E) Smoking history

Answer: D

Which of the following current or potential future findings is most likely to increase the patient’s risk for developing macroalbuminuria?

(A) Age at diagnosis
(B) Cigarette use
(C) Elevated systolic blood pressure
(D) Gender
(E) Poor glycemic control

Answer: E

Abstract set continues on next page.

One year later, the patient's urinalysis shows microalbuminuria for the first time. In a population of children of a similar age who have type 1 diabetes mellitus and had microalbuminuria detected for the first time, which of the following outcomes is most likely?

(A) Hemoglobin A_{1c} will normalize within 10 years
(B) Hypertension will be diagnosed within 2 years
(C) Macroalbuminuria will be detected within 1 year
(D) Microalbuminuria will resolve within 5 years

Answer: D

NOTE: When additional question formats are added to the examination, notice will be provided on the USMLE website (www.usmle.org). You must monitor the website to stay informed about the types of questions that occur in the examination, and you must practice with the downloadable sample test questions available on the USMLE website in order to be fully prepared for the examination.

INTRODUCTION TO USMLE STEP 2 CK SAMPLE TEST QUESTIONS

The following pages include 120 sample test questions. Most of these practice questions are the same as those on the USMLE website.

ONLINE:

Please note that reviewing the sample questions as they appear on pages 13–62 is **not a substitute for practicing on the website**.

1. You should run the tutorial and items in the interactive testing experience on the USMLE website well before your test date.
2. The items in the interactive testing experience include additional questions and formats that do not appear in this booklet, such as items with associated audio.

CONTENT:

You should become familiar with all test question formats that will be used in the actual examination.

Although the sample questions exemplify content on the examination, they may not reflect the content coverage on individual examinations.

In the actual examination, questions will be presented in random order. The questions will be presented one at a time in a format designed for easy on-screen reading, including a Normal Laboratory Values button (Table included here on pages 10–12). Photographs, charts, and x-rays in this booklet are not of the same quality as the pictorials used in the actual examination. In addition, you will be able to adjust the brightness and contrast of the computer screen.

TIMING:

To take the following sample test questions as they would be timed in the actual examination, you should allow a maximum of one hour for each block, for a total of three hours. Please be aware that most examinees perceive the time pressure to be greater during an actual examination.

An answer form for recording answers for this practice is provided on page 63. An answer key is provided on page 64. In the actual examination, answers will be selected on the screen; no answer form will be provided.

	Reference Range	SI Reference Intervals
**SERUM**		
General Chemistry:		
Electrolytes		
Sodium (Na^+)	136–146 mEq/L	136–146 mmol/L
Potassium (K^+)	3.5–5.0 mEq/L	3.5–5.0 mmol/L
Chloride (Cl^-)	95–105 mEq/L	95–105 mmol/L
Bicarbonate (HCO_3^-)	22–28 mEq/L	22–28 mmol/L
Urea nitrogen	7–18 mg/dL	2.5–6.4 mmol/L
Creatinine	0.6–1.2 mg/dL	53–106 µmol/L
Glucose	Fasting: 70–100 mg/dL	3.8–5.6 mmol/L
	Random, non-fasting: <140 mg/dL	<7.77 mmol/L
Calcium	8.4–10.2 mg/dL	2.1–2.6 mmol/L
Magnesium (Mg^{2+})	1.5–2.0 mg/dL	0.75–1.0 mmol/L
Phosphorus (inorganic)	3.0–4.5 mg/dL	1.0–1.5 mmol/L
Hepatic:		
Alanine aminotransferase (ALT)	10–40 U/L	10–40 U/L
Aspartate aminotransferase (AST)	12–38 U/L	12–38 U/L
Alkaline phosphatase	25–100 U/L	25–100 U/L
Amylase	25–125 U/L	25–125 U/L
Bilirubin, total // direct	0.1–1.0 mg/dL // 0.0–0.3 mg/dL	2–17 µmol/L // 0–5 µmol/L
Proteins, total	6.0–7.8 g/dL	60–78 g/L
Albumin	3.5–5.5 g/dL	35–55 g/L
Globulin	2.3–3.5 g/dL	23–35 g/L
Lipids:		
Cholesterol		
Total	Normal: <200 mg/dL	<5.2 mmol/L
	High: >240 mg/dL	>6.2 mmol/L
HDL	40–60 mg/dL	1.0–1.6 mmol/L
LDL	<160 mg/dL	<4.2 mmol/L
Triglycerides	Normal: <150 mg/dL	<1.70 mmol/L
	Borderline: 151–199 mg/dL	1.71–2.25 mmol/L
Iron Studies:		
Ferritin	Male: 20–250 ng/mL	20–250 µg/L
	Female: 10–120 ng/mL	10–120 µg/L
Iron	Male: 65–175 µg/dL	11.6–31.3 µmol/L
	Female: 50–170 µg/dL	9.0–30.4 µmol/L
Total iron-binding capacity	250–400 µg/dL	44.8–71.6 µmol/L
Transferrin	200–360 mg/dL	2.0–3.6 g/L

Continued on Next Page

	Reference Range	SI Reference Intervals
Endocrine:		
Follicle-stimulating hormone	Male: 4–25 mIU/mL	4–25 IU/L
	Female: premenopause 4–30 mIU/mL	4–30 IU/L
	midcycle peak 10–90 mIU/mL	10–90 IU/L
	postmenopause 40–250 mIU/mL	40–250 IU/L
Luteinizing hormone	Male: 6–23 mIU/mL	6–23 IU/L
	Female: follicular phase 5–30 mIU/mL	5–30 IU/L
	midcycle 75–150 mIU/mL	75–150 IU/L
	postmenopause 30–200 mIU/mL	30–200 IU/L
Growth hormone - arginine stimulation	Fasting: <5 ng/mL	<5 µg/L
	Provocative stimuli: >7 ng/mL	>7 µg/L
Prolactin (hPRL)	Male: <17 ng/mL	<17 µg/L
	Female: <25 ng/mL	<25 µg/L
Cortisol	0800 h: 5–23 µg/dL	138–635 nmol/L
	1600 h: 3–15 µg/dL	82–413 nmol/L
	2000 h: <50% of 0800 h	Fraction of 0800 h: <0.50
TSH	0.4–4.0 µU/mL	0.4–4.0 mIU/L
Triiodothyronine (T_3) (RIA)	100–200 ng/dL	1.5–3.1 nmol/L
Triiodothyronine (T_3) resin uptake	25%–35%	0.25–0.35
Thyroxine (T_4)	5–12 µg/dL	64–155 nmol/L
Free T_4	0.9–1.7 ng/dL	12.0–21.9 pmol/L
Thyroidal iodine (^{123}I) uptake	8%–30% of administered dose/24 h	0.08–0.30/24 h
Intact PTH	10–60 pg/mL	10–60 ng/L
17-Hydroxycorticosteroids	Male: 3.0–10.0 mg/24 h	8.2–27.6 µmol/24 h
	Female: 2.0–8.0 mg/24 h	5.5–22.0 µmol/24 h
17-Ketosteroids, total	Male: 8–20 mg/24 h	28–70 µmol/24 h
	Female: 6–15 mg/24 h	21–52 µmol/24 h
Immunoglobulins:		
IgA	76–390 mg/dL	0.76–3.90 g/L
IgE	0–380 IU/mL	0–380 kIU/L
IgG	650–1500 mg/dL	6.5–15.0 g/L
IgM	50–300 mg/dL	0.5–3.0 g/L
Other, serum:		
Creatinine clearance	Male: 97–137 mL/min	97–137 mL/min
	Female: 88–128 mL/min	88–128 mL/min
Creatine kinase	Male: 25–90 U/L	25–90 U/L
	Female: 10–70 U/L	10–70 U/L
Lactate dehydrogenase	45–200 U/L	45–200 U/L
Osmolality	275–295 mOsmol/kg H_2O	275–295 mOsmol/kg H_2O
Uric acid	3.0–8.2 mg/dL	0.18–0.48 mmol/L
**GASES, ARTERIAL BLOOD (ROOM AIR)**		
P_{O_2}	75–105 mm Hg	10.0–14.0 kPa
P_{CO_2}	33–45 mm Hg	4.4–5.9 kPa
pH	7.35–7.45	$[H^+]$ 36–44 nmol/L
**CEREBROSPINAL FLUID**		
Cell count	0–5/mm^3	0–5 × 10^6/L
Chloride	118–132 mEq/L	118–132 mmol/L
Gamma globulin	3%–12% total proteins	0.03–0.12
Glucose	40–70 mg/dL	2.2–3.9 mmol/L
Pressure	70–180 mm H_2O	70–180 mm H_2O
Proteins, total	<40 mg/dL	<0.40 g/L

Continued on Next Page

	Reference Range	SI Reference Intervals
**HEMATOLOGIC**		
Complete Blood Count:		
Hematocrit	Male: 41%–53%	0.41–0.53
	Female: 36%–46%	0.36–0.46
Hemoglobin, blood	Male: 13.5–17.5 g/dL	135–175 g/L
	Female: 12.0–16.0 g/dL	120–160 g/L
Mean corpuscular hemoglobin (MCH)	25–35 pg/cell	0.39–0.54 fmol/cell
Mean corpuscular hemoglobin conc. (MCHC)	31%–36% Hb/cell	4.8–5.6 mmol Hb/L
Mean corpuscular volume (MCV)	80–100 μm^3	80–100 fL
Volume		
Plasma	Male: 25–43 mL/kg	0.025–0.043 L/kg
	Female: 28–45 mL/kg	0.028–0.045 L/kg
Red cell	Male: 20–36 mL/kg	0.020–0.036 L/kg
	Female: 19–31 mL/kg	0.019–0.031 L/kg
Leukocyte count (WBC)	4500–11,000/mm^3	4.5–11.0 × 10^9/L
Neutrophils, segmented	54%–62%	0.54–0.62
Neutrophils, bands	3%–5%	0.03–0.05
Lymphocytes	25%–33%	0.25–0.33
Monocytes	3%–7%	0.03–0.07
Eosinophils	1%–3%	0.01–0.03
Basophils	0%–0.75%	0.00–0.0075
Platelet count	150,000–400,000/mm^3	150–400 × 10^9/L
Coagulation:		
Partial thromboplastin time (PTT) (activated)	25–40 seconds	25–40 seconds
Prothrombin time (PT)	11–15 seconds	11–15 seconds
D-Dimer	≤250 ng/mL	≤1.4 nmol/L
Other, Hematologic:		
Reticulocyte count	0.5%–1.5%	0.005–0.015
Erythrocyte count (RBC)	Male: 4.3–5.9 million/mm^3	4.3–5.9 × 10^{12}/L
	Female: 3.5–5.5 million/mm^3	3.5–5.5 × 10^{12}/L
Erythrocyte sedimentation rate (Westergren)	Male: 0–15 mm/h	0–15 mm/h
	Female: 0–20 mm/h	0–20 mm/h
CD4+ T-lymphocyte count	≥500/mm^3	≥0.5 × 10^9/L
Troponin I	≤0.04 ng/mL	≤0.04 µg/L
Endocrine:		
Hemoglobin A_{1c}	≤6%	≤42 mmol/mol
**URINE**		
Calcium	100–300 mg/24 h	2.5–7.5 mmol/24 h
Osmolality	50–1200 mOsmol/kg H_2O	50–1200 mOsmol/kg H_2O
Oxalate	8–40 µg/mL	90–445 µmol/L
Proteins, total	<150 mg/24 h	<0.15 g/24 h
**BODY MASS INDEX (BMI)**	Adult: 19–25 kg/m^2	

BLOCK 1, ITEMS 1–40

1. A 21-year-old man comes to student health services because of a 6-month history of increasingly frequent episodes of moderate chest pain. The first episode occurred while he was sitting in traffic and feeling stressed because he was late for a college class. At that time, he had the sudden onset of moderate chest pain, a rapid heartbeat, sweating, and nausea. He says he felt as though he were going to die. The episode lasted approximately 10 minutes. He had a similar episode 1 month later while on a date; the symptoms were so severe that he abruptly ended the date. During the past 3 weeks, he has experienced two to three episodes weekly. He says he fears having an episode while in public or on a date, so he has decreased his participation in social activities and the amount of time he spends outside of his apartment. He has no history of serious illness and takes no medications. He does not drink alcohol or use other substances. Vital signs are within normal limits. Physical examination discloses no abnormalities. On mental status examination, he has an anxious mood and full range of affect. Which of the following is the most likely diagnosis?

   (A) Agoraphobia
   (B) Generalized anxiety disorder
   (C) Illness anxiety disorder (hypochondriasis)
   (D) Social anxiety disorder (social phobia)
   (E) Somatic symptom disorder

2. A 35-year-old man is brought to the emergency department by a friend 30 minutes after the sudden onset of right-sided weakness and difficulty speaking. The symptoms began while he was lifting weights at the gym. He has not had headache or changes in vision. He has no history of serious illness and takes no medications. He does not smoke cigarettes or drink alcohol. He exercises regularly. His speech is incoherent, but he can understand what others are saying. Vital signs are within normal limits. No bruits are heard over the carotid arteries. Cardiopulmonary examination shows no abnormalities. There is swelling and mild tenderness of the right lower extremity. No cords are palpated. Neurologic examination shows a right facial droop and right upper extremity weakness. An MRI of the brain shows an acute stroke in the left middle cerebral artery territory. Carotid ultrasonography shows no abnormalities. Which of the following is the most appropriate next step in diagnosis?

   (A) Adenosine stress test
   (B) Cardiac catheterization
   (C) Cardiac MRI with gadolinium
   (D) CT angiography
   (E) Echocardiography with bubble study

3. A 65-year-old man comes to the office because of a 2-year history of progressive shortness of breath on exertion and a 6-month history of nonproductive cough. He now has shortness of breath when walking to his mailbox. He has not had fever, weight loss, or chest pain. He has not had recent sick contacts, has had no occupational exposures, and does not own any pets. He has difficult-to-control atrial fibrillation, hypertension, chronic obstructive pulmonary disease, and migraines. Medications are amiodarone, warfarin, lisinopril, tiotropium, and propranolol. Temperature is 37.0°C (98.6°F), pulse is 80/min and irregular, respirations are 16/min, and blood pressure is 110/70 mm Hg. There is no jugular venous distention. Auscultation of the lungs discloses fine crackles bilaterally, both anteriorly and posteriorly, but no egophony. The remainder of the physical examination discloses no abnormalities. An adverse effect of which of the following medications is the most likely cause of these findings?

   (A) Amiodarone
   (B) Lisinopril
   (C) Propranolol
   (D) Tiotropium
   (E) Warfarin

4. An 18-month-old boy is brought to the emergency department by his mother 30 minutes after he fell from his bed onto the floor. Two months ago, he sustained a fracture of the right humerus when he fell while playing in the park. The fracture healed quickly with immobilization and casting. His mother sustained several bone fractures in early childhood, but she currently does not have any medical concerns. On arrival, the boy is crying. Pulse is 162/min, respirations are 48/min, and blood pressure is 122/80 mm Hg. Examination discloses bluish sclera and ecchymoses and tenderness over the right tibia. X-ray of the right lower extremity confirms a fracture of the tibia. The right lower extremity is immobilized with a cast. Limitation of physical activity is recommended to prevent future fractures. Given this patient's condition, it is most appropriate for which of the following types of screening to be done regularly?

(A) Audiography
(B) DEXA scan
(C) Echocardiography
(D) Retinal examination
(E) Serum calcium and vitamin D concentrations

5. A 70-year-old woman is admitted to the hospital because of a 1-hour history of shortness of breath. She has lung cancer and dementia, Alzheimer type, and has had a decline in mental status during the past month. She lives with her boyfriend of 20 years; he has cared for her and has taken care of the home since she was diagnosed with dementia 2 years ago. She has one adult son who lives out of state, but she speaks to him on the phone daily. The patient's neighbor, who is a nurse and a long-term friend, takes her to all medical appointments and ensures she takes her medications appropriately. The patient is unable to understand the poor prognosis of her condition. She has not designated a health care power of attorney. Which of the following is the most appropriate person to make medical decisions for this patient?

(A) Boyfriend
(B) Neighbor
(C) Patient
(D) Physician
(E) Son

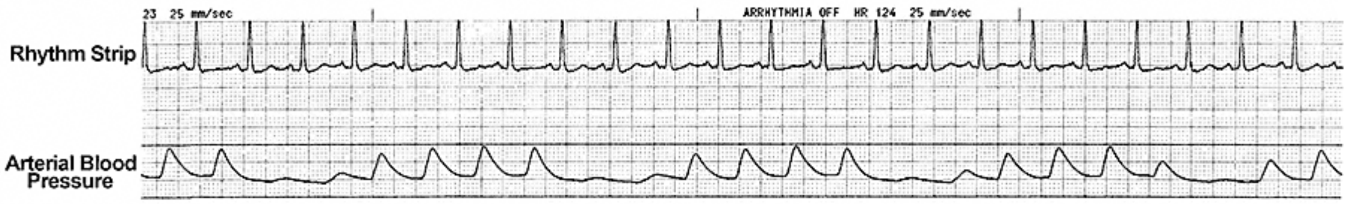

6. A 4-month-old female infant is recovering in the hospital 5 days after uncomplicated surgical repair of a ventricular septal defect via median sternotomy utilizing cardiopulmonary bypass. She was extubated on postoperative day 1 and by postoperative day 4 she was no longer receiving any inotropic infusions or intravenous drugs. She is breast-feeding well; plans are made for discharge from the hospital the next morning. However, on the next morning she develops a temperature of 38.8°C (101.8°F), cool and clammy skin, cold distal extremities, delayed capillary refill, and minimal urine output. Cardiac rhythm strip and tracing of arterial blood pressure are shown. Which of the following is the most appropriate next step in evaluation?

(A) Cardiac catheterization
(B) CT angiography
(C) Echocardiography
(D) Electrophysiology study
(E) MRI of the heart

7. **Patient Information**

Age: 6 years
Sex Assigned at Birth: F
Race/Ethnicity: unspecified
Site of Care: emergency department

History

Reason for Visit/Chief Concern: "My daughter's eyes and legs are swollen."
History of Present Illness:
- 2-week history of cough, congestion, and runny nose that seems to be improving
- parents report the patient's eyes seemed swollen 4 days ago, which they initially attributed to the patient's cold
- 2-day history of swelling over the tops of her feet
- this morning she was unable to put on her shoes because her feet were too swollen

Past Medical History:
- unremarkable

Medications:
- none

Vaccinations:
- up-to-date

Allergies:
- no known drug allergies

Family History:
- mother age 30 years, father age 32 years, and twin sisters age 10 years: alive with no chronic conditions

Physical Examination

Temp	Pulse	Resp	BP	O_2 Sat	Ht	Wt	BMI
37.0°C	140/min	20/min	120/70 mm Hg	99%	115 cm (3 ft 9 in)	25 kg (55 lb)	19 kg/m^2
(98.6°F)				on RA	50th %ile	90th %ile	95th %ile

- Appearance: mildly uncomfortable
- Skin: 2+ pitting edema of both extremities up to the knees
- HEENT: periorbital edema, no erythema; PERRLA; ocular movements are intact
- Pulmonary: clear to auscultation
- Cardiac: regular rhythm; no murmurs
- Abdominal: normoactive bowel sounds; soft, mildly distended, mildly tender to palpation in all quadrants

Diagnostic Studies

Urine	
Specific gravity	1.020 (N=1.003–1.029)
Protein	Large
Ketones	Negative
Blood	Negative
Leukocyte esterase	Negative
WBCs	Negative
RBCs	5–10/hpf

Question: Which of the following additional laboratory findings are most likely to be decreased in this patient?

(A) Hemoglobin
(B) Serum albumin concentration
(C) Serum C3 and C4 concentrations
(D) Serum triglycerides concentration
(E) Serum urea nitrogen concentration

8. A 9-year-old girl is brought to the clinic for a routine examination. She has a 4-year history of asthma. Her only medication is inhaled albuterol with a spacer as needed. She uses it only occasionally and has not used it at all during the past 4 weeks. Her father reports that his daughter has an episode of coughing that awakens her at night once weekly. He also says that his daughter has a sedentary lifestyle because "she coughs if she runs too much." No one in the family smokes cigarettes, and there are no pets. Gas heaters are used in the home. The patient is at the 25th percentile for height and 90th percentile for weight and BMI. Respirations are 16/min. Lungs are clear to auscultation. There is no clubbing of the digits. The remainder of the examination shows no abnormalities. Which of the following is the most appropriate next step in management?

(A) Add oral theophylline to the regimen
(B) Add fluticasone by metered-dose inhaler with a spacer to the regimen
(C) Add salmeterol by metered-dose inhaler with a spacer to the regimen
(D) Begin a 5-day course of oral prednisone
(E) No change in management is necessary

9. A 30-year-old man comes to the office to establish primary care. Medical history is unremarkable and he takes no medications. His father had a myocardial infarction at age 48 years. The patient has smoked one-half pack of cigarettes daily for 10 years. He does not drink alcoholic beverages. He is 168 cm (5 ft 6 in) tall and weighs 82 kg (180 lb); BMI is 29 kg/m^2. Vital signs are within normal limits. The patient is not in distress. Physical examination discloses no abnormalities. Smoking cessation is recommended. Which of the following is the most appropriate screening study for this patient at this time?

(A) ECG
(B) Fasting serum lipid studies
(C) Serum chemistry profile
(D) No screening studies are indicated

10. An 82-year-old woman comes to the office because of a 1-month history of increasing numbness of her feet. She has no history of serious illness. She has taken over-the-counter calcium carbonate tablets for intermittent abdominal pain during the past 40 years; she takes no other medications. She has drunk two glasses of wine with dinner nightly for 50 years. On examination, gastrocnemius deep tendon reflexes are absent. Babinski sign is absent bilaterally. Light touch to the distal lower extremities and feet produces a tingling sensation. Sensation to vibration over the great toes is severely decreased. There is a mild to moderate decrease in proprioception of the great toes. Which of the following is most likely to prevent progression of these neurologic findings?

(A) Alcohol cessation
(B) Calcium carbonate cessation
(C) Folic acid supplementation
(D) Niacin supplementation
(E) Vitamin B_1 (thiamine) supplementation
(F) Vitamin B_{12} (cyanocobalamin) supplementation

11. A 50-year-old man comes to the office for a health maintenance examination. He says he has felt well and reports no symptoms. Medical history is unremarkable. He takes no medications. He uses up to three cans of chewing tobacco weekly and occasionally sleeps with tobacco in his mouth. He says he would like to quit but cannot seem to do it on his own. Vital signs are within normal limits. Examination shows a whitish discoloration that measures approximately 1 cm in diameter on the buccal mucosa. The tongue has a brownish discoloration. Palpation of the neck discloses no lymphadenopathy. In addition to encouraging the patient to quit using chewing tobacco, which of the following is the most appropriate next step in management?

(A) Prescribing oral nystatin suspension
(B) Surgical biopsy of the oral lesion
(C) Swabbing of the white area and sending for cytology
(D) Observation only

12. A 48-year-old man is brought to the emergency department by ambulance 45 minutes after he collapsed at home. He did not have loss of consciousness. He has a 6-week history of shortness of breath and severe fatigue when he walks 20 to 30 feet. During the past 6 weeks, he also has had a 5-kg (11-lb) weight gain despite a decreased appetite. He has no history of serious illness. He cannot remember his last visit to a physician. His only medication is ibuprofen for intermittent mild headaches. His father died of "heart problems" at the age of 78 years. The patient has smoked one pack of cigarettes daily for 22 years. He has consumed 24 cans of beer weekly for 20 years. He works as a painting and drywall contractor but was unable to work last week because of fatigue. Temperature is 36.8°C (98.2°F), pulse is 100/min and regular, respirations are 16/min, and blood pressure is 90/55 mm Hg while supine. Pulse oximetry on 4 L/min of oxygen by nasal cannula shows an oxygen saturation of 91%. On examination, crackles are heard halfway up the lung fields bilaterally. An S_3 is heard; no murmurs are heard. The liver span is 5 cm. There is moderate pitting edema of the calves and ankle swelling bilaterally. Echocardiography is most likely to show which of the following findings?

(A) A large pericardial effusion
(B) Dilated cardiomyopathy
(C) Left ventricular hypertrophy
(D) Paradoxical septal motion
(E) Regional wall motion abnormality

13. A 68-year-old man comes to the clinic because of a 6-month history of frequent falls. He sustained a fracture of the left wrist 4 weeks ago during a fall. He has not had loss of consciousness before or during the falls. He says that turning and pivoting often cause him to fall, and he has a tendency to fall backward when walking. He has Parkinson disease treated with carbidopa-levodopa and entacapone. Vital signs are within normal limits. Examination shows masked facies and a resting tremor of the right upper extremity. Romberg sign is absent. When the patient stands still and is pulled backward, he is unable to maintain his posture. Which of the following is the most appropriate intervention to decrease this patient's risk for future falls?

(A) Biofeedback
(B) Physical therapy
(C) Pramipexole therapy
(D) Ropinirole therapy
(E) Rotigotine therapy

14. A 6-year-old boy is brought to the office by his parents as a new patient. Three months ago, he and his 3-year-old sister and 4-year-old brother were adopted from a Russian orphanage, where they had lived for 2 years. Their biological parents died in a motor vehicle collision; they have no other relatives. The adoptive parents say they were told that the children had stable caregivers, age-appropriate stimulation, and schooling at the orphanage. The children were shy initially on meeting them but now seem comfortable. They play well with each other; the patient is protective of his siblings. His medical and developmental history prior to 2 years ago is unknown. He has been learning English slowly; his parents hope he will learn enough by the end of the summer to begin kindergarten, a grade below that of his peers. Although he can perform basic mathematics, he is unable to read, even in Russian. Results of receptive language testing are consistent with an average IQ. His daily living skills are appropriate for age. He is at the 10th percentile for height and weight. During the examination, the patient's adoptive mother, who is fluent in Russian, translates for him. Physical examination shows no abnormalities. On mental status examination, he is shy and maintains intermittent eye contact. He slowly answers questions with brief statements. He smiles and says he is excited and pleased about being adopted. Results of laboratory studies are within the reference ranges. Which of the following is the most likely diagnosis?

(A) Autism spectrum disorder
(B) Fetal alcohol syndrome
(C) Intellectual developmental disorder
(D) Learning disorder
(E) Post-traumatic stress disorder
(F) Reactive attachment disorder

15. A 17-year-old boy is brought to the emergency department because of a 1-hour history of progressive difficulty breathing and a 2-hour history of an itchy, red rash over his trunk, arms, and legs. He has not used new soaps, detergents, or lotions during the past week. Thirty-six hours ago, he began a 1-week course of amoxicillin for an ear infection; he has taken four doses since that time. He has no other history of serious illness and no known allergies. He takes no other medications. Pulse is 100/min, respirations are 28/min, and blood pressure is 90/60 mm Hg. Pulse oximetry on room air shows an oxygen saturation of 95%. He appears anxious and is in moderate respiratory distress. Examination shows an erythematous, raised, demarcated rash over the trunk and all extremities. Which of the following is the most appropriate next step in management?

(A) Administration of albuterol
(B) Administration of diphenhydramine
(C) Administration of epinephrine
(D) Complete blood count
(E) Observation only

16. Two weeks after undergoing open cholecystectomy for gangrenous cholecystitis, a 47-year-old woman comes to the clinic for removal of surgical staples. She was treated with oral vancomycin for *Clostridioides difficile* colitis during hospitalization. She has type 2 diabetes mellitus. Current medications are metformin and pantoprazole. She reports loose stools four times daily during the past 2 weeks. In addition to wearing a clean isolation gown, which of the following is the most appropriate precaution for the physician to take?

(A) Washing hands thoroughly with alcohol gel
(B) Washing hands thoroughly with soap and water
(C) Wearing a surgical mask
(D) Wearing sterile gloves and preparing the staple skin line with chlorhexidine
(E) No additional precautions are necessary

The abstract displayed on this page is for use with items #17–19 on the following page.

Question

In patients with opioid use disorder, what is the relative efficacy of buprenorphine and methadone maintenance therapy?

Review scope

Included studies compared buprenorphine maintenance therapy (BMT), >1 mg/day, with methadone maintenance therapy (MMT), ≥20 mg/day, or placebo (including 1 mg/day of buprenorphine) in patients who were dependent on heroin or other opioids. Studies of pregnant women and those assessing buprenorphine or methadone for detoxification but with no maintenance phase were excluded. Outcomes included treatment retention and urinalysis-confirmed use of morphine, cocaine, and benzodiazepines.

Review methods

MEDLINE and EMBASE/Excerpta Medica (2003 to Jan 2013), Cochrane Central and Cochrane Library (2013, Issue 1), PsycLIT, Current Contents, several other databases, ClinicalTrials.gov and other electronic sources of ongoing trials, conference proceedings, Library of Congress databases, national focal points for drug research, and reference lists were searched for randomized controlled trials (RCTs). Authors were consulted. 31 RCTs (n=5430), ranging in size from 40 to 736 patients, met the selection criteria. Duration of study interventions ranged from 2 weeks to 52 weeks. 20 trials compared BMT with MMT, and 11 compared BMT with placebo.

Main results

As shown in the table below, low-, medium-, and high-dose BMT increased treatment retention more than placebo; BMT did not differ from equivalent doses of MMT for retention or had lower retention.

High-dose (standard mean difference −1.17, 95% CI −1.85 to −0.49), but not medium- or low-dose, BMT decreased morphine use more than placebo. Medium- and flexible-dose BMT and MMT did not differ for morphine use or cocaine use. BMT vs MMT and high-dose BMT vs placebo did not differ for benzodiazepine use.

Buprenorphine maintenance therapy (BMT) vs methadone maintenance therapy (MMT) or placebo for treatment retention in patients with opioid use disorder

Comparisons*	Number of Trials (n)	Weighted Retention Rates	RBI (95% CI)	NNT (95% CI)
High-dose BMT vs placebo	5 (1001)	72% vs 40%	82% (15 to 190)	4 (3 to 7)
Medium-dose BMT vs placebo	4 (887)	66% vs 38%	74% (6 to 187)	4 (2 to 34)
Low-dose BMT vs placebo	5 (1131)	60% vs 40%	50% (19 to 88)	5 (4 to 10)
			**RBR (95% CI)**	**NNH (95% CI)**
High-dose BMT vs high-dose MMT	1 (134)	5.2% vs 6.6%	21% (−216 to 20)	Not significant
Medium-dose BMT vs medium-dose MMT	7 (780)	43% vs 48%	13% (−10 to 31)	Not significant
Flexible-dose BMT vs flexible-dose MMT	11 (1391)	50% vs 63%	17% (5 to 27)	8 (5 to 25)
Low-dose BMT vs low-dose MMT	3 (253)	38% vs 56%	33% (13 to 48)	6 (4 to 17)

CI = confidence interval; NNH = number needed to harm; NNT = number needed to treat; RBI = relative benefit increase; RBR = relative benefit reduction.
*Flexible dose = dose is titrated within a broad dose range according to patient preference; low-dose BMT = 2 to 6 mg; medium-dose BMT = 7 to 15 mg; high-dose BMT = 16 mg; medium-dose MMT = 40 to 85 mg.

Conclusions

In patients with opioid use disorder, buprenorphine is more efficacious than placebo, but less efficacious than methadone therapy, for treatment retention.

Source of funding: Australian Government Department of Health.

Structured abstract based on: Mattick RP, Breen, C, Kimber J, Davoli M. **Buprenorphine maintenance versus placebo or methadone maintenance for opioid dependence**. Cochrane Database Syst Rev. 2014;(2):CD002207. 24500948

Items #17–19 refer to the abstract displayed on the previous page.

17. A 38-year-old woman comes to the clinic for follow-up examination. She has opioid use disorder and currently takes methadone 40 mg daily as maintenance therapy. She says the methadone is causing mild sedation that is interfering with her job. She would like to discontinue methadone maintenance therapy but is concerned about heroin relapse. She says she also takes a friend's valium when she is feeling "stressed out." The physician discusses alternative maintenance therapy strategies with the patient. The physician discusses the pros and cons of medium- or high-dose buprenorphine maintenance therapy compared with medium- or high-dose methadone therapy with the patient. Based on this abstract, which of the following is the most appropriate information for the physician to convey to the patient?

(A) Buprenorphine is 13% and 21% less effective than methadone at medium and high doses, respectively
(B) Buprenorphine is a better maintenance strategy if the patient is also regularly using benzodiazepines
(C) Methadone is statistically more likely to result in treatment retention
(D) Neither drug is more effective than placebo
(E) There is an unclear difference in efficacy between the two drugs at these doses

18. Which of the following aspects of the study is most likely to limit confidence in the authors' conclusions?

(A) Exclusion of patients using methadone for acute detoxification
(B) Exclusion of pregnant patients
(C) Inclusion of randomized controlled trials only
(D) Inclusion of trials that compared active drug with placebo
(E) Inclusion of trials with an intervention duration of 2 weeks
(F) Use of urinalysis to determine treatment retention

19. Which of the following is most likely to bias the results of this study?

(A) Earlier diagnosis of relapse in patients receiving methadone maintenance therapy
(B) Exclusion criteria of studies used for the review
(C) Likelihood that more trials with positive results will be published
(D) Source of funding
(E) Variety of sample sizes of the included trials

END OF SET

20. A 57-year-old man is brought to the emergency department by ambulance 30 minutes after he had loss of consciousness. His wife says he was making dinner when he fell on the kitchen floor. He has hypertension and his only medication is lisinopril. He has no history of operative procedures. En route, his temperature was 37.2°C (99.0°F), pulse was 90/min, respirations were 10/min and labored, and blood pressure was 180/100 mm Hg. Pulse oximetry on 40% oxygen by face mask showed an oxygen saturation of 99%. On arrival, he is unresponsive. His Glasgow Coma Scale score is 3. He is intubated and mechanically ventilated. Temperature is 37.2°C (99.0°F), pulse is 90/min, ventilatory rate is 10/min, and blood pressure is 180/100 mm Hg. Pulse oximetry on an FIO_2 of 0.4 shows an oxygen saturation of 98%. On examination, Doll's eye (oculocephalic) maneuver shows absent eye movements, and the corneal reflex is absent. On ice-water caloric testing, there is no nystagmus or deviation of the eyes toward the ear being irrigated. CT scan of the head shows a ruptured cerebral aneurysm. When the patient's wife is notified of the patient's condition, she says he wanted to be an organ donor but does not have an advance directive. Which of the following is the most appropriate next step in management?

(A) Consult with the hospital ethics committee
(B) Contact the organ bank for potential donation
(C) Determine if there are any patients awaiting organ donation in the hospital
(D) Explain that the patient is not a candidate for organ donation because he does not have an advance directive
(E) Explain that the patient is not a candidate for organ donation because of his history of hypertension

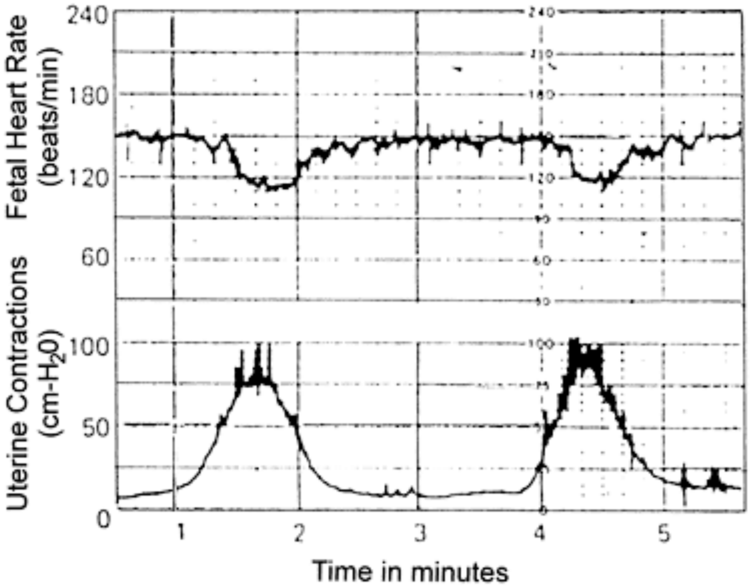

21. A 36-year-old woman, gravida 2, para 1, at 39 weeks' gestation is admitted to the hospital in labor. She reports painful contractions that occur every 2 to 3 minutes and last 60 seconds. She has not had vaginal bleeding. She has no history of serious illness, and pregnancy has been uncomplicated. Temperature is 37.0°C (98.6°F), pulse is 84/min and regular, and blood pressure is 100/70 mm Hg. The abdomen is nontender and consistent in size with a 39-week gestation. External fetal monitoring shows a heart rate of 150/min with moderate variability, several spontaneous accelerations, and no decelerations. An external tocometer shows regular uterine contractions every 2 to 3 minutes. The cervix is 6 cm dilated and 100% effaced; the vertex is at 0 station. Ultrasonography shows the fetus in a cephalic presentation. The membranes are artificially ruptured, yielding copious clear fluid. Thirty minutes later, the cervix is 9 cm dilated and 100% effaced; the vertex is at +1 station. A fetal heart tracing is shown. Which of the following is the most appropriate next step in management?

(A) Advising the patient to begin pushing
(B) Amnioinfusion
(C) Expectant management
(D) Forceps-assisted vaginal delivery
(E) Immediate cesarean delivery

---

22. A 27-year-old woman comes to the office because of a 3-week history of nasal congestion, cough productive of sputum, intermittent moderate headache, and intermittent moderate pain over her cheeks. She has had similar symptoms three to four times yearly during the past 5 years. The symptoms last 2 to 8 weeks and generally resolve spontaneously. She has had two episodes of bacterial pneumonia during the past 5 years; both episodes resolved with antibiotic therapy. Her only current medication is acetaminophen as needed for headache. Vital signs are within normal limits. Examination shows no abnormalities. Serum study results show decreased IgA, IgG, and IgM concentrations. Antibiotic therapy is begun, and the patient's condition improves. Which of the following is most likely to decrease the likelihood of recurrent infection in this patient?

(A) Daily inhaled tobramycin therapy
(B) Daily intranasal glucocorticoid therapy
(C) Daily trimethoprim-sulfamethoxazole therapy
(D) Intranasal influenza virus vaccine administration
(E) Monthly immune globulin replacement therapy

23. A 37-year-old man comes to the office because of a 2-year history of mild to moderate fatigue. The fatigue waxes and wanes without relation to activity. He has no history of serious illness and takes no medications. He drinks three glasses of whiskey weekly. He has had two lifetime female sexual partners; he uses condoms consistently. He immigrated to the United States from China 15 years ago. Vital signs are within normal limits. Examination discloses no abnormalities. Results of laboratory studies are shown:

Serum	
Creatinine	1.2 mg/dL
ALT	85 U/L
AST	60 U/L
HBsAg	positive
Anti-HCV	negative
Anti-HBs	negative
IgG anti-HBc	positive
Blood	
Hematocrit	35%
Hemoglobin	12 g/dL
MCV	82 μm^3

Without treatment of his current condition, this patient is most likely to develop which of the following?

(A) Amyloidosis
(B) Essential mixed cryoglobulinemia
(C) Hepatocellular carcinoma
(D) Membranoproliferative glomerulonephritis
(E) Polyarteritis nodosa
(F) Sjögren syndrome

24. A 10-year-old boy is brought to the office because of moderate right knee pain. He is in no distress. Medical history is remarkable for hemophilia A. Examination of the right knee shows warmth and swelling; there is pain with flexion. Range of motion is limited by pain and swelling. Which of the following is the most appropriate therapy for this patient?

(A) ADH (vasopressin)
(B) Factor VIII concentrate
(C) Factor IX concentrate
(D) Ferrous sulfate
(E) Fresh frozen plasma

25. A 72-year-old man comes to the clinic because of a 3-week history of visual changes in his right eye. During this period, he has noticed that the lines of his daily crossword puzzle look curved, and the blinds in his apartment appear wavy. He wears magnifying lenses for reading. He has not had pain in his eye or photophobia. He has no history of trauma to the area. On examination, visual acuity is 20/200 in the right eye and 20/100 in the left eye. Both pupils are round and reactive to light. Palpation of both globes through closed eyelids shows no abnormalities. There is normal red reflex bilaterally. The lenses appear clear. Which of the following is the most likely diagnosis?

(A) Cataracts
(B) Central retinal artery occlusion
(C) Closed-angle glaucoma
(D) Macular degeneration
(E) Temporal arteritis

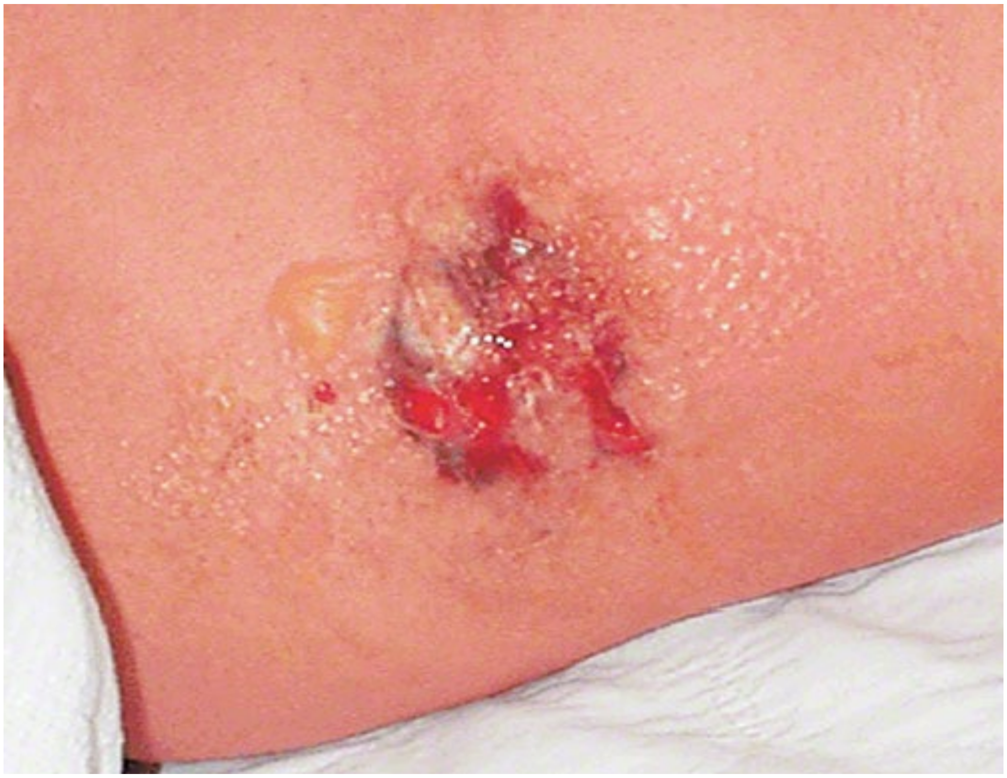

26. A 28-year-old man comes to the clinic because of a 2-day history of a red, severely painful lesion on his left forearm. He says that he felt stinging pain as he put his shirt on in the dark 2 days ago; the pain was so severe he immediately threw down the shirt and saw a spider running across the floor. He returned yesterday from a 1-week trip to the mountains in the southeastern United States. He has type 1 diabetes mellitus treated with lisinopril and insulin. He does not drink alcoholic beverages. He is sexually active with two male partners and uses condoms inconsistently. Vital signs are within normal limits. A photograph of the lesion is shown. No other abnormalities are noted. Which of the following physical findings is most likely to appear during the next 24 hours?

(A) Areas of eschar at the center of the wound site
(B) Gangrene of the distal aspect of the left extremity
(C) Generalized upper extremity edema
(D) Palpable purpuric lesions across the trunk and upper extremities
(E) Rapidly spreading erythema around the wound site
(F) Tender, erythematous streaks up to the epitrochlear nodes

---

27. A 37-year-old man comes to the clinic for a health maintenance examination prior to employment. He feels well. He has no history of serious illness and takes no medications. One year ago, his blood pressure was 136/85 mm Hg. He is 173 cm (5 ft 8 in) tall and weighs 100 kg (220 lb); BMI is 33 kg/m^2. Today, his blood pressure is 138/87 mm Hg. Examination shows no abnormalities. In addition to recommending weight loss, which of the following is the most appropriate next step to prevent cardiovascular morbidity in this patient?

(A) ACE inhibitor therapy
(B) Calcium supplementation
(C) DASH diet
(D) Fish oil supplementation
(E) Thiazide diuretic therapy

28. A 39-year-old man comes to the office because of a 6-week history of mild fatigue and increased thirst and urination. During this time, he also has had a 6.8-kg (15-lb) weight loss despite no change in appetite. He is otherwise asymptomatic. He has no history of serious illness and takes no medications. He is 180 cm (5 ft 11 in) tall and weighs 61 kg (135 lb); BMI is 19 kg/m^2. Temperature is 37.0°C (98.6°F), pulse is 80/min, respirations are 18/min, and blood pressure is 118/70 mm Hg. Examination shows no abnormalities. Results of laboratory studies are shown:

Serum	
Na^+	135 mEq/L
K^+	4.2 mEq/L
Cl^-	100 mEq/L
Urea nitrogen	25 mg/dL
Glucose	578 mg/dL
Blood	
Hemoglobin A_{1c}	10.7%

In addition to recommending dietary modification, which of the following is the most appropriate next step in management?

(A) Glyburide therapy
(B) Insulin therapy
(C) Metformin therapy
(D) Pioglitazone therapy
(E) Sitagliptin therapy

29. Two days after admission to the hospital for treatment of a fractured femur and closed head injury sustained in a motor vehicle collision, an 8-year-old girl has a brief, generalized tonic-clonic seizure. CT scan of the head on admission disclosed no abnormalities. She has no history of serious illness. Oral acetaminophen was initiated for pain, and the patient underwent open reduction and internal fixation of the fracture. She receives no routine medications. Today, she appears drowsy but responds to questions appropriately. She is at the 55th percentile for height, weight, and BMI. Temperature is 36.8°C (98.2°F), pulse is 92/min, respirations are 24/min, and blood pressure is 106/64 mm Hg. Pulse oximetry on room air shows an oxygen saturation of 95%. Pupils are equal and reactive to light and accommodation. Ocular movements are full. Muscle strength is 5/5, and deep tendon reflexes are normal. Results of serum studies are shown:

Na^+	122 mEq/L
K^+	3.8 mEq/L
Cl^-	94 mEq/L
HCO_3^-	24 mEq/L
Glucose	80 mg/dL
Calcium	8.8 mg/dL

Which of the following is the most appropriate next step in management?

(A) Administer a bolus of intravenous 3% saline
(B) Administer subcutaneous vasopressin
(C) Initiate fosphenytoin therapy
(D) Initiate therapy with 0.9% saline at 1.5 times the maintenance requirements
(E) Order EEG
(F) Order MRI of the brain

30. A 43-year-old man comes to the office because of a 2-week history of fatigue and mild pain in his right armpit. He has not had fever or weight loss. Medical history is remarkable for type 2 diabetes mellitus and hypercholesterolemia. Medications are metformin, simvastatin, and aspirin. He is a social worker and frequently visits clients in homeless shelters. He lives alone with two dogs and a cat and has one male sexual partner. Vital signs are within normal limits. He appears well. Examination of the right upper extremity shows an erythematous axilla with a 4-cm, mobile, tender, nonfluctuant axillary lymph node and a tender, 1-cm epitrochlear lymph node. Abdominal examination shows no hepatosplenomegaly. Which of the following is the most likely diagnosis?

(A) Castleman disease
(B) Cat-scratch disease
(C) Hidradenitis suppurativa
(D) T-cell lymphoma
(E) Tuberculosis

31. A 2-year-old girl is brought to the office by her parents for a well-child examination. She was adopted 2 months ago. She has no history of serious illness and receives no medications. Vital signs are within normal limits. Examination shows no abnormalities. After the examination, the parents ask when they should tell their daughter about the adoption. Which of the following is the most appropriate advice regarding the best time to tell this patient?

(A) After she turns 18 years of age
(B) As early as possible, even if she cannot process the whole experience
(C) Once the seal on the adoption records has been lifted
(D) Right before she is enrolled in kindergarten
(E) When she learns that she is not biologically related to her parents

32. A surgical intensive care unit observes that the number of days that patients have central venous catheters in place and the number of bloodstream infections have been increasing. A multidisciplinary team from the unit would like to decrease the number of days that central venous catheters are in place in order to improve performance in this area. Which of the following is the most appropriate next step?

(A) Ask clinical providers in the unit to identify what they believe to be the most common factors leading to nonremoval of central venous catheters
(B) Change all central venous catheters every 5 days
(C) Place stickers on all intensive care unit doors reminding staff to reassess the need for a central line
(D) Plan a randomized controlled trial to test the effects of a reminder to reassess the need for a central line on a daily goals sheet implemented during rounds

33. A 17-year-old girl comes to the office because of a 3-month history of daily moderate epigastric pain that is more severe after meals. Three weeks ago, she had watery diarrhea for 2 days, which resolved spontaneously. She has not had vomiting. She has no history of serious illness and takes no medications. Vital signs are within normal limits. Examination, including abdominal examination, shows no abnormalities. Which of the following is most likely to confirm the diagnosis?

(A) CT scan of the abdomen
(B) Endoscopy
(C) Examination of the stool for ova and parasites
(D) Stool culture
(E) Upper gastrointestinal series

34. A 23-year-old man comes to the emergency department because of a 1-hour history of intermittent coughing spasms that began suddenly while he was attempting to clean mold from his garage with a mixture of acetic acid and cleaning agents. The cough is not productive. He reports shortness of breath only during the coughing spasms. Medical history is unremarkable and he takes no medications. He does not smoke cigarettes, drink alcoholic beverages, or use other substances. He is 180 cm (5 ft 11 in) tall and weighs 75 kg (165 lb); BMI is 23 kg/m^2. Vital signs are within normal limits. Pulse oximetry on room air shows an oxygen saturation of 94%. The patient has periodic paroxysmal coughing during the examination. Oropharynx is clear. Auscultation of the lungs discloses bilateral wheezes throughout all lung fields. There is no use of accessory muscles of respiration. The remainder of the physical examination discloses no abnormalities. Chest x-ray shows mild hyperexpansion of the lungs bilaterally. Results of arterial blood gas analysis on room air are shown:

P_{O_2}	72 mm Hg
P_{CO_2}	38 mm Hg
pH	7.41
HCO_3^-	23 mEq/L

Which of the following is the most appropriate initial step in management?

(A) Albuterol therapy
(B) Bronchoscopy
(C) CT scan of the chest
(D) Intubation
(E) Methylprednisolone therapy

35. An 87-year-old man is admitted to the hospital because of a 4-day history of moderate abdominal cramps, nausea, and vomiting. He has not had a bowel movement or passed gas in the past 24 hours. He has colon cancer metastatic to the lungs, liver, and peritoneum. Eighteen months ago, he underwent a low anterior resection for T3N2 rectal cancer. Six months ago, exploratory laparotomy showed diffuse carcinomatosis and bowel obstruction, and an ileotransverse bypass was performed. The patient and his family were informed of the findings and advised that he would not be a candidate for a laparotomy in the future because there would be no surgical options to treat another obstruction. The patient has no other history of serious illness. Medications are 5-fluorouracil and bevacizumab. Temperature is 37.2°C (99.0°F), pulse is 100/min, respirations are 20/min, and blood pressure is 130/80 mm Hg. The abdomen is distended and mildly tender to palpation. There are no peritoneal signs. CT scan of the abdomen shows a mid-jejunal small-bowel obstruction, multiple intraperitoneal tumor deposits, and ascites. The physician recommends insertion of nasogastric and percutaneous endoscopic gastrostomy tubes. The patient and his family request that the patient undergo a laparotomy and intestinal bypass. Which of the following is the most appropriate next step in management?

(A) Abide by the wishes of the patient and his family
(B) Alter the chemotherapy regimen
(C) Explain the futility of the operation to the patient and his family
(D) Offer to transfer the patient to another facility
(E) Suggest external beam radiation therapy

36. A 62-year-old woman comes to the office because of a 3-month history of anxiety, insomnia, and frequent bowel movements. She has had a 9-kg (20-lb) weight loss during this period. Pulse is 90/min and irregularly irregular, respirations are 22/min, and blood pressure is 150/65 mm Hg. She is restless. The thyroid gland is diffusely enlarged. A bruit is heard over the thyroid. Examination shows palmar erythema; some of her fingernails are separated from the nail beds. There is widening of the palpebral fissures and thickening of the skin over the dorsum of the feet with a peau d'orange appearance. There is a fine tremor. Which of the following is the most likely diagnosis?

(A) Addison disease
(B) Carcinoid syndrome
(C) Cushing syndrome
(D) Pheochromocytoma
(E) Thyrotoxicosis

37. **Patient Information**

Age: 28 years
Gender: M, self-identified
Race/Ethnicity: unspecified
Site of Care: clinic

History

Reason for Visit/Chief Concern: follow-up 1 week after undergoing colonoscopy because of a 3-month history of hematochezia and iron deficiency anemia

History of Present Illness:

- 3-month history of three loose bowel movements daily
- no fever or abdominal pain
- since colonoscopy, no bright red blood per rectum
- reports no new symptoms today

Past Medical History:

- generalized anxiety disorder

Medications:

- citalopram

Allergies:

- no known drug allergies

Family History:

- mother and father alive with no chronic conditions

Psychosocial History:

- has smoked two packs of cigarettes daily for 10 years
- does not drink alcoholic beverages

Physical Examination

Temp	Pulse	Resp	BP	O_2 Sat	Ht	Wt	BMI
36.1°C (97.0°F)	76/min	14/min	100/62 mm Hg	–	170 cm (5 ft 7 in)	59 kg (130 lb)	20 kg/m^2

- Abdominal: scaphoid; normoactive bowel sounds; liver span is 9 cm by percussion; mild tenderness to palpation over the right lower quadrant without rebound or guarding; no hepatosplenomegaly

Diagnostic Studies

- colonoscopy: areas of coalesced ulcers with normal appearing mucosa between, involving the ileum

Question: Which of the following is the most appropriate recommendation for this patient considering his condition?

(A) Annual screening for lymphoproliferative disorders
(B) Discontinuation of citalopram therapy
(C) Prophylactic colectomy
(D) Smoking cessation

38. A 19-year-old man is brought to the emergency department (ED) by ambulance 15 minutes after he was stabbed in the chest during a fight. The stab wound is located medially to the left nipple. At the scene, pulse was 105/min, respirations were 18/min, and blood pressure was 120/80 mm Hg. Pulse oximetry on room air showed an oxygen saturation of 96%. Two large-bore intravenous catheters were placed en route to the ED, and lactated Ringer solution was initiated. On arrival in the trauma bay, the patient develops a pulse of 130/min and blood pressure of 80/40 mm Hg. There is jugular venous distention. Results of focused assessment with sonography for trauma (FAST) are most likely to lead to which of the following next steps for this patient?

(A) CT scan of the chest and abdomen with contrast
(B) Exploratory laparotomy
(C) Needle decompression and chest tube placement
(D) Pericardiocentesis
(E) Peritoneal lavage

39. A 47-year-old woman is scheduled to undergo right knee arthroscopy and partial medial meniscectomy. Medical history is otherwise unremarkable. At her preoperative examination 2 weeks ago, her surgeon discussed the risks and benefits of the procedure, and both parties signed the operative consent form. On the patient's arrival at the hospital, the paperwork is reviewed. She is taken into the operating room and sedated, and the anesthesiology team starts a spinal anesthetic. After the patient's right lower extremity is prepped for the procedure, a surgical pause is taken to review the patient's name and consent form and to confirm the site of surgery. The operating room team is unable to locate the consent form for review. No markings are present on the patient's extremities to indicate the site of the procedure. The surgeon says that he remembers reviewing the consent form with the patient and seeing her sign it. The patient's husband is in the waiting room. Which of the following is the most appropriate course of action?

(A) Ask the patient if she recalls signing the consent form and, if so, proceed with the operation
(B) Ask the patient to sign a new consent form and proceed with the operation
(C) Do not proceed, and remove the patient from the operating room
(D) Obtain verbal consent from the patient's husband before proceeding with the operation
(E) Proceed with the operative procedure as planned

40. A 30-year-old man comes to the physician because he and his wife have been unable to conceive during the past 2 years. Previous evaluation of his wife showed no abnormalities. He has not been exposed to pesticides, heavy metals, radiation, or testicular overheating. Physical examination shows no abnormalities. Serum studies show a testosterone concentration within the lower reference range. A diagnosis of oligospermia is made. Which of the following is the most appropriate initial action by the physician?

(A) Ask the patient about his alcohol intake, smoking, and stress level
(B) Educate the patient about declining sperm counts with increased age
(C) Prescribe a phosphodiesterase inhibitor
(D) Provide a topical testosterone gel
(E) Recommend in vitro fertilization

BLOCK 2, ITEMS 41–80

41. A 40-year-old man comes to the office during the summer because of a 3-day history of fever, diffuse muscle aches, fatigue, and sore throat. He has had no sick contacts. Medical history is unremarkable and he takes no medications. He received the influenza virus vaccine this year. Ten days ago, he returned from a trip to Malaysia, where he says he enjoyed the local food and nightlife. Temperature is 38.8°C (101.8°F), pulse is 90/min, respirations are 16/min, and blood pressure is 126/70 mm Hg. The remainder of the examination shows no abnormalities. Results of laboratory studies are shown:

Blood	
Hemoglobin	10 g/dL
MCV	90 μm^3
WBC	2400/mm^3
Neutrophils, segmented	60%
Neutrophils, bands	5%
Lymphocytes	5%
Monocytes	20%
Eosinophils	4%
Basophils	6%
Platelet count	350,000/mm^3
Red cell distribution width	14%

Monospot test result is negative. Which of the following is the most appropriate next step in diagnosis?

(A) Cytomegalovirus serologic testing
(B) Epstein-Barr virus serologic testing
(C) Hemagglutination inhibition assay
(D) HIV RNA polymerase chain reaction testing
(E) Serum hepatitis B surface antigen testing

42. A 22-year-old man is brought to the emergency department by his girlfriend 30 minutes after she found him unconscious in his home. She last saw him yesterday and he appeared well. He has a history of cocaine and heroin use, and also use of prescription narcotic analgesics that he obtains from friends. On arrival, he is lethargic. Temperature is 35.0°C (95.0°F), pulse is 68/min, respirations are 8/min, and blood pressure is 96/50 mm Hg. Pulse oximetry on room air shows an oxygen saturation of 96%. The left lower extremity is cyanotic and cold to the touch. The remainder of the examination shows no abnormalities. Results of serum studies are shown:

K^+	5.5 mEq/L
HCO_3^-	18 mEq/L
Creatinine	1 mg/dL
Creatine kinase	50,000 U/L

This patient is at greatest risk for which of the following conditions?

(A) Acute kidney injury
(B) Acute liver failure
(C) Cardiac arrhythmia
(D) Hypocalcemia
(E) Hypophosphatemia

43. A 62-year-old woman is brought to the emergency department (ED) by her husband because of a 3-day history of increasing confusion. She has a 20-year history of bipolar disorder well controlled with medication for the past 5 years. Her husband forgot to bring her medication bottles and cannot remember which medication she takes. He reports that she has recently had mild headaches and has taken acetaminophen as needed. On questioning, he says that there has been no particular change in their routine, but during the past 2 days they have been outside playing tennis more than usual because of the onset of sunny, warm weather. While in the ED, the patient has a generalized tonic-clonic seizure lasting 2 minutes. She is 163 cm (5 ft 4 in) tall and weighs 72 kg (160 lb); BMI is 27 kg/m^2. Pulse is 104/min, respirations are 18/min, and blood pressure is 140/90 mm Hg. Examination shows dry mucous membranes. ECG shows a second-degree atrioventricular block. Which of the following is most likely responsible for this patient's confusion?

(A) Acetaminophen
(B) Bupropion
(C) Lithium
(D) Risperidone
(E) Topiramate

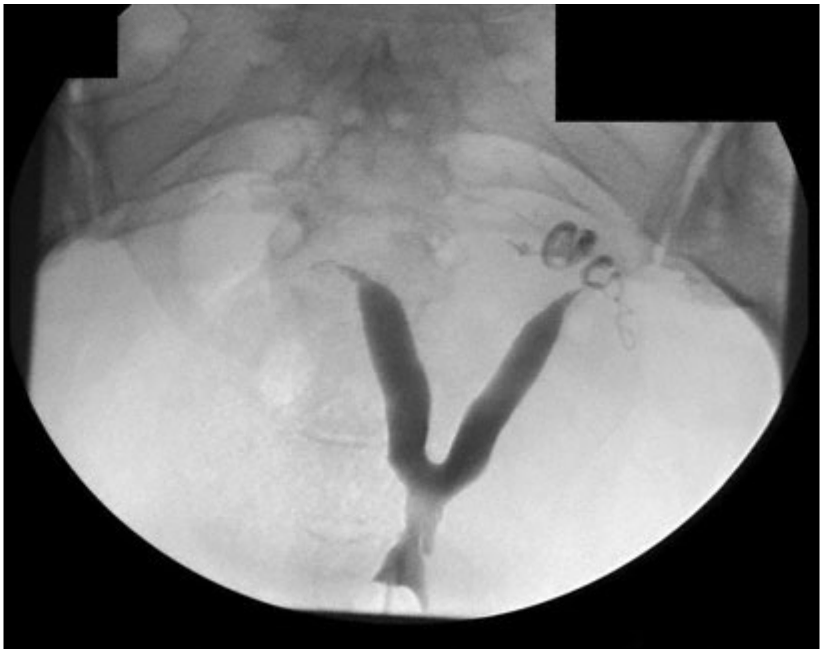

44. A 32-year-old nulligravid woman comes to the office because she has been unable to conceive during the past 12 months. She and her husband have unprotected sexual intercourse three times weekly. Two years ago, she was involved in a motor vehicle collision; CT scan at that time showed absence of the left kidney. She has no other history of serious illness, and her only medication is a prenatal vitamin. She has no known allergies. Vital signs are within normal limits. Pelvic examination shows no abnormalities. Hysterosalpingography is shown. When this patient conceives, she is at greatest risk for which of the following pregnancy complications?

(A) Fetal macrosomia
(B) Multiple gestation
(C) Oligohydramnios
(D) Preeclampsia
(E) Preterm labor

45. **Patient Information**

Age: 14 years
Gender: F, self-identified
Ethnicity: unspecified
Site of Care: office

History

Reason for Visit/Chief Concern: "I feel light-headed."

History of Present Illness:
- 1-week history of episodes of light-headedness
- almost lost consciousness during three episodes
- experienced tunnel vision and almost fell when getting out of bed this morning
- evaluated 10 days ago for follow-up of attention-deficit/hyperactivity disorder; clonidine therapy initiated at that time
- sister is currently hospitalized for meningitis; patient has been receiving prophylactic rifampin

Past Medical History:
- moderate persistent asthma that is well controlled
- last asthma exacerbation was 1 week ago; 5-day course of prednisone was initiated at that time, which she completed
- attention-deficit/hyperactivity disorder diagnosed at age 8 years; has been on numerous different medications

Medications:
- albuterol
- fluticasone
- methylphenidate
- clonidine
- rifampin

Vaccinations:
- up-to-date

Allergies:
- no known drug allergies

Family History:
- mother and father: alive with no chronic conditions
- sister: currently being treated for meningitis

Psychosocial History:
- lives with parents, 18-year-old sister, and 2-year-old sister
- has difficulty in school because of attention-deficit/hyperactivity disorder
- does not smoke cigarettes, drink alcoholic beverages, or use nonprescribed medications or other substances
- not sexually active

Physical Examination

Temp	Pulse	Resp	BP	O_2 Sat	Ht	Wt	BMI
37.0°C (98.6°F)	90/min	20/min	115/70 mm Hg (lying down) 95/55 mm Hg (after standing for 5 min)	99% on RA	155 cm (5 ft 1 in) 25th %ile	42 kg (93 lb) 10th %ile	17.5 kg/m^2 25th %ile

- Appearance: sitting in a chair; comfortable, not in acute distress
- Skin: no rashes
- Pulmonary: clear to auscultation
- Cardiac: regular rhythm; no murmurs; capillary refill time of <2 seconds
- Abdominal: nondistended; normoactive bowel sounds; soft, nontender
- Neurologic: alert; cranial nerves intact

Question: An adverse effect of which of the following medications is the most likely cause of this patient's current condition?

(A) Albuterol
(B) Clonidine
(C) Methylphenidate
(D) Prednisone
(E) Rifampin

46. A 51-year-old woman comes to the office for a health maintenance examination. She feels well. She has no history of serious illness and takes no medications. She smoked one pack of cigarettes daily for 28 years but quit 6 years ago. Pulse is 80/min, respirations are 12/min, and blood pressure is 122/78 mm Hg. On examination, pedal pulses are absent bilaterally. A femoral bruit is heard in the left lower extremity. Which of the following is the most appropriate next step in management?

(A) Pentoxifylline therapy
(B) Peripheral artery catheterization
(C) Serum lipid studies
(D) Warfarin therapy
(E) No further management is indicated

47. A 47-year-old woman comes to the office for a follow-up examination. Three days ago, she was brought to the emergency department because of cellulitis of her right lower extremity, and trimethoprim-sulfamethoxazole therapy was begun. She has had no adverse reaction to the medication. She has kept her leg elevated, and her cellulitis has improved moderately. She also has hypertension, gout, and congenital heart disease. Her other medications are lisinopril, allopurinol, and warfarin. Five years ago, she underwent mechanical mitral valve replacement. Temperature today is 36.7°C (98.0°F), pulse is 72/min, and blood pressure is 128/83 mm Hg. Examination shows a 4 × 5-cm, erythematous, warm rash over the right lower extremity. There is no red streaking, vesicles, or purulence. There is no rash elsewhere. Pulses in the lower extremities are intact. Lungs are clear to auscultation. On cardiac examination, a mechanical click is heard during S_1. A grade 2/6 holosystolic murmur is heard best at the apex. The remainder of the examination shows no abnormalities. Results of laboratory studies are shown:

Blood	
Hematocrit	35%
WBC	13,000/mm^3
Platelet count	357,000/mm^3
PTT	38 sec
PT	57 sec (INR=5.2)

Which of the following actions is most likely to have prevented the near miss in this patient?

(A) Review for potential drug-drug interaction
(B) Dietary counseling
(C) Echocardiography
(D) Venous duplex ultrasonography of the right lower extremity
(E) Discontinuation of allopurinol

48. A 58-year-old woman comes to the office for a routine examination. She has type 2 diabetes mellitus. During the past 3 months, her fingerstick blood glucose concentrations have ranged from 80 mg/dL to 230 mg/dL; previously, they ranged from 80 mg/dL to 140 mg/dL. Medications are metformin and aspirin. During the past 3 months, she has exercised three times weekly; previously, she exercised once weekly. She follows a balanced diet. Despite exercise and diet, she has not had weight loss. She is 157 cm (5 ft 2 in) tall and weighs 72 kg (160 lb); BMI is 29 kg/m^2. Examination shows a 1 × 2-cm, erythematous, soft lesion over the posterior plantar aspect of the left foot. Sensation to pinprick and touch is decreased over the ankles and feet. Hemoglobin A_{1c} is 7%. Which of the following findings are most likely to be present in this patient?

	**Glucose**	**Insulin**	**Insulin Receptor Responsiveness**
(A)	Increased	increased	decreased
(B)	Increased	increased	normal
(C)	Increased	normal	decreased
(D)	Increased	normal	normal
(E)	Normal	normal	normal

49. A 57-year-old woman comes to the emergency department because of a 2-day history of fever, chills, and sore throat. She is on day 6 of a 7-day course of trimethoprim-sulfamethoxazole for treatment of cystitis. She has been taking lisinopril nightly for 2 years for treatment of hypertension. She has no other history of serious illness and takes no other medications. Temperature is 38.6°C (101.4°F), pulse is 84/min, respirations are 16/min, and blood pressure is 118/76 mm Hg. Examination shows erythema of the pharynx but no exudates. No other abnormalities are noted. Results of laboratory studies are shown:

Blood	
Hematocrit	38%
WBC	2200/mm^3
Neutrophils, segmented	16%
Neutrophils, bands	4%
Lymphocytes	64%
Monocytes	16%
Platelet count	225,000/mm^3

Which of the following is the most likely explanation for these findings?

(A) Acute mononucleosis
(B) Acute myelogenous leukemia
(C) Adverse effect of trimethoprim-sulfamethoxazole
(D) Allergic reaction to lisinopril
(E) Myelofibrosis
(F) Sepsis syndrome

50. A 39-year-old man is brought to the emergency department because of a 3-hour history of moderate pain and swelling of his right knee and ankle. His symptoms began after he scaled a wall during an obstacle course. He landed awkwardly on his right leg and rolled his right ankle; his knee buckled. He limped to the finish line. He underwent reconstruction of a torn right anterior cruciate ligament 15 years ago. He and his boyfriend are engaged to be married. He feels safe at home. He does not appear to be in distress. Examination of the right knee shows mild tenderness to palpation of the midsagittal joint line. Adduction and abduction stress testing is stable at 0 and 30 degrees of flexion. Range of motion is full. Lachman test is negative. There is moderate tenderness to palpation of the distal aspect of the fibula. There are ecchymoses over the anterolateral aspect of the right ankle. The ankle is swollen. There is no tenderness to palpation of the medial aspect of the ankle. Range of motion of the ankle is limited to 5 degrees of dorsiflexion and 30 degrees of plantar flexion because of pain. Which of the following is the most appropriate next step in diagnosis?

	**Knee**	**Ankle**
(A)	X-ray	x-ray
(B)	X-ray	no diagnostic testing indicated
(C)	No diagnostic testing indicated	x-ray
(D)	No diagnostic testing indicated	no diagnostic testing indicated

51. A 13-year-old girl is brought to the office for evaluation of short stature. Since birth, she has been below the 3rd percentile for height and at the 3rd percentile for weight. She has never had a menstrual period. There is no family history of short stature, but her maternal uncle did not begin puberty until the age of 16 years. Her current height is consistent with that of a 9½-year-old girl. Pulse is 82/min and blood pressure is 110/70 mm Hg. Sexual maturity rating is stage 1 for breast development and stage 2 for pubic hair development. Which of the following is the most appropriate next step in diagnosis?

(A) Complete blood count and serum chemistry panel
(B) Measurement of urine free cortisol concentration
(C) X-rays of the left hand and wrist
(D) X-rays of the long bones and spine
(E) X-ray of the skull

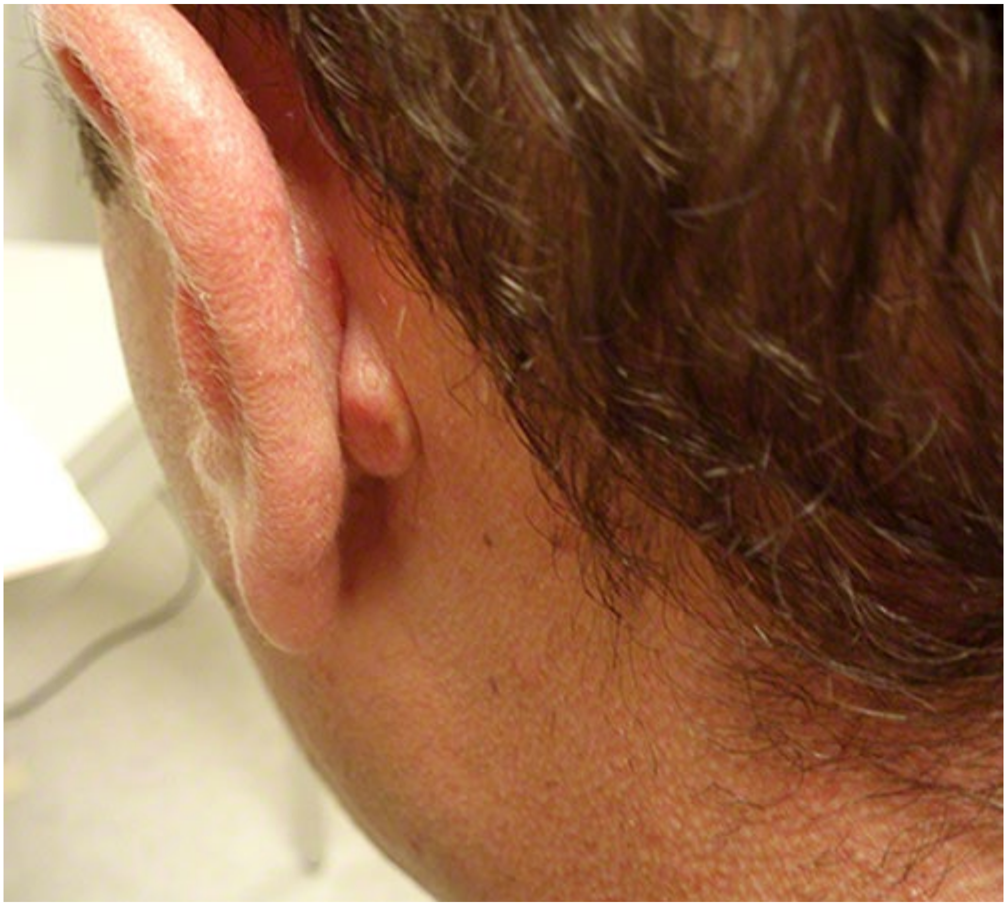

52. A 20-year-old woman comes to the office because of a 1-year history of a lump on her left ear that appeared 2 months after she had the ear pierced. Physical examination shows the findings in the photograph. Which of the following is the most appropriate initial treatment for this patient?

(A) Corticosteroid injection
(B) Cryotherapy
(C) Laser ablation
(D) Needle aspiration
(E) Wide excision

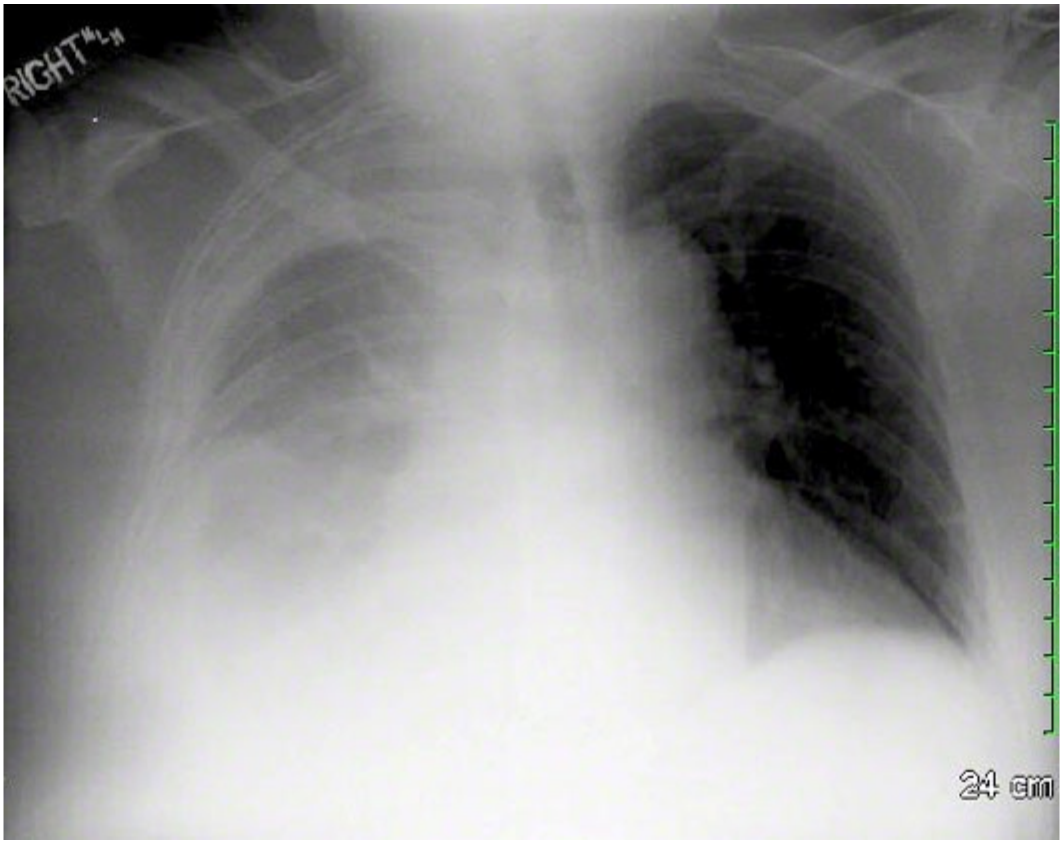

53. A 42-year-old woman is brought to the emergency department by ambulance 20 minutes after a head-on motor vehicle collision in which she was the restrained driver. On arrival, she is alert and reports chest and abdominal pain. Medical history is unremarkable and she takes no medications. Temperature is 36.7°C (98.0°F), pulse is 120/min, respirations are 28/min, and blood pressure is 130/80 mm Hg. Pulse oximetry on 4 L/min of oxygen via nasal cannula shows an oxygen saturation of 90%. Breath sounds are decreased on the right. Trachea is in the midline position. Cardiac examination discloses no abnormalities. An abrasion consistent in shape with a seat belt is present over the right upper abdominal quadrant; the area is tender to palpation. Radial and dorsalis pedis pulses are normal bilaterally. Results of laboratory studies are shown:

Arterial blood gas analysis on oxygen at 4 L/min	
PO_2	65 mm Hg
PCO_2	32 mm Hg
pH	7.38
Blood	
Hemoglobin	10.0 g/dL

Chest x-ray is shown. In addition to intravenous analgesic therapy, which of the following is the most appropriate next step in management?

(A) Bronchoscopy
(B) Intubation and mechanical ventilation
(C) Placement of a thoracic epidural
(D) Thoracentesis
(E) Thoracotomy
(F) Tube thoracostomy
(G) Observation only

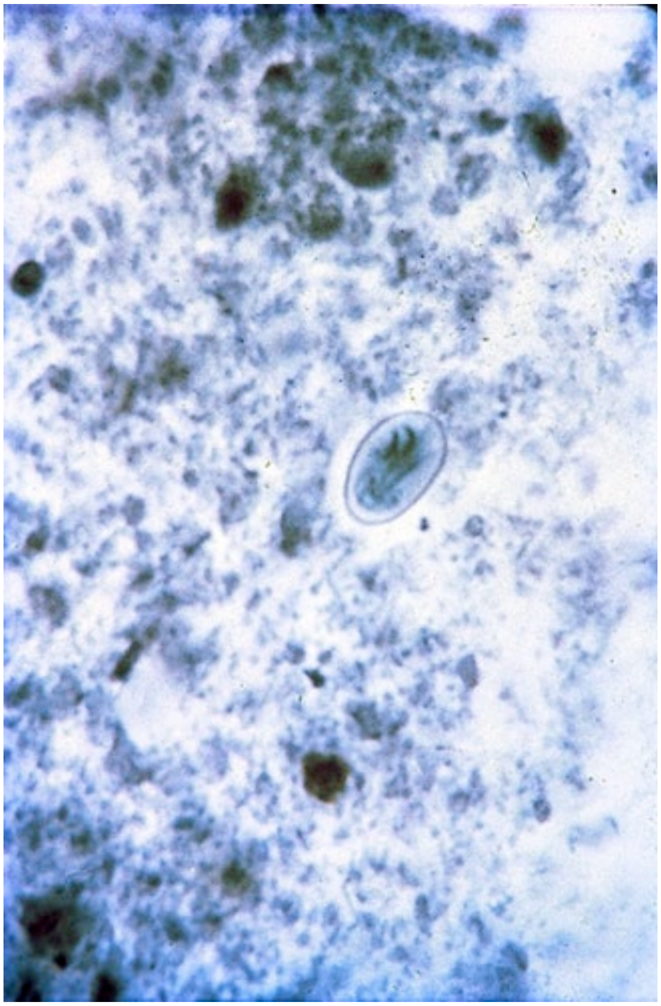

54. A 31-year-old woman comes to the office because of a 3-month history of intermittent nonbloody diarrhea. The first episode began 5 days after a camping trip in rural Virginia with her family. During the trip, she drank from a spring-fed pond that she says was “clean.” During the past 3 weeks, she also has had a 2.3-kg (5-lb) weight loss despite no change in appetite. She has not had fever or other gastrointestinal symptoms. She has no history of serious illness and takes no medications. She is sexually active with one female partner. The patient is 163 cm (5 ft 4 in) tall and weighs 54 kg (119 lb); BMI is 20 kg/m^2. Temperature is 37.9°C (100.2°F), pulse is 85/min and regular, respirations are 12/min, and blood pressure is 115/70 mm Hg. Abdominal examination shows mild, diffuse tenderness to deep palpation; bowel sounds are increased. Test of the stool for occult blood is negative. Results of a stool smear are shown. Which of the following is the most likely infectious agent?

(A) *Campylobacter jejuni*
(B) *Clostridioides difficile*
(C) *Entamoeba histolytica*
(D) *Escherichia coli*
(E) *Giardia lamblia*
(F) *Salmonella enteritidis*
(G) *Shigella dysenteriae*

55. A randomized placebo-controlled clinical trial is conducted to assess the effectiveness of a selective estrogen receptor modulator (SERM) for chemoprophylaxis of breast cancer. A subset of the results shows:

	SERM	Placebo
Number of participants	2400	1200
Woman-years of follow-up	7200	3600
Invasive breast cancer	7	13
Breast cancer rate (per 1000 woman-years)	1	3.6
Estrogen receptor status (invasive cancers)		
Positive	2	10
Negative	4	2
Unknown	1	1

Which of the following represents the absolute risk reduction for invasive breast cancer per 1000 woman-years in those treated with a SERM?

(A) 0.28
(B) 2.6
(C) 3.6
(D) 6
(E) 7

Items #56–57 are part of a sequential item set. In the actual examination environment, you will not be able to view the second item until you click "Proceed to Next Item." After navigating to the second item, you will not be able to add or change an answer to the first item.

A 57-year-old woman comes to the emergency department 1 hour after vomiting bright red blood. She thinks it was approximately 1 cup of blood. She reports that during the past month, she has felt full sooner than usual while eating. She has not had other episodes of vomiting and has not had weight loss. She has no history of operative procedures. She has hypertension and type 2 diabetes mellitus. Medications are hydrochlorothiazide and metformin. She has not taken aspirin or nonsteroidal anti-inflammatory drugs during the past month. She does not smoke cigarettes, drink alcohol, or use other substances. Temperature is 37.0°C (98.6°F), pulse is 116/min, respirations are 16/min, and blood pressure is 110/78 mm Hg. Examination shows mild tenderness to palpation in the midepigastric area; there is no rebound tenderness. Test of the stool for occult blood is positive. Hematocrit is 31%, platelet count is 240,000/mm^3, and prothrombin time is 13 seconds (INR=1.2).

56. In addition to intravenous administration of fluids and nasogastric tube lavage, which of the following is the most appropriate next step in management?

(A) Abdominal CT scan
(B) Octreotide scan
(C) Technetium 99m scan
(D) Colonoscopy
(E) Esophagogastroduodenoscopy
(F) Mesenteric angiography

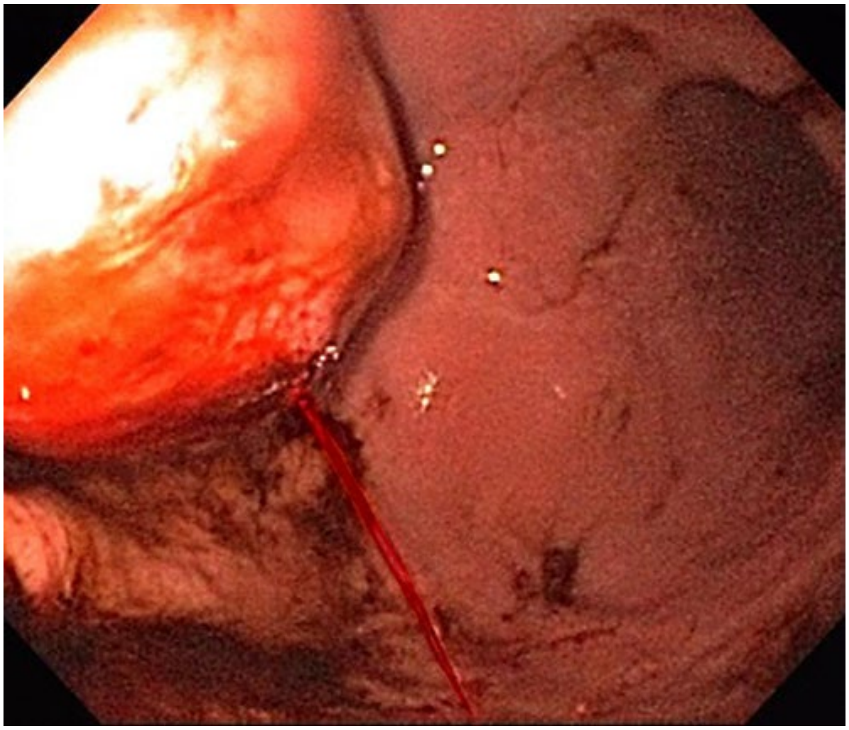

57. After intravenous administration of fluids and nasogastric tube lavage, the patient undergoes esophagogastroduodenoscopy. A photograph of the endoscopic findings is shown. Which of the following is the most appropriate next step in management?

(A) Octreotide therapy
(B) Omeprazole therapy
(C) Tyrosine kinase inhibitor therapy
(D) Endoscopic hemostatic therapy
(E) Endoscopic biopsy
(F) Endoscopic resection

END OF SET

---

58. A 77-year-old woman who resides in a skilled nursing care facility is examined by the physician 1 day after staff noticed some blood on her bedsheets. She sustained a cerebral infarction 8 years ago and has been confined to bed since then because of residual left hemiparesis. She is currently taking warfarin. She is 160 cm (5 ft 3 in) tall and weighs 50 kg (110 lb); BMI is 20 kg/m^2. Temperature is 36.0°C (96.8°F), pulse is 70/min, respirations are 18/min, and blood pressure is 180/90 mm Hg. There is no lymphadenopathy. Examination shows a small, clean, decubitus ulcer on the sacrum. Pelvic examination shows a 3-cm, raised, fleshy lesion on the right labium majus. Vaginal examination cannot be performed because of the patient's discomfort. Which of the following is the most appropriate next step in management?

(A) Cytologic evaluation of the vulva
(B) CT scan of the abdomen and pelvis
(C) Application of an antifungal cream
(D) Application of a corticosteroid cream
(E) Colonoscopy
(F) Biopsy of the vulva

59. A 68-year-old man comes to the office because of a 3-month history of moderate low back pain that extends into both legs. He first noticed the pain while standing in the ticket line at a theater. At that time, the pain was accompanied by tingling in both legs. The symptoms recurred a few days later as he was standing in the grocery checkout line. The pain has not responded to ibuprofen or naproxen but improves with sitting or lying down. Prior to onset of the pain, the patient typically walked for 30 minutes four times weekly. He discontinued his walking regimen 2 months ago because it seemed to trigger his back pain. He has hypertension and hyperlipidemia. Medications are hydrochlorothiazide-triamterene, simvastatin, and aspirin. The patient has smoked one pack of cigarettes daily for 40 years. Pulse is 74/min and blood pressure is 140/90 mm Hg. Lungs are clear to auscultation; expiration phase is delayed. On cardiac examination, a grade 2/6 systolic crescendo-decrescendo murmur is heard at the upper right sternal border. There is trace ankle edema bilaterally; ankle brachial index is 1.0. Musculoskeletal and neurologic examinations show no abnormalities. Which of the following is the most likely diagnosis?

(A) Abdominal aortic aneurysm
(B) Osteoporosis
(C) Peripheral neuropathy
(D) Spinal stenosis
(E) Vascular claudication

60. A 50-year-old man comes to the office because of a 3-day history of mild headache and intermittent confusion. He has a 30-year history of schizophrenia and a 5-year history of emphysema. Medications are clozapine, sertraline, and inhaled beclomethasone and salmeterol. He also receives home oxygen therapy. He smoked two packs of cigarettes daily for 33 years but quit 5 years ago. Temperature is 36.8°C (98.2°F), respirations are 20/min, pulse is 90/min, and blood pressure is 115/70 mm Hg. Examination shows moist mucous membranes. Lungs are clear to auscultation. There is no peripheral edema. Neurologic examination shows no focal findings. Results of a complete blood count are within the reference ranges. Results of other laboratory studies are shown:

Serum	
Na^+	116 mEq/L
K^+	4 mEq/L
Cl^-	90 mEq/L
Urea nitrogen	10 mg/dL
Creatinine	1 mg/dL
Glucose	100 mg/dL

Which of the following is the most appropriate next step in management?

(A) CT scan of the chest
(B) Measurement of serum cortisol and TSH concentrations
(C) Measurement of serum uric acid concentration
(D) Measurement of urine and plasma osmolarity
(E) MRI of the brain

61. A 67-year-old man is evaluated in the intensive care unit. He has end-stage pancreatic cancer and was hospitalized 3 days ago for treatment of pneumonia. Respirations are 6/min. Pulse oximetry on 100% oxygen by face mask shows an oxygen saturation of 78%. Examination shows feeble respiratory efforts; he is using accessory muscles of respiration. On mental status examination, the patient is oriented to person but not to place or time. If the patient is not endotracheally intubated and mechanically ventilated, he will die within hours. His wife says the patient recently told her that he would never want mechanical ventilation, but they never completed paperwork regarding his wishes. His daughter insists that he be mechanically ventilated. Which of the following is the most appropriate action for the physician to take?

(A) Perform endotracheal intubation and begin mechanical ventilation
(B) Perform endotracheal intubation and then consult the hospital ethics committee regarding mechanical ventilation
(C) Perform endotracheal intubation only
(D) Provide palliative therapy only
(E) Seek a court order to assign a legal guardian

62. A 5-day-old boy is brought to the office for an initial well-child examination. He was born at 40 weeks' gestation and discharged at 60 hours of life. On newborn screening, hemoglobin electrophoresis showed an FS pattern. He is at the 50th percentile for length and weight. Temperature is 37.0°C (98.6°F), pulse is 136/min, and respirations are 34/min. He appears well. Examination shows no abnormalities. Which of the following is the most appropriate next step in management?

(A) Deferoxamine therapy
(B) Hydroxyurea therapy
(C) Iron supplementation
(D) Monthly blood transfusions
(E) Penicillin prophylaxis
(F) Vitamin B_{12} (cyanocobalamin) supplementation

63. A 1-week-old female newborn is brought to the office for a follow-up examination after newborn screening showed a serum TSH concentration of 40 µU/mL (N=1–20). She has been breast-feeding well. She passes five to six stools and has multiple wet diapers daily. She has returned to her birth weight. Vital signs are within normal limits. Examination discloses no abnormalities. Serum studies today show a TSH concentration of 14 µU/mL (N=0.5–6.5) and free thyroxine (FT_4) concentration of 0.4 ng/dL (N=0.9–2.2). Which of the following is the most appropriate next step in management?

(A) Hydrocortisone therapy
(B) Levothyroxine therapy
(C) Monthly serial measurement of serum thyroglobulin concentration
(D) Monthly serial measurement of serum TSH and free thyroxine (FT_4) concentrations
(E) Radioactive iodine uptake scan
(F) Ultrasonography of the thyroid gland

64. An 18-year-old patient comes to the emergency department because of a 2-day history of severe abdominal pain. The pain began in the middle of the abdomen after a large meal but now is localized to the right lower quadrant. The patient also has a 2-month history of intermittent diarrhea. Three weeks ago, the patient had an upper respiratory tract infection that resolved spontaneously. The patient currently takes no medications. The patient identifies as nonbinary and uses they/they pronouns; their assigned sex at birth was male. They do not smoke cigarettes or use other substances. Temperature is 37.8°C (100.0°F), pulse is 80/min, respirations are 20/min, and blood pressure is 110/70 mm Hg. Abdominal examination shows fullness and tenderness to palpation of the right lower quadrant. Leukocyte count is 11,000/mm^3. Which of the following is the most appropriate next step in diagnosis?

(A) Abdominal ultrasonography of the right lower quadrant
(B) Air-contrast barium enema
(C) CT scan of the abdomen
(D) Technetium 99m scan of the bowel
(E) Upper gastrointestinal series with small bowel follow-through

65. A 25-year-old woman, gravida 3, para 2, at 36 weeks' gestation comes to the emergency department because of heavy vaginal bleeding following sexual intercourse 3 hours ago. She has received no prenatal care. She has no history of serious illness or operative procedures. Vital signs are within normal limits. Continuous external fetal heart monitoring shows a baseline of 155/min with no accelerations or decelerations. The uterus is consistent in size with a 36-week gestation. Tocodynamometer monitoring shows contractions every 8 minutes. Which of the following is the most appropriate next step in management?

(A) Cesarean delivery now
(B) Digital examination of the cervix
(C) Intravenous administration of magnesium sulfate
(D) Intravenous administration of oxytocin
(E) Transabdominal ultrasonography

66. A 75-year-old man is admitted to the hospital because of a 3-day history of fever. He has type 2 diabetes mellitus, hypertension, and chronic obstructive pulmonary disease. One year ago, he had a stroke with residual right lower extremity hemiparesis. Medications are amlodipine, tiotropium, metformin, glimepiride, lisinopril, and aspirin. He does not drink alcoholic beverages and has smoked one pack of cigarettes daily for 50 years. The patient is widowed, lives alone, and has three adult children. He is not in acute distress. He is 178 cm (5 ft 10 in) tall and weighs 82 kg (180 lb); BMI is 26 kg/m^2. Temperature is 39.2°C (102.6°F), pulse is 110/min, respirations are 22/min, and blood pressure is 150/90 mm Hg. Pulse oximetry on room air shows an oxygen saturation of 96%. Scattered end-expiratory wheezes are heard. There is dense hemiparesis of the right lower extremity. Appropriate therapy is initiated, and 3 days later, the patient's condition has improved. He is awake, alert, and conversing with one of his sons about current events. Temperature is 37.2°C (99.0°F), pulse is 72/min, and blood pressure is 130/82 mm Hg. Discharge plans are discussed with the patient, his son, the physician, and a physical therapist. The physical therapist says the patient would be best cared for in a nursing care facility. The patient acknowledges that a nursing facility would provide better care for him but says he would prefer to remain at home with a visiting nurse. Once outside the patient's room, his son says, "I have his power of attorney. Don't listen to him. He needs to be in a nursing home." Which of the following is the most appropriate next step?

(A) Inform the son that the decision is the patient's to make, regardless of the medical team's recommendation
(B) Reassure the son that the patient will be admitted to a nursing care facility in accordance with the physical therapist's recommendation
(C) Refrain from making any decisions until a family meeting with the son and siblings is arranged to discuss the matter
(D) Request psychiatric evaluation of the patient's mental competency
(E) Request to review the legal documentation designating the son as having power of attorney

67. A 59-year-old man is brought to the emergency department because of a 1-hour history of weakness of his left arm and leg and mild headache. He says that, when he was gardening with his wife, he suddenly became unable to hold his gardening tool, lift his left arm, or walk. Two weeks ago, he had a nonproductive cough and nasal congestion that resolved spontaneously after 1 week. He has hypertension treated with lisinopril. On arrival, he is lethargic. He is 183 cm (6 ft) tall and weighs 97 kg (215 lb); BMI is 29 kg/m^2. Temperature is 36.6°C (97.9°F), pulse is 94/min, respirations are 16/min, and blood pressure is 174/109 mm Hg. The pupils measure 3 mm and are reactive to light. Muscle movement is decreased over the left lower aspect of the face. Muscle strength is 0/5 in the left extremities. Deep tendon reflexes are absent in the left extremities and 2+ in the right extremities. CT scan of the head shows a large area of increased attenuation in the right putamen region. Which of the following is the most likely cause of this patient's condition?

(A) Amyloid angiopathy
(B) Embolism
(C) Small vessel disease
(D) Vasculitis
(E) Venous thrombosis

68. A 24-year-old primigravid woman at 10 weeks' gestation comes to the office for a scheduled dilatation and suction curettage. Ultrasonography 2 days ago showed a fetus consistent in size with a 9-week gestation with absent cardiac activity. Two weeks ago, the patient had an upper respiratory tract infection that resolved spontaneously. One year ago, she was diagnosed with herpes simplex virus (HSV) 2; her last outbreak was 6 months ago. She has no other history of serious illness. She is 163 cm (5 ft 4 in) tall. She weighed 82 kg (180 lb) prior to her pregnancy; BMI was 31 kg/m^2. She has had a 3.6-kg (8-lb) weight gain during her pregnancy. Vital signs are within normal limits. The vulvar area is shaven; no lesions are noted. Examination shows a normal-appearing cervix. The uterus is nontender and consistent in size with an 8-week gestation. There is homogenous white-gray vaginal discharge; the pH of the discharge is 5.5. She undergoes dilatation and suction curettage with sterile instruments and povidone-iodine vaginal cleansing. Which of the following is the strongest predisposing risk factor for postoperative infection in this patient?

(A) History of upper respiratory tract infection
(B) HSV 2
(C) Obesity
(D) Type of vaginal discharge
(E) Vulvar shaving

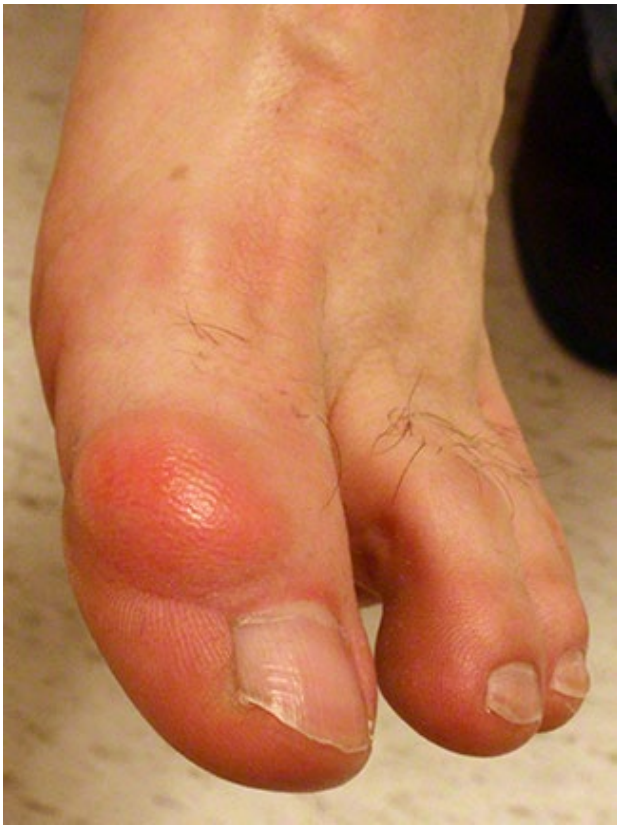

69. A 74-year-old woman comes to the clinic 2 days after the sudden onset of severe left foot pain. She has had difficulty walking during this time and has been using a cane. She has not sustained trauma to the foot. She has type 2 diabetes mellitus and hypertension. Medications are metformin, insulin glargine, hydrochlorothiazide, lisinopril, and atorvastatin. She typically wears support stockings because of lower leg swelling, but the foot pain has made it difficult for her to wear them recently. She is a traveling pastor and spends more than 4 hours daily in her car. She has been monogamous with her wife for the past 30 years. Blood pressure is 146/88 mm Hg; other vital signs are within normal limits. A photograph of the left great toe is shown. No other abnormalities are noted. Hemoglobin A_{1c} is 7.4%, serum creatinine concentration is 1.4 mg/dL, and serum uric acid concentration is 7.8 mg/dL. Results of serum electrolyte concentrations are within the reference ranges. Which of the following is the most likely diagnosis?

(A) Cellulitis
(B) Gout
(C) Infected mucoid cyst
(D) Osteoarthritis
(E) Pseudogout
(F) Rheumatoid arthritis

---

70. A 47-year-old man comes to the office for a routine examination before beginning an exercise program. He feels well. He has a 25-year history of type 1 diabetes mellitus. A grade 1/6, early peaking, systolic ejection murmur is heard best at the left third intercostal space. Sensation to light touch is mildly decreased over the toes. The remainder of the examination shows no abnormalities. An exercise stress test shows a 3-mm ST-segment depression early in the test. The test does not produce any pain. Which of the following is the most likely diagnosis?

(A) Cardiomyopathy
(B) Congenital heart disease
(C) Coronary artery disease
(D) Valvular heart disease
(E) Normal cardiac findings

71. A 22-year-old man comes to the office for a health maintenance examination. He feels well and has not noticed any health issues. He is in the US Army. Two months ago, he returned from a 10-month deployment to Southwest Asia, during which he started smoking one pack of cigarettes daily. The patient says he is happy to be home and has been celebrating with his friends and family. He has been consuming an average of six beers daily since returning to the United States. He has no history of major medical illness. He does not use any medications or other substances. His mother has type 2 diabetes mellitus. The patient is 180 cm (5 ft 11 in) tall and weighs 72 kg (160 lb); BMI is 22 kg/m^2. His first blood pressure reading is 156/95 mm Hg; repeat reading is 160/92 mm Hg. He says he remembers his blood pressure being normal when he had it checked in the past. Physical examination discloses no other abnormalities. Which of the following is the most likely cause of this patient's increased blood pressure?

(A) Cigarette smoking
(B) Essential hypertension
(C) Excessive alcohol use
(D) Pheochromocytoma
(E) Renal artery stenosis

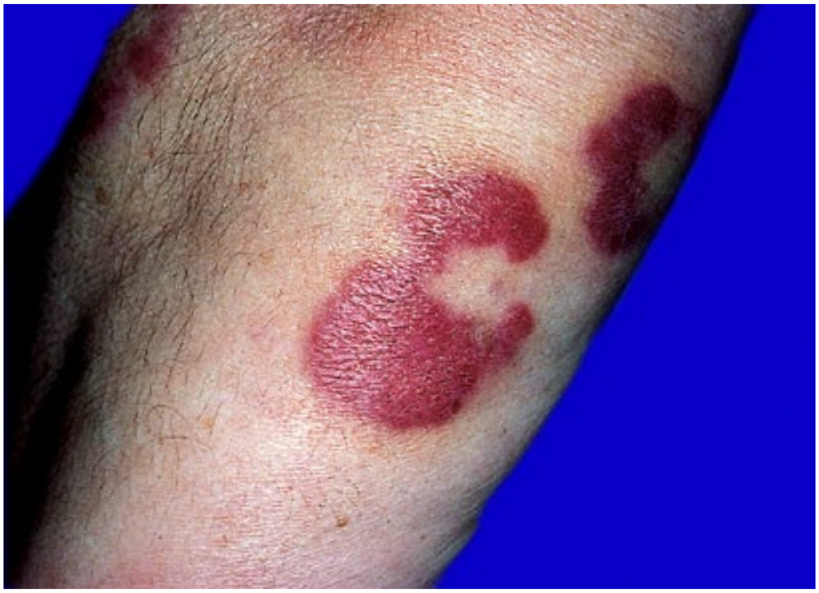

72. A 32-year-old man comes to the office because of a 3-month history of a painless rash over his arms. During the past month, he also has had a 3.2-kg (7-lb) weight loss despite no change in appetite. He has no history of serious illness and takes no medications. He is 175 cm (5 ft 9 in) tall and weighs 72 kg (158 lb); BMI is 23 kg/m^2. Pulse is 98/min; other vital signs are within normal limits. Lungs are clear to auscultation. Cardiac examination shows no abnormalities. A photograph of the left upper extremity is shown. Similar findings are noted over the right upper extremity. Which of the following is the most likely cause of this patient's dermatologic findings?

(A) Actinic keratosis
(B) Dermatitis herpetiformis
(C) Kaposi sarcoma
(D) Lichen planus
(E) Seborrheic dermatitis

73. A 42-year-old nulligravid woman comes to the office because of a 6-month history of hot flashes and intermittent palpitations. Her last menstrual period was 6 months ago. Menses previously occurred at regular 29-day intervals but gradually had occurred less frequently during the past 2 years. She underwent ovarian cystectomy 13 years ago for a small benign teratoma. She used an oral contraceptive for 12 years until tubal ligation 6 years ago. Her mother and sister had menopause at the ages of 47 and 49 years, respectively. The patient has smoked one pack of cigarettes daily for 26 years. She is sexually active and monogamous with one male partner. She is 170 cm (5 ft 7 in) tall and weighs 86 kg (191 lb); BMI is 30 kg/m^2. Pulse is 82/min and blood pressure is 130/85 mm Hg. Physical examination, including pelvic examination, discloses no abnormalities. Which of the following is the most appropriate next step in diagnosis?

(A) Measurement of serum follicle-stimulating hormone concentration
(B) Measurement of serum luteinizing hormone concentration
(C) Measurement of serum testosterone concentration
(D) ECG
(E) Pelvic ultrasonography

74. A colleague of a 47-year-old physician who is conducting patient evaluations during afternoon walk-in clinic hours notices that the physician appears groggy, has slurred speech, and smells of alcohol. Earlier in the day, the physician had attended a celebratory lunch for the graduation of one of the clinic's employees from nursing school. The colleague has not witnessed similar findings in the physician in the past. The physician is scheduled to work at the clinic for the next 4 hours. A physician assistant is present at the clinic and typically aids the physician with evaluating patients. Which of the following is the most appropriate action for the colleague to take at this time?

(A) Allow the physician to finish his time at the clinic, but raise concern about the episode at the next physicians' staff meeting
(B) Ask the office administrator to cancel the remainder of the clinic hours
(C) Ask the physician assistant to examine the physician's patients and discharge them before the physician has a chance to examine them
(D) Confront the physician and ask him to leave the clinic
(E) Report the physician to the clinic administrator and conduct the remainder of the clinic hours without him
(F) Report the physician to the state medical board

75. A 68-year-old woman returns to the office for a follow-up examination. One month ago, urinalysis showed protein during work-up of a suspected urinary tract infection. At that time, the patient was prescribed a 3-day course of oral cephalexin. Today, she says her previous pain on urination and urinary frequency have resolved. She has major depressive disorder and osteoarthritis. Current medications are venlafaxine and acetaminophen. Temperature is 37.0°C (98.6°F), pulse is 66/min, respirations are 18/min, and blood pressure is 122/66 mm Hg. The patient appears physically fit. Examination discloses no abnormalities. Repeat urinalysis is shown:

Specific gravity	1.015 (N=1.003–1.029)
pH	6.0 (N=4.5–7.8)
Protein	negative
Blood	negative
Leukocyte esterase	negative
Nitrite	negative
WBCs	3/hpf
RBCs	1/hpf

Which of the following is the most appropriate additional diagnostic study at this time?

(A) Determination of urine albumin:creatinine ratio
(B) 24-Hour urine collection for measurement of protein concentration
(C) Ultrasonography of the kidneys
(D) Urine sulfosalicylic acid method
(E) No additional testing is indicated

76. A 22-year-old man is brought to the emergency department because of severe right lower leg pain since he fell in the woods during a camping trip 2 days ago. The pain has worsened during the past 12 hours. He sustained a small scratch below his knee as a result of the fall. He has no history of serious illness and uses no medications or other substances. He does not drink alcohol. Pulse is 110/min, respirations are 14/min, and blood pressure is 105/60 mm Hg. Examination of the right lower extremity shows edema, diffuse cellulitis, and hemorrhagic bullae from the knee to the ankle. No abnormalities of the left lower extremity are noted. Peripheral intravenous antibiotic therapy and infusion of 2 L of crystalloid fluid are begun. Which of the following is the most appropriate next step in management?

(A) Fine-needle aspiration of the bullae
(B) Hyperbaric oxygen therapy
(C) MRI of the right lower extremity
(D) Surgical debridement
(E) Hospital admission for observation only

77. A 27-year-old woman, gravida 3, para 2, at 12 weeks' gestation comes to the office for her first prenatal visit. Her first child was delivered at 41 weeks' gestation and weighed 4167 g (9 lb 3 oz). Her second child was delivered at 37 weeks' gestation and weighed 4309 g (9 lb 8 oz). She has no history of serious illness and her only medication is a prenatal vitamin. Her sister has a history of delivering a stillborn infant. Her mother and father both have type 2 diabetes mellitus. Eight months ago, the patient immigrated to the United States from Mexico. The patient is 157 cm (5 ft 2 in) tall. She weighed 95 kg (210 lb) prior to her pregnancy; BMI was 38 kg/m^2. She has had a 3-kg (6.6-lb) weight gain during her pregnancy. Pulse is 80/min, respirations are 14/min, and blood pressure is 110/60 mm Hg. Bimanual examination shows a uterus consistent in size with a 12-week gestation. Ultrasonography confirms a 12-week gestation. Which of the following is the most appropriate next step in management?

(A) Antiphospholipid antibody screening
(B) Chest x-ray
(C) Fasting glucose tolerance test
(D) Measurement of serum α-fetoprotein concentration
(E) Measurement of serum free thyroxine concentration

78. An 84-year-old woman comes to the office because of a 1-month history of malaise. She has not had fever, weight loss, or abdominal pain. She has type 2 diabetes mellitus well controlled with long-acting insulin and metformin. She has missed multiple appointments during the past 6 months with no reasons given. Her hemoglobin A_{1c} 6 months ago was 6.7%. She says she has been “feeling fine.” Her son moved back into her home 9 months ago because he had lost his job. The patient’s husband died 5 years ago, and she has no other children. She appears unkempt. She is alert and fully oriented. She is 163 cm (5 ft 4 in) tall and weighs 77 kg (170 lb); BMI is 29 kg/m^2. Her pulse is 80/min and regular, respirations are 18/min, and blood pressure is 160/94 mm Hg. Mucous membranes are dry; there is no thrush. The skin is dry and intact. The remainder of the physical examination shows no abnormalities. Mental status examination shows no memory deficit. She has a normal mood and flat affect. Her fasting serum glucose concentration is 380 mg/dL. Results of urinalysis are shown:

Glucose	4+
Ketones	none
Nitrites	none
Leukocyte esterase	none

Which of the following is the most likely diagnosis?

(A) Dietary indiscretion
(B) Elder neglect
(C) Major depressive disorder
(D) Pancreatic malignancy
(E) Urinary tract infection

79. A 15-year-old boy is brought to the office 2 hours after he awoke with weakness of the right side of his face. He has a 2-week history of intermittent headache, sensitivity to light, and mild to moderate neck pain. He has no history of serious illness. His only medication is acetaminophen as needed. He lives in New Jersey and has not traveled outside of the state during the past 6 months. Temperature is 37.4°C (99.3°F), pulse is 78/min and regular, and blood pressure is 108/70 mm Hg. Flexion of the neck elicits moderate pain and resistance. The right eyelids do not close completely. Ocular movements are normal. There is no evidence of ptosis. The right side of the forehead does not wrinkle when the patient looks up. There is severe weakness of the right corner of the mouth. There are no abnormalities of the left side of the face. Muscle strength in the jaw, tongue, and palate is normal and symmetric. There is no dysarthria. Muscle strength is 5/5 in all extremities. Coordination and sensation are intact. Gait is normal. A lumbar puncture is performed, and opening pressure is 180 mm H_2O. Cerebrospinal fluid analysis results are shown:

Glucose	60 mg/dL
Total protein	52 mg/dL
WBC	85/mm^3
Segmented neutrophils	30%
Lymphocytes	70%
RBC	1/mm^3

Which of the following is the most appropriate next step in diagnosis?

(A) Measurement of serum angiotensin-converting enzyme activity
(B) Measurement of serum Lyme (*Borrelia burgdorferi*) antibody concentration
(C) Polymerase chain reaction test for cytomegalovirus
(D) Serum antinuclear antibody assay
(E) Serum protein electrophoresis

80. A 2-year-old girl is brought to the emergency department because of loud breathing, harsh cough, and a hoarse cry since she awoke 1 hour ago. Her parents state that yesterday she had a runny nose. Medical history is unremarkable and she receives no medications. Temperature is 38.5°C (101.3°F), pulse is 120/min, respirations are 32/min, and blood pressure is 105/65 mm Hg. There are moderate suprasternal and subcostal retractions. Inspiratory stridor is heard at rest. Which of the following is the most appropriate initial pharmacotherapy?

(A) Intravenous dexamethasone
(B) Nebulized albuterol
(C) Nebulized budesonide
(D) Nebulized epinephrine
(E) Oral albuterol
(F) Oral prednisone
(G) Subcutaneous epinephrine

BLOCK 3, ITEMS 81–120

81. A 28-year-old man is admitted to the intensive care unit 3 hours after undergoing resection and anastomosis of a small-bowel injury and nephrectomy because of a right renal laceration sustained in a motor vehicle collision. He also has bilateral, minimally displaced pubic rami fractures. He is intubated and mechanically ventilated. He is receiving morphine and propofol. He responds only to painful stimuli. Pulse is 110/min, ventilatory rate is 12/min, and blood pressure is 133/68 mm Hg. The abdomen is distended and soft, and the incision is clean, dry, and intact. Since the operation, urine output had been 40 mL/h and bloody. During the past hour, urine output has been 5 mL and bloody. In addition to intravenous administration of 0.9% saline, which of the following is the most appropriate next step in management?

(A) CT scan of the abdomen and pelvis
(B) Repeat surgical exploration of the abdomen
(C) Transfusion of packed red blood cells
(D) Ultrasonography of the bladder

82. A 15-year-old girl is brought to the office because of a 3-day history of diffuse abdominal pain, fever, and vomiting. She has cerebral palsy, situs inversus, and severe scoliosis. She uses a wheelchair for ambulation. During previous visits, transferring this patient from her wheelchair to the examination table has been time-consuming and has caused the patient discomfort. Today, the patient appears uncomfortable. The physician decides to conduct the examination while the patient is sitting in her wheelchair. Temperature is 38.3°C (101.0°F), pulse is 110/min, respirations are 20/min, and blood pressure is 110/50 mm Hg. Pulse oximetry on room air shows an oxygen saturation of 100%. Examination shows mildly dry mucous membranes. The physician evaluated four previous patients today who had vomiting and fever; he diagnosed gastroenteritis in all four patients. The physician attributes this patient's increased pulse to mild dehydration and fever, and he diagnoses this patient with gastroenteritis. The physician prescribes ondansetron and tells the patient to return if she has intractable vomiting or if her abdominal pain worsens. Five hours later, the patient is taken to the emergency department after her mother finds her listless and barely responsive. Temperature is 38.9°C (102.0°F), pulse is 180/min, respirations are 35/min, and blood pressure is 70/50 mm Hg. Pulse oximetry on room air shows an oxygen saturation of 96%. CT scan of the abdomen shows volvulus, and emergency resection of necrotic bowel is done. Which of the following best describes the error that occurred?

(A) Latent error
(B) Near miss
(C) Non-preventable error
(D) Premature closure
(E) Systems failure

83. A 27-year-old man comes to the office because of a 1-month history of depressed mood and fatigue. He reports that it takes him at least 1 hour to fall asleep at night. He has had decreased concentration at work and decreased interest in socializing with his friends. He no longer exercises. He has epilepsy well controlled with levetiracetam. Physical examination discloses no abnormalities. On mental status examination, he has a sad mood and is briefly tearful. He has no suicidal ideation, hallucinations, or delusions. Which of the following is the most appropriate pharmacotherapy?

(A) Alprazolam
(B) Aripiprazole
(C) Bupropion
(D) Buspirone
(E) Mirtazapine

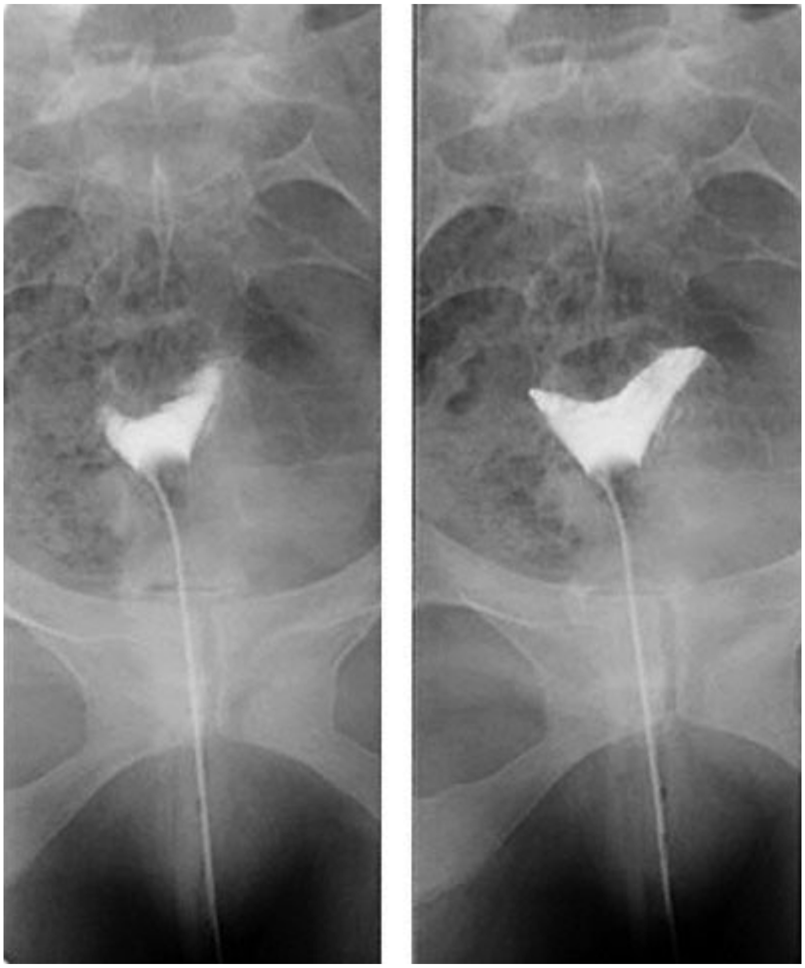

84. A 23-year-old nulligravid woman comes to the office because she has been unable to conceive during the past year. She and her husband have had regular unprotected intercourse during this time. The husband has no children. Prior to attempting to conceive, the patient regularly used depot medroxyprogesterone. Menses occur at regular 28-day intervals with occasional mid-cycle abdominal pain previously diagnosed as mittelschmerz. She had a ruptured appendix at age 17 years and pelvic inflammatory disease at age 19 years. At age 21 years, cervical cytology showed a low-grade squamous intraepithelial lesion; repeat cervical cytology 1 year later showed no abnormalities. Current medications are a prenatal vitamin and occasional ibuprofen for mid-cycle abdominal pain. She is 168 cm (5 ft 6 in) tall and weighs 59 kg (130 lb); BMI is 21 kg/m^2. Vital signs are within normal limits. Physical examination discloses no abnormalities. Hysterosalpingography is done, and x-rays obtained during the procedure are shown. Without treatment, which of the following is the most likely clinical course for this patient?

(A) Female factor infertility
(B) Hydatidiform mole
(C) Recurrent spontaneous abortions
(D) Successful pregnancy within the next year

---

85. A 50-year-old woman comes to the office for a health maintenance examination. She feels well. Medical history is unremarkable and she takes no medications. There is no family history of serious illness. Physical examination, including breast examination, discloses no abnormalities. Mammography shows a cluster of 20 pleomorphic microcalcifications in a 1-cm area at the 2-o'clock position of the right breast. Which of the following is the most likely diagnosis?

(A) Ductal carcinoma in situ
(B) Fat necrosis
(C) Fibroadenoma
(D) Mastitis
(E) Sclerosing adenosis

86. A community has created standard-of-care guidelines for ambulatory patients with several common diagnoses. The community's standard of care for patients with asthma is annual pulmonary function testing. Recent analysis of billing records from one clinic in this community shows that only 34% of patients with asthma at the clinic undergo pulmonary function testing. Enacting which of the following procedures is most likely to improve this clinic's adherence to the community's standard of care for patients with asthma?

(A) Annual chart reviews of patients with asthma and feedback to the physicians
(B) Diagnosis-driven reminders in patient charts
(C) Placement of flyers in the clinic waiting room that remind patients with asthma to undergo annual pulmonary function testing
(D) Pulmonary function testing of all patients examined for respiratory symptoms
(E) Pulmonary function testing of patients with asthma at each examination preceded by an exacerbation

87. A 27-year-old woman, gravida 1, para 1, comes to the emergency department because of a 1-day history of moderate pain in her right breast. Three weeks ago, she underwent cesarean delivery of a healthy newborn at term because of breech presentation. She is breast-feeding, but the newborn is not latching properly on the right. She has no history of serious illness. Medications are acetaminophen for postoperative pain and a prenatal vitamin. Temperature is 38.9°C (102.0°F). Examination of the right breast shows a 5 × 2-cm wedge-shaped area of erythema and moderate tenderness. The left breast is normal. Abdominal examination shows a well-healing surgical incision. In addition to beginning antipyretic therapy, which of the following is the most appropriate next step in management?

(A) Application of a breast binder on the right
(B) Dicloxacillin therapy
(C) Fluconazole therapy
(D) Fine-needle aspiration of the erythematous area
(E) No further management is indicated

88. A 12-year-old girl is brought to the office for a well-child examination. She feels well. She has no history of serious illness and receives no medications. Menarche has not yet occurred. She is at the 30th percentile for height and 60th percentile for weight. Examination of the breasts shows no glandular tissue; the areolae follow the skin contours of the chest. There is no pubic hair. The remainder of the examination shows no abnormalities. Which of the following is the most appropriate next step in diagnosis?

(A) Measurement of serum follicle-stimulating hormone and luteinizing hormone concentrations
(B) Measurement of serum growth hormone and thyroxine concentrations
(C) MRI of the brain
(D) X-ray of the left hand and wrist to determine bone age
(E) No additional diagnostic steps are indicated

89. A 42-year-old woman comes to the office because of a 2-month history of episodes of light-headedness and loss of consciousness for 10 seconds. The episodes occur two to three times weekly at work. She says her light-headedness resolves after she eats snacks. During this time, she also has had a 9-kg (20-lb) weight gain. She has no history of serious illness and takes no medications. She is 160 cm (5 ft 3 in) tall and weighs 72 kg (160 lb); BMI is 28 kg/m^2. Vital signs are within normal limits. Physical examination, including neurologic examination, shows no abnormalities. Serum glucose concentration is 41 mg/dL, serum C peptide concentration is 0.5 ng/mL (N=0.5–2.5), and serum insulin concentration is 80 μIU/mL (N=5–20). CT scan of the abdomen is most likely to show which of the following findings?

(A) Duodenal mass with multiple liver metastases
(B) Fluid-filled mass between the posterior aspect of the stomach and pancreas
(C) Low-density mass in the head of the pancreas obstructing the main duct
(D) Vascular mass in the neck of the pancreas
(E) No abnormalities

90. A hospital with a large intensive care unit (ICU) would like to improve communication among team members who care for patients with complex conditions. Research has shown that team communication is most effective when one clinician is designated as the team leader and assumes responsibility for directing the care. The team leader ensures that all team members share a common understanding of the patient and openly discuss their views about the case. The common understanding includes the patient's diagnosis, prognosis, and care plan. This allows the team to quickly recognize when new data deviate from the expected and then reassess the team's approach to the patient. Which of the following is the most appropriate method for maintaining a common mental model among all team members?

(A) Conduct weekly structured team briefings and daily huddles
(B) Educate all team members on protocols for common illnesses
(C) Have the team leader discuss the case with each team member individually on a regular basis
(D) Maintain a consistent team of intensivists in the ICU
(E) Maintain a disease-specific ICU

91. A 12-month-old girl is brought to the office by her father because of a 3-day history of fever and mildly decreased appetite and activity. She has not had cough, vomiting, or diarrhea. She receives no medications. Vaccinations are up-to-date. She is at the 35th percentile for height, 50th percentile for weight, and 60th percentile for head circumference. Temperature is 39.2°C (102.6°F); other vital signs are within normal limits. Examination shows no other abnormalities. A catheter urine sample is obtained, and urinalysis shows 20–30 WBC/hpf and is positive for nitrites. Oral trimethoprim-sulfamethoxazole therapy is begun. The next day, urine culture grows greater than 100,000 colonies/mL of *Escherichia coli* that is susceptible to trimethoprim-sulfamethoxazole. Which of the following is the most appropriate next step in management?

(A) Ciprofloxacin prophylaxis
(B) Clindamycin prophylaxis
(C) CT urography
(D) Ultrasonography of the kidneys and bladder
(E) Voiding cystourethrography

92. A 32-year-old man comes to the office because of a 2-week history of persistent nonproductive cough occurring in short bursts. During the past week, he has had bilateral chest pain with severe coughing. He has vomited several times after coughing vigorously. He has not had shortness of breath or fever. Three weeks ago, he had a runny nose, sneezing, and mild malaise that resolved spontaneously after 1 week. Toward the end of that period, he noticed the onset of cough, which has since worsened. He has no history of serious illness, and his only medication is over-the-counter cough drops. He drinks 24 to 36 oz of beer weekly. He does not smoke cigarettes or use other substances. He is sexually active with one male partner; they use condoms consistently. He is 173 cm (5 ft 8 in) tall and weighs 86 kg (190 lb); BMI is 29 kg/m^2. Temperature is 37.0°C (98.6°F), pulse is 70/min, respirations are 12/min, and blood pressure is 120/80 mm Hg. Pulse oximetry on room air shows an oxygen saturation of 98%. During the examination, the patient coughs several times. There is no sinus tenderness to palpation and no cervical lymphadenopathy. The oropharynx appears normal. Lungs are clear to auscultation. Cardiac examination discloses no abnormalities. Which of the following is the most appropriate pharmacotherapy?

(A) Albuterol
(B) Azithromycin
(C) Levofloxacin
(D) Omeprazole
(E) Prednisone

93. **Patient Information**

Age: 18 years
Gender: F, self-identified
Race/Ethnicity: unspecified
Site of Care: emergency department

History

Reason for Visit/Chief Concern: "I'm coughing up green and yellow mucus."

History of Present Illness:

- cystic fibrosis with two to three exacerbations yearly for past 5 years
- 3-day history of cough productive of copious amounts of yellow and green sputum
- associated with subjective fever, shaking chills, and progressive shortness of breath
- symptoms occurred first only with exertion but now occur at rest
- also reports sharp left-sided chest pain that she attributes to extended paroxysms of coughing
- symptoms are similar to previous exacerbations

Past Medical History:

- cystic fibrosis complicated by bronchiectasis and pancreatic insufficiency

Medications:

- pancrelipase
- dornase alfa
- ivacaftor
- albuterol prn for pulmonary symptoms
- 3.0% inhaled saline prn for pulmonary symptoms

Allergies:

- no known drug allergies

Family History:

- brother: age 13 years with cystic fibrosis

Psychosocial History:

- does not smoke cigarettes, drink alcoholic beverages, or use other substances
- high school student

Physical Examination

Temp	Pulse	Resp	BP	O_2 Sat	Ht	Wt	BMI
37.9°C (100.2°F)	112/min and regular	22/min	138/74 mm Hg	92% on RA	157 cm (5 ft 2 in)	54 kg (118 lb)	22 kg/m^2

- Appearance: in mild respiratory distress; using accessory muscles of respiration; frequent coughing productive of yellow and green sputum
- Pulmonary: bilateral diffuse crackles with moderate diffuse expiratory wheezes more prominent in the apices

Diagnostic Studies

Blood	
Hematocrit	42%
Hemoglobin	13.1 g/dL
WBC	16,400/mm^3
Platelet count	334,000/mm^3

- portable chest x-ray: bilateral basilar consolidations with increased interstitial markings; no effusions or pneumothorax

Question: In addition to initiating broad-spectrum antibiotic therapy, which of the following is the most appropriate next step in evaluation?

(A) CT angiography of the chest
(B) Serum immunoglobulin concentrations
(C) Spirometry with lung volume measurement
(D) Sputum culture
(E) Transbronchial lung biopsy

94. A 16-year-old boy comes to the emergency department (ED) because of moderate left knee pain and abrasions sustained 2 hours ago in a skateboarding collision. He has a history of occasional injuries sustained while skateboarding, including an injury to the same knee 6 months ago. He says his knee had felt fine until today's injury, and he has been fit and active. The patient has an *EXT1* mutation, which is also present in his father. The patient takes no medications. Vital signs are within normal limits. Examination shows superficial abrasions over the anterolateral aspect of the left knee. No effusion or instability is noted in the knee, but there is tenderness to palpation. The patient walks with a limp but is able to bear weight on the leg. X-ray of the left knee shows two 1 × 2-cm bone polyps extending, one medially and one laterally, from the proximal tibia at the level of the closing physis; this is unchanged from an x-ray of the same knee obtained during his previous ED visit 6 months ago. Which of the following is the most appropriate recommendation for activity and additional workup?

(A) Biopsy of the polyps and recommendation to bear weight as tolerated
(B) Biopsy of the polyps and recommendation for no weight bearing
(C) MRI of the knee and recommendation to bear weight as tolerated
(D) MRI of the knee and recommendation for no weight bearing
(E) Recommendation to bear weight as tolerated; no further workup needed
(F) Recommendation for no weight bearing; no further workup needed

95. A 70-year-old woman comes to the office because of three episodes of rectal bleeding during the past 4 days. During each episode, she had the sudden urge to have a bowel movement and then passed a bright red, bloody stool. She has not had chest, abdominal, or rectal pain, palpitations, dizziness, light-headedness, or black stools. Her most recent colonoscopy 8 years ago showed no abnormalities. She has osteoarthritis treated with daily ibuprofen. She takes no other medications. She is 168 cm (5 ft 6 in) tall and weighs 69 kg (152 lb); BMI is 25 kg/m^2. Temperature is 37.0°C (98.6°F), and respirations are 16/min. While sitting, her pulse is 80/min and blood pressure is 130/76 mm Hg. While standing, her pulse is 88/min and blood pressure is 126/74 mm Hg. On examination, mucous membranes are moist. The abdomen is soft, nontender, and nondistended. There is no rebound tenderness or guarding. Bowel sounds are normal. Rectal examination shows no stool in the vault; there are no masses. Which of the following is the most likely diagnosis?

(A) Anal fissure
(B) Colonic polyp
(C) Diverticulosis
(D) Duodenal ulcer
(E) Gastritis
(F) Ulcerative colitis

96. A 14-year-old boy is brought to the office because of a 6-month history of changes in behavior. Two days ago, the patient and his friends were detained by police for trespassing on abandoned property and entering an unoccupied house. No alcohol or other substances were involved. The police officer who brought the patient home reported that there was evidence indicating this had not been the first time the boys entered the property. The mother says her son was polite and close with his parents until 6 months ago when he began socializing with a different group of boys in his class. She says the patient is constantly exchanging text messages with his new friends. The group has inside jokes and nicknames for one another. Every weekend, the patient goes out with his friends and argues with his parents about his curfew. At home, he is irritable and shares few details about his outside activities with his parents. He has no history of serious illness and receives no medications. Examination shows no abnormalities. Urine toxicology screening is negative. Which of the following is the most likely explanation for this patient’s behavior?

(A) Adjustment disorder
(B) Attention-deficit/hyperactivity disorder
(C) Conduct disorder
(D) Oppositional defiant disorder
(E) Normal development

97. A 6-year-old boy is brought to the emergency department by his mother because of the acute onset 1 hour ago of severe right-sided lumbar pain and three episodes of emesis. The pain is sharp and causes him to double over and cry. Medical and family histories are unremarkable. The patient takes no medications. Temperature is 36.7°C (98.0°F), pulse is 150/min, respirations are 30/min, and blood pressure is 110/70 mm Hg. He appears to be in severe pain. Abdominal examination discloses right upper quadrant tenderness to palpation. The remainder of the examination discloses no abnormalities. Intravenous morphine is administered and results in improvement in the patient's pain. Results of dipstick urinalysis are shown:

Specific gravity	1.035 (N=1.003–1.029)
pH	5.5
Protein	30 mg/dL
Glucose	Negative
Bilirubin	Negative
Blood	4+
Leukocyte esterase	1+
Nitrite	Negative
Urobilinogen	Negative
WBCs	5–10/hpf
RBCs	50–100/hpf

Renal ultrasonography shows severe right-sided hydronephrosis with moderate hydroureter to the bladder. Which of the following is the most appropriate next step in evaluation?

(A) Captopril renography
(B) CT scan of the abdomen and pelvis
(C) MAG-3 renal scan with furosemide
(D) Radionuclide cystography
(E) Retrograde pyelography
(F) Ultrasonography of the spinal column
(G) Voiding cystourethrography

98. A 20-year-old man comes to the office at the end of the summer because of a 2-month history of generalized malaise, fatigue, intermittent cough productive of whitish sputum, and decreased appetite. During this time, he has lost approximately 4 kg (10 lb). The patient previously was on the college track team but now feels winded when he tries to go running. He has a history of childhood asthma. He currently takes no medications. He does not smoke cigarettes, drink alcoholic beverages, or use other substances. He identifies as African American. He has been working at a lumber mill for the summer, feeding logs into a saw. He is 178 cm (5 ft 10 in) tall and weighs 77 kg (170 lb); BMI is 24 kg/m^2. Temperature is 37.7°C (99.9°F), pulse is 78/min, respirations are 20/min, and blood pressure is 114/70 mm Hg. Diffuse crackles are heard bilaterally. The remainder of the examination shows no abnormalities. Chest x-ray shows a reticulonodular pattern that is more prominent in the upper lung fields. Results of pulmonary function tests show a mixed obstructive and restrictive pattern. Which of the following is the most likely diagnosis?

(A) Aspergillosis
(B) Asthma recurrence
(C) Hypersensitivity pneumonitis
(D) Sarcoidosis
(E) Silicosis

99. A 32-year-old woman is admitted to the hospital because of a 2-year history of intractable seizures. Her boyfriend, who has accompanied her, describes the seizures as episodes of bilateral limb shaking and moving her head from side to side, during which the patient closes her eyes and cries. The episodes last 15 to 20 minutes, and she is intermittently responsive during this time. Trials of phenytoin, carbamazepine, gabapentin, oxcarbazepine, and divalproex have not provided relief of her symptoms. Her current medications are zonisamide and pregabalin. The patient has a history of childhood sexual and physical abuse. Vital signs are within normal limits. Neurologic examination shows no focal findings. The patient undergoes long-term video EEG monitoring, which shows that her seizures are not associated with any EEG changes. Which of the following is the most appropriate next step in management?

(A) Cognitive behavioral therapy
(B) Hypnotic therapy
(C) Increasing the dosage of pregabalin
(D) Psychoanalytic therapy
(E) Surgical resection of the epileptogenic focus

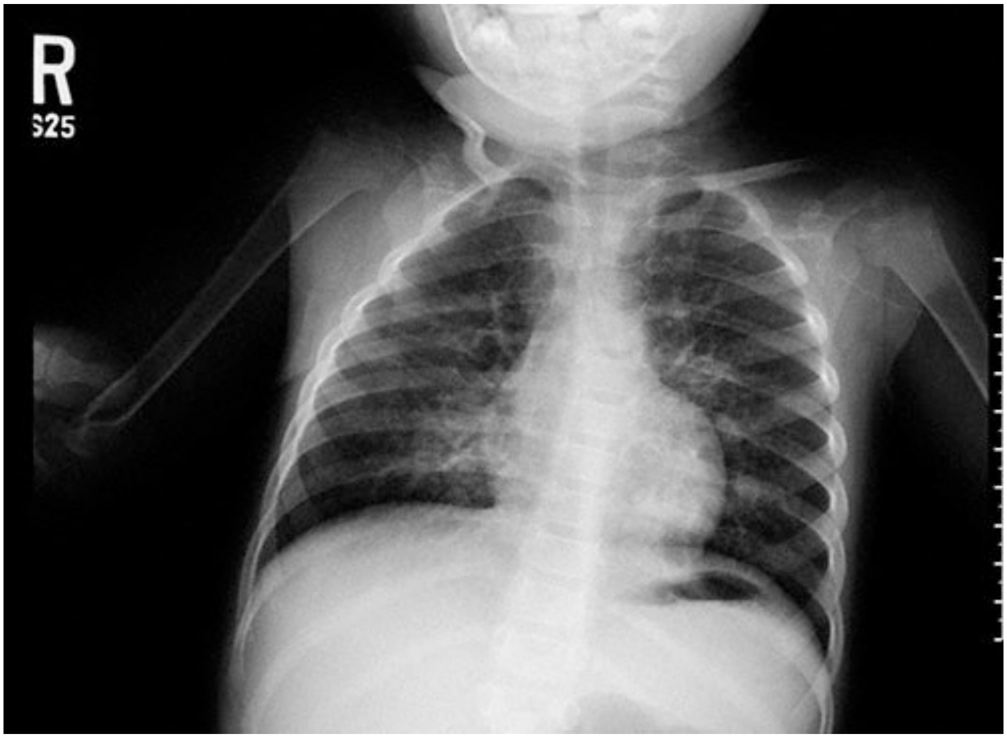

100. A 2-year-old girl is brought to the clinic by her parents for an initial examination. Medical records show that she has a cleft palate and ventriculoseptal defects that have not been repaired. During the past 18 months, she has had two episodes of pneumonia and at least 10 episodes of otitis media treated with antibiotic therapy. The patient's parents recently adopted her from China. She is at the 10th percentile for length and 5th percentile for weight. Vital signs are within normal limits. Examination shows middle ear effusions bilaterally and a cleft palate. Lungs are clear to auscultation. A grade 3/6 holosystolic murmur is heard on cardiac examination. There is no hepatosplenomegaly. Chest x-ray is shown. A complete blood count is most likely to show which of the following leukocyte findings in this patient?

(A) Eosinophilia
(B) Lymphocytosis
(C) Lymphopenia
(D) Neutropenia
(E) Neutrophilia

101. A 25-year-old primigravid woman at 28 weeks' gestation comes to the office for a routine prenatal visit. At 18 weeks' gestation, she had vaginal bleeding that resolved spontaneously. At that time, her blood group was determined to be O, Rh-negative and she received $Rh_o(D)$ immune globulin. She reports good fetal movement. Blood pressure is 120/70 mm Hg. Fetal heart rate is 160/min. Fundal height is 30 cm. The fetus is in a vertex presentation. Hemoglobin concentration is 11 g/dL and hematocrit is 34%. Serum antibody assay is positive; the anti-D titer is too weak to measure. Results of serologic testing for syphilis and HIV antibody testing are negative. Which of the following is the most appropriate next step in management?

(A) Repeat serum anti-D antibody titer
(B) Ultrasonography of the pelvis
(C) Administration of $Rh_o(D)$ immune globulin
(D) Amniocentesis
(E) Induction of labor

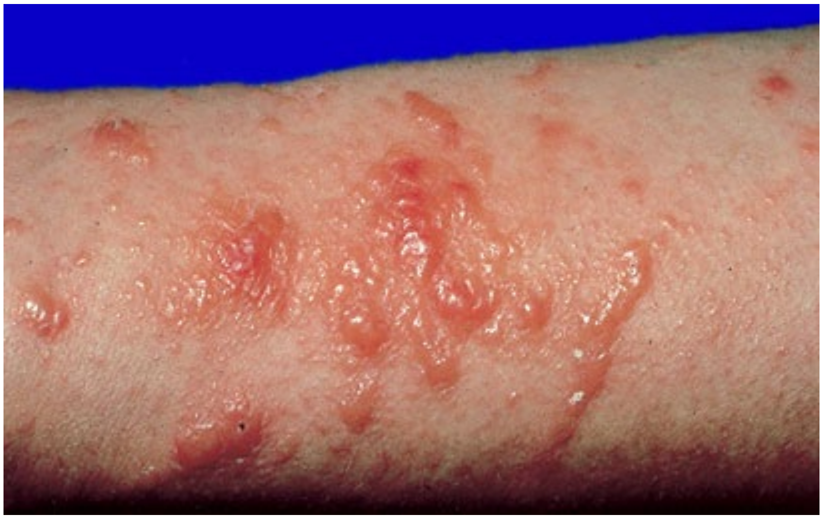

102. A 5-year-old boy is brought to the office by his parents in July because of a 2-day history of an itchy rash. The rash began as small red bumps on the right forearm and spread to the left cheek and right ankle within the same day. He has not had fever, vomiting, or diarrhea. No other family members have similar symptoms. Medical history is unremarkable and he receives no medications. The family switched to a new brand of bath soap last week. They have three pet cats and one dog. Three days ago, the family attended a picnic at a nearby park. Temperature is 37.0°C (98.6°F), pulse is 90/min, and respirations are 20/min. The patient is alert and scratching his right forearm. Examination shows mild edema of the left cheek. A photograph of the right forearm is shown. Similar lesions are noted over the left cheek and right ankle. The remainder of the examination shows no abnormalities. Avoidance of which of the following is most likely to have prevented this patient’s condition?

(A) Dog
(B) Flora
(C) Peanut butter
(D) Perfumed bath soap
(E) Sun

103. **Patient Information**

Age: 56 years
Gender: F, self-identified
Race/Ethnicity: mixed race (White and Honduran)
Site of Care: clinic

History

Reason for Visit/Chief Concern: "I'm tired and I don't feel well."
History of Present Illness:
- fatigue and malaise for 3 months; tires quickly with physical activity
- early satiety

Past Medical History:
- 5-year history of hypertension
- 5-year history of hyperlipidemia

Medications:
- chlorthalidone
- enalapril
- atorvastatin

Allergies:
- no known drug allergies

Physical Examination

Temp	Pulse	Resp	BP	O_2 Sat	Ht	Wt	BMI
37.5°C (99.5°F)	75/min	14/min	139/78 mm Hg	–	162 cm (5 ft 4 in)	94 kg (207 lb)	36 kg/m^2

- HEENT: normal conjunctivae and oropharynx; thyroid gland is normal in size with no mass or tenderness
- Pulmonary: clear to auscultation
- Cardiac: S_1 and S_2; no murmur or gallop
- Abdominal: nontender; liver edge palpated at the right costal margin with inspiration; dullness to percussion over the fifth to eighth intercostal spaces at the left anterior axillary line

Diagnostic Studies

Blood		Urine	
RBC	4.5 million/mm^3	Protein	3+
Hematocrit	39%		
Hemoglobin	13.0 g/dL		
MCV	86.7 μm^3		
WBC	67,000/mm^3		
Neutrophils, segmented	83%		
Lymphocytes	2%		
Eosinophils	5%		
Basophils	4%		
Metamyelocytes	3%		
Myelocytes	3%		

Question: Which of the following is the most likely diagnosis?

(A) Acute lymphocytic leukemia
(B) Acute myeloid leukemia
(C) Chronic lymphocytic leukemia
(D) Chronic myeloid leukemia
(E) Monoclonal gammopathy of undetermined significance

104. A 65-year-old woman comes to the office because of a 6-month history of progressive shortness of breath and a 1-year history of nonproductive cough. Initially, the shortness of breath occurred only with exertion but now occurs at rest. It does not worsen when she lies down but is still present. Her symptoms have limited her ability to travel internationally with the Peace Corps. She has not had chest pain, fever, chills, or blood-tinged sputum. She has hypertension, type 2 diabetes mellitus, and major depressive disorder. Medications are amlodipine, lisinopril, insulin glargine, and sertraline. She has smoked one pack of cigarettes daily for 50 years. She drinks one glass of wine on special occasions. Temperature is 37.2°C (99.0°F), pulse is 77/min, respirations are 16/min, and blood pressure is 132/82 mm Hg. Pulse oximetry on room air shows an oxygen saturation of 94%. There is no jugular venous distention. On pulmonary examination, end-expiratory wheezes are heard; there is a prolonged expiratory phase. Heart sounds are distant. Which of the following is the most appropriate next step in diagnosis?

(A) CT scan of the chest
(B) Echocardiography
(C) Exercise stress testing
(D) PPD skin testing and chest x-ray
(E) Pulmonary function testing

105. A 45-year-old man comes to the office because of a 6-month history of difficulty sleeping, nervousness, and fatigue. He goes to bed at 11 PM every night, falls asleep within 15 minutes, then sleeps "fitfully." After he awakens, he has mild neck pain and feels tired, edgy, and tense during the rest of the day. The company where he worked for 27 years closed 6 months ago, and he has been unable to find another job. He says he worries about his finances and job prospects. Medical history is remarkable for alcohol use disorder. He has not drunk alcohol for 1 year. He says he is committed to being sober but recently has "wanted a drink." He currently takes no medications. Vital signs are within normal limits. Physical examination discloses no abnormalities. Mental status examination shows a constricted, anxious affect. Which of the following is the most appropriate pharmacotherapy?

(A) Buspirone
(B) Clonazepam
(C) Diphenhydramine
(D) Imipramine
(E) Quetiapine
(F) Temazepam

106. A 56-year-old man comes to the clinic with his wife because of a 6-month history of restlessness while sleeping. The wife says he motions as if he were throwing or kicking a ball and sometimes punches the pillow during the night; he has hit her twice. Recently, he began talking in his sleep and yelling expletives. Occasionally, he seems to be "acting out" a dream; twice, he has awakened and could tell his wife what the dream was about. Otherwise, he is unaware of these episodes and feels well rested in the mornings. He has a history of constipation treated with polyethylene glycol. He takes no other medications. Vital signs are within normal limits. Examination shows no abnormalities. This patient is at greatest risk for developing which of the following?

(A) Alzheimer disease
(B) Amyotrophic lateral sclerosis
(C) Cerebral infarction
(D) Narcolepsy
(E) Parkinson disease

107. A 77-year-old woman is brought to the emergency department by her son because of a 3-day history of pain in her right wrist. The patient has advanced dementia, Alzheimer type, and is unable to provide her medical history. She is completely dependent on her son, with whom she lives and who is her only caregiver. The son says he is unaware of any trauma to the patient's wrist. The son says the patient has no other history of serious illness. Her only medication is memantine. She is nonverbal and has a flat affect. She appears unkempt and to have poor hygiene. Temperature is 36.7°C (98.1°F), pulse is 82/min and regular, respirations are 18/min, and blood pressure is 150/90 mm Hg. The patient moans when the physician touches her right wrist. Examination shows multiple bruises over both forearms and wrists. The remainder of the examination shows no abnormalities. X-ray of the right wrist shows a distal radial fracture; the fracture is treated with cast immobilization. Which of the following is the most appropriate next step in management?

(A) Admit the patient to the hospital for further evaluation
(B) Arrange for placement of the patient in a nursing care facility
(C) Contact adult protective services
(D) Provide phone numbers of nearby nursing care facilities
(E) Recommend further evaluation at a clinic specializing in geriatric patients

108. A 27-year-old man comes to the emergency department because of a 4-week history of progressive shortness of breath and swelling of the lower extremities. During this time, he also has had shortness of breath during the night while lying down. He also has a 6-month history of intermittent, moderate substernal chest pain and pressure while exercising. He has not had syncope. He has no history of serious illness and takes no medications. Temperature is 37.0°C (98.6°F), pulse is 110/min, respirations are 24/min, and blood pressure is 145/92 mm Hg. Examination shows severe, diffuse gingivitis. Crackles are heard halfway up the posterior lung bases bilaterally. On cardiac examination, a prominent S_3 is heard. There is 3+ pitting edema of the lower extremities. ECG shows sinus tachycardia. Which of the following is the most likely substance used?

(A) Cocaine
(B) Heroin
(C) Methadone
(D) Methamphetamine
(E) Toluene

109. A 39-year-old man comes to the office because of a 3-month history of episodes of headaches, palpitations, and sweating. The episodes occur two to three times monthly. He has hypertension treated with a calcium-channel blocking agent and an ACE inhibitor. Blood pressure is 166/112 mm Hg. Examination shows no other abnormalities. CT scan of the abdomen shows a 4-cm left adrenal mass. Which of the following is the most appropriate next step in management?

(A) 24-Hour urine collection for measurement of vanillylmandelic acid and 5-hydroxyindoleacetic acid
(B) Adrenal venous sampling
(C) Laparoscopic left adrenalectomy and right adrenal biopsy
(D) Measurement of plasma and urine catecholamine and metabolite concentrations
(E) Transsphenoidal hypophysectomy

110. A 41-year-old woman is being prepared for discharge from the hospital after treatment for acute pancreatitis. She has tolerated oral fluids well for the past 12 hours. She has had three prior admissions for pancreatitis. Medical history also is remarkable for alcohol use disorder. The patient does not have secure housing. Which of the following recommendations is most likely to reduce this patient's risk for future hospital admissions?

(A) Abstinence from alcoholic beverages
(B) Enrollment in a health insurance plan
(C) Nutritional counseling
(D) Placement in a women's shelter

111. An 8-year-old boy is brought to the urgent care center by his mother while on a family vacation in Florida because of a 3-day history of an itchy, burning rash. The ambient temperature during the past 3 days has been 37.8°C (100.0°F). The mother does not recall any recent exposures for her son, and none of her other children have a rash. The patient's medical history is unremarkable. Examination of the patient's back shows superficial, clear, 1- to 2-mm vesicles that resemble water droplets. The vesicles rupture easily on palpation. There is no surrounding erythema. It is most appropriate for the physician to recommend which of the following for this patient?

(A) Antifungal cream
(B) Heat avoidance
(C) Oral antibiotic therapy
(D) Oral antiviral therapy
(E) Oral retinoid therapy
(F) Ultraviolet therapy

112. A 55-year-old woman comes to the office for a follow-up examination 3 months after sustaining an uncomplicated myocardial infarction in the distribution of the left anterior descending artery. She has participated in an outpatient rehabilitation program since discharge from the hospital 6 weeks ago, but her attendance in the program has become irregular during the past 4 weeks. When questioned about this, she reports a 4-week history of depressed mood, inability to enjoy activities she previously found pleasurable, loss of appetite, and insomnia. She also has hyperlipidemia and hypertension. Medications are aspirin, atorvastatin, and metoprolol. She is 163 cm (5 ft 4 in) tall and weighs 68 kg (150 lb); BMI is 26 kg/m^2. Vital signs are within normal limits. Lungs are clear to auscultation. Cardiac examination discloses a normal rhythm. There is no peripheral edema. Results of serum studies are within the reference ranges. Which of the following is the most likely effect of this patient's depression on her overall health?

(A) It is likely to decrease her perceived quality of life but have no impact on her mortality
(B) It is likely to double her risk for death during the next 3 months
(C) It is likely to decrease her risk for ischemia because she is likely to rest and avoid socializing
(D) It likely will have no significant effect on her physical health

113. A 78-year-old man is evaluated in the hospital following admission 8 hours ago for treatment of aspiration pneumonia. He has a 5-year history of intermittent dysphagia resulting in spontaneous regurgitation of undigested food and liquids. Over-the-counter antacids had provided no improvement in his dysphagia. Until the development of his recent fever, chest pain, and shortness of breath, the patient had not had any other symptoms and had not sought medical care. He has no other history of serious illness. In the hospital, he has been receiving intravenous piperacillin-tazobactam. He does not smoke cigarettes or drink alcoholic beverages. Temperature is 38.1°C (100.6°F), pulse is 90/min, respirations are 24/min, and blood pressure is 100/65 mm Hg. He appears comfortable and is sitting upright in bed. Decreased breath sounds are heard in the right lower lung field. Abdomen is soft and nontender. Which of the following is the most likely underlying cause of this patient's dysphagia?

(A) Achalasia
(B) Esophageal cancer
(C) Hiatal hernia
(D) Zenker diverticulum

114. A 27-year-old woman comes to the office because of a 3-day history of constant mild pain in her left lower abdomen. Vigorous movement makes the pain worse. She has not had nausea. She is otherwise healthy and takes no medications. Menses occur at regular 28-day intervals and last 4 to 6 days. Her last menstrual period was 3 weeks ago. She is sexually active with one male partner, and they use condoms inconsistently. Temperature is 36.7°C (98.0°F), pulse is 80/min, respirations are 18/min, and blood pressure is 104/60 mm Hg. The abdomen is flat and soft with voluntary guarding of the left lower quadrant. Bowel sounds are normal. Pelvic examination shows tender fullness of the left adnexa. Urine pregnancy test is negative. Ultrasonography shows a normal uterus with a 3 × 4-cm left adnexal cyst and a small amount of free fluid in the pelvis. Which of the following is the most appropriate next step in management?

(A) Antibiotic therapy
(B) Appendectomy
(C) CT scan-guided aspiration
(D) Laparoscopy
(E) Observation only

115. A 60-year-old woman comes to the office for a health maintenance examination. She says she feels well. She has no history of serious illness and takes no medications. Vital signs are within normal limits. Observation of the tympanic membranes is limited because of cerumen filling both external auditory canals. The external ears appear normal. No other abnormalities are noted. On questioning, she says she hears well. Which of the following is the most appropriate next step in management?

(A) Irrigation of the ear canals by the physician
(B) Manual removal of the cerumen by an otorhinolaryngologist
(C) Recommendation for daily use of cotton swabs
(D) Recommendation for use of a ceruminolytic agent
(E) No further management is indicated at this time

116. A hospitalized 37-year-old woman has a 2-hour history of severe chest pain and blood-streaked sputum 4 days after undergoing hysterectomy and oophorectomy for ovarian cancer. Current medications are oxycodone, prophylactic low-molecular-weight heparin, and a multivitamin. Temperature is 37.0°C (98.6°F), pulse is 90/min, respirations are 22/min, and blood pressure is 110/70 mm Hg. Examination shows a left pleural rub. An x-ray and CT scan of the chest show a triangular pleural-based density in the lower lobe of the left lung. No pulmonary emboli are seen in the major branches. Venous duplex ultrasonography of the lower extremities shows no deep venous thrombosis. Which of the following is the most likely diagnosis?

(A) Air embolism
(B) Empyema
(C) Hemothorax
(D) Pericarditis
(E) Pneumonia
(F) Pulmonary hemorrhage
(G) Pulmonary hypertension
(H) Pulmonary infarction

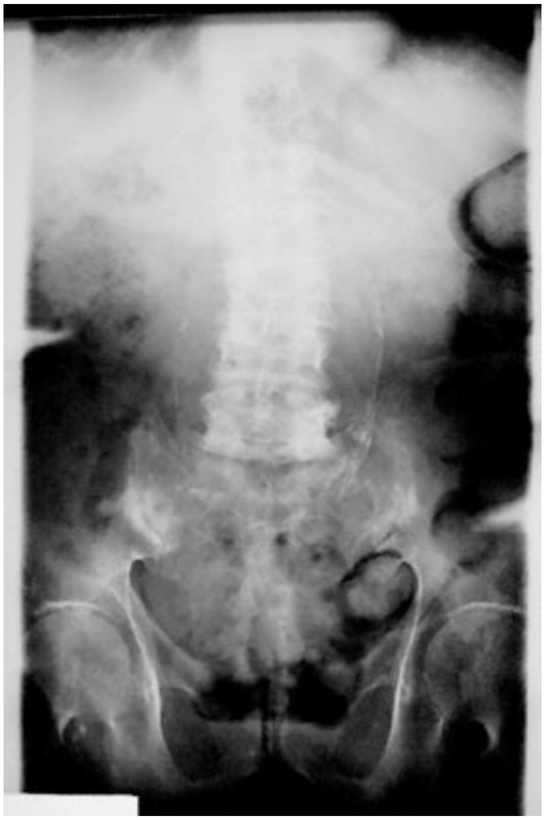

117. A 77-year-old man is brought to the emergency department because of a 1-hour history of severe, constant abdominal pain and dizziness. He has hypertension well controlled with hydrochlorothiazide. He has smoked two packs of cigarettes daily for 60 years. He is 175 cm (5 ft 9 in) tall and weighs 113 kg (250 lb); BMI is 37 kg/m^2. Temperature is 37.0°C (98.6°F), pulse is 120/min, respirations are 25/min, and blood pressure is 80/60 mm Hg. Pulse oximetry on room air shows an oxygen saturation of 93%. He appears uncomfortable. Abdominal examination shows mild distention; bowel sounds are decreased. Groin pulses are 1+. Dorsalis pedis pulses are not palpable. Hematocrit is 37% and leukocyte count is 10,000/mm^3. Serum electrolyte concentrations, liver function tests, and amylase activity are within the reference ranges. An x-ray of the abdomen is shown. Which of the following is the most likely diagnosis?

(A) Acute pancreatitis
(B) Mesenteric ischemia
(C) Perforated duodenal ulcer
(D) Ruptured abdominal aortic aneurysm
(E) Small-bowel obstruction

---

118. A 1-week-old male newborn is evaluated in the hospital nursery 4 days after undergoing operative repair of a myelomeningocele. He was born at 37 weeks' gestation via uncomplicated, spontaneous vaginal delivery to an 18-year-old primigravid woman. The defect was diagnosed prenatally by ultrasonography, and the repair was done 72 hours after delivery. He is at the 5th percentile for length, 10th percentile for weight, and 25th percentile for head circumference. Temperature is 36.9°C (98.4°F), pulse is 138/min, and respirations are 42/min. Pulse oximetry on room air shows an oxygen saturation of 98%. The newborn appears comfortable. Physical examination shows a soft, flat, anterior fontanel. Red reflexes are normal. Moro reflex is symmetric in the upper extremities; there is no movement in the lower extremities. Ultrasonography of the brain shows mildly enlarged ventricles. Which of the following is the most likely associated finding in this patient?

(A) Congenital heart malformation
(B) Cryptorchidism
(C) Impaired folate metabolism
(D) Neurogenic bladder
(E) Severe cognitive impairment

119. A randomized controlled trial is conducted to assess the effectiveness of a new antiarrhythmic drug in patients with recurrent ventricular tachycardia. A total of 100 participants with ventricular tachycardia are randomly assigned to receive either the new drug or a placebo. Results show that the new drug group has fewer episodes of recurrent ventricular tachycardia compared with the placebo group. The difference was found to be statistically significant ($p<0.05$). The investigators conclude that the new drug can be used to decrease mortality from cardiac arrhythmia. Which of the following factors most likely invalidates the author's conclusions?

(A) Extrapolation of findings beyond data
(B) Insufficient power
(C) No information regarding confidence interval
(D) Selection bias

120. A 42-year-old woman comes to the office because of a 2-week history of sore throat and fullness in her neck. She has no history of serious illness and takes no medications. She appears anxious. Temperature is 37.8°C (100.0°F), pulse is 102/min, respirations are 22/min, and blood pressure is 146/82 mm Hg. The oropharynx is clear, and there are no exudates. There is palpable fullness and tenderness of the anterior aspect of the neck. There is no lymphadenopathy. Lungs are clear to auscultation. Results of a complete blood count are within the reference ranges. Chest x-ray shows no abnormalities. Which of the following is the most likely diagnosis?

(A) Acute mononucleosis
(B) Gastroesophageal reflux disease
(C) Laryngitis
(D) Subacute thyroiditis
(E) Tracheitis

ANSWER FORM FOR USMLE STEP 2 CK SAMPLE TEST QUESTIONS

Block 1 (Questions 1–40)

1._____	9._____	17._____	25._____	33._____
2._____	10._____	18._____	26._____	34._____
3._____	11._____	19._____	27._____	35._____
4._____	12._____	20._____	28._____	36._____
5._____	13._____	21._____	29._____	37._____
6._____	14._____	22._____	30._____	38._____
7._____	15._____	23._____	31._____	39._____
8._____	16._____	24._____	32._____	40._____

Block 2 (Questions 41–80)

41._____	49._____	57._____	65._____	73._____
42._____	50._____	58._____	66._____	74._____
43._____	51._____	59._____	67._____	75._____
44._____	52._____	60._____	68._____	76._____
45._____	53._____	61._____	69._____	77._____
46._____	54._____	62._____	70._____	78._____
47._____	55._____	63._____	71._____	79._____
48._____	56._____	64._____	72._____	80._____

Block 3 (Questions 81–120)

81._____	89._____	97._____	105._____	113._____
82._____	90._____	98._____	106._____	114._____
83._____	91._____	99._____	107._____	115._____
84._____	92._____	100._____	108._____	116._____
85._____	93._____	101._____	109._____	117._____
86._____	94._____	102._____	110._____	118._____
87._____	95._____	103._____	111._____	119._____
88._____	96._____	104._____	112._____	120._____

Answer Key for USMLE Step 2 CK Sample Test Questions

Block 1 (Questions 1–40)

1.	A	9.	B	17.	E	25.	D	33.	B
2.	E	10.	F	18.	E	26.	A	34.	A
3.	A	11.	B	19.	C	27.	C	35.	C
4.	A	12.	B	20.	B	28.	B	36.	E
5.	E	13.	B	21.	C	29.	A	37.	D
6.	C	14.	D	22.	E	30.	B	38.	D
7.	B	15.	C	23.	C	31.	B	39.	C
8.	B	16.	B	24.	B	32.	A	40.	A

Block 2 (Questions 41–80)

41.	D	49.	C	57.	D	65.	E	73.	A
42.	A	50.	C	58.	F	66.	A	74.	E
43.	C	51.	C	59.	D	67.	C	75.	E
44.	E	52.	A	60.	D	68.	D	76.	D
45.	B	53.	F	61.	D	69.	B	77.	C
46.	C	54.	E	62.	E	70.	C	78.	B
47.	A	55.	B	63.	B	71.	C	79.	B
48.	A	56.	E	64.	C	72.	C	80.	D

Block 3 (Questions 81–120)

81.	D	89.	E	97.	B	105.	A	113.	D
82.	D	90.	A	98.	C	106.	E	114.	E
83.	E	91.	D	99.	A	107.	C	115.	E
84.	A	92.	B	100.	C	108.	D	116.	H
85.	A	93.	D	101.	C	109.	D	117.	D
86.	B	94.	E	102.	B	110.	A	118.	D
87.	B	95.	C	103.	D	111.	B	119.	A
88.	E	96.	E	104.	E	112.	B	120.	D

Sample Test Questions

Step 3

A Joint Program of the Federation of State Medical Boards of the United States, Inc. (FSMB), and National Board of Medical Examiners® (NBME®)

This booklet was updated December 2023.

Copyright © 2023 by the Federation of State Medical Boards of the United States, Inc. (FSMB), and National Board of Medical Examiners® (NBME®). All rights reserved. Printed in the United States of America. The United States Medical Licensing Examination® (USMLE®) is a joint program of the FSMB and NBME.

CONTENTS

USMLE Step 3 Multiple Choice Test Question Formats 2
Introduction to USMLE Step 3 Sample Test Questions.. 5
USMLE Step 3 Normal Laboratory Values 6
Answer Form for USMLE Step 3 Sample Questions.. 9
USMLE Step 3 Sample Test Questions 10
Answer Key for USMLE Step 3 Sample Questions 95

USMLE STEP 3 MULTIPLE-CHOICE TEST QUESTION FORMATS

The following are strategies for answering one-best-answer questions (eg, Single Items, Multiple Item Sets, and Sequential Item Sets):

- Read each patient vignette and question carefully. It is important to understand what is being asked.
- Try to generate an answer and then look for it in the option list.
- Alternatively, read each option carefully, eliminating those that are clearly incorrect. Of the remaining options, select the one that is most correct.
- If unsure about an answer, it is better to guess since unanswered questions are automatically counted as wrong answers.

Patient Scenario Formats

Patient scenarios for any Single Item or Sequential Item Set may be provided in either Vignette (paragraph) format, or in Chart/Tabular format. Questions written in Chart/Tabular format will contain relevant patient information in list form, organized in clearly marked sections for ease of review. Familiar medical abbreviations may be used within Chart/Tabular format questions.

Single-Item Questions

A single patient-centered scenario is associated with one question followed by four or more response options. The response options for all questions are lettered (ie, A, B, C, D, E). You are required to select the best answer to the question. Other options may be partially correct, but there is only ONE BEST answer. This is the traditional, most frequently used multiple-choice question format on the examination.

Example Question 1

1. A 30-year-old man comes to the emergency department because of an acute episode of renal colic. Medical history is remarkable for episodes of painful urination and passing of what he calls "gravel in my urine." Urinalysis demonstrates microscopic hematuria with some crystalluria and no casts. Supine x-ray of the abdomen shows no abnormalities. A 4-mm renal calculus is detected in the distal right ureter on ultrasonography. There is no evidence of dilation of the collecting system. The patient's pain is responsive to narcotic medication. In addition to administering intravenous fluids, which of the following is the most appropriate next step?

   A. Acidification of urine by drinking cranberry juice
   B. Cystoscopic removal of the calculus
   C. Cystoscopic ureteral lavage
   D. Shock wave lithotripsy
   E. Straining of the urine
   (Answer: E)

Sequential Item Sets

A single patient-centered scenario may be associated with two or three consecutive questions about the information presented. Each question is associated with the initial patient scenario but is testing a different point. You are required to select the ONE BEST answer to each question. Questions are designed to be answered in sequential order. You must click "Proceed to Next Item" to view the next question in the set; once you click on this button, the next question will be displayed, and you will not be able to add or change an answer to the previous question.

Example Questions 4 to 5

A 2-year-old girl is brought to the office by her mother for evaluation of fever. You have been the girl's physician since birth. While in the office, the girl stiffens and then has bilateral, symmetrical shaking of her upper and lower extremities; she becomes mildly cyanotic. The episode lasts for approximately 45 seconds, after which she becomes relaxed and appears to fall asleep. Vital signs at this time are temperature 40.0°C (104.0°F), pulse 120/min, and respirations 40/min. On physical examination she has a generally pink complexion and flushed cheeks. She is limp and somnolent and responds with a cry to noxious stimulus. Tympanic membranes are inflamed bilaterally, nose has a scant, clear discharge, and throat is mildly erythematous. Lungs are clear to auscultation except for transmitted upper airway sounds. Heart has rapid rate with a grade 1/6 systolic murmur at the left sternal border. Complete blood count, blood culture, lumbar puncture, and catheterized urine specimen are obtained and sent for stat analysis. Acetaminophen is administered by rectal suppository. Thirty minutes later the patient awakens and is smiling. She is afebrile. Additional history discloses that she was born at term, she had an uneventful neonatal course, she has normal growth and development, and vaccinations are up-to-date. She has never had an episode similar to this. Initial laboratory results are shown:

Blood	
WBC	10,400/mm^3
Neutrophils, segmented	25%
Neutrophils, bands	5%
Lymphocytes	65%
Monocytes	5%
Cerebrospinal fluid	0 RBC/mm^3
Urinalysis	Normal

Other laboratory studies are pending.

4. In addition to ampicillin for otitis media and acetaminophen, this child also should receive which of the following?

   A. Oral ethosuximide
   B. Oral phenobarbital
   C. Oral phenytoin
   D. Rectal diazepam
   E. No additional medications
   (Answer E)

5. Two weeks later the patient is brought to the office for a follow-up visit. Her mother says that she is doing well and she has had no recurrence of her symptoms. Examination of the ears shows resolution of the otitis media. Which of the following is the most important diagnostic step at this time?

   A. Audiology testing
   B. Cognitive testing
   C. CT scan of the head
   D. EEG
   E. No additional testing
   (Answer E)

INTRODUCTION TO USMLE STEP 3 SAMPLE TEST QUESTIONS

The following pages include 137 sample test questions. Please note that reviewing the sample questions is not a substitute for acquainting yourself with the test software. You should run the Step 3 tutorial and practice test questions that are provided on the USMLE website well before your test date. The sample materials on the USMLE website include additional item formats that do not appear in this booklet (eg, items with associated audio findings). Note that the function of items such as pharmaceutical ads, abstracts, and sequential item sets is unique in an examination interface. You should become familiar with all test question/item formats as they will be used in the actual examination.

In addition, the computer-based case simulation (CCS) format you will see on an actual Step 3 examination is not represented in this booklet. You must become familiar with the CCS format by reading information available in the *USMLE Content Description and General Information* booklet and by practicing with sample CCS cases before you take the Step 3 examination; the information and the practice materials are available on the USMLE Web site (www.usmle.org).

These sample questions are illustrative of the types of questions used in the Step 3 examination. Although the questions exemplify content on the examination, they may not reflect the content coverage on individual examinations. Questions are grouped together by the content appropriate for each examination day in the same manner as in the actual computer-administered test blocks. In the actual examination, the questions will be presented one at a time in a format designed for easy on-screen reading, including use of a panel for the table of normal laboratory values (included here on pages 6–8) and some pictorials. Photographs, charts, and x-rays referred to in this booklet are not of the same quality as the pictorials used in the actual examination. In addition, you will have the capability to adjust the brightness and contrast of pictorials on the computer screen.

To take the following sample test questions as they would be timed in the actual examination, you should allow a maximum of 1 hour for each of the Foundations of Independent Practice (FIP) blocks, and a maximum of 45 minutes for each of the Advanced Clinical Medicine (ACM) blocks, for a total of 3 hours 30 minutes. Please be aware that most examinees perceive the time pressure to be greater during an actual examination. An answer sheet for recording answers for this practice is provided on page 9. An answer key is provided on page 95. In the actual examination, answers will be selected on the screen; **no answer form will be provided**.

USMLE LABORATORY VALUES

	Reference Range	SI Reference Intervals
**SERUM**		
General Chemistry:		
Electrolytes		
Sodium (Na^+)	136–146 mEq/L	136–146 mmol/L
Potassium (K^+)	3.5–5.0 mEq/L	3.5–5.0 mmol/L
Chloride (Cl^-)	95–105 mEq/L	95–105 mmol/L
Bicarbonate (HCO_3^-)	22–28 mEq/L	22–28 mmol/L
Urea nitrogen	7–18 mg/dL	2.5–6.4 mmol/L
Creatinine	0.6–1.2 mg/dL	53–106 µmol/L
Glucose	Fasting: 70–100 mg/dL	3.8–5.6 mmol/L
	Random, non-fasting: <140 mg/dL	<7.77 mmol/L
Calcium	8.4–10.2 mg/dL	2.1–2.6 mmol/L
Magnesium (Mg^{2+})	1.5–2.0 mg/dL	0.75–1.0 mmol/L
Phosphorus (inorganic)	3.0–4.5 mg/dL	1.0–1.5 mmol/L
Hepatic:		
Alanine aminotransferase (ALT)	10–40 U/L	10–40 U/L
Aspartate aminotransferase (AST)	12–38 U/L	12–38 U/L
Alkaline phosphatase	25–100 U/L	25–100 U/L
Amylase	25–125 U/L	25–125 U/L
Bilirubin, Total // Direct	0.1–1.0 mg/dL // 0.0–0.3 mg/dL	2–17 µmol/L // 0–5 µmol/L
Proteins, total	6.0–7.8 g/dL	60–78 g/L
Albumin	3.5–5.5 g/dL	35–55 g/L
Globulin	2.3–3.5 g/dL	23–35 g/L
Lipids:		
Cholesterol		
Total	Normal: <200 mg/dL	<5.2 mmol/L
	High: >240 mg/dL	>6.2 mmol/L
HDL	40–60 mg/dL	1.0–1.6 mmol/L
LDL	<160 mg/dL	<4.2 mmol/L
Triglycerides	Normal: <150 mg/dL	<1.70 mmol/L
	Borderline: 151–199 mg/dL	1.71–2.25 mmol/L
Iron Studies:		
Ferritin	Male: 20–250 ng/mL	20–250 µg/L
	Female: 10–120 ng/mL	10–120 µg/L
Iron	Male: 65–175 µg/dL	11.6–31.3 µmol/L
	Female: 50–170 µg/dL	9.0–30.4 µmol/L
Total iron-binding capacity	250–400 µg/dL	44.8–71.6 µmol/L
Transferrin	200–360 mg/dL	2.0–3.6 g/L

Continued on Next Page

USMLE LABORATORY VALUES (continued)

	Reference Range	SI Reference Intervals
Endocrine:		
Follicle-stimulating hormone	Male: 4–25 mIU/mL	4–25 IU/L
	Female: premenopause 4–30 mIU/mL	4–30 IU/L
	midcycle peak 10–90 mIU/mL	10–90 IU/L
	postmenopause 40–250 mIU/mL	40–250 IU/L
Luteinizing hormone	Male: 6–23 mIU/mL	6–23 IU/L
	Female: follicular phase 5–30 mIU/mL	5–30 IU/L
	midcycle 75–150 mIU/mL	75–150 IU/L
	postmenopause 30–200 mIU/mL	30–200 IU/L
Growth hormone - arginine stimulation	Fasting: <5 ng/mL	<5 µg/L
	Provocative stimuli: >7 ng/mL	>7 µg/L
Prolactin (hPRL)	Male: <17 ng/mL	<17 µg/L
	Female: <25 ng/mL	<25 µg/L
Cortisol	0800 h: 5–23 µg/dL	138–635 nmol/L
	1600 h: 3–15 µg/dL	82–413 nmol/L
	2000 h: <50% of 0800 h	Fraction of 0800 h: <0.50
TSH	0.4–4.0 µU/mL	0.4–4.0 mIU/L
Triiodothyronine (T_3) (RIA)	100–200 ng/dL	1.5–3.1 nmol/L
Triiodothyronine (T_3) resin uptake	25%–35%	0.25–0.35
Thyroxine (T_4)	5–12 µg/dL	64–155 nmol/L
Free T_4	0.9–1.7 ng/dL	12.0–21.9 pmol/L
Thyroidal iodine (^{123}I) uptake	8%–30% of administered dose/24 h	0.08–0.30/24 h
Intact PTH	10–60 pg/mL	10–60 ng/L
17-Hydroxycorticosteroids	Male: 3.0–10.0 mg/24 h	8.2–27.6 µmol/24 h
	Female: 2.0–8.0 mg/24 h	5.5–22.0 µmol/24 h
17-Ketosteroids, total	Male: 8–20 mg/24 h	28–70 µmol/24 h
	Female: 6–15 mg/24 h	21–52 µmol/24 h
Immunoglobulins:		
IgA	76–390 mg/dL	0.76–3.90 g/L
IgE	0–380 IU/mL	0–380 kIU/L
IgG	650–1500 mg/dL	6.5–15.0 g/L
IgM	50–300 mg/dL	0.5–3.0 g/L
Other, serum:		
Creatinine clearance	Male: 97–137 mL/min	97–137 mL/min
	Female: 88–128 mL/min	88–128 mL/min
Creatine kinase	Male: 25–90 U/L	25–90 U/L
	Female: 10–70 U/L	10–70 U/L
Lactate dehydrogenase	45–200 U/L	45–200 U/L
Osmolality	275–295 mOsmol/kg H_2O	275–295 mOsmol/kg H_2O
Uric acid	3.0–8.2 mg/dL	0.18–0.48 mmol/L
**GASES, ARTERIAL BLOOD (ROOM AIR)**		
P_{O_2}	75–105 mm Hg	10.0–14.0 kPa
P_{CO_2}	33–45 mm Hg	4.4–5.9 kPa
pH	7.35–7.45	$[H^+]$ 36–44 nmol/L
**CEREBROSPINAL FLUID**		
Cell count	0–5/mm^3	0–5 × 10^6/L
Chloride	118–132 mEq/L	118–132 mmol/L
Gamma globulin	3%–12% total proteins	0.03–0.12
Glucose	40–70 mg/dL	2.2–3.9 mmol/L
Pressure	70–180 mm H_2O	70–180 mm H_2O
Proteins, total	<40 mg/dL	<0.40 g/L

Continued on Next Page

USMLE LABORATORY VALUES (continued)

	Reference Range	SI Reference Intervals
**HEMATOLOGIC**		
Complete Blood Count:		
Hematocrit	Male: 41%–53%	0.41–0.53
	Female: 36%–46%	0.36–0.46
Hemoglobin, blood	Male: 13.5–17.5 g/dL	135–175 g/L
	Female: 12.0–16.0 g/dL	120–160 g/L
Mean corpuscular hemoglobin (MCH)	25–35 pg/cell	0.39–0.54 fmol/cell
Mean corpuscular hemoglobin conc. (MCHC)	31%–36% Hb/cell	4.8–5.6 mmol Hb/L
Mean corpuscular volume (MCV)	80–100 μm^3	80–100 fL
Volume		
Plasma	Male: 25–43 mL/kg	0.025–0.043 L/kg
	Female: 28–45 mL/kg	0.028–0.045 L/kg
Red cell	Male: 20–36 mL/kg	0.020–0.036 L/kg
	Female: 19–31 mL/kg	0.019–0.031 L/kg
Leukocyte count (WBC)	4500–11,000/mm^3	4.5–11.0 × 10^9/L
Neutrophils, segmented	54%–62%	0.54–0.62
Neutrophils, bands	3%–5%	0.03–0.05
Lymphocytes	25%–33%	0.25–0.33
Monocytes	3%–7%	0.03–0.07
Eosinophils	1%–3%	0.01–0.03
Basophils	0%–0.75%	0.00–0.0075
Platelet count	150,000–400,000/mm^3	150–400 × 10^9/L
Coagulation:		
Partial thromboplastin time (PTT) (activated)	25–40 seconds	25–40 seconds
Prothrombin time (PT)	11–15 seconds	11–15 seconds
D-Dimer	≤250 ng/mL	≤1.4 nmol/L
Other, Hematologic:		
Reticulocyte count	0.5%–1.5%	0.005–0.015
Erythrocyte count (RBC)	Male: 4.3–5.9 million/mm^3	4.3–5.9 × 10^{12}/L
	Female: 3.5–5.5 million/mm^3	3.5–5.5 × 10^{12}/L
Erythrocyte sedimentation rate (Westergren)	Male: 0–15 mm/h	0–15 mm/h
	Female: 0–20 mm/h	0–20 mm/h
CD4+ T-lymphocyte count	≥500/mm^3	≥0.5 × 10^9/L
Troponin I	≤0.04 ng/mL	≤0.04 µg/L
Endocrine:		
Hemoglobin A_{1c}	≤6%	≤42 mmol/mol
**URINE**		
Calcium	100–300 mg/24 h	2.5–7.5 mmol/24 h
Osmolality	50–1200 mOsmol/kg H_2O	50–1200 mOsmol/kg H_2O
Oxalate	8–40 µg/mL	90–445 µmol/L
Proteins, total	<150 mg/24 h	<0.15 g/24 h
**BODY MASS INDEX (BMI)**	Adult: 19–25 kg/m^2	

ANSWER FORM FOR USMLE STEP 3 SAMPLE QUESTIONS

Block 1 (Questions 1–38): FIP

1. ______	9. ______	17. ______	25. ______	33. ______
2. ______	10. ______	18. ______	26. ______	34. ______
3. ______	11. ______	19. ______	27. ______	35. ______
4. ______	12. ______	20. ______	28. ______	36. ______
5. ______	13. ______	21. ______	29. ______	37. ______
6. ______	14. ______	22. ______	30. ______	38. ______
7. ______	15. ______	23. ______	31. ______	
8. ______	16. ______	24. ______	32. ______	

Block 2 (Questions 39–77): FIP

39. ______	47. ______	55. ______	63. ______	71. ______
40. ______	48. ______	56. ______	64. ______	72. ______
41. ______	49. ______	57. ______	65. ______	73. ______
42. ______	50. ______	58. ______	66. ______	74. ______
43. ______	51. ______	59. ______	67. ______	75. ______
44. ______	52. ______	60. ______	68. ______	76. ______
45. ______	53. ______	61. ______	69. ______	77. ______
46. ______	54. ______	62. ______	70. ______	

Block 3 (Questions 78–107): ACM

78. ______	84. ______	90. ______	96. ______	102. ______
79. ______	85. ______	91. ______	97. ______	103. ______
80. ______	86. ______	92. ______	98. ______	104. ______
81. ______	87. ______	93. ______	99. ______	105. ______
82. ______	88. ______	94. ______	100. ______	106. ______
83. ______	89. ______	95. ______	101. ______	107. ______

Block 4 (Questions 108–137): ACM

108. ______	114. ______	120. ______	126. ______	132. ______
109. ______	115. ______	121. ______	127. ______	133. ______
110. ______	116. ______	122. ______	128. ______	134. ______
111. ______	117. ______	123. ______	129. ______	135. ______
112. ______	118. ______	124. ______	130. ______	136. ______
113. ______	119. ______	125. ______	131. ______	137. ______

USMLE STEP 3 SAMPLE TEST QUESTIONS

GENERAL INSTRUCTIONS: Read each question carefully and in the order in which it is presented. Then select the one best response option of the choices offered. More than one option may be partially correct. You must select the **ONE BEST** answer and fill in the corresponding blank line on the answer sheet.

Some items are grouped together around a clinical vignette as a set or case; be particularly careful to read and answer these cases or sets of items in the order they are presented.

The items in this exam are divided into blocks according to the day they will appear on the actual Step 3 examination. The first day of the Step 3 examination is referred to as Foundations of Independent Practice (FIP), and the second day is referred to as Advanced Clinical Medicine (ACM).

Block 1:	Foundations of Independent Practice (FIP)	Items 1–38
Block 2:	Foundations of Independent Practice (FIP)	Items 39–77
Block 3:	Advanced Clinical Medicine (ACM)	Items 78–107
Block 4:	Advanced Clinical Medicine (ACM)	Items 108–137

Block 1: FIP

Items 1–38; Time - 1 hour

ALL ITEMS REQUIRE SELECTION OF ONE BEST CHOICE.

1. A 75-year-old man is brought to the emergency department by his son 2 hours after the sudden onset of fever, chills, pleuritic chest pain, and cough productive of rust-colored sputum. He rates his chest pain as an 8 on a 10-point scale. Temperature is 38.9°C (102°F), pulse is 106/min, respirations are 22/min, and blood pressure is 130/80 mm Hg. Oxygen saturation is 94% on room air. The patient appears to be in moderate respiratory distress. Physical examination shows splinting on the left side. There is dullness to percussion and egophony over the left lower lobe. Abdominal examination shows no abnormalities. Results of laboratory studies are shown:

Serum	
Calcium	8.4 mg/dL
Urea nitrogen	18 mg/dL
Creatinine	1.4 mg/dL
Na^+	131 mEq/L
K^+	4.1 mEq/L
Cl^-	108 mEq/L
HCO_3^-	25 mEq/L
Blood	
Hematocrit	37%
Hemoglobin	12.4 g/dL
WBC	21,000/mm^3
Neutrophils, segmented	79%
Neutrophils, bands	10%
Lymphocytes	11%
Platelet count	250,000/mm^3
Arterial blood gas analysis on room air:	
pH	7.45
P_{CO_2}	45 mm Hg
P_{O_2}	62 mm Hg
HCO_3^-	24 mEq/L
O_2 saturation	95%

Urinalysis shows no abnormalities. An ECG shows sinus tachycardia. A chest x-ray shows consolidation in the left lower lobe and no cardiomegaly. Intravenous antibiotics are administered, and the patient receives oxygen via nasal cannula. Three hours later, he is lying on his left side and has increased dyspnea; he is rolled to his right side and his symptoms improve within minutes. Which of the following best explains this improvement?

(A) Positionally apparent pulmonary emboli
(B) Positionally decreased alveolar-arterial gradient
(C) Positionally impeded filling of the left ventricle
(D) Positionally impeded movement of the diaphragm
(E) Positionally increased left pleural effusion

2. A 55-year-old woman, gravida 4, para 4, comes to the office because of vaginal spotting that has occurred once monthly for the past 2 months. Menopause occurred 1 year ago, and this is the patient's first postmenopausal examination. Medical history is significant for elevated blood pressure readings during the past 2 years and diabetes mellitus for which she follows an 1800-calorie diet. She takes no medications. Her most recent cervical cytology and mammography obtained 1 year ago disclosed no abnormalities. Family history is significant for hypertension in her mother who died at age 58 years from an unknown cancer. The patient has smoked one pack of cigarettes daily for the past 30 years and drinks beer occasionally. BMI is 42 kg/m^2. Temperature is 36.7°C (98.0°F), pulse is 90/min, respirations are 16/min, and blood pressure is 136/84 mm Hg. Bimanual examination discloses a firm, smooth, mobile, nontender uterus that is consistent in size with a 6-week gestation. The remainder of the physical examination discloses no abnormalities. Results of dipstick urinalysis are within the reference ranges; fingerstick hematocrit is 35% and 2-hour postprandial serum glucose concentration is 188 mg/dL. Which of the following is the most appropriate diagnostic study at this time?

(A) Complete blood count
(B) CT scan of the pelvis
(C) Endometrial biopsy
(D) Hysterosalpingography
(E) Serum CA 125 concentration

3. A 28-year-old woman comes to the health center for an initial visit. The patient appears somewhat anxious. After introductions she says, "Doctor, something has been happening to me for the past 6 weeks or so that has me a bit scared. About once or twice a week, and out of nowhere and for no reason, I have this sudden feeling of anxiety. It really gets severe and I get short of breath. I just don't know what's going on." She has recently divorced her husband of 7 years. Given the evidence at this time, which of the following is the most likely diagnosis?

(A) Adjustment disorder with anxiety
(B) Agoraphobia
(C) Dysthymic disorder
(D) Generalized anxiety disorder
(E) Posttraumatic stress disorder

4. A 71-year-old woman with a 15-year history of severe chronic obstructive pulmonary disease (COPD) is admitted to the intensive care unit for treatment of an acute COPD exacerbation. The patient has been receiving home oxygen therapy for the past 3 years; FEV_1 1 year ago was 27%. She has a do-not-resuscitate order stating that she does not want cardiopulmonary resuscitation, with no specific comment on intubation. Her daughter, who is not present, has power of attorney for the patient's health care decisions, although the patient has made all decisions in the past. Medical history also is significant for hypertension. Medications are lisinopril and hydrochlorothiazide, as well as inhaled tiotropium, albuterol, and fluticasone. The patient is intubated and mechanical ventilation is begun. She is treated with antibiotics, corticosteroids, and nebulized bronchodilators. Her condition gradually improves and she is extubated 4 days later. Within 2 hours of extubation, the patient becomes short of breath and has difficulty clearing her secretions. She is not using accessory muscles of respiration. Temperature is 37.0°C (98.6°F), pulse is 98/min, respirations are 25/min, and blood pressure is 168/98 mm Hg. Oxygen saturation is 89% on oxygen at 6 L/min. The patient is alert, fully oriented, and conversant. Results of arterial blood gas analysis are shown:

PO_2	61 mm Hg
PCO_2	55 mm Hg
pH	7.32

An attempt is made to manage the patient with noninvasive positive pressure ventilation, but the patient reports discomfort caused by the mask and requests that it be removed. When initially asked, she declines to be reintubated. Which of the following is the most appropriate next step in management?

(A) Clearly state to the patient that if she again declines intubation when offered to her one more time, she will receive comfort care only
(B) Contact the patient's daughter to request additional information regarding the patient's previous feelings regarding intubation
(C) Explain to the patient that because her advance directive is confusing, discussing her options would be helpful
(D) Proceed with reintubating the patient
(E) Request psychiatric evaluation of the patient's capacity to make health care decisions

5. A phase 3 trial is planned to investigate the use of a new medication for the prevention of type 2 diabetes mellitus. Which of the following inclusion criteria should be selected to design the most efficient study?

(A) Patients aged 18 years and older who have a BMI of less than 23 kg/m^2 and are smokers
(B) Patients aged 18 years and older with no significant medical history
(C) Patients aged 45 years and older with hyperlipidemia and central obesity
(D) Patients aged 65 years and older with a hemoglobin A_{1c} of 6.5% or greater

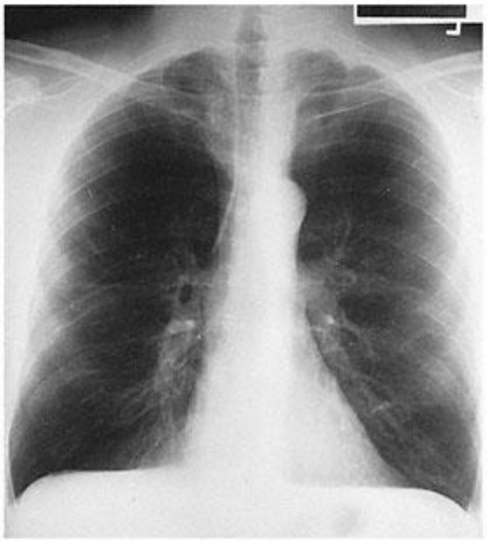

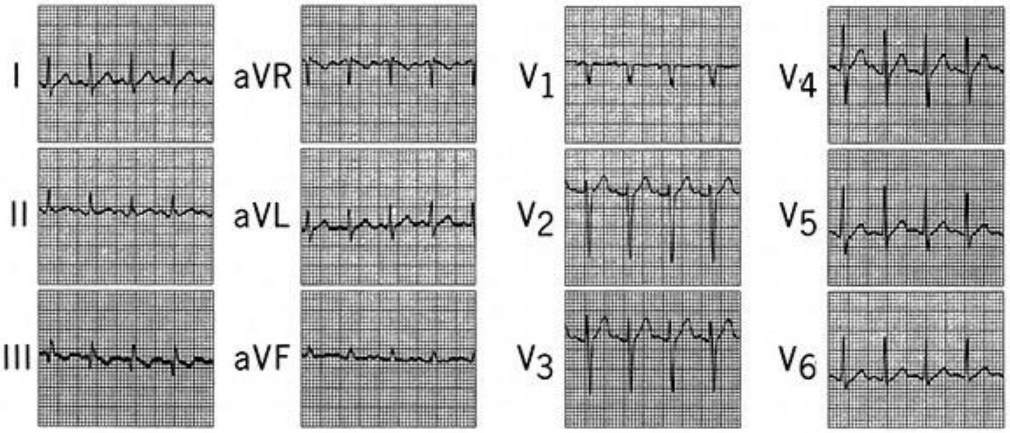

6. A 60-year-old man is admitted to the hospital 4 hours after he awoke early this morning with shortness of breath, dizziness, and pleuritic chest pain. Medical history is significant for coronary artery disease, hyperlipidemia, and chronic obstructive pulmonary disease. Medications are atorvastatin, enteric-coated 81-mg aspirin, atenolol, albuterol, ipratropium, and home oxygen at 1 L/min via nasal cannula. He has not had any recent illness or exposure to anyone known to be ill. He has smoked one pack of cigarettes daily for the past 40 years and he drinks three to four alcoholic beverages monthly. BMI is 21 kg/m^2. Temperature is 36.9°C (98.4°F), pulse is 150/min, respirations are 36/min, and blood pressure is 105/72 mm Hg. Oxygen saturation is 84% on oxygen at 1 L/min via nasal cannula. The patient appears uncomfortable and is speaking in three- to four-word sentences. Jugular venous pressure is 10 cm H_2O at 45 degrees. Auscultation of the lungs discloses poor air movement and diffuse scattered inspiratory wheezes bilaterally. Cardiac examination discloses a palpable parasternal lift. The abdomen is protuberant with a visible fluid wave and a palpable epigastric pulsation. The liver edge is palpated 3 cm below the right costal margin and the area is tender. Examination of the lower extremities shows 2+ bilateral pitting edema. Chest x-ray and ECG are shown. Which of the following is the most likely underlying explanation for this patient's condition?

(A) External compression of the superior vena cava
(B) *Helicobacter pylori* infection with transmural ulceration and peritoneal leaking
(C) Lactic acidosis caused by systemic hypoperfusion
(D) Pulmonary vasculature compromise and cor pulmonale
(E) Widespread hepatocellular necrosis and parenchymal edema

7. A 67-year-old male US military veteran is admitted to the rehabilitation facility 3 days after undergoing uncomplicated revision of his left transtibial amputation to a transfemoral amputation, which was required because of dehiscence of the previous incision. Today, the patient reports pain at the site of incision that he rates as a 3 on a 10-point scale. He reports phantom sensation but no phantom pain. He also has atrial fibrillation, type 2 diabetes mellitus, mild diabetic retinopathy, hypertension, and severe peripheral vascular disease that required aortic to bilateral iliac arterial bypass 2 years ago. Medications are dabigatran, lisinopril, metformin, and metoprolol. The patient is 172 cm (5 ft 8 in) tall and weighs 86 kg (190 lb); BMI is 29 kg/m^2. Temperature is 37.0°C (98.6°F), pulse is 68/min, respirations are 18/min, and blood pressure is 138/88 mm Hg. The patient does not appear to be in distress. Examination shows a well-healing incision. Fingerstick blood glucose concentration is 120 mg/dL. Which of the following findings on further physical examination is most likely to inhibit this patient's long-term ability to ambulate with a prosthesis?

(A) A 3-cm-diameter blackened eschar on the right heel
(B) A 5-degree left hip flexion contracture
(C) An irregular pulse
(D) Loss of proprioception in the right great toe

8. Researchers at a large health sciences center would like to evaluate the relationship between dental x-rays and risk for thyroid cancer. They plan to enroll 300 participants with thyroid cancer and 400 participants without thyroid cancer. All participants will be asked to report past exposure to dental x-rays. The study will be federally funded. Since many of the participants may have received dental x-rays at the institution, a member of the institutional review board (IRB) is concerned about increased liability arising from the results of the study, especially if a positive association between dental x-rays and thyroid cancer is found. Which of the following is the most appropriate response by the IRB regarding the member’s concern?

(A) Approve the study as submitted
(B) Do not approve the study
(C) Require language in the informed consent document advising participants of the right to sue should a positive association between dental x-rays and thyroid cancer be found
(D) Require language in the informed consent document releasing the institution from liability

9. A 39-year-old man comes to the emergency department because of a 1-day history of progressively worsening generalized itchiness of his skin. He also has had pain with urination and blood in his urine during the past 5 days. He has not had fever, chills, nausea, vomiting, flank pain, or diarrhea. Two days ago he was seen in the emergency department and diagnosed with renal calculi. Medical history otherwise is remarkable for psoriasis. His current medications are oxycodone-ibuprofen, tamsulosin, and topical clobetasol. Vital signs are temperature 36.7°C (98.0°F), pulse 76/min, respirations 12/min, and blood pressure 118/74 mm Hg. Examination of the skin shows multiple excoriations over the back and abdomen; no lesions are noted. The remainder of the physical examination discloses no abnormalities. Results of urinalysis are shown:

Specific gravity	1.010 (N=1.003–1.029)
Bilirubin	Trace positive
Blood	3+
Leukocyte esterase	Negative
RBCs	Positive

Which of the following is the most likely cause of this patient's pruritus?

(A) Exacerbation of psoriasis
(B) Obstruction of the common bile duct
(C) Oxycodone therapy
(D) Tamsulosin therapy
(E) Ureteral obstruction

10. A 72-year-old man is admitted to the hospital through the emergency department because of a 12-hour history of fever, chills, increasingly severe abdominal pain, nausea, and vomiting. Medical history includes hypertension, hypercholesterolemia, and atherosclerotic heart disease with placement of a coronary artery stent 3 years ago. Current medications are atorvastatin and lisinopril. He appears anxious and pale. BMI is 30 kg/m^2. Temperature is 40.0°C (104.0°F), pulse is 116/min, respirations are 20/min, and blood pressure is 100/65 mm Hg. Oxygen saturation is 94% on room air. A bruit is heard over the right carotid artery. Cardiac examination discloses no murmurs; an S_4 is present. Lungs are clear to auscultation. Abdominal examination discloses decreased bowel sounds and diffuse tenderness with guarding that is most severe in the left upper quadrant. Both lower extremities are cool to touch with weak pedal pulses. Abdominal x-ray shows dilation of the proximal colon. Results of serum laboratory studies are shown:

Amylase	230 U/L
Urea nitrogen	20 mg/dL
Creatinine	1.0 mg/dL
Na^+	138 mEq/L
K^+	3.5 mEq/L
Cl^-	101 mEq/L
HCO_3^-	12 mEq/L
Glucose	180 mg/dL

Surgical resection of the colon is done. Which of the following histopathologic changes will most likely be identified in the resected colon?

(A) Caseating granulomatous inflammation with serosal fibrous adhesions
(B) Coagulative necrosis involving mucosa and submucosa
(C) Neutrophilic infiltrates in the mucosa with venous congestion and edema
(D) Patchy mucopurulent exudate with exploding glandular crypts
(E) Transmural chronic inflammation with ulcerations extending into submucosa

11. A 37-year-old woman comes to the emergency department because of a 4-hour history of severe right-sided abdominal pain. She describes the pain as sharp and says it radiates to the pelvic region; she rates the pain as a 9 on a 10-point scale. She also has had associated nausea but has not vomited. She has not had any recent trauma, fever, chills, changes in bowel habits, or urinary symptoms. She took ibuprofen 3 hours ago but has had no relief of her current symptoms. Medical history is otherwise unremarkable and she takes no other medications. She does not smoke cigarettes or drink alcoholic beverages. She is not sexually active and her last menstrual period ended 4 days ago. The patient is holding her right side and is grimacing in pain. Temperature is 37.2°C (99.0°F), pulse is 80/min, respirations are 22/min, and blood pressure is 136/74 mm Hg. Auscultation of the chest discloses normal breath sounds and normal S_1 and S_2. Abdominal examination discloses normal bowel sounds, tenderness with voluntary guarding on the right side, but no palpable masses. Pain is elicited on percussion over the right costovertebral angle. Results of laboratory studies are shown:

Serum	
Urea nitrogen	16 mg/dL
Creatinine	0.8 mg/dL
Calcium	9.8 mg/dL
Blood	
Hemoglobin	13.9 g/dL
WBC	13,000/mm^3
Neutrophils	68%
Lymphocytes	27%
Monocytes	3%
Eosinophils	2%
Urine	
Specific gravity	1.010 (N=1.003–1.029)
Occult blood	Trace positive
pH	6.0
Glucose	Negative
Protein	Negative

Intravenous fluids and analgesic therapy are initiated. Which of the following is the most appropriate next step in evaluation?

(A) Cystoscopy
(B) Exploratory laparotomy
(C) Helical CT scan of the abdomen
(D) X-ray of the kidney, ureter, and bladder
(E) No further testing is indicated at this time

12. A 75-year-old woman is brought to the emergency department by her husband 60 minutes after she "fainted" while standing at the bathroom sink. The husband witnessed her fainting spell and says she lost consciousness for approximately 15 to 30 seconds. He says she was alert upon awakening but looked pale. The patient did not have any tonic-clonic movements and sustained no traumatic injuries during the fall. She did not have palpitations or other warning signs prior to losing consciousness and has not had slurred speech or abnormalities of movement upon awakening. Medical history is significant for hypertension and gout. Medications include lisinopril, metoprolol, 81-mg aspirin, and allopurinol. She has no drug allergies. She does not smoke cigarettes or drink alcoholic beverages. The patient is in no acute distress. Temperature is 37.0°C (98.6°F), pulse is 68/min, respirations are 15/min, and blood pressure is 162/74 mm Hg. Standing pulse and blood pressure are 70/min and 172/75 mm Hg, respectively. Pulse and blood pressure after standing for 2 minutes are 68/min and 148/58 mm Hg, respectively. Examination of the neck discloses jugular venous distention, 5 cm at 30 degrees; there are no carotid bruits. Lungs are clear to auscultation. Cardiac examination discloses a regular rhythm with a soft systolic ejection murmur heard best at the left upper sternal border. There is no peripheral edema and pulses are 2+ bilaterally. Neurologic examination and ECG disclose no abnormalities. Serum troponin I concentration is 0.1 ng/mL (N<0.35). Results of the remaining laboratory studies are within the reference ranges. CT scan of the head shows no abnormalities. Which of the following is the most appropriate next step in evaluation?

(A) Electroencephalography
(B) Outpatient ambulatory ECG monitoring (24-hour)
(C) Outpatient echocardiography
(D) Telemetry observation
(E) Tilt test

13. A 52-year-old man comes to the emergency department because of fever and nonpruritic lesions that suddenly developed over his legs 2 hours ago. He has not had any gastrointestinal symptoms. Medical history is significant for type 2 diabetes mellitus and cirrhosis secondary to hemochromatosis. Medications include insulin and spironolactone. Vaccinations are up-to-date. The patient returned home 1 day ago from a vacation in a southeastern state. He has had no known contact with anyone who has been ill. On arrival, temperature is 38.6°C (101.5°F), pulse is 118/min, respirations are 22/min, and blood pressure is 84/50 mm Hg. Oxygen saturation is 93% on room air. Lungs are clear to auscultation. Cardiac examination discloses a grade 2/6 systolic murmur heard best along the left sternal border. Spider angiomas are present over the torso. Abdomen is distended and diffusely dull to percussion. Palpation of the abdomen does not disclose tenderness. The liver and spleen are not palpated. The lower extremities are erythematous and warm to the touch; hemorrhagic bullae are present from the dorsum of the feet to the knees bilaterally. Results of laboratory studies are shown:

Serum		Blood	
ALT	68 U/L	Hematocrit	33%
AST	61 U/L	Hemoglobin	11.0 g/dL
Urea nitrogen	31 mg/dL	WBC	16,000/mm^3
Creatinine	2.1 mg/dL	Neutrophils, segmented	85%
Na^+	127 mEq/L	Neutrophils, bands	4%
K^+	5.0 mEq/L	Lymphocytes	7%
Cl^-	95 mEq/L	Monocytes	4%
HCO_3^-	16 mEq/L	Platelet count	83,000/mm^3
Lactate dehydrogenase	166 U/L		
Glucose	354 mg/dL		

Which of the following microorganisms is the most likely causal agent of this patient's condition?

(A) *Enterobacter aerogenes*
(B) *Enterococcus faecalis*
(C) *Mycobacterium marinum*
(D) *Vibrio vulnificus*

14. A 58-year-old man comes to the office for follow-up of an 8-month history of blood pressure readings ranging between 150 and 180 mm Hg systolic, and 85 and 100 mm Hg diastolic. Medical history is otherwise unremarkable. Medications include 20-mg lisinopril, 100-mg atenolol, 25-mg hydrochlorothiazide, and amlodipine. At the patient's last visit 6 weeks ago, the dose of amlodipine was increased from 5 mg to 10 mg. The patient does not smoke cigarettes and drinks alcoholic beverages socially. BMI is 24 kg/m^2. Temperature is 37.0°C (98.6°F), pulse is 86/min, respirations are 14/min, and blood pressure is 176/102 mm Hg. Physical examination discloses no abnormalities. Results of laboratory studies are shown:

Serum	
Urea nitrogen	18 mg/dL
Creatinine	1.0 mg/dL
Na^+	140 mEq/L
K^+	4.2 mEq/L
Cl^-	105 mEq/L
HCO_3^-	25 mEq/L

Specific additional history should be obtained regarding which of the following?

(A) Caffeine use
(B) Exercise history
(C) Frequency of fast food consumption
(D) Refill patterns on medications

15. A 65-year-old man comes to the office because of a 4-month history of increasingly severe joint pain. Medical history is significant for an acute anterior myocardial infarction 5 years ago. Current medications are metoprolol and 81-mg aspirin. Active and passive motion of the elbow and knee joints elicits pain. A diagnosis of osteoarthritis is made. The patient says that a colleague of his recommended celecoxib to treat his pain. The concerns with adding celecoxib to this patient's medication regimen are related to inhibition of which of the following processes?

(A) Both cyclooxygenase-1 (COX-1) and cyclooxygenase-2 (COX-2) decreasing prostanoid production
(B) COX-1 decreasing prostacyclin (PGI_2) production
(C) COX-1 decreasing thromboxane A_2 production
(D) COX-2 decreasing prostacyclin (PGI_2) production
(E) COX-2 decreasing thromboxane A_2 production

16. The quality improvement department of a hospital has received several incident reports indicating that patients admitted to the hospital have received a different amount of oxygen than was originally ordered by the admitting physician. On admission, the current practice for ordering oxygen therapy is that the admitting physician includes oxygen in the written admission orders. An example of admission orders for oxygen is shown:

Oxygen: 2 liters via nasal cannula with goal of oxygen saturation >90%

After patients are admitted to the hospital, the amount of oxygen a patient receives is based upon physician and nurse discussion, which is not usually documented in the medical record as an additional order. In addition to reviewing the hospital's protocol for oxygen delivery, instituting which of the following measures would most likely decrease discrepancies in oxygen delivery without creating additional risks?

(A) Conducting an in-service program on oxygen use and documentation of orders
(B) Creating a standard set of oxygen orders that includes initial dose, titration parameters, and goal dose
(C) Encouraging the nurses to refrain from adjusting the patient's oxygen dose without a written order from the physician
(D) Requiring nurses to enter verbal orders in the electronic medical record and requiring physician signature within 24 hours
(E) Requiring nurses to telephone a physician prior to any deviation from the original admission order for oxygen therapy

17. A 21-year-old ballet dancer comes to the office because she and her husband would like to conceive. She has not had a menstrual period during the past 8 months. She runs 13 km (8 mi) daily. The patient identifies as White. She is 165 cm (5 ft 5 in) tall and weighs 48 kg (105 lb); BMI is 18 kg/m^2. Temperature is 36.1°C (97.0°F), pulse is 52/min, respirations are 20/min, and blood pressure is 90/70 mm Hg. Physical examination discloses a thin woman. The remainder of the physical examination shows no abnormalities. The patient is advised to gain weight and to decrease the amount of strenuous exercise. She returns for follow-up 3 months later. She says she still has not had a period even though she has gained 2 kg (4.4 lb). Which of the following is the most likely cause of this patient's amenorrhea?

(A) Addison disease
(B) Hypothalamic hypogonadism
(C) Partial hypopituitarism
(D) Polycystic ovarian syndrome
(E) Premature ovarian failure

18. A 19-year-old woman who is at 32 weeks' gestation comes to the clinic for a prenatal visit. She says, "I tried to quit smoking as soon as I knew I was pregnant," but she admits that she has only been able to decrease her smoking to 5 to 10 cigarettes daily. Her partner has continued to smoke during the pregnancy. The patient has a history of cocaine use but her urine screening studies have been negative during this pregnancy. The patient is unemployed and dropped out of school in the 11th grade. She has been receiving supplemental nutritional foods during this pregnancy. She says she plans to bottle-feed this baby because her friends have told her, "Breast-feeding hurts you, and besides, formula makes babies grow faster." Which of the following factors in this patient's history places her infant at greatest risk for sudden infant death syndrome (SIDS)?

(A) Employment status
(B) History of cocaine use
(C) Lack of breast-feeding
(D) Maternal smoking

19. **<u>Patient Information</u>**
Age: 66 years
Gender: F, self-identified
Race/Ethnicity: unspecified
Site of Care: office

The patient presents because of a 3-day history of increasingly severe pain when swallowing solids and liquids. Use of over-the-counter antacids has not resulted in relief. There is no associated nausea, vomiting, or loss of appetite. Medical history is remarkable for hypertension, diet-controlled type 2 diabetes mellitus, migraine, and osteoporosis. Current medications are alendronate, chlorthalidone, propranolol, sumatriptan, and calcium citrate with vitamin D supplements. She has smoked one-half pack of cigarettes daily for 10 years and drinks one to two alcoholic beverages monthly. She does not use other substances. BMI is 24 kg/m^2. Temperature is 37.0°C (98.6°F), pulse is 84/min, respirations are 16/min, and blood pressure is 130/90 mm Hg. Oxygen saturation is 98% on room air. Physical examination shows no abnormalities. Endoscopy is most likely to show ulceration of which of the following structures?

(A) Duodenum
(B) Esophagus
(C) Gastric cardia
(D) Gastric fundus
(E) Pylorus

20. An 11-year-old boy is brought to the office by his parents for a well-child examination and routine vaccinations. His medical history is unremarkable and he receives no medications. Vaccinations were up-to-date at his most recent examination 1 year ago. He is not sexually active, and the family is affiliated with a religion that teaches abstinence. The parents express reluctance about administering the human papillomavirus (HPV) vaccine today because of the potential adverse effects associated with the vaccine. They ask if their son really needs this vaccine today, given that he will not become sexually active until he is much older and married. The patient's vital signs are within normal limits. Physical examination shows no abnormalities. Which of the following is the most appropriate response to the parents?

(A) Acknowledge that there are risks associated with vaccines but emphasize that those associated with the HPV vaccine are small because it is an inactivated vaccine
(B) Advise the parents to vaccinate their son in order to contribute to the elimination of HPV from the general population
(C) Explain that even if their son waits until marriage to have sex, he could still be exposed to HPV by his future partner
(D) Provide the parents with literature about the vaccine and advise them that they may defer the decision because the vaccine may be administered until the age of 26 years

The abstract displayed on these following pages is for use with items #21-23 on page 26.

Question

In patients with cirrhosis and acute bleeding esophageal varices, how do endoscopic sclerotherapy and emergency portacaval shunt compare for control of bleeding and survival?

Methods

Design: Randomized controlled trial (San Diego Bleeding Esophageal Varices Study). ClinicalTrials.gov NCT00690027.

Allocation: Concealed.

Blinding: Blinded (gastroenterologist who evaluated patients for portal-systemic encephalopathy).

Follow-up period: Up to 17 years.

Setting: University of California San Diego Medical Center.

Patients: 211 patients (mean age 49 years, 77% men) with acute bleeding esophageal varices resulting from cirrhosis, who required a transfusion of ≥ 2 units of blood and, for patients transferred from other hospitals, observation of upper gastrointestinal bleeding within 48 hours of transfer. Exclusion criterion was > 1 previous session of endoscopic sclerotherapy.

Intervention: Endoscopic sclerotherapy (n = 106) or emergency portacaval shunt (n = 105). Emergency portacaval shunt comprised a direct side-to-side or direct end-to-side portacaval shunt done within 8 hours of initial contact.

Outcomes: Control of bleeding at > 30 days, survival, readmissions for variceal or nonvariceal bleeding requiring transfusion of packed red blood cells, and recurrent portal-systemic encephalopathy.

Patient follow-up: 100% (minimum follow-up until death or 9.4 years).

Main results

15-year survival was lower with endoscopic sclerotherapy than with emergency portacaval shunt (10/106 vs 48/105, relative benefit reduction 79%, 95% CI 62 to 89; number needed to harm 3, CI 2 to 4). Other main results are shown in the Table.

Endoscopic sclerotherapy (EST) vs emergency portacaval shunt (EPCS) in patients with cirrhosis and acute bleeding esophageal varices

Outcomes	Child risk class	EST	EPCS	*P* value
Control of bleeding at > 30 days*		20%	100%	<.001
Median survival (years)	A	4.62	10.43	.003
	B	2.61	6.19	<.001
	C	0.58	5.30	.005
Mean number of readmissions for variceal bleeding requiring packed red blood cell transfusion		6.8	0.4	<.001
Recurrent portal-systemic encephalopathy†		35%	15%	.001

*Excluding indeterminate deaths at 14 days from nonbleeding causes.

†In patients who survived 30 days and left hospital.

Conclusion

In patients with cirrhosis and acute bleeding esophageal varices, emergency portacaval shunt was better than endoscopic sclerotherapy for control of bleeding, recurrent encephalopathy, and survival.

Sources of funding: National Institutes of Health and Surgical Education and Research Foundation.

Structured abstract based on: Orloff MJ, Isenberg JI, Wheeler HO, et al. **Randomized trial of emergency endoscopic sclerotherapy versus emergency portacaval shunt for acutely bleeding esophageal varices in cirrhosis.** J Am Coll Surg. 2009;209:25-40. 19651060

Items #21–23 refer to the abstract displayed on the previous pages.

21. A 52-year-old man with hepatic cirrhosis comes to the emergency department because of a 3-hour history of vomiting blood. Esophagogastroduodenoscopy confirms actively bleeding esophageal varices. Based on the abstract shown, the physician is considering an emergency portacaval shunt (EPCS) procedure rather than endoscopic sclerotherapy (EST). According to the results in the abstract, approximately how many patients must be treated with EPCS rather than EST to prevent one case of recurrent portal-systemic encephalopathy?

(A) 1
(B) 3
(C) 5
(D) 10
(E) 16

22. Which of the following most strongly limits the generalizability of this study's findings?

(A) The allocation was concealed
(B) EPCS is available only at specialty centers
(C) The follow-up period was too short
(D) The patients were not blinded
(E) Unmeasured confounders were not controlled by the study design

23. Which of the following conclusions is most appropriate based on the results presented in the table?

(A) The 95% confidence interval for the difference in survival between EPCS and EST for Child-Pugh class A patients includes 0 years
(B) EPCS is more effective than EST in decreasing hospital readmissions for variceal bleeding requiring transfusion
(C) The median survival after EPCS is statistically significantly less for Child-Pugh class C than for Child-Pugh class B
(D) The randomization procedure was ineffective in decreasing bias in this study

END OF SET

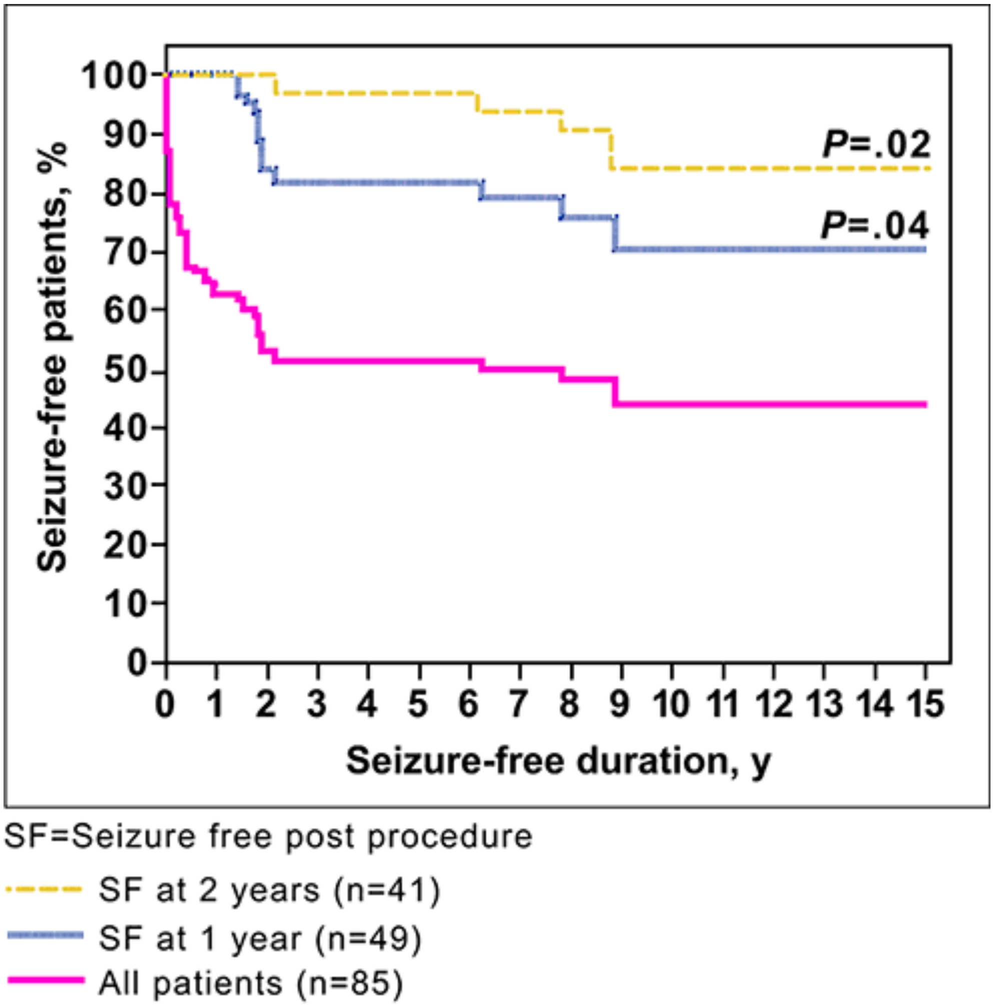

24. An 18-year-old woman with a 5-year history of epilepsy comes to the office with her parents for a follow-up examination. She has sustained three seizures during the past 6 months; all of her seizures originate from the temporal lobe. Anticonvulsant therapy has not decreased the frequency of her seizures. The patient and her parents ask about the potential benefits of partial temporal lobectomy. The physician performs a literature search and finds a published study conducted by a large surgical center evaluating outcomes of the operation. A total of 85 patients who underwent partial temporal lobectomy during a 3-year period were observed for 15 years to determine the number and frequency of seizures sustained postoperatively. Subgroup analyses were conducted of patients who reported no seizures 1 and 2 years postoperatively. A Kaplan-Meier plot of long-term outcomes is shown; the *P*-values provided represent subgroups compared with all other study patients. Which of the following is the most appropriate conclusion to draw from these data regarding seizure recurrence after the procedure?

(A) The absence of seizures 2 years postoperatively is the best predictor of long-term seizure control
(B) The majority of seizure recurrence occurred between 2 and 3 years postoperatively
(C) 30% of patients were seizure free 6 months after the procedure
(D) No conclusion can be determined because the subgroup analysis lacks statistical significance

25. A 2-week-old newborn with trisomy 18 diagnosed at birth is brought to the office by his parents for a follow-up visit. He is unable to suck properly and receives all his nutrition via nasogastric tube. The newborn was born at 37 weeks' gestation to a 29-year-old woman, gravida 2, para 1. Apgar scores were 4 at 1 minute and 8 at 5 minutes. The father is age 30 years. They have a 4-year-old daughter who is healthy. The mother reports that her son has trouble breathing and often stops breathing for up to 20 seconds at a time. He has had no fever, vomiting, or diarrhea. Length is 46 cm (18 in; 5th percentile), weight is 2400 g (5 lb 5 oz; 5th percentile), and head circumference is 32 cm (13 in; 10th percentile). Physical examination shows dysmorphic features with a small jaw and small head, as noted at birth. The patient is listless and his breaths are shallow. During the examination, he has episodes of apnea lasting up to 15 seconds. His tone is increased and he has overlapping fingers and rocker bottom feet. There is a 3/6 systolic murmur heard loudest over the left sternal border. Which of the following is the most appropriate next step?

(A) Admission to the hospital for a sleep apnea study
(B) CT scan of the head
(C) Electroencephalography
(D) Referral to a cardiologist
(E) Referral to home hospice care

26. A 79-year-old retired executive schedules an urgent office visit. When you see him, he says that he had difficulty walking upon getting out of bed this morning because of dizziness, which started last week and worsened last night. He has a 20-year history of hypertension, for which he was treated, but for the past 10 years his blood pressure has been normal without treatment. At his most recent visit 3 weeks ago, his blood pressure was 150/90 mm Hg and he was instructed to adhere to a low-salt diet. He drank a moderate amount of alcoholic beverages in the past and smoked about 16 cigarettes daily from age 20 years to age 50 years. He has not smoked cigarettes during the past 29 years. He is taking no medication at present. All his close relatives have lived to be at least in their 80s. This morning, pulse is 78/min and regular and blood pressure is 160/90 mm Hg. Neurologic examination shows instability when the patient is turning and horizontal nystagmus with change in position. Funduscopic examination shows no abnormalities. Which of the following is the most likely diagnosis?

(A) Cardiac arrhythmia
(B) Cerebellar ataxia due to alcohol abuse
(C) Labyrinthitis
(D) Orthostatic hypotension
(E) Stroke

27. A 78-year-old woman who resides in a nursing care facility has had increasing confusion and drowsiness during the past 10 days. The head nurse says, "The patient is usually alert, talkative, and outgoing, but during the past several days she has become increasingly confused and withdrawn. She drifts off to sleep even during activities that she enjoys." Vital signs have remained normal, and she has been eating well. There has been no change in her medication regimen, which includes pravastatin for hypercholesterolemia, amlodipine for hypertension, a daily 81-mg aspirin, and periodic sublingual nitroglycerin for stable angina pectoris. On interview, the patient says she does not have any symptoms. The patient identifies as White. Temperature is 37.0°C (98.6°F), pulse is 80/min, respirations are 16/min, and blood pressure is 117/84 mm Hg. The patient appears to be drifting in and out of sleep during the physical examination, but she is arousable and not in distress. She is oriented only to person and place. There is a 5-cm ecchymosis without hematoma on the right parietal scalp. Pupils are equal, round, and reactive to light and accommodation. Ocular movements are intact. Optic discs are poorly visualized due to bilateral cataracts. The remainder of the physical examination, including neurologic examination, shows no abnormalities. Results of laboratory studies are shown:

Serum		Blood	
Urea nitrogen	15 mg/dL	Hemoglobin	13.0 g/dL
Creatinine	1.0 mg/dL	WBC	8000/mm^3
Na^+	136 mEq/L	Platelet count	160,000/mm^3
K^+	3.9 mEq/L	Urine	
Cl^-	95 mEq/L	Specific gravity	1.015 (N=1.003–1.029)
HCO_3^-	25 mEq/L	pH	6.5 (N=4.5–7.8)
Glucose	98 mg/dL	Protein	Negative
		Glucose	Negative
		Occult blood	Negative
		Leukocyte esterase	Negative
		Nitrite	Negative

Which of the following is the most likely explanation for the patient's worsening condition?

(A) Drug-drug interaction
(B) Head trauma
(C) Hypothyroidism
(D) Major depressive episode
(E) Viral encephalitis

28. A 12-year-old girl with a seizure disorder is brought to the emergency department 40 minutes after her aunt found her unconscious in her backyard. The aunt reports that the patient is visiting her for the summer. On arrival, the patient is awake but drowsy. She says she is embarrassed because she forgot to bring her anticonvulsant medication from home. She requests that you not call her mother "because she will yell and get angry." Temperature is 37.0°C (98.6°F), pulse is 74/min, respirations are 16/min, and blood pressure is 100/70 mm Hg. Physical examination shows no focal neurologic deficits. The patient requests that she receive one dose of carbamazepine now and that she be given a prescription to fill at the nearby pharmacy. Which of the following is the most appropriate next step?

(A) Administer the dose of carbamazepine and provide a prescription
(B) Administer the dose of carbamazepine but do not provide a prescription
(C) Attempt to contact the mother for permission to treat before proceeding
(D) Do not contact the mother but request that the aunt sign a consent form
(E) Provide a prescription, but do not administer the dose of carbamazepine

29. A 63-year-old man comes to the office because of a 10-minute episode of right eye blindness 2 days ago. The episode resolved spontaneously and there was no associated eye pain. Medical history is remarkable for hypertension and type 2 diabetes mellitus. Medications include hydrochlorothiazide, metoprolol, glipizide, simvastatin, and 81-mg aspirin. He is 183 cm (6 ft) tall and weighs 104 kg (229 lb); BMI is 31 kg/m^2. Vital signs are within normal limits. Physical examination discloses pupils that are equal in size and reactive to light. Visual fields are intact. Visual acuity is 20/20 in both eyes with corrective lenses. Cardiac examination discloses a regular rate and rhythm with no murmurs. Neurologic examination discloses no abnormalities. Which of the following is the most appropriate diagnostic study at this time?

(A) Fluorescein angiography of the right eye
(B) Measurement of intraocular pressures
(C) Transesophageal echocardiography
(D) Ultrasonography of the neck
(E) No further evaluation is indicated

30. A 55-year-old man comes to the office because of a 4- to 6-week history of what he describes as "chest pounding" that worsens when he lies on his back. He also reports occasional episodes of nonexertional chest pain lasting 1 to 2 minutes. He is unable to further characterize the pain. He says that he has had moderate shortness of breath after walking three blocks, but he has not had nocturnal dyspnea. Medical history is remarkable for type 2 diabetes mellitus and gastroesophageal reflux disease. Current medications include simvastatin, metformin, esomeprazole, and 81-mg aspirin. The patient has smoked one pack of cigarettes daily for the past 35 years. Family history is remarkable for myocardial infarction in his father at age 58 years. The patient is 183 cm (6 ft) tall and weighs 91 kg (200 lb); BMI is 27 kg/m^2. Temperature is 36.7°C (98.1°F), pulse is 88/min, respirations are 16/min, and blood pressure is 165/55 mm Hg. There is no jugular venous distention. Carotid pulses are brisk and bounding. Auscultation of the lungs discloses mild, diffuse wheezes. Cardiac examination discloses a point of maximal impulse that is displaced laterally. There is a soft S_1 and S_2; a grade 3/6 blowing murmur is audible at the lower left sternal border that extends through two-thirds of diastole. Systolic and diastolic bruits are heard over both femoral arteries. Results of laboratory studies are shown:

Serum		Blood	
Cholesterol		Hemoglobin	13.0 g/dL
Total	300 mg/dL	WBC	10,500/mm^3
HDL	30 mg/dL		
LDL	180 mg/dL		
Triglycerides	190 mg/dL		
Urea nitrogen	28 mg/dL		
Creatinine	1.4 mg/dL		

ECG shows normal sinus rhythm and left ventricular hypertrophy. Chest x-ray shows an enlarged cardiac silhouette and prominent ascending aorta. Which of the following is the most appropriate additional diagnostic study at this time?

(A) Arterial brachial index of the lower extremity
(B) Coronary angiography
(C) Pulmonary function testing
(D) Renal ultrasonography
(E) Transthoracic echocardiography

31. A 39-year-old woman comes to the office because of a 1-year history of progressively worsening shortness of breath. She had been generally healthy and says that initially the shortness of breath occurred only after strenuous exercise; however, she now becomes fatigued and short of breath after walking two to three blocks. During the past 2 weeks, she has noted a rapid heart rate and light-headedness. She has not had chest pain, lower extremity edema, orthopnea, or any nocturnal symptoms. Medical history is remarkable for Graves disease, which was diagnosed 4 years ago and treated with radioactive iodine. Current medications include levothyroxine, a daily multivitamin, and an oral contraceptive. She does not smoke cigarettes or drink alcoholic beverages. She is 168 cm (5 ft 6 in) tall and weighs 57 kg (125 lb); BMI is 20 kg/m^2. Temperature is 37.0°C (98.6°F), pulse is 120/min and irregular, respirations are 16/min, and blood pressure is 110/85 mm Hg. Lungs are clear to auscultation. Cardiac examination discloses a rapid rate, a palpable parasternal lift, a normal S_1, and a widely split, prominent S_2. There is a grade 2/6 systolic murmur heard best over the second intercostal space. Examination of the lower extremities discloses trace edema of both feet. The remainder of the physical examination discloses no abnormalities. Results of laboratory studies are shown:

Serum		Blood	
Na^+	140 mEq/L	Hemoglobin	13.0 g/dL
K^+	3.4 mEq/L	Platelet count	155,000/mm^3
Cl^-	100 mEq/L		
HCO_3^-	25 mEq/L		
Glucose	130 mg/dL		
TSH	0.58 µU/mL		

ECG shows atrial fibrillation with a ventricular rate of 140/min and right axis deviation. Chest x-ray shows clear lung fields, no effusion, and prominent pulmonary arteries. Which of the following is the most appropriate additional diagnostic study?

(A) CT scan of the chest
(B) Echocardiography
(C) Perfusion lung scan
(D) Pulmonary function testing
(E) Ultrasonography of the thyroid

32. A 53-year-old man is admitted to the hospital for treatment of acute pancreatitis. Temperature is 37.8°C (100.0°F), pulse is 90/min, respirations are 16/min, and blood pressure is 160/90 mm Hg. Oxygen saturation is 94% on room air. There is tenderness and voluntary guarding in the epigastrium with hypoactive bowel sounds. Physical examination otherwise shows no abnormalities. Pneumatic compression devices are in place on both lower extremities. Results of laboratory studies show a leukocyte count of 18,700/mm^3, serum amylase concentration of 540 U/L, and serum lipase concentration of 500 U/L (N<200). Orders are written that the patient is to be given nothing by mouth; intravenous fluids are administered and he is given fentanyl intravenously for pain. On the third day in the hospital he is noted to be diaphoretic with labored breathing. Temperature now is 38.2°C (100.8°F), pulse is 95/min, respirations are 30/min, and blood pressure is 110/70 mm Hg. There are decreased breath sounds at the left lung base. Abdominal examination is unchanged. Which of the following is the most appropriate next step in the evaluation of these new findings?

(A) Chest x-ray
(B) Determination of cardiac enzyme activity
(C) Echocardiography
(D) Ultrasonography of the chest
(E) Ventilation-perfusion lung scans

33. A 24-year-old woman comes to the office because of a 3-month history of intermittent episodes of irritability and insomnia. She says, "I'll be fine for 2 to 3 weeks, but then I get so sensitive to anything my husband says. My mood will last for a few days, then I'm fine again." In addition to insomnia, she has associated headache, fatigue, and overeating during the same period. The patient married 3 months ago, and she says her husband has become frustrated with the unpredictability of her moods. She works as a phlebotomist. Medical history is unremarkable and she takes no prescribed medications. She does not smoke cigarettes or use other substances. She drinks one to two alcoholic beverages weekly. Her last menstrual period was 1 week ago. BMI is 20 kg/m^2. Vital signs are within normal limits and physical examination discloses no abnormalities. At this time, specific additional history should be obtained regarding which of the following?

(A) Carbohydrate intake
(B) Coital frequency
(C) Frequency of physical activity
(D) History of psychological trauma
(E) Stress level
(F) Timing of symptoms

34. A 55-year-old businessman comes to the office for a preemployment examination. The patient's last visit to a physician's office was 10 years ago. Examination at that time disclosed no abnormalities. Medical history is unremarkable, and the patient takes no medications. His father died of coronary artery disease at age 66 years. His 80-year-old mother was once treated for "some type of thyroid disease." The patient has a sedentary lifestyle and says, "My energy level isn't what it used to be." He frequently eats at fast-food restaurants. BMI is 30 kg/m^2. Temperature is 37.0°C (98.6°F), pulse is 82/min, respirations are 14/min, and blood pressure is 142/92 mm Hg. Physical examination discloses obesity but no other abnormalities. Results of fasting laboratory studies are shown:

Serum		Blood	
Cholesterol		Hematocrit	42%
Total	200 mg/dL	WBC	10,500/mm^3
HDL	34 mg/dL		
LDL	160 mg/dL		
Triglycerides	249 mg/dL		
Creatinine	1.1 mg/dL		
Glucose	126 mg/dL		

Results of serum liver function tests are within the reference ranges. Chest x-ray and ECG disclose no abnormalities. Determination of which of the following is the most appropriate next step in evaluation?

(A) Arterial blood gas values
(B) Hemoglobin A_{1c}
(C) 3-Hour glucose tolerance test
(D) Serum cortisol concentration
(E) Serum fructosamine concentration

35. An 8-year-old girl is brought to the emergency department via ambulance 1 hour after the onset of a generalized tonic-clonic seizure. Paramedics noted a pulse of 50/min followed by a 30-minute period of asystole. En route, the patient was intubated and oxygen therapy and an epinephrine inotropic drip were initiated. On arrival, the patient is unconscious. Temperature is 36.7°C (98.0°F), pulse is 40/min, respirations are 16/min, and blood pressure cannot be obtained. Oxygen saturation is 99% on an FIO_2 of 1.0. Physical examination shows mildly dysmorphic facies. Pupils are sluggish in response to light. Lungs are clear to auscultation with ventilated sounds. Cardiac examination discloses a regular rhythm and sinus bradycardia. Abdomen is soft and nontender. Neurologic examination discloses paralysis. Distal pulses are 1+ and weak. Echocardiography shows global hypokinesis with lateral inferior wall and septal akinesis. Left ventricular function is 20%. The patient is admitted to the hospital. Four days later, there has been no improvement in her condition. Life support is withdrawn, and the patient dies. At autopsy, examination of the heart shows evidence of an acute myocardial infarction. Which of the following is the most likely underlying cause of this patient's death?

(A) Cardiac conduction abnormality
(B) Coronary artery plaque rupture
(C) Increased pulmonary vascular resistance
(D) Systolic prolapse of mitral valve
(E) Thickened left ventricular wall and contractile dysfunction

36. A 2-month-old boy is brought to the office because of a 3-day history of yellow-tinged eyes and skin. He was delivered at 41 weeks' gestation. Pregnancy and delivery were uncomplicated. At delivery, length was 54 cm (21.3 in; 90th percentile), weight was 4200 g (9 lb 4 oz; 90th percentile), and head circumference was 37 cm (14.6 in; 90th percentile). Apgar scores were 9 and 10 at 1 and 5 minutes, respectively. He has been formula-fed and feeds every 3 to 4 hours without spitting up or emesis. He has pale bowel movements two to three times daily. He receives no medications. Current length is 60 cm (23.6 in; 75th percentile), weight is 5.7 kg (12 lb 9 oz; 65th percentile), and head circumference is 41 cm (16.1 in; 90th percentile). Temperature is 36.9°C (98.4°F), pulse is 130/min and regular, respirations are 40/min, and blood pressure is 105/65 mm Hg. Physical examination shows diffuse jaundice and conjunctival icterus. Cardiopulmonary examination discloses no abnormalities. The abdomen is soft, and there is mild hepatomegaly. Results of serum studies are shown:

ALT	265 U/L (N=12–45)
AST	280 U/L (N= 22–63)
Bilirubin, total	9.1 mg/dL (N<1.0)
Direct	6.6 mg/dL (N<0.3)
Protein, total	6.6 g/dL (N=4.6–7.9)
Albumin	4.5 g/dL (N=1.9–4.9)

Abdominal ultrasonography shows a hypoplastic gallbladder and small common bile duct. Which of the following changes is most likely occurring in this patient's liver at this time?

(A) Bile ductular proliferation
(B) Centrilobular necrosis
(C) Increased glycogen stores
(D) Intranuclear hepatocyte inclusions
(E) Macrovesicular steatosis

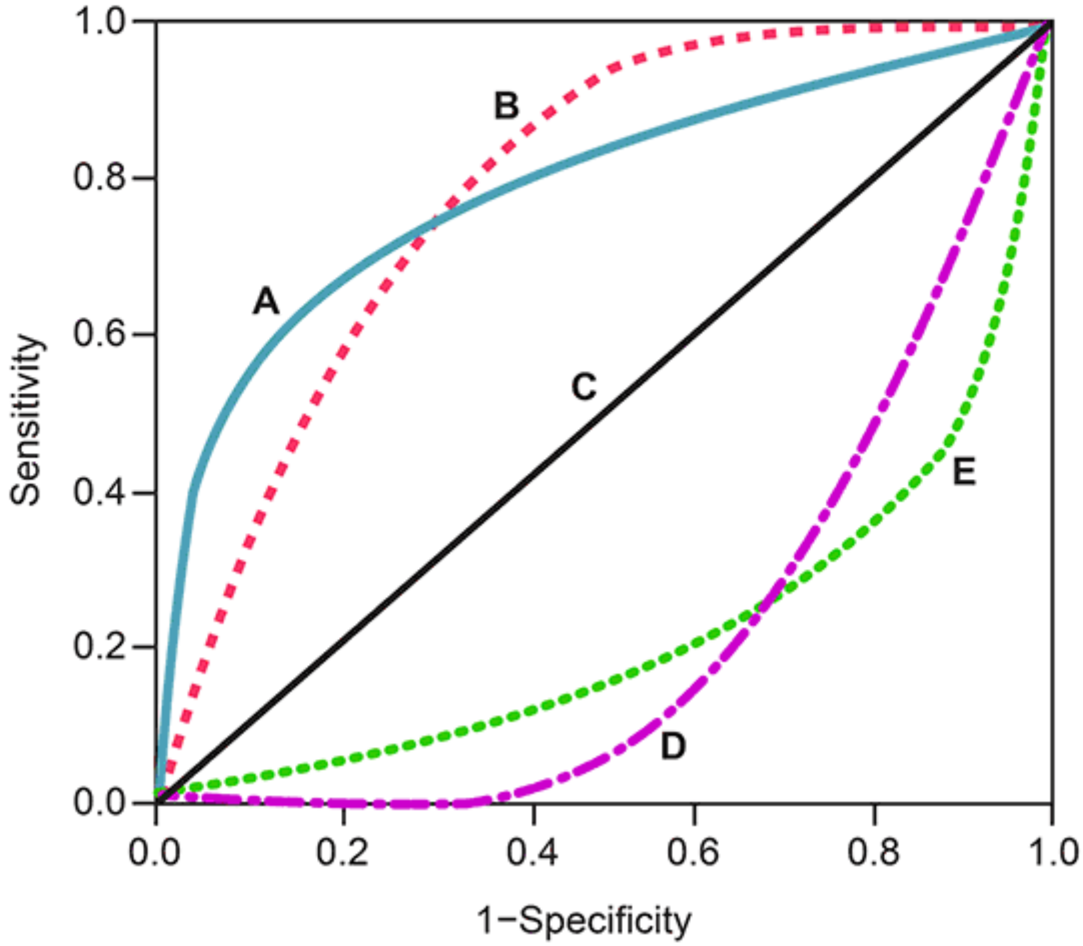

37. A 24-year-old primigravid woman comes to the clinic for her first prenatal visit. Her last menstrual period was 6 weeks ago. Medical history otherwise is unremarkable and her only medication is a prenatal vitamin. Family history is remarkable for gestational diabetes mellitus in her maternal aunt. The patient's vital signs are within normal limits. Physical examination discloses no abnormalities. The patient tells the physician that she would like to be screened for gestational diabetes mellitus and wants to make sure the test will not yield a false-negative result. Several screening tests are available and each test's performance is depicted in the graph shown. Based on these data, which of the following screening tests is the most appropriate choice for this patient?

(A) A
(B) B
(C) C
(D) D
(E) E
(F) Cannot be determined from the data provided

38. A 2-year-old girl is brought to the office by her father because of a 2-day history of a painful mass over the right side of her neck that has been increasing in size. The father says she cries when her neck is touched. She also has a 1-day history of a temperature of 38.1°C (100.6°F). The pain and fever have been well controlled with acetaminophen and ibuprofen. The patient has no history of serious illness and receives no other medications. Her maternal first cousin had non-Hodgkin lymphoma, and her paternal aunt had acute myelogenous leukemia. Examination and appropriate testing are done, and the patient is diagnosed with lymphadenitis. The physician informs the patient's father of the diagnosis and prescribes amoxicillin-clavulanate therapy. The patient's father questions the diagnosis and asks that his daughter be referred to a specialist. In addition to expressing empathy, which of the following is the most appropriate physician response?

(A) "I don't think she needs a specialist. What are you worried about?"
(B) "I know you're worried because of your family history of cancer, but I'm confident in the diagnosis and see no need to refer your daughter to a specialist."
(C) "Tell me what your greatest concerns are."
(D) "There's no need for your daughter to see a specialist. She'll feel better once she completes this course of antibiotics."
(E) "Why do you think that your daughter needs to see a specialist?"

NOTE: THIS IS THE END OF BLOCK 1.
ANY REMAINING TIME MAY BE USED TO CHECK ITEMS IN THIS BLOCK.

Block 2: FIP
Items 39–77; Time - 1 hour

ALL ITEMS REQUIRE SELECTION OF ONE BEST CHOICE.

39. A 68-year-old man is brought to the emergency department by his wife 2 hours after he collapsed into a chair and lost consciousness for 30 seconds. The wife says that during the 10 minutes prior to collapsing, the patient had nausea and two episodes of vomiting. The patient's medical history is significant for hypertension, hyperlipidemia, and coronary artery disease. Medications are metoprolol, 81-mg aspirin, and simvastatin. BMI is 28 kg/m^2. The patient is awake and fully oriented. Temperature is 37.3°C (99.2°F), pulse is 104/min, respirations are 24/min, and blood pressure is 163/94 mm Hg. Oxygen saturation is 95% on room air. Physical examination discloses drooping of the right side of the patient's face. Muscle strength is 3/5 in the left upper extremity and 4/5 in the left lower extremity; strength in the right extremities is normal. Gait is ataxic. The patient has difficulty swallowing when trying to drink a cup of water. This patient's symptoms are most consistent with injury to the brain in the area supplied by which of the following cerebral arteries?

(A) Anterior
(B) Internal carotid
(C) Middle
(D) Posterior
(E) Vertebrobasilar

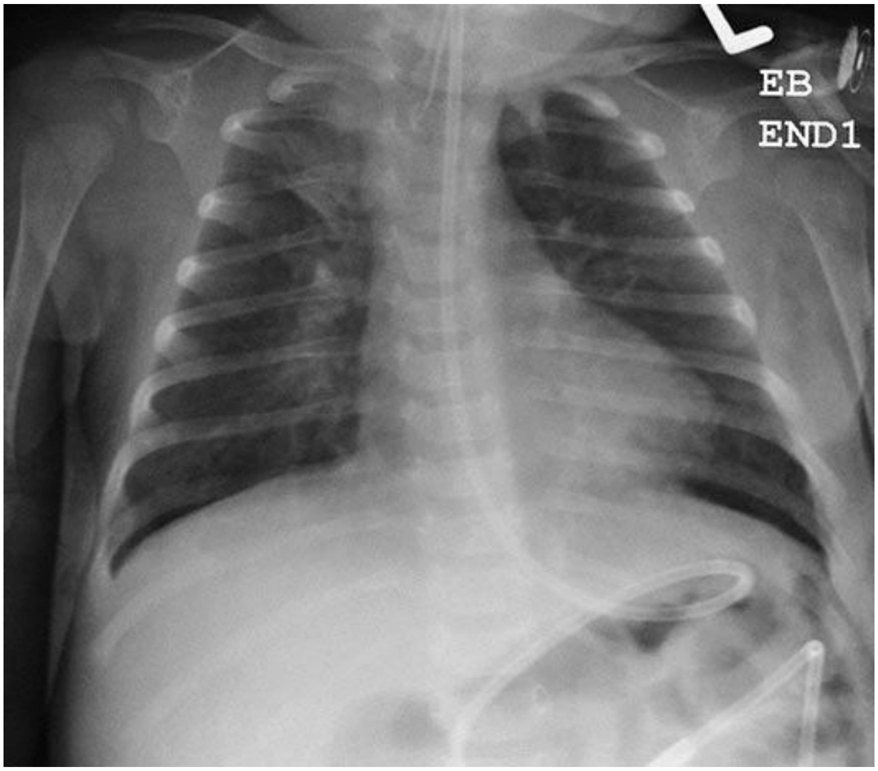

40. A 3-month-old female infant is admitted to the intensive care unit via the emergency department in winter because of respiratory distress. The infant first became ill 3 days ago with fever and congestion, but her symptoms have worsened since that time. This morning she developed pallor, lethargy, cough, copious respiratory secretions, and tachypnea. On arrival at the emergency department today, temperature was 37.1°C (98.8°F), pulse was 124/min, respirations were 80/min, and blood pressure was 80/35 mm Hg. Oxygen saturation was 78% on room air; after administration of supplemental oxygen, oxygen saturation increased to 94%. The infant's pallor has not subsided, and she is in obvious respiratory distress. Physical examination discloses head bobbing, nasal flaring, and subcostal retractions with each breath. Auscultation of the lungs discloses crepitant crackles throughout both lung fields with scattered expiratory wheezing. The remainder of the physical examination discloses no abnormalities. The infant is prepared for intubation based on her clinical status. Results of laboratory studies are shown:

Capillary blood gas analysis on oxygen at 2 L/min	
P_{O_2}	55 mm Hg (N>90)
P_{CO_2}	64 mm Hg (N=26.4–41.2)
pH	7.22 (N=7.35–7.45)
HCO_3^-	26 mEq/L

Blood	
Hematocrit	30%
Hemoglobin	10.1 g/dL
WBC	9800/mm^3
Neutrophils, segmented	18%
Lymphocytes	80%
Platelet count	220,000/mm^3

Chest x-ray is shown. Which of the following is the most likely cause of this infant's illness?

(A) *Haemophilus influenzae*
(B) Herpes virus
(C) *Mycoplasma pneumoniae*
(D) Respiratory syncytial virus
(E) *Streptococcus pneumoniae*

41. A 44-year-old woman, who is a known patient, comes to the office for a Pap smear. She says she has felt fine but has noted a significant increase in the amount of bleeding with her menstrual periods. She says, "My periods are still regular but they now last 10 days instead of 6. It seems the flow is very heavy for at least 5 of the 10 days. With periods like this, I am anxious for menopause." She also reports frequent fatigue and occasional insomnia, but she attributes these symptoms to having three teenaged children. She has not had dysmenorrhea, dyspareunia, or vaginal discharge. Medical history is unremarkable. She does not use any medications and says that she does not like to take pills. Family history is positive for colonic polyps. The patient has been separated from her husband for 5 years. She is occasionally sexually active and always uses condoms. Pelvic examination today discloses no abnormalities except for an enlarged uterus, approximately 8 weeks' gestational size, which is unchanged from her previous examination. Ultrasonography 2 years ago disclosed a myomatous uterus. Cervical cytology and appropriate cultures are obtained, and complete blood count and serum lipid profile are ordered. Which of the following is the most appropriate diagnostic study at this time?

(A) Colposcopy
(B) Determination of serum follicle-stimulating hormone and luteinizing hormone concentrations
(C) Determination of serum prolactin concentration
(D) Endometrial biopsy
(E) Hysteroscopy

42. A 58-year-old man, who is recovering in the hospital 2 days after an uncomplicated elective right total knee arthroplasty, suddenly develops left-sided chest pressure without radiation. The patient was able to ambulate with assistance earlier today. Medical history is significant for hypertension, type 2 diabetes mellitus, dyslipidemia, and osteoarthritis. His routine medications are enalapril, metoprolol, metformin, and rosuvastatin; since admission his routine medications have been continued except for metformin, which has been replaced with sliding-scale insulin. Enoxaparin was added for deep venous thrombosis prophylaxis. The patient is diaphoretic. Temperature is 37.0°C (98.6°F), pulse is 100/min, respirations are 20/min, and blood pressure is 90/60 mm Hg. Oxygen saturation is 92% on room air. Jugular venous pressure is 15 cm H_2O. Lungs are clear to auscultation. Cardiac examination discloses an S_3 and a grade 2/6 murmur heard best at the right second intercostal space with no radiation. ECG shows sinus tachycardia with 2-mm ST-segment elevations in leads II, III, aVF, V_1, and V_2. Intravenous heparin, 325-mg aspirin, and sublingual nitroglycerin are administered, after which the patient's systolic blood pressure immediately decreases to 72 mm Hg. Which of the following is the most likely cause of this patient's symptoms?

(A) Acute mitral regurgitation
(B) Pericardial tamponade
(C) Pulmonary embolism
(D) Right ventricular infarction
(E) Vasovagal reaction

43. A study is conducted to assess physician use of β-adrenergic blocking agents as treatment for heart failure. A random sample of 5000 practicing physicians is selected and surveyed using a 10-item validated questionnaire. Researchers stratify the current prescribing patterns by age of physician, and by location and type of practice. Which of the following most accurately characterizes this study design?

(A) Case-control study
(B) Case series
(C) Clinical trial
(D) Cross-sectional study
(E) Prospective cohort study
(F) Retrospective cohort study

44. A 28-year-old man comes to the office because of a 3-day history of increasing pain on urination. He says, "I'm worried that I might have a sexual disease." He has been married for the past 7 years, and he states that his only sexual relations since then have been with his wife. Medical history is unremarkable and the patient takes no medications. He works at a gardening center. Vital signs are within normal limits. Abdominal examination discloses shotty bilateral inguinal lymphadenopathy. Examination of the penis discloses a minimal amount of watery urethral discharge. The testes appear normal. The remainder of the physical examination shows no abnormalities. Which of the following is the most appropriate diagnostic study at this time?

(A) Gram stain of urethral discharge before and after prostatic massage
(B) Polymerase chain reaction test for *Neisseria gonorrhoeae* and *Chlamydia trachomatis*
(C) Urinalysis
(D) Urine culture and sensitivity
(E) No study, pending reevaluation of sexual history

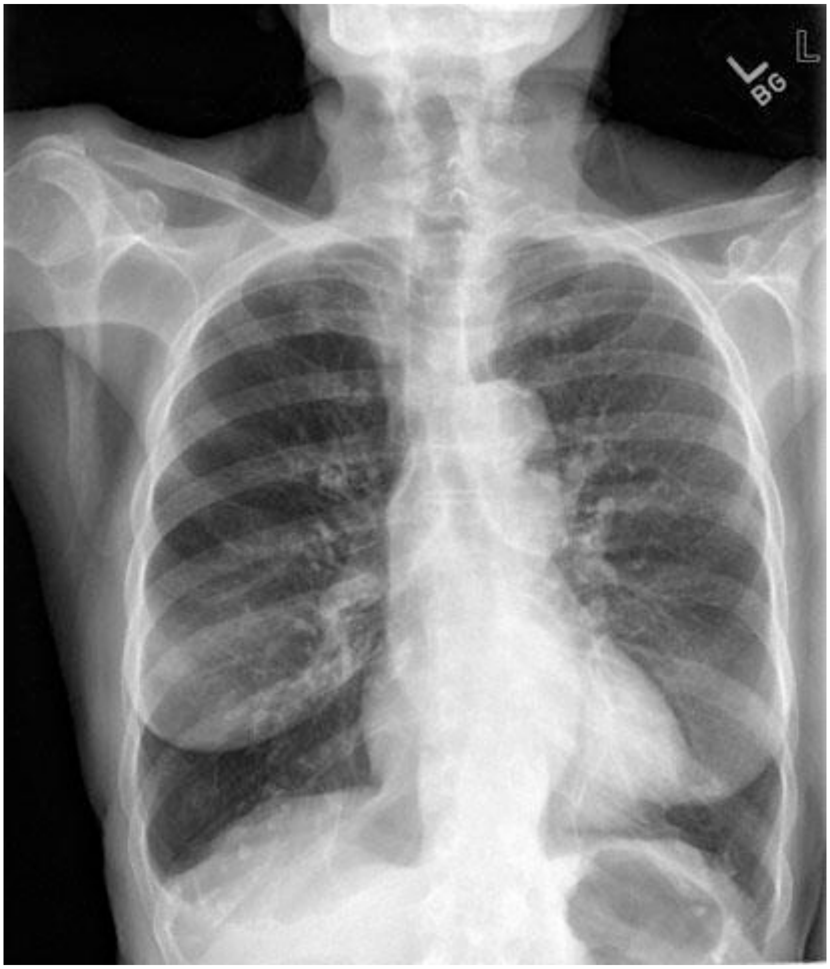

45. A 72-year-old woman comes to the office because of a 1-year history of worsening shortness of breath and occasional chest tightness and nonproductive cough. Her symptoms have varied in intensity but she has had several periods of worsening symptoms during the past 6 months. Medical history is unremarkable and she takes no medications. She has smoked 1½ packs of cigarettes daily for the past 50 years; she does not drink alcoholic beverages. She works as a florist. BMI is 22 kg/m^2. Vital signs are within normal limits. The patient is not in respiratory distress. Auscultation of the lungs discloses a prolonged expiratory phase. Cardiac examination discloses no abnormalities. There are no rashes or lesions. Results of spirometry done in the office are shown:

	**Baseline**	**Post Bronchodilator**
FEV_1	60% of predicted	65% of predicted
FVC	82% of predicted	82% of predicted
FEV_1/FVC ratio	0.55	0.62

Chest x-ray is shown. Which of the following is the most likely underlying cause of this patient's condition?

(A) Bacterial colonization of large- and medium-sized airways
(B) Bronchial constriction in response to environmental allergens
(C) Destruction of alveolar walls leading to enlargement of air spaces
(D) Diffuse patchy interstitial inflammation and fibrosis
(E) Eosinophilic infiltration of the pulmonary interstitium

46. **Patient Information**
Age: 16 years
Gender: M, self-identified
Race/Ethnicity: unspecified
Site of Care: office

The patient is brought for a well-child visit. The patient's mother is concerned because his grades have declined from B's and C's to C's and D's during the past year. She says he has been having difficulty concentrating in school and asks the physician to write a prescription for methylphenidate. Medical history is unremarkable and the patient takes no medications. The mother has a history of major depressive disorder treated with sertraline. The patient's maternal grandfather, uncle, and cousin have alcohol use disorder. The physician asks the patient's mother to leave the examination room. When alone with the physician, the patient says he has smoked cannabis at least three times weekly during the past 6 months. He often smokes with friends in the morning before school starts. He does not drink alcoholic beverages or take prescription drugs or other substances. Physical examination shows no abnormalities. On mental status examination, he reports no depressive symptoms or thoughts of harming himself. Urine toxicology screening is positive for Δ^9-tetrahydrocannabinol. After the examination, the patient becomes distressed and says, "You cannot tell my mother about the marijuana. This is confidential." Which of the following is the most appropriate initial physician response?

(A) "I am worried about your cannabis use, so I recommend that you decrease your cannabis use and see me again in 4 weeks for a repeat urine test."
(B) "I can give you a telephone number for enrollment in a substance abuse treatment program."
(C) "Cannabis is bad for your brain, so let me tell you how 12-step programs can help you stop using cannabis."
(D) "Because you are not actually unsafe, this discussion is between the two of us."
(E) "We need to discuss how cannabis affects your functioning. How can I help you tell your mother?"

47. A 77-year-old man is admitted to the hospital because of a 2-week history of gangrene of the right foot. Three years ago, he underwent a left transmetatarsal amputation. During hospitalization, he required intubation and mechanical ventilation for 2 weeks because of sepsis. He then spent 2 months in an acute rehabilitation facility. Medical history also is remarkable for type 2 diabetes mellitus, peripheral vascular disease, and hypertension. His medications are insulin, irbesartan, hydrochlorothiazide, and aspirin. He is widowed, lives alone, and keeps in contact with his two sons. He has a living will, and his younger son is designated health care proxy. The patient is alert and fully oriented. Temperature is 38.4°C (101.1°F), pulse is 90/min and regular, respirations are 12/min, and blood pressure is 156/90 mm Hg. Oxygen saturation is 96% on room air. There is a 5 × 6-cm ulcer with black margins over the plantar surface of the right foot; malodorous, white-green exudate; and visible bone in the center of the ulcer. Popliteal fossa pulses are decreased and posterior tibial pulses are absent bilaterally. Antibiotic therapy is begun. The patient is informed that he requires amputation of the right leg below the knee. He declines amputation, saying, "I don't ever want to go through what I went through before with my left foot." He is told that the potential risks for not proceeding with amputation are loss of the entire right lower extremity and death. He still refuses the procedure. Which of the following is the most appropriate action for this physician to take?

(A) Abide by the patient's wishes
(B) Consult the hospital bioethics department
(C) Obtain consent for amputation from both sons
(D) Obtain consent for amputation from the courts
(E) Obtain consent for amputation from the younger son
(F) Review any additional instructions in the patient's living will

48. A 63-year-old woman is evaluated in the hospital 2 days after she was admitted for management of left sternal chest pain and a hypertensive emergency with a blood pressure of 195/110 mm Hg. Workup included CT angiography of the chest, which showed no evidence of pulmonary embolism. During the past 6 hours, she has had decreased urine output. A bladder catheter yielded only 50 mL of urine during the past 3 hours. She has not had abdominal pain. Medical history is remarkable for hypertension, osteoarthritis, and stage 3 chronic kidney disease with a baseline serum creatinine concentration of 1.7 mg/dL. Routine medications are lisinopril, amlodipine, and hydrochlorothiazide. The chest pain she had on admission has resolved. Temperature is 37.6°C (99.7°F), pulse is 87/min, respirations are 18/min, and blood pressure is 147/87 mm Hg. Physical examination discloses no abnormalities. Results of laboratory studies obtained on admission and today are shown:

	**On Admission**	**Today**
Serum		
Na^+	138 mEq/L	141 mEq/L
K^+	4.2 mEq/L	5.1 mEq/L
Cl^-	119 mEq/L	118 mEq/L
HCO_3^-	23 mEq/L	21 mEq/L
Urea nitrogen	26 mg/dL	26 mg/dL
Creatinine	1.6 mg/dL	2.4 mg/dL
Glucose	106 mg/dL	108 mg/dL
Urine		
Specific gravity	–	1.010 (N=1.003–1.029)
Protein	–	Negative
Blood	–	Negative
Leukocyte esterase	–	Negative
WBCs	–	0–3/hpf
RBCs	–	0–3/hpf
Casts	–	Muddy brown

Which of the following factors in this patient's history most increased her risk for developing her current complication?

(A) Bladder catheterization
(B) Chronic kidney disease
(C) Uncontrolled hypertension
(D) Use of lisinopril
(E) Volume depletion

49. A randomized placebo-controlled trial is conducted to assess the efficacy of duloxetine in patients with severe rheumatoid arthritis who are currently treated with methotrexate. Patients who have six or more joints that are actively inflamed, swollen, or tender to palpation are eligible for inclusion in the study. The primary outcome measure is the number of joints that are no longer inflamed after 4 months of treatment. One hundred fifty patients are enrolled and randomized to the duloxetine group (n=75) or the placebo group (n=75). The mean number of involved joints is 12. Data show that patients in the intervention group have a mean of seven joints that are no longer inflamed at the end of the trial; patients in the placebo group have a mean of two joints that are no longer inflamed at the end of the trial. Results of a two-tailed *t* test show *P*=.02. Which of the following is the most appropriate conclusion to draw regarding the clinical and statistical significance of this study?

	Clinically Significant	Statistically Significant
(A)	No	no
(B)	No	yes
(C)	Yes	no
(D)	Yes	yes

---

50. A physician would like to evaluate the benefit of a new osteoporosis medication in preventing fractures in women aged 40 to 60 years. A total of 300 women aged 40 to 60 years with osteoporosis documented by DEXA scan are enrolled in the study from general gynecology clinics. The patients are equally randomized into two groups. Group A receives the new osteoporosis medication and Group B receives placebo. The patients are followed for 5 years and bone density is measured annually by DEXA scanning. Results show that the bone density of patients who received the study medication is unchanged and the bone density in patients who received placebo decreased by 2% (*P*=.01). The physician concludes that the new medication decreases the risk for fracture in women aged 40 to 60 years. Which of the following is the most appropriate conclusion to draw from these data?

(A) The impact of the medication on bone density may not correlate with the risk for fracture
(B) The lack of a change in bone density among patients who received the study medication suggests the presence of a confounding variable
(C) The lack of a change in bone density among the treatment group suggests that the medication regimen is too complicated
(D) The lack of women younger than 40 years of age makes it difficult to evaluate the long-term benefit of treatment
(E) Selection bias is likely because the patients were all selected from general gynecology practices

51. A 15-year-old boy is brought to the clinic after his father noticed significant swelling of the boy’s chest when he was getting dressed 6 hours ago. The patient reports a 3-month history of gradually worsening left-sided chest pain and swelling, but he says he was too embarrassed to tell his parents. He has not had fever, chills, night sweats, loss of appetite, weight loss, or any recent trauma to the chest. Medical history is unremarkable and he takes no medications. The patient is a good student. He does not smoke cigarettes or drink alcoholic beverages. Temperature is 36.4°C (97.5°F), pulse is 69/min, respirations are 14/min, and blood pressure is 115/70 mm Hg. Oxygen saturation is 100% on room air. Examination of the head, neck, and skin discloses no abnormalities. There is a fixed palpable mass measuring approximately 14 × 12 cm in the left chest wall that is tender to palpation. The remainder of the physical examination discloses no abnormalities. Biopsy of this mass is most likely to show which of the following?

(A) Acid-fast bacilli
(B) Gram-positive cocci in clusters
(C) Infiltrating nests of squamous cells and increased keratin
(D) Malignant syncytiotrophoblasts and cytotrophoblasts
(E) Poorly differentiated anaplastic spindle cells

52. A 4-year-old boy is brought to the office by his mother because of a 2-kg (5-lb) weight loss, decreased appetite, and daily fatigue during the past 2 months. The mother says the patient's fatigue has limited his physical activity. The patient's medical history is remarkable for asthma and seasonal allergies. He has had three asthma exacerbations during the past 2 months that have required treatment with oral prednisone. Routine medications are cetirizine, albuterol, and budesonide. The patient is 99 cm (3 ft 3 in; 25th percentile) tall and weighs 14 kg (31 lb; 25th percentile); BMI is 14.3 kg/m^2 (10th percentile). Temperature is 36.7°C (98.0°F), pulse is 70/min, respirations are 18/min, and blood pressure is 80/50 mm Hg. The patient appears lethargic. Physical examination discloses no abnormalities. Results of laboratory studies are shown:

Serum		Blood	
Creatinine	0.8 mg/dL (N=0.03–0.59)	Hemoglobin	12.0 g/dL (N=11.5–14.5)
Na^+	125 mEq/L	WBC	10,000/mm^3
K^+	4.5 mEq/L	Platelet count	250,000/mm^3
Cl^-	90 mEq/L	Urine	
HCO_3^-	25 mEq/L	Specific gravity	1.025 (N=1.003–1.029)
Glucose	60 mg/dL (N=60–100)	Protein	Negative
		Blood	Negative
		Leukocyte esterase	Negative
		Nitrite	Negative

Which of the following is the most likely underlying cause of this patient's current condition?

(A) Decreased activity of 21-hydroxylase enzyme
(B) Decreased production of adrenocorticotropic hormone (ACTH)
(C) Decreased production of antidiuretic hormone
(D) Increased aldosterone production
(E) Increased insulin production

53. A 38-year-old woman is brought to the emergency department because of a 4-hour history of confusion and lethargy. Medical history is remarkable for major depressive disorder and a 6-month history of moderate low back pain that began after she lifted heavy boxes during a move. Her medications are fluoxetine daily and acetaminophen with codeine as needed. Family history is remarkable for hypertension and congestive heart failure in her mother. The patient lives with her husband and her mother. The patient drinks two glasses of wine on weekends. On arrival, she is somnolent but arousable to sternal rub. Temperature is 36.4°C (97.5°F), pulse is 32/min, respirations are 16/min, and blood pressure is 74/36 mm Hg. Oxygen saturation is 100% on room air. Pupils measure 6 mm in diameter. Examination shows no other abnormalities. Results of laboratory studies are shown:

Serum		Blood	
ALT	180 U/L	Hemoglobin	12 g/dL
AST	150 U/L	WBC	12,000/mm^3
Creatinine	2.5 mg/dL	Platelet count	400,000/mm^3
Acetaminophen	negative		
Urine toxicology			
Opiates	positive		

An overdose of a drug with which of the following mechanisms of action is the most likely cause of this patient’s condition?

(A) Agonism of μ-opioid receptors
(B) Inhibition of aldosterone
(C) Inhibition of angiotensin-converting enzyme
(D) Inhibition of β_1-adrenergic receptors
(E) Inhibition of central nervous system neuron serotonin reuptake
(F) Inhibition of prostaglandin synthesis
(G) Inhibition of renal sodium and chloride resorption

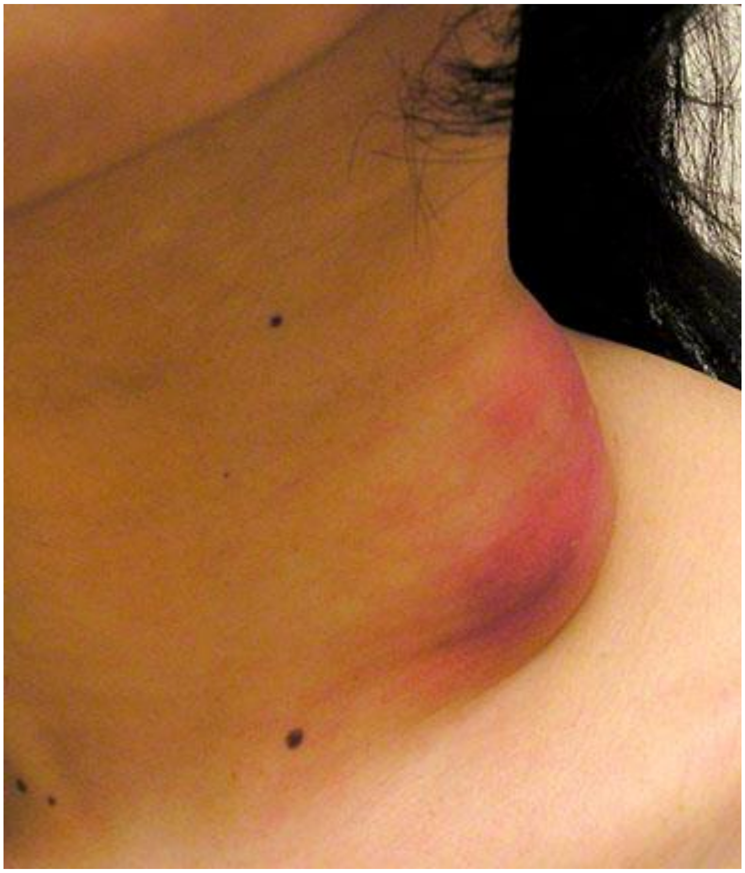

54. A 56-year-old woman comes to the office because of a 1-week history of fever and moderate pain and swelling of the left side of her neck. Medical history is remarkable for a 4-year history of HIV infection. She has declined antiretroviral therapy and takes no medications. BMI is 20 kg/m^2. Temperature is 38.3°C (100.9°F); other vital signs, including oxygen saturation on room air, are within normal limits. Physical examination discloses warmth and moderate tenderness over the swelling shown in the photograph. Lungs are clear to auscultation. There is no rash or hepatosplenomegaly. CD4+ T-lymphocyte count is 160/mm^3, and plasma HIV viral load is 56,000 copies/mL. Pathologic examination of a biopsy specimen of a cervical lymph node shows 2+ acid-fast bacilli, and result of a nucleic acid amplification test is positive for a drug-sensitive mycobacterium. The infection in this patient most likely reached the cervical lymph nodes through which of the following routes?

(A) Direct inoculation via the skin
(B) Direct tissue invasion from the hilar lymph nodes
(C) Hematogenous spread from the lungs
(D) Lymphatic drainage from pharyngeal tissues
(E) Trafficking of infected lymphocytes

55. **Patient Information**

Age: 64 years
Gender: F, self-identified
Ethnicity: unspecified
Site of Care: office

History

Reason for Visit/Chief Concern: "I can't catch my breath even when I'm sitting still."

History of Present Illness:
- shortness of breath at rest for 12 hours

Past Medical History:
- 15-year history of chronic obstructive pulmonary disease (COPD)
- inguinal hernia repair 1 week ago

Medications:
- albuterol

Allergies:
- no known drug allergies

Psychosocial History:
- does not smoke cigarettes or drink alcoholic beverages

Physical Examination

Temp	Pulse	Resp	BP	O_2 Sat	Ht	Wt	BMI
36.7°C (98.0°F)	110/min	24/min	140/80 mm Hg	86% on RA	168 cm (5 ft 6 in)	63 kg (140 lb)	23 kg/m^2

- Appearance: in acute distress; bilateral sternocleidomastoid muscles and intercostal muscles are retracted
- Neck: jugular venous pressure of 4 cm H_2O above the sternal angle
- Pulmonary: clear lung fields bilaterally
- Cardiac: tachycardia; S_1 and S_2, no murmurs, rubs, or gallops

Diagnostic Studies

Plasma	
Brain natriuretic peptide (BNP)	100 pg/mL (N<167)
D-dimer	25 µg/mL (N<0.5)

Blood	
Hematocrit	39%
Hemoglobin	13.0 g/dL
WBC	9000/mm^3
Platelet count	325,000/mm^3

- chest x-ray: no abnormalities

Question: Which of the following is the most likely diagnosis?

(A) COPD exacerbation
(B) Heart failure
(C) Pneumonia
(D) Pneumothorax
(E) Pulmonary embolism

56. A 20-year-old primigravid woman comes to the emergency department at 8 weeks' gestation because of a 2-day history of persistent nausea and vomiting with a 0.9-kg (2-lb) weight loss during this time. She also notes that she has not voided during the past 8 hours. She has not had fever, chills, or contact with anyone known to be ill. Medical history is unremarkable. She has taken prenatal vitamins sporadically during her pregnancy, but they worsen her nausea and vomiting. Temperature is 36.8°C (98.2°F), pulse is 98/min, respirations are 18/min, and blood pressure is 118/60 mm Hg. Oxygen saturation is 99% on room air. Physical examination discloses no abnormalities. Urine specific gravity is greater than 1.030 (N=1.003–1.029). Which of the following laboratory study results is most likely to be abnormal in this patient?

(A) Leukocyte count
(B) Serum ALT and AST activities
(C) Serum calcium concentration
(D) Serum glucose concentration
(E) Serum urea nitrogen concentration
(F) No abnormality is likely

57. A 60-year-old woman, who lives alone, is brought to the emergency department on a Friday evening by her next-door neighbor who says the patient has fallen several times during the past several days. An emergency department record from a visit 1 year ago for a minor laceration indicates that the patient has Parkinson disease with mild dementia, bipolar disorder, and hypertension. Her brother fills her weekly medication organizer, but he is overseas on a business trip for the week. The patient is unable to remember which medications she takes and whether there have been any changes recently to her medication regimen. The neighbor was unable to locate her pill organizer in her home, and the patient thinks her brother keeps her pill bottles at his house to prevent her from inadvertently taking extra doses. She has her brother's address on a card in her wallet, along with the names and phone numbers of her primary care physician, psychiatrist, and pharmacy. The medical record from her visit 1 year ago shows that her medications included clonazepam, clonidine, and lithium carbonate. Doses of each medication are also listed. Attempts to contact the patient's primary care physician and psychiatrist for more detailed medication history have been unsuccessful. Which of the following is the most appropriate initial approach to managing the patient's medications?

(A) Ask the police to enter the brother's house to locate the patient's pill bottles
(B) Contact the patient's pharmacy to obtain information on her current medications
(C) Observe the patient closely while continuing to try to reach her psychiatrist
(D) Order the medications as listed in the emergency department records from her previous visit
(E) Order serum concentrations of the medications listed on the previous emergency department record, then prescribe doses of the medications in accordance with those concentrations

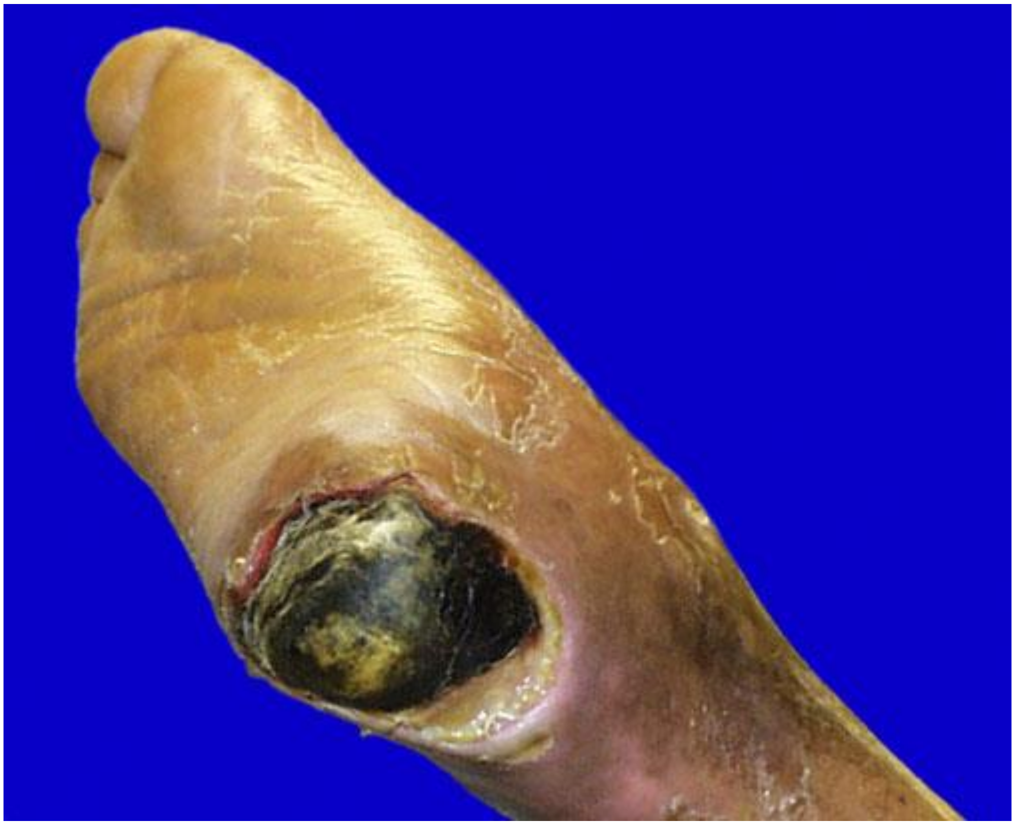

58. A 73-year-old man is admitted to a nursing care facility from the hospital following a prolonged hospitalization for pneumonia, septicemia, and respiratory failure. Medical history is remarkable for advanced dementia, Alzheimer type, hypertension, and type 2 diabetes mellitus. Current medications include lisinopril, enteric-coated aspirin, and insulin glargine. The patient appears frail and is confined to bed. He opens his eyes to voice but is noncommunicative. BMI is 27 kg/m^2. Temperature is 37.2°C (99.0°F), pulse is 66/min, respirations are 15/min, and blood pressure is 142/86 mm Hg. Oxygen saturation is 97% on room air. Physical examination discloses the finding on the patient's right foot as shown in the photograph. The remainder of the examination discloses no abnormalities. Which of the following is the most likely underlying cause of this patient's condition?

(A) Infection with methicillin-resistant *Staphylococcus aureus*
(B) Prolonged immobility
(C) Pyoderma gangrenosum
(D) Systemic atheroemboli
(E) Venous stasis secondary to valvular incompetence

59. A 78-year-old woman, who was admitted to the hospital 3 days ago because of worsening renal failure, has become increasingly lethargic and nonresponsive. Medical history is significant for type 2 diabetes mellitus, hypertension, and stroke 1 year ago with residual right-sided weakness and slurred speech. Medications include insulin, aspirin, and furosemide. On admission, the patient was alert with mildly slurred speech; she was oriented to person, place, and time. Physical examination disclosed decreased motor strength and muscle atrophy in the right upper and lower extremities. Results of laboratory studies showed a serum urea nitrogen concentration of 82 mg/dL and a creatinine concentration of 6.1 mg/dL. Hemodialysis was recommended but the patient declined and expressed an understanding that she would die without the intervention. In addition, she requested a do-not-attempt resuscitation (DNAR) order. This was documented in her medical record and her code status was changed to DNR. Serum urea nitrogen concentration now is 130 mg/dL. She has had no urine output during the past 48 hours. The patient's daughter arrives and states that she is named as the surrogate decision maker by her mother's advance directive. The daughter insists that hemodialysis be initiated and the code status be changed to full code. Which of the following is the most appropriate course of action?

(A) Continue current management; do not initiate hemodialysis
(B) Initiate hemodialysis and change the code status to full code until the patient is more alert
(C) Initiate hemodialysis but keep the code status as DNAR until the patient is more alert
(D) Obtain consent from the patient's daughter to initiate all medically indicated interventions

60. An 88-year-old woman is transferred to the emergency department from the nursing care facility where she resides 4 hours after facility staff noted that the patient had rectal bleeding. Medical history is significant for long-standing dementia secondary to multiple prior strokes, coronary artery disease, hypertension, hypercholesterolemia, and chronic renal disease. Medications include 81-mg aspirin, simvastatin, lisinopril, and hydrochlorothiazide. On arrival in the emergency department, temperature is 37.2°C (98.9°F), pulse is 102/min, respirations are 20/min, and blood pressure is 105/60 mm Hg. Oxygen saturation is 95% on room air. The patient is awake but is not oriented to person, place, or time. She is unable to speak coherently and becomes agitated and combative when a physical examination is attempted. Which of the following is the most appropriate initial intervention?

(A) Administer lorazepam and proceed with physical examination
(B) Contact the patient's health care proxy to discuss how to proceed
(C) Place the patient in soft wrist restraints and proceed with physical examination
(D) Schedule outpatient flexible sigmoidoscopy and discharge the patient

61. A 56-year-old man comes to the office because of a 3-week history of right arm weakness and drooping of his right eyelid. Medical history is significant for hypertension and dyslipidemia, but he takes no medications. He has smoked one-half pack of cigarettes daily for the past 40 years. BMI is 26 kg/m^2. Temperature is 37.2°C (99.0°F), pulse is 82/min, respirations are 20/min, and blood pressure is 138/74 mm Hg. Physical examination shows ptosis on the right side. The left pupil measures 5 mm in diameter and the right pupil measures 4 mm in diameter; both pupils are reactive to light and accommodation. Muscle strength is 3/5 in the right finger flexors and hand grip. Muscle strength and deep tendon reflexes are otherwise normal. Fingerstick blood glucose concentration is 119 mg/dL. Which of the following is the most likely diagnosis?

(A) Cerebellar artery aneurysm
(B) Diabetic oculomotor paresis
(C) Myasthenia gravis
(D) Myasthenic (Lambert-Eaton) syndrome
(E) Pancoast tumor

Items #62–63 are part of a sequential item set. In the actual examination environment, you will **not** be able to view the second item until you click "Proceed to Next Item." After navigating to the second item, you will not be able to add or change an answer to the first item.

A 16-year-old boy comes to the office because of a 2-week history of six to eight daily episodes of loose, watery stools. The episodes are associated with abdominal cramps. His stool has not contained any blood. Medical history is significant for recurrent sinopulmonary infections typically treated with oral antibiotics. His last infection occurred 1 month ago and resolved within 10 days. He currently takes no medications and is not sexually active. He has no history of recent travel. He does not smoke cigarettes, drink alcoholic beverages, or use other substances. BMI is 18 kg/m2. Temperature is 37.7°C (99.9°F), pulse is 100/min, respirations are 18/min, and blood pressure is 110/60 mm Hg. Lungs are clear to auscultation. Cardiac examination discloses no abnormalities. Bowel sounds are normoactive. Abdomen is soft; palpation discloses mild, diffuse tenderness but no masses or hepatosplenomegaly. Digital rectal examination discloses brown stool; test of the stool for occult blood is negative. A culture of the stool is obtained. Acid-fast smear of a stool specimen shows numerous 6-µm ovoid oocysts.

62. Which of the following is the most likely causal agent of this patient's condition?

(A) *Cryptosporidium parvum*
(B) *Entamoeba histolytica*
(C) Norovirus
(D) *Salmonella typhi*
(E) *Shigella flexneri*

63. Appropriate treatment is prescribed for the patient's gastrointestinal infection. It is noted that this is the patient's sixth illness during the past 18 months. In determining whether this patient has an immune deficiency, which of the following is the most appropriate diagnostic study to obtain at this time?

(A) CD4+ T-lymphocyte count
(B) CD8+ T-lymphocyte count
(C) Serum complement concentrations
(D) Serum interferon gamma assay
(E) Serum quantitative immunoglobulin concentrations

END OF SET

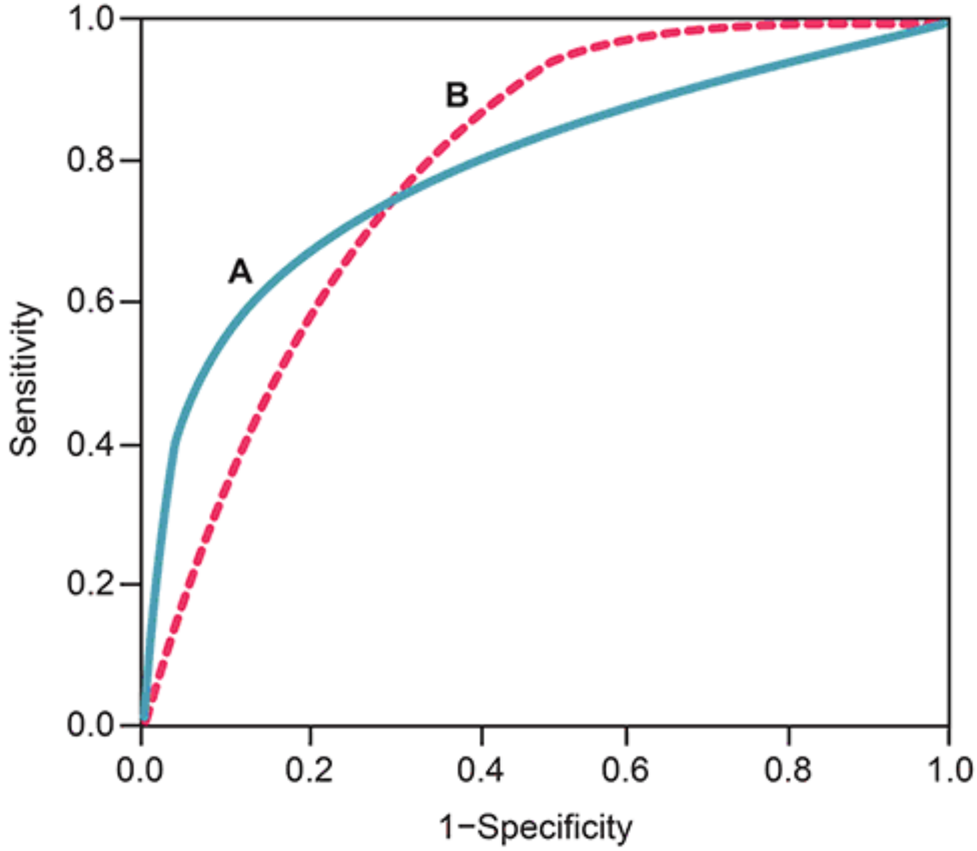

64. Physicians at an ambulatory clinic would like to develop a rapid screening test to diagnose influenza virus infection after the clinic reported 20% more cases of influenza virus infection this year than any other year. Patients at the clinic currently wait almost 90 minutes for results of a nasopharyngeal swab test to determine if they have influenza virus infection. The physicians develop two tests, Test A and Test B, which take only 10 minutes to yield results. The receiver operator characteristic (ROC) curves for the two tests are shown. Based on these data, which of the following is the most appropriate conclusion to draw about Test A compared with Test B?

(A) In the high false positive rate range, Test A is more accurate than Test B
(B) In the high sensitivity range, Test A is more accurate than Test B
(C) In the low false positive rate range, Test A is more accurate than Test B
(D) In the low sensitivity range, Test B is more accurate than Test A

65. A 5-year-old boy is brought to the office by his parents because of a 2-month history of progressive difficulty climbing stairs, playing on playground equipment, and rising from a chair. He has mild cognitive delay for which he is receiving academic assistance, and he has had delayed milestones such as walking. He otherwise is healthy. He has no siblings. Family history is unremarkable; neither of his parents has any siblings. The patient's vital signs are within normal limits. Physical examination shows hip girdle proximal muscle weakness and increased circumference of the calves bilaterally. The patient has to use his arms to stand up. Serum creatine kinase activity is 25,000 U/L (N=20–200). Genetic testing is ordered. Which of the following mutations is most likely to be identified in this patient?

(A) Frameshift insertion in the gene encoding alpha-1 (III) collagen
(B) Large deletion in the gene encoding dystrophin
(C) Missense mutation in the gene encoding fibrillin 1
(D) Splice-site mutation in the gene encoding spectrin
(E) Translocation involving the gene encoding myosin I

66. Researchers are attempting to recruit patients from the primary care clinic of a large urban hospital to take part in a minimal-risk research study. When initiating the informed consent process, it is observed that African American patients tend to exhibit a greater reluctance to take part in the study. During interim review, data confirm low enrollment totals among African Americans in the community. Which of the following is the best course of action to take?

(A) Ask the institutional review board to waive the requirement for informed consent because it is deterring potential participants
(B) Convene a representative focus group of potential participants to ask questions regarding reluctance to enroll
(C) Focus recruitment efforts on White patients who are more likely to enroll and acknowledge this study limitation in the publication
(D) Recognize this as an unavoidable impediment and continue the recruitment practices in the same manner
(E) Require all patients to take part in the study if they wish to receive care in this clinic to ensure a representative sample

67. A 54-year-old woman comes to the office because of a 10-month history of moderate to severe upper abdominal pain, nausea after eating, and a "burning" sensation in her throat and chest. This is her third visit for these symptoms during the past 3 months. She says that the recommended diet modification and weight loss regimen have not relieved her symptoms, but she later acknowledges that her diet changes have been minimal and she has not been exercising regularly. She takes over-the-counter omeprazole daily but says she is unsure how many tablets she takes. She says her symptoms have caused her to use all of her sick days at work and she is "miserable." She says she is convinced that she has Barrett esophagus. BMI is 29 kg/m^2. Vital signs are within normal limits. She appears anxious and preoccupied with her symptoms. Physical examination shows no abnormalities. On mental status examination, she is irritable but is otherwise communicative and fully oriented. Cognition is intact. She uses medical terms with ease. An upper gastrointestinal series 1 month ago showed no abnormalities. The patient requests an endoscopy because she read about them while researching her symptoms on the Internet. Which of the following is the most appropriate next step in management?

(A) Ask the patient if she would like to see a specialist
(B) Ask the patient to describe her goals for and expectations of treatment
(C) Ask the patient to further explain why she thinks she has Barrett esophagus
(D) Discuss why the patient has not made the suggested changes to her diet and exercise
(E) Encourage the patient to describe how she feels about having an unresolved medical condition

68. A 64-year-old woman comes to the office because of a 5-month history of pain in her right calf on exertion. The pain occurs when she walks and resolves within 5 minutes of rest. Initially, the pain developed after the patient walked three blocks, but now it occurs after she walks only one block. She has no other symptoms. Medical history is remarkable for hypertension, hyperlipidemia, and type 2 diabetes mellitus. Medications are metformin, lisinopril, and atorvastatin. She does not smoke cigarettes, drink alcoholic beverages, or use any other substances. BMI is 31 kg/m^2. Temperature is 37.0°C (98.6°F), pulse is 80/min, respirations are 16/min, and blood pressure is 120/80 mm Hg. Oxygen saturation is 95% on room air. Ankle brachial index is 0.6 on the right and 0.8 on the left. Physical examination discloses a left carotid bruit, a right femoral bruit, and decreased pedal pulses bilaterally. Laboratory studies show a hemoglobin A_{1c} of 7.0%. The results of additional laboratory studies, including urinalysis, are within the reference ranges. The most appropriate pharmacotherapy for this patient targets which of the following?

(A) Cyclooxygenase
(B) Factor Xa
(C) Nitric oxide synthase
(D) Phosphodiesterase
(E) Prostacyclin synthase

The pharmaceutical advertisement on the following pages is for use with items #69 and 70 on page 56.

WHEN CONSISTENT FOCUS AND IMPULSE CONTROL ARE NEEDED

Essepro XL tabs

(focophenydate sodium) 10 mg, 25 mg

Better sustained action than other medications in its class

Improved late afternoon performance aids in homework

No adverse impact on sleep

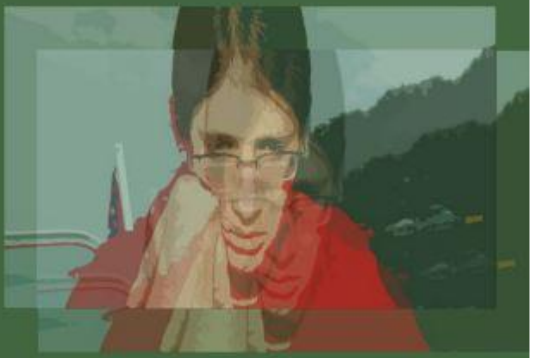

In the Featured Outcomes of Concentration in US children (FOCUS) study, Essepro XL 10 mg and 25 mg were compared with methylphenidate XL and placebo as monotherapy for attention deficit-hyperactivity disorder (ADHD) in children ages 6–17 years in a 12-week, double-blind trial. Outcomes for at least 50 patients in each of the four treatment arms were collected. The criteria for enrollment were based on DSM-V criteria for ADHD and, in some cases, physician assessment outside of DSM criteria. The FOCUS study used the NBMEC test of concentration. Previous studies have demonstrated that a score of 3.5 or greater is consistent with current attention to task. Both Essepro XL 10 mg and 25 mg demonstrated sustained duration scores of >3.5 on the NBMEC test of concentration.[1,2]

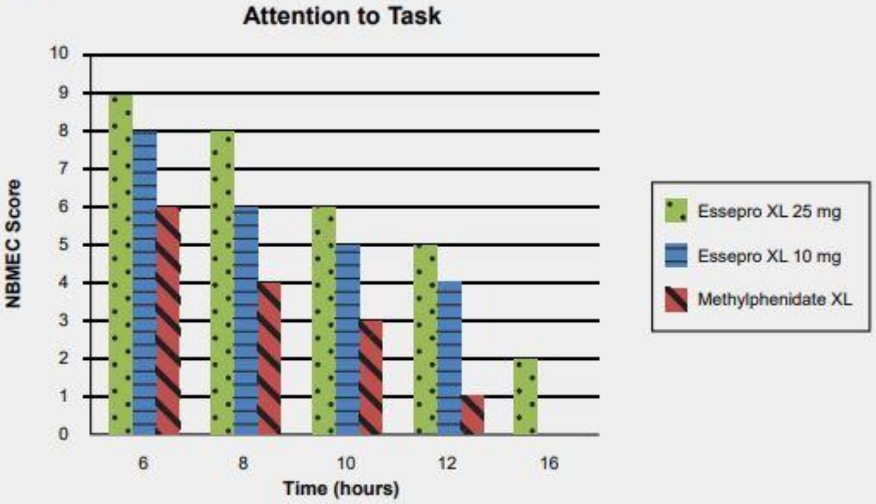

WARNING: Essepro XL (focophenydate sodium) is a schedule II controlled substance that can lead to abuse or dependency.

Essepro XL is approved for children ages 6 to 12 years and adolescents ages 13 to 17 years as once daily monotherapy for the treatment of ADHD.

The safety and efficacy of Essepro XL in adults with ADHD have not been established.

Most Commonly Reported Adverse Events in the FOCUS Trial (a 12-week, active-comparator, placebo-controlled trial[3])

Adverse Events	Essepro XL 25 mg (n=55)	Essepro XL 10 mg (n=50)	Methylphenidate XL (n=52)	Placebo (n=57)
Decreased appetite	11	7	6	7
Insomnia	8	7	6	5
Irritability	8	6	7	11
Tachycardia	7	5	3	1
Headache	7	4	6	2
Emotional lability	7	3	2	0
Dizziness	6	7	8	4
Weight loss >2 kg	5	3	1	0
Nausea	4	3	8	4
Abdominal pain	3	3	4	4
Vomiting	1	1	2	3
New onset tic	1	1	0	0

Important Safety Information

Essepro XL should not be used in patients with known hypersensitivity to stimulant medications, such as methylphenidate.

Use with caution in patients with a history of hypertension or who, on two separate occasions, have had systolic blood pressure measurements greater than the 95th percentile for age and height.

Essepro XL should not be used in patients with a history of hyperthyroidism, symptomatic cardiovascular disease, glaucoma, psychotic symptoms, drug abuse, or monoamine oxidase inhibitor (MAOI) or selective-serotonin reuptake inhibitor (SSRI) use.

Psychosis, mania, growth suppression, visual disturbances, and aggression have been reported in patients taking stimulant medications.

Dosing

Essepro XL is available in 10 mg and 25 mg tablets. It is recommended that Essepro XL be taken in the morning with food.

Tablets should be swallowed whole and not divided or crushed.

1. Jones T, Barone M. Prevalidation of attention to task scale in children and adolescents. *USMLE J Psychometric Measures*. 2011;34:112-117. 2. Smith TA, Mathes B. Kinetics and efficacy of once daily focophenydate sodium for children and adolescents with attention deficit-hyperactivity disorder. *USMLE J Attention Distraction*. 2012;9:35-42. 3. Ellis M, Carr S. Safety of focophenydate sodium in children and adolescents with attention deficit-hyperactivity disorder: results of the 12-week FOCUS study. *USMLE J Ped Psych*. 2013;44:18-22.

Essepro XL tabs
(focophenydate sodium) 10 mg, 25 mg

NB Pharma, Inc.

Items #69–70 refer to the pharmaceutical advertisement displayed on the previous pages.

An 18-year-old woman comes to the university health center requesting medication to improve her concentration. She says she needs to maintain a high grade-point average so that she will be accepted into graduate school. She graduated in the top 10% of her class at a selective preparatory high school. She is taking six classes this semester and says that she is having trouble keeping up with her workload. Several of her friends take medication for attention-deficit/hyperactivity disorder (ADHD) and have told her that these medications help them study and perform better on examinations. The patient says she took some of her roommate's Essepro XL and was able to study for hours without being distracted. She says Essepro XL has the added benefit of suppressing her appetite, which might help her lose weight. Medical history is remarkable for asthma and seasonal allergies since early childhood. Medications are albuterol inhaler as needed, fluticasone inhaler twice daily, and oral 24-hour loratadine 10 mg with pseudoephedrine once daily. BMI is 30 kg/m^2. Blood pressure is 130/84 mm Hg; other vital signs are within normal limits. The lungs are clear to auscultation. Cardiac examination discloses a midsystolic click. The patient has an advertisement for Essepro XL, as shown, and asks the physician if she could be prescribed this medication to improve her concentration and to lose weight. The physician decides not to prescribe Essepro XL for this patient.

69. Based on the advertisement, which of the following is the most appropriate rationale for this decision?

(A) Essepro XL is contraindicated in patients who also are taking stimulant medications
(B) Essepro XL is likely to exacerbate the patient's heart condition
(C) Essepro XL is not indicated for this patient
(D) The patient is likely to share Essepro XL with her friends
(E) There is no evidence that Essepro XL will help the patient lose weight

70. Based on the advertisement, which of the following is the greatest advantage of Essepro XL over methylphenidate XL?

(A) Decreased likelihood of disrupted sleep patterns
(B) Decreased potential for dependency
(C) Decreased rates of nausea and anorexia
(D) Increased academic performance
(E) Increased availability of safety data
(F) Increased duration of action

END OF SET

---

71. A 29-year-old woman comes to the office for counseling. She learned that she is HIV-positive 1 month ago and she believes that she most likely contracted the infection approximately 6 months ago. Laboratory studies ordered in preparation for this visit show a CD4+ T-lymphocyte count of 800/mm^3 and undetectable serum HIV RNA levels. She has no symptoms of AIDS. She has never been pregnant, but she is considering pregnancy and wants to know her risk for transmitting HIV to future children. Which of the following is the most accurate statement?

(A) As a subgroup, newborns have the fastest rate of increase in HIV infection
(B) Intrapartum transmission accounts for less than 50% of neonatal HIV infection
(C) Maternal viral load is the best predictor of perinatal transmission of HIV infection
(D) The risk for perinatal transmission is higher among women who conceive within 1 year of initial HIV infection
(E) Route of delivery does not influence the risk for perinatal transmission of HIV infection

72. A 50-year-old woman who has been your patient for the past 3 years comes to the office because of heartburn and increased belching and bloating after meals for the past month. Antacids provide little relief. The symptoms tend to occur after eating. She is currently asymptomatic. She has never had an operation. The patient identifies as African American. Physical examination shows mild scleral icterus and a 4-cm mass in the epigastric area. Which of the following is the most appropriate initial diagnostic study?

(A) CT scan of the abdomen
(B) Determination of serum amylase and lipase concentrations
(C) Endoscopic retrograde cholangiopancreatography
(D) HIDA scan
(E) Upper gastrointestinal barium study

73. A 50-year-old woman comes to the office because of the gradual onset of pain and swelling of both knees and distal interphalangeal joints of the hands during the past 12 weeks. The pain is exacerbated with movement and is accompanied by morning stiffness that usually resolves within 15 to 20 minutes. Recently the joint pain has occurred at night and has restricted her activity. Ibuprofen has provided moderate relief of her symptoms. Medical history is remarkable for type 2 diabetes mellitus, hypertension, and obesity. Additional medications include glipizide, metformin, and hydrochlorothiazide. She is 168 cm (5 ft 6 in) tall and weighs 105 kg (230 lb); BMI is 37 kg/m^2. Temperature is 37.0°C (98.6°F), pulse is 70/min, respirations are 16/min, and blood pressure is 140/90 mm Hg. Examination of the hands shows bony enlargements of the distal and proximal interphalangeal joints with mild tenderness to the interphalangeal joints. Examination of both knees shows bony enlargement with tenderness at the joint margin, limited range of motion, and mild joint effusions. Results of laboratory studies are shown:

Serum		Blood	
Rheumatoid factor	Nonreactive	WBC	8000/mm^3
Uric acid	6 mg/dL		

X-rays of the knees are most likely to show which of the following?

(A) Bone demineralization and erosions
(B) Soft tissue swelling, sclerotic joint margins, and large cystic erosions
(C) Soft tissue swelling with no evidence of cartilage or bone destruction
(D) Subchondral sclerosis of bone and asymmetric joint space narrowing
(E) No abnormal findings

74. A 17-year-old high school football player is brought to the emergency department by his parents 1 hour after sustaining an injury to his upper back during a game. He says another player struck him in the back with his helmet. Immediately following the incident, the patient noticed weakness of his lower extremities. On arrival, temperature is 36.7°C (98.0°F), pulse is 110/min, respirations are 20/min, and blood pressure is 130/80 mm Hg. Physical examination discloses point tenderness at the midthoracic spine. Muscle strength is 3/5 in the lower extremities. Sensation is diminished to pain and vibration below the lower chest margins. Deep tendon reflexes are absent in the lower extremities. Babinski sign is absent bilaterally. MRI of the spine is most likely to show which of the following?

(A) Anterior spinal artery territory ischemia
(B) Contusion of the thoracic spinal cord
(C) Lateral T7 disc herniation with nerve root compression
(D) Thoracic cord hemisection
(E) Traumatic syrinx

75. **Patient Information**
Age: 19 years
Gender: M, self-identified
Race/Ethnicity: unspecified
Site of Care: hospital

The patient who is a college student is admitted because of fever, chills, intense headache, nonproductive cough, and generalized weakness for the past 3 days. Medical history is unremarkable and he takes no medications. He does not use any substances. He works as a greenskeeper at a local golf course in western North Carolina. On admission, he appears acutely ill. He is somnolent but arousable. Temperature is 39.0°C (102.2°F), pulse is 106/min, respirations are 22/min, and blood pressure is 90/56 mm Hg. Oxygen saturation is 98% on room air. Examination of the skin shows an erythematous, macular rash with superimposed petechiae on the palms, soles, legs, and thighs. Funduscopic examination discloses no abnormalities. Neck is supple with no adenopathy. The remainder of the physical examination, including neurologic examination, discloses no abnormalities. Intravenous access is established. Lumbar puncture for examination of cerebrospinal fluid discloses an opening pressure of 210 mm H_2O; the cerebrospinal fluid is slightly turbid. ECG shows nonspecific ST-segment and T-wave abnormalities. Portable chest x-ray shows no abnormalities. Results of laboratory studies are shown:

Serum		Blood	
Urea nitrogen	23 mg/dL	Hemoglobin	11.5 g/dL
Creatinine	1.3 mg/dL	WBC	11,600/mm^3
Glucose	99 mg/dL	Neutrophils, segmented	75%
Na^+	134 mEq/L	Neutrophils, bands	15%
K^+	4.5 mEq/L	Lymphocytes	10%
Cl^-	104 mEq/L	Platelet count	43,000/mm^3
HCO_3^-	22 mEq/L	INR	1.3
Cerebrospinal fluid			
Cell count	20 cells/mm^3		
Glucose	70 mg/dL		
Lymphocytes	95%		
Proteins	75 mg/dL		
Gram stain	Negative		
India ink preparation	Negative		

Appropriate therapy is initiated and the patient's condition improves during the next week. Cultures of the blood and cerebrospinal fluid are negative. Which of the following is the most likely source of this patient's illness?

(A) Inhaling contaminated droplets
(B) Insecticide from the golf course
(C) Kissing
(D) Mosquito bite
(E) Tick bite

76. **Patient Information**
Age: 30 years
Gender: F, self-identified
Race/Ethnicity: unspecified
Site of Care: emergency department (ED)

The patient is brought by police because of a 2-hour history of worsening abdominal pain, diarrhea, and joint pain. The symptoms began while the patient was incarcerated in the local jail, where she was placed 12 hours ago after she was arrested for shoplifting. Her medical history is unknown. On arrival at the ED, the patient is irritable and sleepy; she yawns frequently. She is diaphoretic. BMI is 18 kg/m^2. Temperature is 37.1°C (98.7°F), pulse is 110/min, respirations are 30/min, and blood pressure is 145/92 mm Hg. Oxygen saturation is 94% on room air. Pupils are moderately dilated. Bowel sounds are hyperactive. The remainder of the physical examination discloses no abnormalities. Results of urine toxicology screening in this patient will most likely be positive for which of the following?

(A) Cannabinoids
(B) Cocaine
(C) Methamphetamine
(D) Opioids

77. A 39-year-old woman comes to the office because of a 6-week history of malaise, fatigue, and a 4.5-kg (10-lb) weight loss. She has not had difficulty sleeping or depressed mood. Medical history is unremarkable and she takes no medications. She does not smoke cigarettes or drink alcoholic beverages. BMI is 28 kg/m^2. Temperature is 37.2°C (99.0°F), pulse is 110/min, respirations are 12/min, and blood pressure is 140/90 mm Hg. Physical examination discloses a fine tremor. Which of the following additional signs or symptoms would be most consistent with the underlying diagnosis in this patient?

(A) Constipation
(B) Delayed relaxation reflex
(C) Heat intolerance
(D) Tenting of the skin

NOTE: THIS IS THE END OF BLOCK 2.
ANY REMAINING TIME MAY BE USED TO CHECK ITEMS IN THIS BLOCK.

Block 3: ACM
Items 78–107; Time - 45 minutes

ALL ITEMS REQUIRE SELECTION OF ONE BEST CHOICE.

78. **Patient Information**
Age: 25 years
Gender: F, self-identified
Race/Ethnicity: White, self-identified
Site of Care: office

The patient who works as an attorney at a large law firm presents because of a 1-week history of pain in and around her left upper jaw. She is unable to open her mouth wide, and when she attempts to do so she feels a snapping sensation in the joint in front of her ear at the jawbone. She has not sustained any recent trauma. She was recently diagnosed with major depressive disorder, for which she has been taking paroxetine. She remembers being advised in the past by her dentist to consider braces for her teeth, but she never wore them because they were unaffordable. Medical history is otherwise unremarkable, and she takes no other medications. Temperature is 37.0°C (98.6°F), pulse is 80/min, respirations are 20/min, and blood pressure is 104/60 mm Hg. Physical examination discloses point tenderness at the left temporomandibular joint. Examination of the ears shows good light reflex bilaterally on the tympanic membranes. After the patient removes chewing gum from her mouth, examination of the oral cavity shows pink buccal mucosa and no evidence of dental abscess or trauma. The patient should be advised to do which of the following?

(A) Avoid chewing gum and eating hard or chewy foods
(B) Consult an orthodontist
(C) Cradle the phone receiver between shoulder and jaw
(D) Open and close her jaw widely three time daily
(E) Take oxycodone as needed for pain

79. A 68-year-old woman was admitted to the intensive care unit 36 hours ago after becoming obtunded due to respiratory distress. She is now intubated and chest x-ray is consistent with congestive heart failure and pneumonia. She has a long history of morbid obesity, chronic bronchitis, schizoaffective disorder, and cigarette smoking. She is reported to have been relatively stable on haloperidol, benztropine, and lithium carbonate. Her present medication regimen includes acetaminophen, methylprednisolone, ciprofloxacin, haloperidol, benztropine, and lithium carbonate. She also requires intravenous lorazepam every 2 hours for agitation that caused her to fight the ventilator. Vital signs now show a spike in temperature to 38.9°C (102.0°F). Physical examination discloses lead pipe rigidity. Serum creatine kinase concentration is 846 U/L and serum lithium concentration is 0.86 mEq/L (therapeutic=0.5–1.5). Arterial blood gas analysis while breathing 40% oxygen is shown:

P_{O_2}	68 mm Hg
P_{CO_2}	36 mm Hg
pH	7.46
O_2 saturation	91%

At this time it is most important to discontinue which of the following medications?

(A) Benztropine
(B) Ciprofloxacin
(C) Haloperidol
(D) Lorazepam
(E) Methylprednisolone

80. A 35-year-old woman comes to the office because she noticed a lump in her neck 2 months ago. She has not had pain, difficulty swallowing, or change in her voice. She is a native of Germany and immigrated to the United States 10 years ago. Medical history is significant for Hodgkin lymphoma at age 12 years treated with mantle radiation. Hypothyroidism was diagnosed at age 25 years. Her only routine medication is levothyroxine. The patient has smoked one pack of cigarettes daily for the past 10 years. She appears well. BMI is 26 kg/m^2. Temperature is 36.6°C (97.8°F), pulse is 82/min, respirations are 18/min, and blood pressure is 108/66 mm Hg. Physical examination discloses a 2.5-cm (1-in) nodule in the right lobe of the thyroid gland. There is also a 1-cm (0.4-in) nodule in the left thyroid lobe. Results of serum thyroid function tests show a thyroid-stimulating hormone concentration of 0.6 µU/mL and a free thyroxine concentration of 1.7 ng/dL (N=0.7–1.8). Which of the following factors in this patient is most indicative of a poor prognosis?

(A) Cigarette smoking
(B) Hyperthyroidism
(C) Iodine deficiency
(D) Presence of multiple nodules
(E) Radiation exposure

81. A 38-year-old man with type 1 diabetes mellitus and hemochromatosis comes to the office for a routine health maintenance examination. Medical history is otherwise unremarkable. His conditions have been well managed with therapeutic phlebotomy and insulin therapy. He currently feels well. BMI is 24 kg/m^2. Temperature is 36.7°C (98.1°F), pulse is 88/min, respirations are 16/min, and blood pressure is 130/84 mm Hg. The patient's skin is not discolored and sclerae are white. Physical examination discloses no abnormalities. Results of laboratory studies are shown:

Serum		Blood	
ALT	72 U/L	Hematocrit	30%
AST	64 U/L	Hemoglobin	9.8 g/dL
Bilirubin, total	0.7 mg/dL	Hemoglobin A_{1c}	6.6%
Glucose, fasting	134 mg/dL		

This patient should be counseled that he is at increased risk for mortality if he consumes which of the following foods?

(A) Bean sprouts
(B) Berries
(C) Raw oysters
(D) Undercooked eggs

82. A 24-year-old woman with asthma comes to the office because of a 2-week history of mild shortness of breath and a nonproductive cough that occurs occasionally during the day and frequently at night. During this time, she has required use of her albuterol inhaler twice daily. Medical history is otherwise unremarkable and her only other medication is an oral contraceptive. The patient drinks three to four alcoholic beverages weekly and does not smoke cigarettes. She appears alert and comfortable. BMI is 25 kg/m^2. Temperature is 37.2°C (99.0°F), pulse is 88/min, respirations are 16/min, and blood pressure is 114/72 mm Hg. Oxygen saturation is 97% on room air. FEV_1 is 70% of predicted. Physical examination discloses no abnormalities. Which of the following is the most appropriate next step in pharmacotherapy?

(A) Add inhaled fluticasone
(B) Add inhaled tiotropium
(C) Add oral azithromycin
(D) Add oral montelukast
(E) Increase the dosage of the inhaled albuterol to four times daily
(F) Prescribe a 5-day course of oral prednisone

83. A 36-year-old woman is admitted to the hospital because of a 1-day history of sore throat and fever with temperatures to 40.0°C (104.0°F). She has not had cough, dyspnea, headache, neck stiffness, rash, diarrhea, or pain. Medical history is significant for Graves disease diagnosed by her primary care physician 3 weeks ago. At that time, the patient had a goiter and reported generalized weakness and hair loss. Therapy with propranolol and methimazole was initiated, and the patient's symptoms had been improving. BMI is 20 kg/m^2. Temperature is 39.9°C (103.8°F), pulse is 123/min, respirations are 20/min, and blood pressure is 108/68 mm Hg. Oxygen saturation is 98% on room air. Physical examination shows pharyngeal erythema without exudate and a diffuse and symmetric goiter. Lungs are clear to auscultation. Cardiac examination discloses a grade 2/6 systolic murmur at the left upper sternal border. There is no hepatosplenomegaly. Results of laboratory studies are shown:

Blood		Urine	
Hematocrit	35%	Protein	1+
Hemoglobin	11.4 g/dL	WBCs	Negative
WBC	3300/mm^3		
Neutrophils, segmented	2%		
Neutrophils, bands	1%		
Eosinophils	4%		
Basophils	1%		
Lymphocytes	78%		
Monocytes	14%		
MCV	82 μm^3		
Platelet count	221,000/mm^3		

Blood cultures are ordered and methimazole is discontinued. Which of the following is the most appropriate next step?

(A) Antinuclear antibody test
(B) Bone marrow aspiration and biopsy
(C) Cefepime therapy
(D) Filgrastim therapy
(E) HIV antibody test
(F) Propylthiouracil therapy

84. A 27-year-old woman, gravida 1, para 1, has a meeting with her physician and a lactation consultant in the hospital 1 day after uncomplicated spontaneous vaginal delivery of a 3572-g (7-lb 14-oz) female newborn at 38 weeks' gestation. Pregnancy was uncomplicated. The patient has good social support. Counseling is provided. The patient has begun to produce colostrum, and the newborn latches well. The patient says she is interested in breast-feeding and is convinced that breast milk will be best for her baby, but she is worried about how long she will be able to breast-feed because she plans to return to work as soon as possible. Her job involves packing boxes in an assembly line, and she often must work for multiple hours without a break. Her employer expects her back to work in 4 weeks. Which of the following is the most appropriate recommendation?

(A) Advise continuing breast-feeding now and discussing with her employer a schedule that will allow her to pump during work hours
(B) Encourage the patient to focus on breast-feeding and find a new job once her infant is ready to discontinue breast-feeding
(C) Recommend that the patient not breast-feed her infant
(D) Remind the patient that she needs to concern herself with what is best for her baby's care
(E) Suggest using both breast-feeding and bottle-feeding so the transition to work is easier for the baby

85. A 14-year-old boy is brought to the emergency department by a school official 1 hour after he was sent to the school nurse for behaving strangely in class and then becoming sleepy and difficult to arouse. School records indicate that the patient has no health problems and takes no medications. On arrival at the emergency department, the patient is lethargic but has no signs of trauma. Temperature is 37.3°C (99.1°F), pulse is 62/min, respirations are 10/min, and blood pressure is 106/62 mm Hg. Pulse oximetry on room air shows an oxygen saturation of 96%. Pupils measure 2 mm in diameter and are minimally reactive to light. Cardiopulmonary examination discloses clear lungs and normal cardiac rhythm. Bowel sounds are decreased. The patient moans and moves all extremities to painful stimuli. The remainder of the physical examination discloses no abnormalities. The most likely cause of this patient's condition is abuse of which of the following medications?

(A) Dextromethorphan
(B) Diazepam
(C) Diphenhydramine
(D) Methylphenidate
(E) Phenylephrine

86. **<u>Patient Information</u>**
Age: 70 years
Gender: M, self-identified
Race/Ethnicity: unspecified
Site of Care: office

The patient who has coronary artery disease presents for an initial visit to establish care. He had a myocardial infarction 2 years ago but is now able to walk 2 miles daily with no chest discomfort. Medical history is otherwise unremarkable. Medications include atorvastatin, metoprolol, and an 81-mg aspirin daily. He smoked two packs of cigarettes daily for 40 years but quit at the time of his myocardial infarction. He drinks one glass of red wine daily and has never used other substances. Temperature is 37.0°C (98.6°F), pulse is 82/min, respirations are 16/min, and blood pressure is 150/95 mm Hg. Physical examination discloses no abnormalities. Which of the following is the most appropriate additional pharmacotherapy for this patient?

(A) Amlodipine
(B) Clonidine
(C) Hydrochlorothiazide
(D) Lisinopril
(E) No additional pharmacotherapy is indicated

87. A 27-year-old woman, who underwent resection of a grade 2 astrocytoma in her right parietal lobe 6 weeks ago, is evaluated in the outpatient rehabilitation facility where she has been receiving ongoing care. Medical history otherwise is unremarkable. Her only medication is levetiracetam. The patient is right-hand dominant. She works as a graphic designer. BMI is 22 kg/m^2. Temperature is 37.0°C (98.6°F), pulse is 72/min, respirations are 18/min, and blood pressure is 110/60 mm Hg. Cranial nerves are intact. Muscle strength is 4/5 in the left upper and lower extremities and 5/5 in the right upper and lower extremities. There is mild spasticity at the left knee and ankle. Sensation to pinprick and proprioception are intact, but the patient has left extinction to double simultaneous stimulation, both tactile and visual. She is alert and oriented to person, place, and time. Her short-term memory is intact, and she can spell "world" backward. Speech is fluent. Gait shows mild circumduction; she uses a 4-point cane. Finger-nose testing discloses no ataxia. In considering this patient's ability to resume work, focused evaluation to determine which of the following is most likely to be helpful?

(A) Capacity to articulate ideas and thoughts
(B) Degree of control over her left hand when typing on a keyboard
(C) Effect of hemi-inattention on her visual spatial skills
(D) Overall ability to ambulate

88. A 9-year-old boy is brought to the office by his mother for evaluation of a 6-month history of abnormal movements that the mother describes as sudden, repetitive jerks often involving his shoulders and arms. She also has witnessed multiple episodes of repetitive eye blinking and throat clearing. There is no aura prior to the events. Initially, the episodes occurred approximately once weekly, but now they seem to occur daily and are interfering with his daily activities. His school performance has been acceptable, but his teacher says he is easily distracted and has difficulty sitting through class without fidgeting. Medical history is unremarkable, and he takes no medications. The patient is 133 cm (4 ft 4 in; 50th percentile) tall and weighs 29 kg (64 lb; 50th percentile). Vital signs are within normal limits. The patient is restless but cooperative. Muscle tone, deep tendon reflexes, and gait are normal; there is no tremor. The remainder of the neurologic examination discloses no focal abnormalities. During the examination, the patient experiences an episode of brief repetitive eye blinking; there is no change in consciousness during or after the episode. Based on these findings, this patient is most likely to develop which of the following?

(A) Hepatolenticular degeneration (Wilson disease)
(B) Huntington disease
(C) Intellectual disability
(D) Obsessive-compulsive disorder
(E) Seizure disorder

89. A 32-year-old woman is brought to the emergency department by a friend because of a 2-hour history of nausea, vomiting, sweating, and malaise. On arrival, the patient appears depressed. She says she took "several handfuls" of extra-strength acetaminophen and drank a pint of vodka 3 hours ago in an attempt to hurt herself. Medical history is significant for type 1 diabetes mellitus, major depressive disorder, and seizure disorder. Medications are insulin lispro, insulin glargine, fluoxetine, and phenytoin. She also takes three extra-strength acetaminophen tablets three to four times weekly for headaches or other muscle aches and pains. Vital signs are within normal limits. Palpation of the abdomen discloses mild right upper quadrant tenderness. The remainder of the examination discloses no abnormalities. Results of laboratory studies are shown:

Serum		Blood	
ALT	160 U/L	PT	12 seconds
AST	420 U/L	INR	1.0
Amylase	70 U/L		
Bilirubin, total	1.1 mg/dL		
Urea nitrogen	10 mg/dL		
Creatinine	1.0 mg/dL		
Na^+	132 mEq/L		
K^+	3.2 mEq/L		
Cl^-	108 mEq/L		
HCO_3^-	18 mEq/L		
Acetaminophen	250 µg/mL		
Alcohol	327 mg/dL		
HBcAb	Positive		
HBsAb	Positive		
HBsAg	Negative		
Hepatitis C antibody	Negative		

Which of the following factors in this patient's history most strongly indicates a poor prognosis?

(A) Chronic hepatitis B infection
(B) Concurrent alcohol consumption
(C) Fluoxetine therapy
(D) Type 1 diabetes mellitus

Items #90–91 are part of a sequential item set. In the actual examination environment, you will **not** be able to view the second item until you click "Proceed to Next Item." After navigating to the second item, you will not be able to add or change an answer to the first item.

An 11-year-old girl is brought to the emergency department by her parents because of a 1-day history of shortness of breath, fatigue, excessive thirst, and frequent urination. The parents say the onset of these symptoms coincided with an upper respiratory tract infection. Medical history is significant for type 1 diabetes mellitus. The patient's only medication is insulin. BMI is 23 kg/m^2 (93rd percentile). Temperature is 36.0°C (96.8°F), pulse is 110/min, respirations are 28/min, and blood pressure is 95/65 mm Hg. Oxygen saturation is 97% on room air. The patient appears moderately dehydrated and drowsy, but she is able to speak easily when prompted and communicates in a lucid fashion. Her respiratory pattern is rapid and deep, and she is using accessory muscles of respiration. Results of laboratory studies are shown:

Serum		Arterial blood gas analysis on room air	
Acetone	45 mg/dL (N<10)	Po_2	95 mm Hg
Glucose	693 mg/dL	Pco_2	26 mm Hg
		PH	7.16
		Base excess	−16 mEq/L

90. In addition to volume expansion, which of the following is the most appropriate intervention at this time?

(A) Bolus of intravenous sodium bicarbonate
(B) Continuous infusion of intravenous short-acting insulin
(C) Intermittent boluses of intravenous short-acting insulin
(D) Subcutaneous administration of half short-acting and half intermediate-acting insulin
(E) Subcutaneous administration of intermediate-acting insulin

91. An infusion of intravenous short-acting insulin is begun. Four hours later, laboratory studies are obtained, and results are shown:

Serum		Arterial blood gas analysis on room air	
Acetone	30 mg/dL (N<10)	pH	7.24
Glucose	385 mg/dL		

Shortly thereafter, the patient develops a headache and then suddenly loses consciousness. Physical examination shows increased muscle tone and extensor posturing of the extremities in response to painful stimuli. Which of the following is the most appropriate next step in management?

(A) Administration of sodium bicarbonate
(B) CT scan of the head
(C) EEG
(D) Fosphenytoin therapy
(E) Mannitol therapy

END OF SET

92. A 67-year-old woman with osteopenia comes to the office because of a 6-month history of increasingly severe, constant pain in her left hip and thigh. Before her symptoms began, she walked 1 mile daily. She now walks with a limp and must use a walker. Medical history also is remarkable for hypertension. Medications are hydrochlorothiazide, ibuprofen, and calcium and vitamin D supplementation. Her DEXA scan T-score was –1.8 1 year ago. Vital signs are within normal limits. Examination of the left lower extremity produces mild groin tenderness. Passive range of motion of the left hip is limited by pain. Serum studies show a calcium concentration of 8.6 mg/dL, intact parathyroid hormone concentration of 55 pg/mL (N=10–65), and 25-hydroxyvitamin D concentration of 30 ng/mL (N>20). X-rays of the left hip and femur show cortical thickening of the subtrochanteric region. MRI of the left hip and femur shows moderate edema of the bone marrow and soft tissue in the subtrochanteric region; there is cortical discontinuity laterally. Which of the following is the most appropriate next step in management?

(A) Bisphosphonate therapy
(B) Brace stabilization
(C) Calcitonin therapy
(D) Cast immobilization
(E) Intra-articular dexamethasone injection
(F) Surgical fixation
(G) Observation only

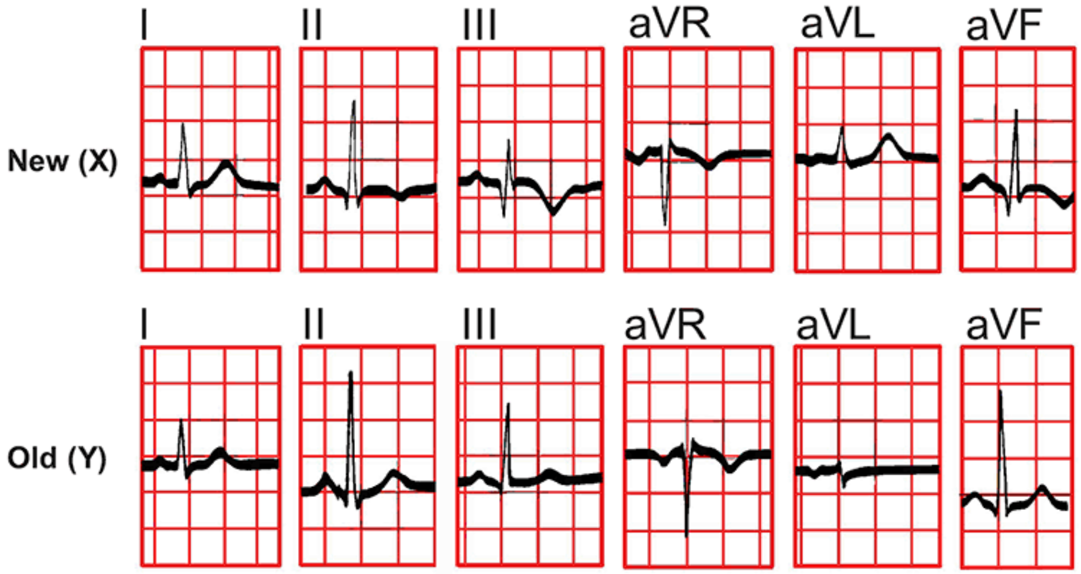

93. **Patient Information**
Age: 65 years
Gender: M, self-identified
Race/Ethnicity: unspecified
Site of Care: hospital

The patient is admitted because of a 4-week history of progressive shortness of breath on exertion and swelling of the lower extremities. The shortness of breath also awakens him at night. Medical history is remarkable for hypertension and type 2 diabetes mellitus. Medications are amlodipine, metformin, and daily aspirin. He does not smoke cigarettes, drink alcoholic beverages, or use other substances. Temperature is 37.0°C (98.6°F), pulse is 90/min and regular, respirations are 20/min, and blood pressure is 142/64 mm Hg. Oxygen saturation is 92% on room air. He appears mildly uncomfortable. Jugular venous pressure is 12 cm H_2O. Auscultation discloses inspiratory crackles to the mid lung fields bilaterally. Cardiac examination discloses an S_3 heard at the base. Abdominal examination shows no abnormalities. There is bilateral pitting edema to the knees. Results of laboratory studies are shown:

Serum		Blood	
Creatine kinase	35 U/L	Hematocrit	34%
Urea nitrogen	18 mg/dL	Hemoglobin	11.4 g/dL
Creatinine	1.3 mg/dL		
Na^+	133 mEq/L		
K^+	4.9 mEq/L		
Cl^-	102 mEq/L		
HCO_3^-	19 mEq/L		
Troponin I	<0.4		

ECG is shown. Administration of which of the following is contraindicated in this patient?

(A) Digoxin
(B) Furosemide
(C) Lisinopril
(D) Metoprolol
(E) Spironolactone

94. A 62-year-old man comes to the office for a routine follow-up examination 1 year after undergoing hemicolectomy for stage III colon cancer. Postoperative serum carcinoembryonic antigen concentration was 1 ng/mL (N≤3.0) at that time. The patient had completed a 6-month course of adjuvant chemotherapy. Today, he says he feels well and reports no symptoms. Medical history otherwise is unremarkable and he currently takes no medications. Vital signs are within normal limits. Physical examination shows only a well-healed midline surgical scar. Colonoscopy shows no lesions, polyps, or recurrence of cancer at the anastomosis site. Serum carcinoembryonic antigen concentration is 20 ng/mL. PET/CT scans of the chest, abdomen, and pelvis show a new 1-cm pulmonary nodule with irregular borders and increased uptake in the left lower lung lobe; the nodule does not involve the pleura or other major structures, and there is no pleural effusion. Which of the following is the most appropriate next step in management?

(A) Bronchoscopy
(B) Chemotherapy
(C) Discussion of palliative care
(D) Radiation therapy
(E) Resection of the nodule

95. A 64-year-old man is admitted to the hospital for evaluation of anemia and constant, nonradiating epigastric abdominal pain that began 3 days ago. He rates the pain as a 4 on a 10-point scale and says it partially improves after eating. He has not had nausea or vomiting. Medical history is remarkable for hypercholesterolemia and hypothyroidism. Medications are amlodipine and levothyroxine. He has no allergies. He drinks alcoholic beverages only occasionally and does not smoke cigarettes. Temperature is 36.7°C (98.0°F), pulse is 90/min, respirations are 14/min, and blood pressure is 110/70 mm Hg. Cardiopulmonary examination discloses no abnormalities. Abdominal examination discloses mild tenderness in the epigastrium with no guarding or rebound. Test of the stool for occult blood is positive. Hemoglobin concentration is 8.0 g/dL. Upper endoscopy shows a nonbleeding ulcer; biopsy of the ulcer is done, and results show a polymorphic infiltrate of small cells with reactive follicles that stain positive for B lymphocyte markers CD19 and CD20. In addition to prescribing omeprazole therapy, which of the following is the most appropriate initial treatment?

(A) Clarithromycin and amoxicillin therapy
(B) Cyclophosphamide and metronidazole therapy
(C) Radiation therapy
(D) Rituximab therapy
(E) Surgical resection

96. A 19-year-old man comes to the urgent care center because of severe lower abdominal and scrotal pain after he was elbowed in the groin 1 hour ago during a basketball game. The pain decreased 15 minutes after the incident and he continued to play in the game. Approximately 20 minutes later, the pain resumed with increased intensity, causing him to vomit. He has otherwise felt well. Medical history is unremarkable and he takes no medications. Family history is unremarkable. Temperature is 37.0°C (98.6°F), pulse is 100/min and regular, respirations are 14/min, and blood pressure is 150/88 mm Hg. The patient is writhing in pain. Bowel sounds are normoactive; the abdomen is soft with no tenderness. Genitourinary examination discloses an exquisitely tender left testicle that is situated in the transverse plane. The patient is fearful that this injury may cause him to be infertile. At this time it is most appropriate to inform the patient of which of the following?

(A) His fertility will be adversely affected because of loss of functional testicular tissue
(B) His fertility will most likely be unaffected following immediate reversal of the condition
(C) His fertility will not be adversely affected with conservative management
(D) Whether his fertility will be affected cannot be predicted without further evaluation and testing

97. A 39-year-old woman at 32 weeks' gestation returns to the office for a prenatal visit. She previously used intravenous heroin and has been in a methadone maintenance program for the past 2 years, including throughout this pregnancy. She admits to cigarette use and occasional cocaine and amphetamine use during the early part of this pregnancy. In the past 2 months she has tested negative for any drug use other than methadone. You have discussed with her the probability of a prolonged hospitalization of this neonate if there are signs of withdrawal after delivery. At this visit, she tells you that she wishes to breast-feed her baby. Which of the following is the most appropriate response?

(A) "Because of your high risk for HIV infection, breast-feeding is unsafe for your baby."
(B) "Because of your past history of drug abuse, breast-feeding is unsafe for your baby."
(C) "Because you have participated in a methadone maintenance program, I encourage you to breast-feed."
(D) "Many drugs enter the breast milk and can cause problems for the baby. Let's develop a plan to give your baby safe breast milk."
(E) "Methadone, cocaine, and amphetamines do not enter the breast milk in sufficient amounts to harm the baby, so breast-feeding can be permitted."

98. A 25-year-old nulligravid woman comes to the office for an annual health maintenance examination. She has a history of polycystic ovarian syndrome and has been taking an oral contraceptive to regulate her menses. Medical history is otherwise unremarkable. Family history is remarkable for obesity. She is 168 cm (5 ft 6 in) tall and weighs 67 kg (148 lb); BMI is 24 kg/m^2. Temperature is 37.0°C (98.6°F), pulse is 82/min, respirations are 16/min, and blood pressure is 126/76 mm Hg. Physical examination shows mild facial hirsutism that appears to have remained unchanged since her last visit 1 year ago. The remainder of the physical examination shows no abnormalities. The patient should be counseled that she is at greatest risk for acquiring which of the following conditions?

(A) Diabetes mellitus
(B) Hypertension
(C) Hypothyroidism
(D) Nephrotic syndrome
(E) Uterine cancer

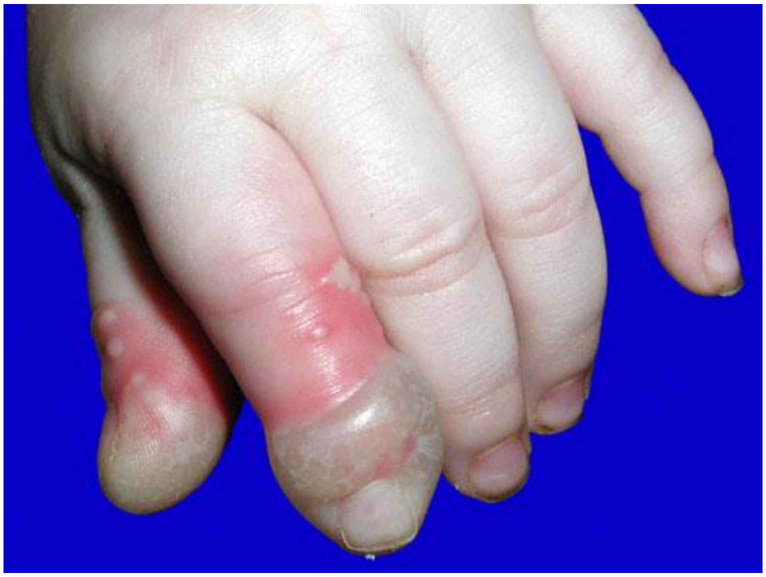

99. An 11-month-old infant is brought to the office by his mother because of a 24-hour history of progressive swelling and blistering of his left index finger and thumb. Two days ago, he developed fever and runny nose. Yesterday his mother noted sores on his left thumb and index finger, which have become larger and more blistered today. He has no known history of trauma. No one at home has similar lesions or history of skin problems. The infant attends day care 5 days weekly. Medical history is significant for recurrent diaper rashes and upper respiratory tract infections. Temperature is 38.8°C (101.8°F), pulse is 140/min, and respirations are 32/min. He appears nontoxic but fussy. Physical examination discloses rhinorrhea with clear mucus and several small erythematous papulovesicular lesions near his mouth. The left hand appears as shown. Which of the following is the most appropriate pharmacotherapy?

(A) Oral acyclovir
(B) Oral cephalexin
(C) Oral clindamycin
(D) Topical mupirocin
(E) Topical silver sulfadiazine

100. A 57-year-old woman comes to the office because of a 2-week history of intermittent palpitations. The palpitations are worse the morning of this visit and are associated with a "queasy feeling" in her upper abdomen. Her symptoms began after she started training for a 5-km race at the urging of her partner and have been increasing in intensity after each training session. Medical history is significant for type 2 diabetes mellitus treated with metformin. Family history is significant for myocardial infarction in her father. She has smoked one-half pack of cigarettes daily for the past 30 years; she does not drink alcoholic beverages. She is 152 cm (5 ft) tall and weighs 64 kg (141 lb); BMI is 28 kg/m^2. Temperature is 37.2°C (98.9°F), pulse is 98/min and irregular, respirations are 18/min, and blood pressure is 92/64 mm Hg. Oxygen saturation is 94% on room air. Auscultation of the lungs discloses bilateral basilar crackles. Cardiac examination discloses a normal S_1 and S_2, an S_4, and a grade 2/6 systolic murmur. The remainder of the physical examination discloses no abnormalities. ECG obtained today shows ST-segment elevation in the anterior leads and Q waves in leads II, III, and aVF; rhythm strip shows intermittent premature ventricular contractions. Which of the following is the most appropriate pharmacotherapy at this time?

(A) Intramuscular morphine sulfate
(B) Oral aspirin
(C) Oral diltiazem
(D) Subcutaneous enoxaparin
(E) Sublingual nitroglycerin

101. A 49-year-old woman is brought to the emergency department by her husband because of a 1-hour history of generalized weakness, dizziness, and palpitations. The patient says she has felt like she is going to faint. She also reports a 3-day history of an upper respiratory infection, for which she has taken over-the-counter cold remedies. Medical history is remarkable for hyperthyroidism diagnosed 1 month ago, for which she was given prescriptions for several medications, none of which she has filled. She is 160 cm (5 ft 3 in) tall and weighs 54 kg (119 lb); BMI is 21 kg/m^2. She appears anxious. Temperature is 38.9°C (102.0°F), pulse is 168/min and irregular, respirations are 26/min, and blood pressure is 124/56 mm Hg supine. Oxygen saturation is 97% on room air. Physical examination shows mild muscle wasting, mild pharyngeal erythema without exudate, and watery nasal discharge. Examination of the neck discloses a goiter, an audible bruit, and no jugular venous distention. Lungs are clear to auscultation with good breath sounds. Cardiac examination shows an irregularly irregular rhythm and a grade 3/6 systolic ejection murmur heard best along the left sternal border. The abdomen is soft with no tenderness. A tremor of the upper extremities is noted. There is no clubbing, cyanosis, or edema to any of the extremities. Neurologic examination shows no abnormalities. ECG shows atrial fibrillation with a rapid ventricular response. The patient is placed on a cardiac monitor and is given a 1 L bolus of isotonic saline. Which of the following is the most appropriate next step?

(A) Cardioversion
(B) Intravenous dexamethasone
(C) Intravenous diltiazem
(D) Intravenous propranolol
(E) Oral potassium iodide

102. **<u>Patient Information</u>**
Age: 20 years
Gender: M, self-identified
Race/Ethnicity: unspecified
Site of Care: student health center

The patient presents because of a 3-day history of burning on urination and penile discharge. He says he had unprotected sexual intercourse 1 week ago with a woman he met at a night club. He describes his usual state of health as excellent. Medical history is unremarkable. He takes no medications except for vitamin supplements and protein powder that he buys at the local health food store. He drinks two to three beers on weekends and smokes cannabis approximately once monthly. He is 185 cm (6 ft 1 in) tall and weighs 82 kg (180 lb); BMI is 24 kg/m^2. The patient appears embarrassed and uncomfortable. Temperature is 37.3°C (99.2°F), pulse is 80/min, respirations are 14/min, and blood pressure is 120/70 mm Hg. Physical examination discloses a circumcised penis with a whitish-yellow discharge at the urethral orifice. There are no penile lesions or ulcerations. Rapid HIV antibody test is negative. A swab of the urethral discharge is obtained and sent for culture. Antibiotic therapy is initiated for empiric treatment of chlamydia and gonorrhea, and the patient is notified that he will be contacted with the final results. Assuming that the patient's symptoms improve with the prescribed pharmacotherapy, which of the following is the most appropriate follow-up plan?

(A) Consult with the patient's parents before determining appropriate follow up
(B) Schedule follow-up examination in 1 week
(C) Schedule HIV serology testing in 3 months
(D) No specific follow up is necessary

103. A 36-year-old woman comes to the emergency department because of abdominal pain, nausea, and vomiting that began suddenly 12 hours ago and has worsened since that time. The patient rates the pain as 10 on a 10-point scale and describes it as stabbing and radiating to her back. She has not had hematemesis. Her last bowel movement was yesterday. She has never had a similar episode in the past. Medical history is unremarkable and she takes no medications. She drinks one to two alcoholic beverages weekly, usually on weekends. Temperature is 37.4°C (99.3°F), pulse is 116/min, respirations are 20/min, and blood pressure is 118/88 mm Hg. The patient appears uncomfortable. The conjunctivae are anicteric. Abdomen is mildly distended with guarding and tenderness to palpation. No telangiectasias are noted. Results of serum laboratory studies are shown:

Alkaline phosphatase	196 U/L
Bilirubin, total	1.8 mg/dL
Triglycerides	275 mg/dL
Lipase	846 U/L
Calcium	8.9 mg/dL
Urea nitrogen	16 mg/dL
Creatinine	1.8 mg/dL
Na^+	144 mEq/L
K^+	3.5 mEq/L
Cl^-	100 mEq/L
HCO_3^-	29 mEq/L
Glucose	125 mg/dL

In addition to beginning intravenous fluids, which of the following is the most appropriate next step in management?

(A) CT scan of the abdomen with contrast
(B) Fenofibrate therapy
(C) Imipenem cilastatin therapy
(D) Insulin therapy
(E) Ultrasonography of the right upper abdominal quadrant

104. A 4-month-old male infant is brought to the office by his parents because of a 1-day history of lethargy and constipation. The parents say the infant has not been breast-feeding well during the past 2 weeks and has not had a bowel movement in 2 days. He has not had fever and has not been exposed to anyone known to be ill. He was born at term to a 26-year-old woman, gravida 2, para 1, via uncomplicated spontaneous vaginal delivery. Birth weight was 3000 g (6 lb 10 oz; 25th percentile). Medical history is unremarkable and he receives no medications. Vaccinations are up-to-date. Family history is unremarkable. The patient is 59 cm (23 in; <3rd percentile) long and weighs 5.5 kg (12 lb; <3rd percentile); head circumference is 41 cm (16.1 in; 25th percentile). Temperature is 37.3°C (99.1°F), pulse is 116/min, respirations are 16/min, and blood pressure is 88/54 mm Hg. The patient is listless upon examination. Physical examination discloses weakness of the upper and lower extremities bilaterally against active resistance. Abdominal and rectal examinations disclose no abnormalities. Results of fasting laboratory studies are shown:

Serum		Urine	
Calcium	9.0 mg/dL	Specific gravity	1.012 (N<1.010)
Urea nitrogen	8 mg/dL	pH	5.5 (N=4.5−7.8)
Creatinine	0.4 mg/dL (N=0.03–0.5)	Glucose	2+
Na^+	133 mEq/L		
K^+	2.7 mEq/L		
Cl^-	105 mEq/L		
HCO_3^-	11 mEq/L		
Glucose	84 mg/dL (N=50–90)		
Phosphorus	2.5 mg/dL (N=3.8–8.2)		

Which of the following is most likely to develop in this patient?

(A) Congestive heart failure
(B) Cushing syndrome
(C) Osteomalacia
(D) Type 1 diabetes mellitus

105. **<u>Patient Information</u>**
Age: 44 years
Gender: F, self-identified
Ethnicity: unspecified
Site of Care: office

<u>History</u>
Reason for Visit/Chief Concern: "My neck and shoulder pain have come back again."

History of Present Illness:
• 5-year history of myofascial pain syndrome
• 12-month history of right-sided neck and shoulder pain; has not sustained any trauma
• rates current neck and right shoulder pain as 6/10
• daily stretching of the neck and right shoulder and naproxen therapy have not improved pain
• 6 months ago, received local anesthetic trigger point injection in the right upper trapezius muscle from another physician with complete resolution of pain for 4 months
• 48 hours after trigger point injection, developed severe itching and redness at injection site; symptoms resolved spontaneously
• informed she is allergic to local anesthetics but was not told which one was used in the injection
Past Medical History:
• 5-year history of major depressive disorder
• 10-year history of hypothyroidism
Medications:
• citalopram
• levothyroxine
• naproxen
Allergies:
• local anesthetics
Family History:
• mother: alive with fibromyalgia
Psychosocial History:
• does not smoke cigarettes or drink alcoholic beverages

<u>Physical Examination</u>

Temp	Pulse	Resp	BP	O_2 Sat	Ht	Wt	BMI
36.9°C (98.4°F)	77/min	15/min	117/67 mm Hg	98% on RA	163 cm (5 ft 4 in)	70 kg (155 lb)	27 kg/m^2

• Pulmonary: clear to auscultation
• Cardiac: regular rhythm; normal S_1 and S_2; no murmurs, rubs, or gallops
• Musculoskeletal: palpation of right upper trapezius muscle discloses a trigger point within a taut band of muscle that reproduces patient's neck and shoulder pain

<u>Question:</u> The patient requests a repeat trigger point injection. Which of the following is the most appropriate next step in management?

(A) Administer the injection using phenol as the anesthetic
(B) Administer the injection using prilocaine as the anesthetic
(C) Administer the injection using tetracaine as the anesthetic
(D) Pretreat the patient with loratadine, then administer the injection using any local anesthetic
(E) Refer the patient to an allergist for further evaluation

106. **Patient Information**
Age: 62 years
Gender: M, self-identified
Ethnicity: unspecified
Site of Care: hospital, post anesthesia care unit (PACU)

History
Reason for Admission/Chief Concern: postoperative knee pain 4 hours after undergoing total right knee arthroplasty

History of Present Illness:
- 2-year history of severe right knee pain
- patient is extubated postoperatively
- rates right knee pain 9/10
- knee pain difficult to control because administration of opioid medications causes sleepiness and episodes of apnea

Past Medical History:
- hypertension
- gastroesophageal reflux disease
- hypercholesterolemia
- hypothyroidism
- osteoarthritis

Medications:

• losartan	home, continued on admission
• hydrochlorothiazide	home, continued on admission
• omeprazole	home, continued on admission
• levothyroxine	home, continued on admission
• hydromorphone	initiated in PACU
• ketorolac	initiated in PACU
• acetaminophen	initiated in PACU

Allergies:
- no known drug allergies

Psychosocial History:
- drinks two cocktails daily
- does not smoke cigarettes or use any other substances
- retired school-teacher
- lives with his wife

Physical Examination

Temp	Pulse	Resp	BP	O_2 Sat	Ht	Wt	BMI
36.0°C	88/min awake	12/min awake	139/82 mm Hg awake	96% on 4LNC awake	178 cm	124 kg	39 kg/m^2
(96.8°F)	82/min sleeping	2/min sleeping	142/86 mm Hg sleeping	86% on 4LNC sleeping	(5 ft 10 in)	(273 lb)	

- Appearance: sleepy, but arousable to voice
- Pulmonary: decreased breath sounds at bases, otherwise clear to auscultation
- Cardiac: regular rhythm
- Extremities: dressing, wrap, and brace immobilization applied to right knee
- Neurologic: fully oriented

This item continues on the following page.

Question: Which of the following is the most appropriate next step to decrease this patient's risk for postoperative respiratory events?

(A) Administration of oxygen via high flow nasal cannula
(B) Bupivacaine femoral nerve block
(C) Chest wall impedance monitoring
(D) Continuous pulse oximetry
(E) Hydromorphone via patient-controlled analgesia

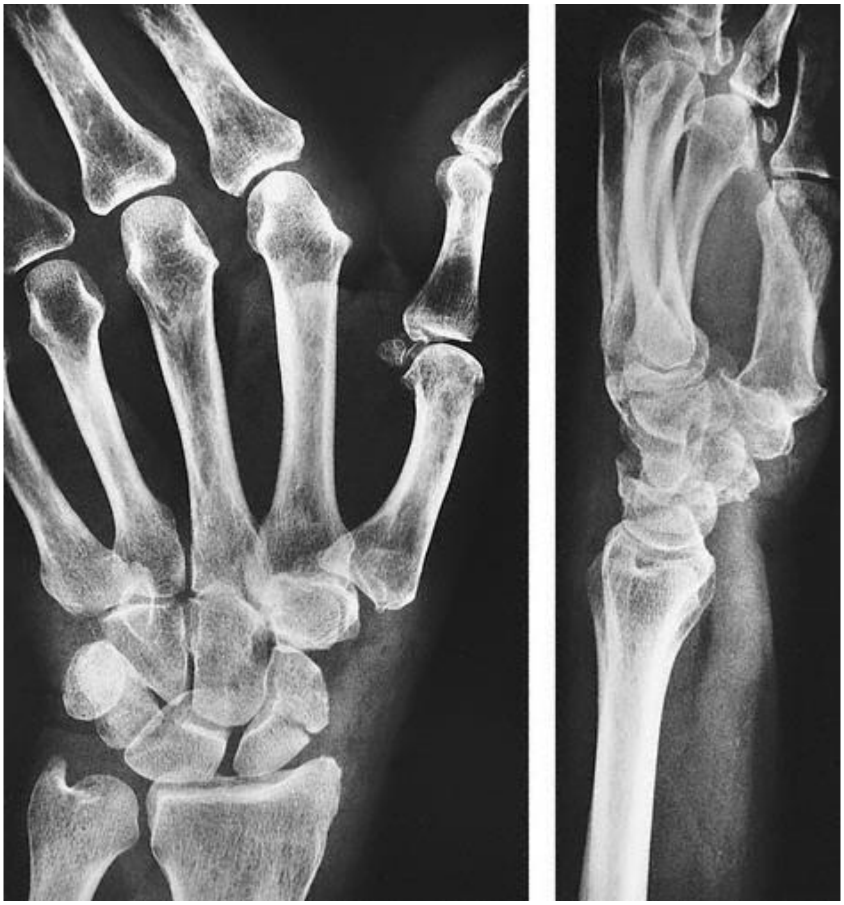

107. **<u>Patient Information</u>**
Age: 52 years
Gender: F, self-identified
Race/Ethnicity: Lebanese American, self-identified
Site of Care: office

The patient presents because of pain in her left wrist following a fall 1 week ago. She is a data entry clerk and you have been treating her for carpal tunnel syndrome for the past year. Physical examination today discloses little swelling, but there is pain with attempts to flex or extend the wrist and fingers. X-rays are shown. Which of the following is the most appropriate management?

(A) Apply an elastic wrist bandage
(B) Apply a plaster cast that incorporates the thumb
(C) Apply a plastic wrist splint
(D) Begin daily aspirin treatment and do not restrict use of the hand
(E) Refer her to an occupational therapist

NOTE: THIS IS THE END OF BLOCK 3.
ANY REMAINING TIME MAY BE USED TO CHECK ITEMS IN THIS BLOCK.

Block 4: ACM
Items 108–137; Time - 45 minutes

ALL ITEMS REQUIRE SELECTION OF ONE BEST CHOICE.

108. A 65-year-old woman who has been your patient for 8 years is being prepared for discharge from the hospital after receiving a prosthetic hip following a hip fracture. Postoperatively she received low-dose heparin therapy, and she has been able to move about with a walker. She has had diabetes mellitus for 12 years, which has been controlled with glipizide. Her hemoglobin A_{1c} 3 months ago was 7.6%. Other than the diabetes her health has been excellent and her weight has not changed in several years. She took estrogen for 4 years for hot flushes but discontinued it because of vaginal bleeding. She does not smoke cigarettes. She drinks a glass of wine or a bottle of beer daily. She lives with her 62-year-old sister, who has severe osteoarthritis but is able to prepare meals and assist her with activities of daily living. During this hospitalization the patient's blood glucose concentrations have ranged from 220 mg/dL to 280 mg/dL, for which she is being treated with insulin. She will continue insulin therapy at home. At this time it is most appropriate to arrange for home services to do which of the following?

(A) Administer daily insulin
(B) Assist the patient in building her upper-body strength
(C) Assist the patient in strengthening her lower extremity muscles
(D) Ensure that the patient follows the diabetic diet prescribed for her
(E) Measure the patient's blood glucose concentration daily

Items #109–110 are part of a sequential item set. In the actual examination environment, you will **not** be able to view the second item until you click "Proceed to Next Item." After navigating to the second item, you will not be able to add or change an answer to the first item.

A 32-year-old man comes to the office because of mild eye irritation, runny nose, nasal congestion, and postnasal drip that have occurred since he moved to his current apartment 10 months ago. He also reports occasional shortness of breath with wheezing and loss of his sense of smell. He has not had fever or cough. Medical history is unremarkable and he takes no medications. Family history is significant for nasal polyps in several family members who also have similar symptoms. The patient's vital signs are within normal limits. Physical examination discloses mildly injected conjunctivae, pale and swollen nasal turbinates, and a slightly injected pharynx. Lungs are clear to auscultation. The remainder of the examination discloses no abnormalities.

109. Which of the following factors in this patient's history most strongly predicts development of a more serious condition?

(A) Associated shortness of breath and wheezing
(B) Family history of similar symptoms
(C) Loss of sense of smell
(D) Perennial nature of symptoms

110. The patient is counseled regarding allergen avoidance, including the use of HEPA air filters, a mattress cover, and frequent vacuuming with dust removal. He returns to the office 1 month later with continued wheezing and shortness of breath. Which of the following is the most appropriate pharmacotherapy at this time?

(A) Inhaled fluticasone
(B) Nasal cromolyn
(C) Nasal oxymetazoline
(D) Oral fexofenadine

END OF SET

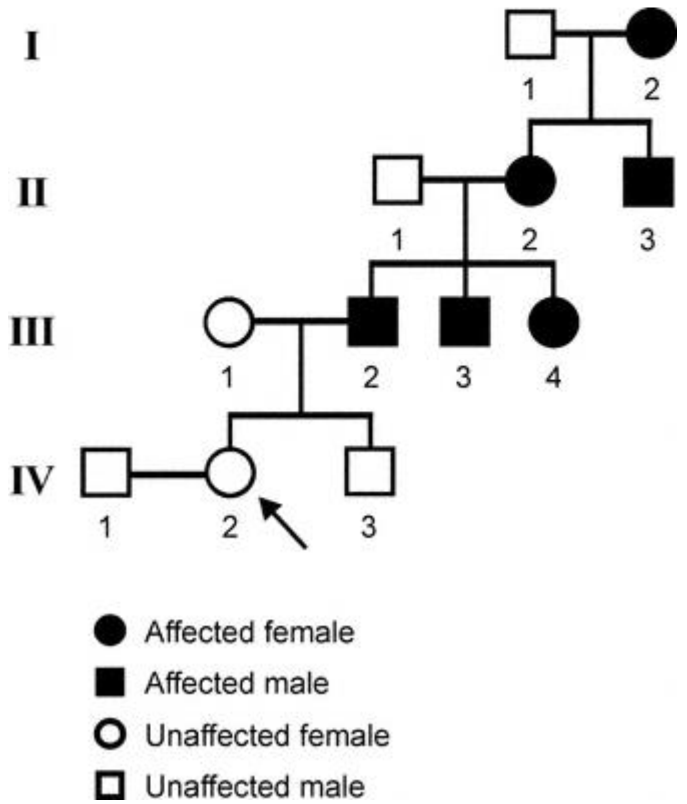

111. A 24-year-old woman comes to the office because she had a positive home pregnancy test 2 days ago. Urine pregnancy test in the office today is positive. Physical examination findings are consistent with a 14-week gestation. Ultrasonography shows a twin pregnancy with two female fetuses. This is her first pregnancy, and she says she is concerned because she has a family history of an eye disease in which family members lose their vision beginning in their 20s. Testing of her vision today discloses no abnormalities. The patient's pedigree is shown; the patient is identified as IV, 2. Which of the following is the most appropriate conclusion about the inheritance of this disorder?

(A) It is an autosomal dominant disorder
(B) It is a chromosomal aneuploidy
(C) It is indeterminant
(D) It is an X-linked dominant condition
(E) It is an X-linked recessive condition

112. An 84-year-old man, who has resided in a nursing care facility for the past 5 years, is being evaluated at the facility because a nurse discovered a sacral ulcer while bathing the patient 2 hours ago. The patient is not in pain. Medical history is significant for type 2 diabetes mellitus, congestive heart failure, chronic obstructive pulmonary disease, and peripheral vascular disease. Medications are insulin, furosemide, metoprolol, lisinopril, albuterol and ipratropium metered-dose inhalers, and 81-mg aspirin. The patient is unable to walk and spends the majority of the day in a manual wheelchair that he self-propels slowly with his feet. BMI is 19 kg/m^2. Temperature is 37.0°C (98.6°F), pulse is 62/min, respirations are 16/min, and blood pressure is 115/69 mm Hg. Oxygen saturation is 94% on oxygen at 2 L/min via nasal cannula. The patient appears frail. Lungs are clear to auscultation; breath sounds are decreased. Cardiac examination discloses no abnormalities. There is a 3 × 3 × 0.2-cm stage II ulcer on the patient's sacrum with yellowish exudate at the base and no surrounding erythema. Examination of the extremities shows edema to the calves bilaterally. Results of recent laboratory studies are shown:

Serum		Blood	
Urea nitrogen	10 mg/dL	Hematocrit	33%
Creatinine	1.1 mg/dL	Hemoglobin	10.8 mg/dL
Na^+	138 mEq/L	WBC	11,500/mm^3
K^+	4.0 mEq/L	Neutrophils	60%
Cl^-	100 mEq/L	Eosinophils	2%
HCO_3^-	24 mEq/L	Lymphocytes	33%
Cholesterol		Monocytes	5%
Total	105 mg/dL	Platelet count	135,000/mm^3

Which of the following treatment modalities is most likely to have the greatest effect on wound healing in this patient?

(A) Hypercaloric diet
(B) Pressure relief
(C) Silver sulfadiazine
(D) Wound debridement

113. A 36-year-old woman is referred to the office for evaluation of a fasting serum total cholesterol concentration of 249 mg/dL. She has a family history of early coronary artery disease (CAD) and her father died suddenly at age 46 years of myocardial infarction. She tells you that she has never had chest pain. She is not currently sexually active and has no children. The patient works as an advertising executive. She claims that her high-stress lifestyle makes it impossible for her to eat regular meals or to follow a special diet, and she usually eats fast food. She exercises two or three times a week for about 20 minutes on a treadmill. She has smoked one pack of cigarettes daily for the past 20 years. Her only medication is acetaminophen for tension headaches. She is 165 cm (5 ft 5 in) tall and weighs 76 kg (167 lb); BMI is 28 kg/m^2. Vital signs today are within normal limits. Physical examination discloses no abnormalities except for mild obesity. Institution of which of the following is the most essential step in the prevention of CAD in this patient?

(A) Biofeedback-based stress reduction program
(B) More rigorous and consistent exercise program
(C) Smoking cessation program
(D) Strict low-calorie diet
(E) Strict low-fat diet

114. A 21-year-old woman is brought to the emergency department by her parents after she told them she consumed an entire bottle of an unspecified pain medication 3 hours ago in a suicide attempt. The patient reports shortness of breath and ringing in her ears. Medical history is significant for major depressive disorder. Her only routine medication is escitalopram and she has no allergies. Temperature is 37.5°C (99.5°F), pulse is 96/min, respirations are 22/min, and blood pressure is 98/60 mm Hg. Oxygen saturation is 92% on room air. The patient appears tired and thin but is not in acute distress. She is slow to respond to questions and has difficulty following instructions. Skin is warm and dry. Examination of tympanic membranes discloses no abnormalities. Auscultation of the lungs discloses bilateral basilar crackles. Cardiac examination discloses no abnormalities. Abdomen is soft and nontender. The remainder of the physical examination discloses no abnormalities. Results of arterial blood gas analysis on room air are shown:

P_{O_2}	88 mm Hg
P_{CO_2}	26 mm Hg
pH	7.32

Which of the following is the most appropriate next step in management?

(A) Administration of lipid emulsion
(B) Administration of *N*-acetylcysteine
(C) Administration of sodium bicarbonate
(D) Endotracheal intubation
(E) Supportive care only

115. A 38-year-old woman comes to the office for an annual health maintenance examination. She has been healthy and her last cervical cytology 3 years ago was normal. The patient has been married for the past 15 years and the couple is monogamous. Today, the patient is afebrile. Pulse is 82/min and blood pressure is 130/82 mm Hg. Physical examination shows no abnormalities. Cervical cytology and human papillomavirus (HPV) testing are obtained. If results from the most recent cervical cytology and HPV test are normal, at which of the following times should this patient have her next cervical cytology?

(A) At the time of menopause
(B) In 1 year
(C) In 5 years
(D) Only if she gets pregnant
(E) The patient does not need another cervical cytology

116. A 21-year-old man, who is a member of the US Air Force, comes to the emergency department because of a 2-day history of shortness of breath on exertion and when lying down, as well as fever, chills, and swelling of his legs. Medical history is unremarkable and he takes no medications. Temperature is 38.1°C (100.5°F), pulse is 97/min, respirations are 20/min, and blood pressure is 140/91 mm Hg. Oxygen saturation is 92% on room air. The patient is dyspneic but able to speak in full sentences. Jugular venous pressure is 14 cm H_2O. Auscultation of the lungs discloses bilateral basilar crackles. Cardiac examination discloses a regular rhythm with an S_3 gallop but no murmur. Abdominal examination discloses no abnormalities. There is 1+ pitting edema of the lower extremities. Results of laboratory studies show a serum B-type natriuretic peptide concentration of 2000 pg/mL (N<100) and a leukocyte count of 13,000/mm^3. Results of serum chemistry profile and remainder of complete blood count are within the reference ranges. ECG shows no abnormalities except for nonspecific ST-segment changes. Echocardiography shows a dilated left ventricle with an ejection fraction of 0.40 and no pericardial effusion. Which of the following is the most likely long-term outcome for this patient?

(A) Complete recovery
(B) Mild diastolic dysfunction
(C) Pulmonary embolism
(D) Recurrent pericarditis
(E) Severe systolic heart failure requiring cardiac transplant

117. A 31-year-old woman who is in the US Air Force comes to the office because of a 1-day history of fever, sore throat, body aches, muscle weakness, and dehydration. Medical history is remarkable for systemic lupus erythematosus. She has no known allergies. Current medications are calcium, hydroxychloroquine, iron, and vitamin D. She has a 5-year-old son who currently has a febrile illness and a rash on his hands and feet. The patient's BMI is 24 kg/m^2. Temperature is 38.3°C (101.0°F), pulse is 96/min, respirations are 20/min, and blood pressure is 115/77 mm Hg. Physical examination shows an erythematous throat with vesicles on both tonsils. There is no ulceration or coating of the tongue. There is bilateral cervical lymphadenopathy. The lungs are clear. A rapid streptococcal test result is negative. Which of the following is the most appropriate treatment for this patient?

(A) Acyclovir
(B) Amoxicillin
(C) Amoxicillin-clavulanic acid
(D) Ibuprofen
(E) Prednisone

118. A 45-year-old woman comes to the office because of a 6-month history of progressively slurred speech. During this time, she has bitten her tongue frequently and has had difficulty swallowing food. She also has felt clumsy performing normal activities. She has not had changes in vision, sensory symptoms, or bladder dysfunction. Five years ago, she underwent cervical fusion for degenerative disc disease. She takes no medications. Vital signs are within normal limits. The patient is unable to protrude her tongue. There is deep furrowing and bilateral fasciculations of the tongue. She is dysarthric. When the patient is asked to say, “Ahh,” there is symmetric but incomplete elevation of the palate. Muscle tone is normal throughout. Muscle strength is 4/5 in the right upper extremity, and there are fasciculations in the right deltoid and biceps; muscle strength is 5/5 elsewhere. Deep tendon reflexes are 1+ in the right biceps and brachioradialis and 3+ in the triceps and right lower extremity. Electromyography and nerve conduction studies show motor denervation in the tongue and right extremities. Which of the following factors in this patient’s history most negatively impacts her life expectancy?

(A) Age
(B) Bulbar weakness
(C) Cervical fusion operation
(D) Fasciculations
(E) Gender

119. A 64-year-old woman comes to the clinic because of a 2-year history of constipation. She initially had bowel movements every other day and passed hard stools. During the past 4 months, she has had three bowel movements weekly. Fiber supplementation during the past 3 months has not increased the frequency of bowel movements. She has not had abdominal pain, fever, weight loss, or blood in the stool. She has hypertension, and her only medication is lisinopril. Colonoscopy 5 years ago and cervical cytology 3 years ago showed no abnormalities. There is no family history of colon cancer or bowel disorders. The patient does not appear to be in distress. She is 163 cm (5 ft 4 in) tall and weighs 66 kg (145 lb); BMI is 25 kg/m^2. Vital signs are within normal limits. The abdomen is soft, nontender, and nondistended. Bowel sounds are normal. Rectal examination shows decreased sphincter tone; there are no masses. Soft stool is noted in the rectal vault. Test of the stool for occult blood is negative. Which of the following is the most appropriate next step in management?

(A) Addition of bisacodyl to the medication regimen
(B) Anorectal manometry
(C) Colonoscopy
(D) Discontinuation of lisinopril
(E) Recommendation to maintain a daily bowel movement journal for 1 month

120. A 72-year-old man is brought to the emergency department by emergency medical services approximately 45 minutes after losing consciousness while attempting to stand from a seated position while watching television at home. He regained consciousness spontaneously after 5 minutes. He has had persistent light-headedness since this episode but no other symptoms. He has felt light-headed during postural changes in the past, but he has never before lost consciousness. Medical history is remarkable for ischemic cardiomyopathy with a left ventricular ejection fraction of 40%. Medications are daily metoprolol, lisinopril, spironolactone, atorvastatin, and 81-mg aspirin, as well as furosemide every other day. There have been no changes in his medication regimen during the past year. Temperature is 35.8°C (96.4°F), pulse is 30/min, respirations are 24/min, and blood pressure is 75/35 mm Hg. Oxygen saturation is 90% on room air. The patient is diaphoretic and pale. He occasionally does not respond appropriately to questions or commands, but he is oriented to person, place, and time. Skin is cool to the touch. Auscultation of the lungs discloses bilateral crackles. Which of the following is the most appropriate next step in management?

(A) Adenosine therapy
(B) Continuous infusion of dobutamine
(C) Continuous infusion of epinephrine
(D) Defibrillation
(E) Intubation and mechanical ventilation
(F) Transcutaneous pacing

121. A 25-year-old man comes to the clinic to request vaccination against human papillomavirus (HPV). Medical history is unremarkable. He takes no medications. He is sexually active with one male partner and uses condoms consistently. He is a member of the US Marine Corps. BMI is 21 kg/m^2. Vital signs are within normal limits. Physical examination shows no abnormalities. Which of the following is the most appropriate next step in management?

(A) Administer the HPV vaccine
(B) Obtain HPV serologic testing
(C) Perform an anal Pap smear for cytologic examination
(D) Use motivational interviewing to encourage abstinence

122. A 55-year-old woman with long-standing hypertension comes to the office because of a 5-month history of fatigue that worsens when she walks upstairs or exerts herself. At least three times weekly she has shortness of breath requiring the use of three pillows to sleep at night. She has not had chest pain. She has been an established patient for 10 years. During this time her hypertension had been well controlled with hydrochlorothiazide therapy; however, 2 months ago she developed increased blood pressure and lisinopril was added to her medication regimen. Medical history also is significant for mitral valve regurgitation that was evaluated by a cardiologist 3 years ago, at which time the patient was asymptomatic. BMI is 28 kg/m^2. Temperature is 36.7°C (98.0°F), pulse is 80/min, respirations are 16/min, and blood pressure is 150/85 mm Hg. Lungs are clear to auscultation. Cardiac examination discloses a diminished S_1, an audible S_3, and a high-pitched grade 4/6 systolic murmur radiating to the left axilla. Examination of the extremities shows 1+ pitting edema to the middle of the tibia bilaterally. Which of the following is the most appropriate next step in management?

(A) Add amlodipine to her medication regimen
(B) Evaluate her for valve replacement
(C) Increase the doses of hydrochlorothiazide and lisinopril
(D) Recommend a low-sodium diet that includes low-fat dairy products and fresh fruits and vegetables
(E) Recommend starting a low-impact aerobic exercise regimen for 30 minutes daily

123. A 68-year-old man is admitted to the hospital because of a 2-month history of gradually worsening dyspnea and swelling of the lower extremities. Medical history also is remarkable for obstructive sleep apnea. The patient takes no medications. BMI is 40 kg/m^2. On admission, temperature is 37.0°C (98.6°F), pulse is 84/min, respirations are 28/min, and blood pressure is 142/78 mm Hg. Oxygen saturation is 89% on room air. Auscultation of the lungs discloses decreased breath sounds on the right. Chest percussion discloses dullness and decreased fremitus. There is bilateral pedal edema. Chest x-ray shows a pleural effusion. Ultrasonography-guided thoracentesis is done and discloses serosanguineous fluid; results of laboratory studies are shown:

Serum		Pleural fluid	
Lactate dehydrogenase	189 U/L	pH	7.33
Protein		Lactate dehydrogenase	169 U/L
Total	5.3 g/dL	Protein	
		Total	3.9 g/dL

Cultures and cytology of the pleural fluid are negative. Which of the following is the most appropriate next step in management?

(A) Bronchoscopy
(B) CT scan of the chest
(C) Echocardiography
(D) Furosemide therapy
(E) Moxifloxacin therapy

124. A 46-year-old woman comes to the office for a routine health maintenance examination. She reports no symptoms. Medical history is remarkable for polycystic kidney disease, hypertension, type 2 diabetes mellitus, and hypothyroidism. The kidney disease was diagnosed 5 years ago; CT scan at that time showed enlarged kidneys with extensive cysts bilaterally. Medications are rosuvastatin, lisinopril, glyburide, and levothyroxine. BMI is 32 kg/m^2. Temperature is 37.0°C (98.6°F), pulse is 88/min, respirations are 16/min, and blood pressure is 138/82 mm Hg. Oxygen saturation is 100% on room air. Cardiopulmonary examination discloses no abnormalities. Muscle strength is 5/5 in all extremities and deep tendon reflexes are 2+ throughout all extremities. Results of fasting laboratory studies are shown:

Serum		Plasma	
Cholesterol		Copeptin	2.0 pmol/L (N=1–12)
Total	220 mg/dL	Urine	
HDL	82 mg/dL	Na^+	25 mEq/L (N<25)
LDL	110 mg/dL		
Triglycerides	140 mg/dL		
Urea nitrogen	30 mg/dL		
Creatinine	2.1 mg/dL		
Na^+	140 mEq/L		
K^+	4.0 mEq/L		
Cl^-	102 mEq/L		
HCO_3^-	26 mEq/L		
Uric acid	3.0 mg/dL		

Which of the following findings most strongly indicates a poor prognosis in this patient?

(A) BMI
(B) Kidney function
(C) Number of kidney cysts
(D) Plasma copeptin concentration
(E) Serum LDL concentration
(F) Urine sodium concentration

125. A 48-year-old woman is referred to the office because a fingerstick blood glucose concentration measured at a health fair 4 days ago was 200 mg/dL. Medical history is unremarkable and she takes no medications. BMI is 30 kg/m^2. Temperature is 37.0°C (98.6°F), pulse is 84/min, respirations are 16/min, and blood pressure is 129/79 mm Hg. The patient is not in acute distress. Physical examination discloses no abnormalities. Results of fasting laboratory studies obtained in preparation for today's visit are shown:

Serum		Blood	
Glucose	146 mg/dL	Hemoglobin A_{1c}	8.3%
		Urine	
		Microalbumin	<30 mg/24 h

The patient is counseled regarding lifestyle modifications. In addition to metformin, which of the following is the most appropriate pharmacotherapy?

(A) 81-mg Aspirin
(B) Chlorthalidone
(C) Lisinopril
(D) Metoprolol
(E) No additional pharmacotherapy is indicated

126. A 65-year-old man who has been your patient for 5 years comes to the office because he has noticed slowly progressive deformity of his left lower leg. He says, "My leg seems to be a lot more bowed than I remember; it didn't look this way last year." On further questioning, he reports that he has had aching pain in his left leg and hip for the past month, and that he has been taking ibuprofen daily for pain relief without much effect. He identifies as Swedish American. Physical examination is normal with the exception of valgus deformity of the left lower leg, with pain on palpation over the tibial shaft. X-rays of the lower leg show cortical thickening and intramedullary sclerosis in the tibia. Which of the following is the most appropriate pharmacotherapy?

(A) Alendronate, orally
(B) Calcitonin, intramuscularly
(C) Calcitonin, nasally
(D) Mithramycin, intravenously
(E) Naproxen, orally

127. A 46-year-old man with a 7-year history of spondylolisthesis and resulting chronic low back pain comes to the office for a refill of his sustained-release oxycodone. He reports persistent back pain for the past 2 months and requests an increase in the dose of oxycodone. He has had two previous unsuccessful back surgeries. The patient's friend, who drove him to the office, informed the nurse that he thinks the patient has been selling his oxycodone on the street. The patient's medical history is otherwise unremarkable and he takes no other medications. Vital signs are within normal limits. Physical examination discloses reproducible pain over the low back but no obvious muscle spasms. There is a well-healed surgical scar over the lumbar spine. Straight-leg raising test is negative bilaterally. The remainder of the physical examination, including neurologic examination, discloses no abnormalities. Which of the following is the most appropriate next step?

(A) Discontinue oxycodone
(B) Notify the police
(C) Order a random urine test for oxycodone
(D) Refer the patient to a methadone clinic
(E) Switch oxycodone to a different pain medication

128. A 40-year-old woman, gravida 2, para 2, comes to the office because of a 1-month history of weakness and intermittent diarrhea. She also reports frequent palpitations and says she has felt shaky and warm during the past 2 weeks. She has lost 9 kg (20 lb) during the past 2 months and was initially excited about the weight loss. Medical history is remarkable for two cesarean deliveries at ages 20 and 22 years. She takes no medications. Family history is remarkable for glaucoma diagnosed in her maternal grandfather at age 65 years. The patient does not smoke cigarettes, drink alcoholic beverages, or use other substances. She is 173 cm (5 ft 8 in) tall and weighs 54 kg (120 lb); BMI is 18 kg/m^2. She appears anxious and is sweating mildly. Temperature is 37.4°C (99.4°F), pulse is 115/min, respirations are 18/min, and blood pressure is 140/90 mm Hg. Physical examination discloses bilateral lid lag and exophthalmos. There is mildly diffuse enlargement of the thyroid with no discrete palpable nodules. Cardiac examination discloses a regular rate and rhythm. There is a fine resting tremor of both hands and hyperreflexia in all extremities. Results of laboratory studies show a serum thyroid-stimulating hormone concentration of 0.2 μU/mL. Without treatment, which of the following is most likely to develop in this patient?

(A) Chronic kidney disease
(B) Congestive heart failure
(C) Fibromyalgia
(D) Glaucoma
(E) Multiple sclerosis

129. A 32-year-old woman, gravida 2, para 2, who is 3 months post partum and breast-feeding, is admitted to the hospital because of a warm, tender, erythematous lump in her right breast that has increased in size during the past week despite applied heat and cephalexin therapy. She has repeatedly pumped milk from that breast but her symptoms have not improved. The patient's pregnancy was complicated by preterm labor, for which she was hospitalized for 1 week prior to giving birth via vaginal delivery at 32 weeks' gestation. Medical history is otherwise unremarkable. She takes a prenatal vitamin and is allergic to sulfa-containing medications. On admission, the patient appears flushed. Temperature is 38.4°C (101.1°F), pulse is 114/min, respirations are 18/min, and blood pressure is 120/76 mm Hg. Physical examination discloses a warm, 10 × 10-cm, erythematous, tender mass in the upper outer quadrant of the right breast. The mass is not fluctuant. Several enlarged, tender lymph nodes are palpable in the right axilla. Ultrasonography-guided aspiration of the mass is scheduled. Which of the following is the most appropriate pharmacotherapy at this time?

(A) Intravenous ampicillin-sulbactam
(B) Intravenous cefazolin
(C) Intravenous piperacillin-tazobactam
(D) Intravenous vancomycin
(E) Oral dicloxacillin

130. A 34-year-old man is referred to the office by his new employer because of a positive PPD skin test. Medical history is remarkable for three episodes of scabies during the past year. He previously harvested asparagus at a local farm but says he recently acquired a position at the local nursing home; part of the required examination was to have the PPD skin test. His test result was positive with 15 mm of induration. Chest x-ray shows no abnormalities. His 32-year-old wife and six children, who range in age from 12 years to 16 months, live in the same house. The patient is started on isoniazid therapy. Which of the following is the most appropriate next step?

(A) Administer PPD skin tests to the whole family
(B) Obtain interferon gamma release assay
(C) Order sputum cultures and gastric washings for the whole family
(D) Schedule another chest x-ray in 3 months
(E) Start the patient's children on isoniazid therapy

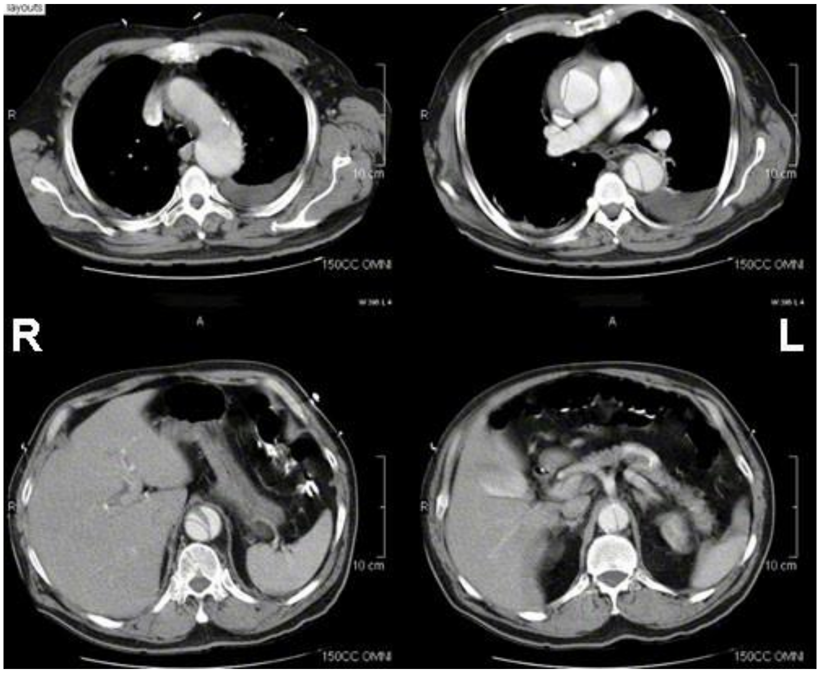

131. **Patient Information**
Age: 62 years
Gender: M, self-identified
Race/Ethnicity: African American, self-identified
Site of Care: emergency department

History
Reason for Visit/Chief Concern: "I passed out suddenly at home."

History of Present Illness:
• collapsed while making breakfast; quickly regained consciousness according to his wife
• does not recall any precipitating factors or symptoms prior to the episode
• the fall was not witnessed, but his wife found him within 1 minute of hearing him fall and called for ambulance
• has chest and back pain that does not radiate
• pain is constant and "crushing"
• pain rated as 10/10
• unable to get comfortable; nothing alleviates the pain
Past Medical History:
• hypertension
Medications:
• amlodipine
Allergies:
• no known drug allergies
Psychosocial History:
• drinks one to two alcoholic beverages daily
• does not smoke cigarettes

This item continues on the following page.

Physical Examination

Temp	Pulse	Resp	BP	O_2 Sat	Ht	Wt	BMI
36.4°C (97.5°F)	104/min	20/min	80/38 mm Hg	94% on RA	183 cm (6 ft)	100 kg (220 lb)	30 kg/m^2

- Appearance: alert; visibly uncomfortable
- HEENT: PERRL; ocular movements intact; oral mucosa pink and moist
- Pulmonary: shallow breath sounds
- Cardiac: tachycardia; diastolic murmur
- Abdominal: nondistended, nontender to palpation
- Extremities: radial pulses 1+; femoral pulses thready bilaterally
- Neurologic: fully oriented; cranial nerves grossly intact; no focal deficits

Diagnostic Studies
- ECG: nonspecific ST-segment and T-wave changes
- CT angiography of the chest: shown

Question: Which of the following is the most appropriate next step in management?

(A) Coronary angiography
(B) CT scan of the head
(C) Norepinephrine infusion
(D) Placement of an intra-aortic balloon pump
(E) Replacement of the aortic root

132. A 63-year-old man is brought to the emergency department by his wife because of a 2-day history of double vision, feeling off-balance, difficulty walking, and leaning to his right side while standing. He also has had sore throat, hoarseness, difficulty swallowing and talking, and fatigue during this time. He had severe stabbing pain on the right side of his face a few minutes before his other symptoms began. The severity of his symptoms has been constant since onset. The patient is right-hand dominant but he has had difficulty accurately using his right arm during this time. When he tries to eat, his right forearm and hand do not accurately guide food to his mouth. He has not had a fever. He has hypertension treated with lisinopril. Temperature is 37.2°C (99.0°F), pulse is 75/min, respirations are 16/min, and blood pressure is 148/98 mm Hg. Oxygen saturation is 98% on room air. On examination, the patient leans to the right while seated. The left pupil measures 6 mm and constricts to 4 mm in response to light; the right pupil measures 4 mm and constricts to 3 mm in response to light. There is no conjunctival icterus. There is horizontal and rotational nystagmus on right gaze. The fast phase of nystagmus is to the left. There is mild right ptosis. The patient can hear finger rubs and whispers bilaterally. The right soft palate does not elevate with phonation; the patient's voice is hoarse, and he has dysarthric speech. Peripheral pulses are 2+ throughout. Muscle strength, bulk, and tone are normal in all extremities. Deep tendon reflexes are 1+ throughout. Babinski sign is absent bilaterally. Sensation to pain and temperature is decreased over the right side of the face, the left extremities, and the left side of the trunk. Finger-nose testing shows dysmetria on the right only. Gait is broad based, and the patient leans to the right while walking. Based on these findings, this patient is most likely to develop which of the following?

(A) Achalasia and gastroparesis
(B) Angle-closure glaucoma
(C) Orthostatic hypertension
(D) Sleep-related hypoventilation
(E) Third-degree atrioventricular block

133. **Patient Information**
Age: 75 years
Gender: F, self-identified
Ethnicity: unspecified
Site of Care: hospital

History
Reason for Visit/Chief Concern: "I've felt tired for a while and I've been bruising easily."

History of Present Illness:
• 3-week history of progressively worsening bruising and fatigue
• gradual onset of symptoms
• no fever, cough, urinary symptoms, chest pain, dyspnea, orthopnea, paroxysmal nocturnal dyspnea, or lower extremity edema
Past Medical History:
• 5-year history of hyperthyroidism
• 10-year history of hyperlipidemia
• 15-year history of coronary artery disease
Medications:
• carvedilol
• lisinopril
• atorvastatin
• methimazole
• daily aspirin
Allergies:
• no known drug allergies
Family History:
• father: deceased at age 56 years from myocardial infarction
Psychosocial History:
• does not smoke cigarettes, drink alcoholic beverages, or use substances or non-prescribed drugs

Physical Examination

Temp	Pulse	Resp	BP	O_2 Sat	Ht	Wt	BMI
37.8°C (100.0°F)	63/min	14/min	135/78 mm Hg	96% on RA	155 cm (5 ft 1 in)	61 kg (135 lb)	26 kg/m^2

• Appearance: in no distress; alert
• HEENT: oropharynx without exudate
• Pulmonary: clear to auscultation
• Cardiac: normal S_1 and S_2 with no murmurs, rubs, or gallops
• Abdominal: bowel sounds normoactive; soft, nontender
• Extremities: no edema or tenderness
• Neurologic: oriented to person, place, and time

This item continues on the following page.

Diagnostic Studies:

Serum		Blood	
Na^+	137 mEq/L	Hematocrit	28%
K^+	4.3 mEq/L	Hemoglobin	9.3 g/dL
Cl^-	110 mEq/L	WBC	1200/mm^3
HCO_3^-	22 mEq/L	Platelet count	43,000/mm^3
Urea nitrogen	12 mg/dL	Urine	
Creatinine	1.2 mg/dL	Specific gravity	1.010 (N=1.003–1.029)
Albumin	3.6 g/dL	Leukocyte esterase	Negative
		Nitrite	Negative
		WBCs	0/hpf
		RBCs	0/hpf

• ECG: sinus rhythm without ST changes
• chest x-ray: no abnormalities

Question: Which of the following medications is the most likely cause of this patient's symptoms?

(A) Aspirin
(B) Atorvastatin
(C) Carvedilol
(D) Lisinopril
(E) Methimazole

134. A 26-year-old man is brought to the emergency department 30 minutes after his roommate found him confused at home with a bump on the left side of his head and fresh urine on the floor. On arrival, the patient is confused but his mental status returns to normal shortly after arrival. The patient is a medical student, and the last thing he remembers is coming home from class 30 minutes before he was found. He has not had dizziness, blurred vision, fever, or nausea. Medical history is remarkable for bacterial meningitis 5 years ago, an anterior cruciate ligament injury sustained while playing basketball 10 years ago, and unilateral renal agenesis with proteinuria. His only medication is lisinopril. He stayed awake all night twice during the past week to study for final examinations. He works the night shift at a fast-food restaurant on weekends. He has smoked one-half pack of cigarettes daily for 5 years. He drinks four 12-oz beers weekly. He uses cannabis three times weekly. He usually drinks one to two cups of coffee daily but has been drinking 48 oz of coffee daily for the past 2 days. BMI is 27 kg/m^2. Temperature is 36.9°C (98.5°F), pulse is 88/min, respirations are 16/min, and blood pressure is 110/70 mm Hg. Oxygen saturation is 96% on room air. Physical examination shows blood in the mouth and bite marks on the left lateral aspect of the tongue. The remainder of the physical examination, including a neurologic examination, discloses no abnormalities. Results of serum laboratory studies are shown:

Urea nitrogen	20 mg/dL
Creatinine	0.8 mg/dL
Na^+	134 mEq/L
K^+	4.8 mEq/L
Cl^-	104 mEq/L
HCO_3^-	22 mEq/L
Glucose	88 mg/dL

Urine toxicology screening results are negative, and blood alcohol concentration is 0 mg/dL. MRI of the brain and EEG disclose no abnormalities. In addition to decreasing his caffeine intake, which of the following recommendations is most likely to prevent recurrent seizures in this patient?

(A) Alcohol avoidance
(B) Discontinuation of lisinopril
(C) Maintenance of adequate sleep
(D) Occupation change
(E) Tobacco cessation

135. A 33-year-old woman is admitted to the hospital because of a 3-day history of fever, shortness of breath, and progressive cough productive of dark yellow sputum. She felt well before the onset of symptoms. Use of cough suppressant and acetaminophen has provided no relief. She has a 5-year history of myasthenia gravis. Her current medications are pyridostigmine and an oral contraceptive. Temperature is 38.9°C (102.0°F), pulse is 104/min, respirations are 16/min, and blood pressure is 138/72 mm Hg. Oxygen saturation is 94% on oxygen at 4 L/min by nasal cannula. She is alert and appears fatigued. She coughs intermittently throughout the examination. Mucous membranes are dry. Pulmonary examination discloses crackles and rhonchi in the left upper lung field. The remainder of the examination shows no abnormalities. X-ray of the chest shows a patchy infiltrate in the left upper lobe. Which of the following is the most appropriate pharmacotherapy for this patient at this time?

(A) Amoxicillin-clavulanic acid
(B) Azithromycin
(C) Cefepime and tobramycin
(D) Ceftriaxone and doxycycline
(E) Levofloxacin
(F) Trimethoprim-sulfamethoxazole

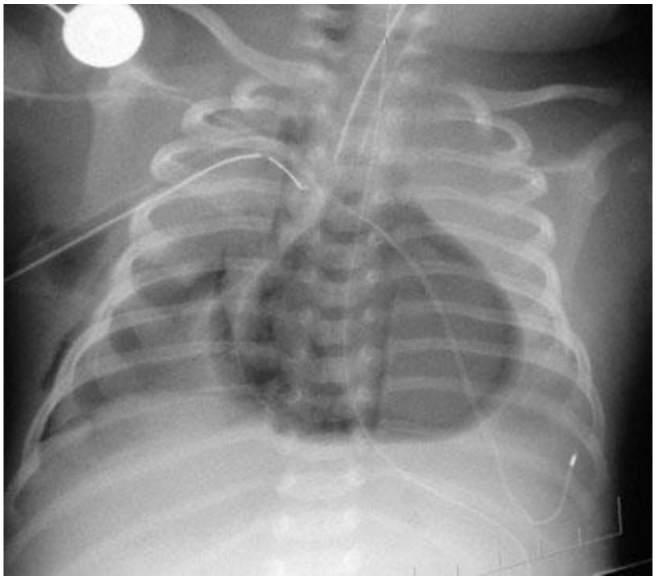

136. A 2-week-old male newborn, who was delivered at 34 weeks' gestation, is being urgently evaluated because the nurses have reported recent decreases in his oxygen saturation and blood pressure. The newborn was diagnosed with respiratory distress syndrome shortly after his birth and he has required continued adjustments to his mechanical ventilation settings, which now show a positive end-expiratory pressure (PEEP) of 10 cm H_2O, a ventilator rate of 50/min, a peak inspiratory pressure of 32 cm H_2O, and an FIO_2 of 0.80. The patient weighs 1600 g (3 lb 9 oz). Now temperature is 36.8°C (98.2°F), pulse is 200/min, respirations are 50/min, and palpable blood pressure is 22 mm Hg. Oxygen saturation is 78%. Physical examination shows cyanosis and poor perfusion to the extremities. Breath sounds are present bilaterally. A fluid bolus is initiated intravenously. Chest x-ray ordered 15 minutes ago is obtained and is shown. Which of the following is the most appropriate management of this patient's condition?

(A) Insertion of a central venous catheter
(B) Median sternotomy
(C) PEEP reduction to 6 cm H_2O
(D) Pericardiocentesis
(E) Placement of a left chest tube

137. A 14-year-old boy is brought to the office by his parents because of a 3-month history of fatigue, recurrent abdominal pain, and 7-kg (15-lb) weight loss. He rates the abdominal pain at its worst as a 6 on a 10-point scale and notes that the pain is associated with loose bowel movements. His stool has not contained mucus or blood. The symptoms have caused him to miss approximately 20 days of school during the past semester. In addition, the patient has developed two to three recurrent mouth ulcers. He has not had any rashes, chest pain, difficulty breathing or swallowing, or joint symptoms, though his heels ache slightly when he walks. Medical history is unremarkable and he takes no medications. He is 150 cm (4 ft 11 in; 5th percentile) tall and weighs 36 kg (80 lb; 3rd percentile). Vital signs are normal. Auscultation of the heart discloses an S_1 and normally split S_2, and no murmur. Abdominal examination discloses mild distention, diffuse tenderness to palpation, and mild fullness in the right lower quadrant. Nail beds appear normal. There is no peripheral edema or arthritis. Neurological examination shows no focal findings. Which of the following is the most likely complication of this patient's condition?

(A) Anal fistula
(B) Colonic carcinoma
(C) Diabetes mellitus
(D) Progressive pulmonary failure
(E) Toxic megacolon

NOTE: THIS IS THE END OF BLOCK 4.
ANY REMAINING TIME MAY BE USED TO CHECK ITEMS IN THIS BLOCK.

Answer Key for USMLE Step 3 Sample Questions

Block 1: FIP

1. B
2. C
3. A
4. C
5. C
6. D
7. A
8. A
9. C
10. B
11. C
12. D
13. D
14. D
15. D
16. B
17. B
18. D
19. B
20. C
21. C
22. B
23. B
24. A
25. E
26. C
27. B
28. C
29. D
30. E
31. B
32. A
33. F
34. B
35. A
36. A
37. B
38. C

Block 2: FIP

39. E
40. D
41. D
42. D
43. D
44. B
45. C
46. E
47. A
48. B
49. D
50. A
51. E
52. B
53. D
54. C
55. E
56. E
57. B
58. B
59. A
60. B
61. E
62. A
63. E
64. C
65. B
66. B
67. B
68. D
69. C
70. F
71. C
72. A
73. D
74. B
75. E
76. D
77. C

Block 3: ACM

78. A
79. C
80. E
81. C
82. A
83. C
84. A
85. A
86. D
87. C
88. D
89. B
90. B
91. E
92. F
93. D
94. E
95. A
96. B
97. D
98. A
99. A
100. B
101. D
102. C
103. E
104. C
105. E
106. B
107. B

Block 4: ACM

108. C
109. A
110. A
111. C
112. B
113. C
114. C
115. C
116. A
117. D
118. B
119. B
120. F
121. A
122. B
123. B
124. B
125. E
126. A
127. C
128. B
129. D
130. A
131. E
132. D
133. E
134. C
135. D
136. D
137. A
